# U.S. FDA Regulatory Monitoring of Ochratoxin A in Human Foods: 2008-2022

**DOI:** 10.14252/foodsafetyfscj.D-25-00002

**Published:** 2026-06-26

**Authors:** Tabitha J. Miller, Anthony Adeuya, Lauren P. Robin

**Affiliations:** Office of Food Chemical Safety, Dietary Supplements and Innovation, Human Foods Program, U.S. Food and Drug Administration, 5001 Campus Drive, College Park, Maryland 20740, USA

**Keywords:** mycotoxins in human foods, ochratoxin A in human foods

## Abstract

Ochratoxin A (OTA) is a naturally occurring mycotoxin produced by *Aspergillus* and *Penicillium* fungi that can contaminate a range of agricultural commodities and products, such as cereal grains, dried fruit, and coffee beans. While some OTA contamination in foods may be unavoidable, there are steps that can be taken to help prevent fungal growth and control the presence of OTA in foods. The U.S. Food and Drug Administration (FDA) has established a regulatory mycotoxin compliance program to sample and analyze human foods for mycotoxins. Each year, under this compliance program, samples of foods susceptible to mycotoxin contamination are collected and analyzed for various mycotoxins, including OTA. In this study, we present and discuss data on OTA levels for over 3,700 food samples collected between fiscal years (FY) 2008 and 2022. OTA levels ranged from non-detect to 116 µg/kg, with the highest findings seen in green coffee beans, dried fruits, grain products, and spices. The vast majority of samples (96.5%) had OTA levels below the levels of quantification (LOQs) of 0.21 to 24.9 µg/kg. The findings were similar to detection rates, levels, and affected foods seen in other surveys of OTA. Our findings support continued monitoring of OTA in susceptible foods and may be useful in future work to determine whether it is appropriate to establish regulatory levels for OTA in the United States.

## Introduction

Ochratoxin A (OTA) is a naturally occurring mycotoxin found in a variety of foods worldwide, such as cereal grains, coffee beans, spices, cocoa beans, pistachios, and grapes. OTA is a secondary metabolite produced by different *Aspergillus* and *Penicillium* fungi, such as *Aspergillus ochraceus* and *Pencillium verrucosum.*^1^^,^^2^^)^ Infestation of agricultural commodities by these fungi can occur at any time during the growth, harvest, and storage stages, with contamination during storage occurring most frequently.^2^^)^ Fungal growth is influenced by factors such as temperature, pH, moisture content, and water activity (a_w_).^3^^)^ Different OTA-producing fungal species have different optimal conditions for growth, with some preferring hot and dry climates and other species thriving in temperate climates.^4^^)^ As a result, OTA contamination occurs in a broad range of commodities from different geographical origins.

Mitigating OTA contamination in foods is important because OTA has been linked to renal cancer in various animal studies and may be linked to kidney disease in humans (Balkan endemic nephropathy).^5^^,^^6^^,^^7^^,^^8^^,^^9^^)^ Both international and national organizations have classified OTA as a possible carcinogen, with the International Agency for Research on Cancer (IARC) classifying OTA as possibly carcinogenic to humans (Group 2B) based on limited evidence of carcinogenicity in animals^6^^)^ and the U.S. National Toxicology Program classifying OTA as reasonably anticipated to be a human carcinogen.^10^^)^

The Joint FAO/WHO Expert Committee on Food Additives (JECFA) identified a provisional tolerable weekly intake (PTWI) for OTA of 100 ng/kg body weight (bw)/week based on nephrotoxicity. JECFA also concluded that exposures, based primarily on European data, were well below the PTWI.^11^^)^ Likewise, the Government of Japan Food Safety Commission identified a tolerable daily intake (TDI) of 16 ng/kg bw/day for non-carcinogenic effects and 15 ng/kg bw/day for carcinogenic effects and concluded that intake of OTA in Japan is below the TDI even in high-risk consumers.^12^^)^ The European Food Safety Authority (EFSA) calculated BMDL_10_s for non-neoplastic (4.73 μg/kg bw/day) and neoplastic (14.5 μg/kg bw/day) endpoints. EFSA identified possible health concerns for non-neoplastic effects for high consumers in younger age groups, and possible health concerns for neoplastic effects across all age groups, but only if there is a direct mechanism for genotoxicity.^5^^)^

Good agricultural, manufacturing, and storage practices can help prevent and control OTA contamination in foods. Codex Alimentarius, an international standard setting organization, has developed three OTA-specific and three general mycotoxin-related codes of practice (CoPs) that provide advice on prevention and reduction of mycotoxins including OTA in food. The three OTA-specific CoPs address OTA in cocoa,^13^^)^ coffee,^14^^)^ and wine,^15^^)^ while the general mycotoxins CoPs cover mycotoxins in spices,^16^^)^ cassava and cassava-based products,^17^^)^ and cereals.^18^^)^

In addition to CoPs, some countries and global organizations have set regulatory limits for OTA in food, as summarized in Table 1. (see **Table S1** for full details). The European Union (EU) has set maximum levels (MLs) for OTA for commodities ranging from foods intended for infants to roasted coffee to licorice, with levels ranging from 0.5 µg/kg to 80 µg/kg.^19^^)^ Codex Alimentarius has established recommended MLs of 5 µg/kg OTA for wheat, barley, and rye, and 20 µg/kg for chili pepper, paprika, and nutmeg.^20^^)^ Other countries, such as Switzerland and Morocco, have adopted levels similar to or less stringent than the EU levels (Table 1**.** and **Table S1**).^19^^,^^21^^,^^22^^,^^23^^,^^24^^,^^25^^)^

**Table 1.** Selected global maximum levels of OTA (µg/kg)*

*Food Category*	*Brazil* ^21^^)^	*Codex* ^20^^)^	*EU* ^19^^)^	*Morocco* ^22^^)^	*United Kingdom* ^23^^)^	*Singapore* ^24^^)^	*Switzerland* ^25^^)^
*Cereal grains*	10	5	3-5	3-5	5	5	5
*Breakfast cereals*	10	–	2-4	3	3	3	2-4
*Bakery products*	10	–	2-4	3	3	–	2-4
*Infant cereals or cereal-based foods for infants*	2	–	0.5	0.5	0.5	0.5	0.5
*Instant coffee*	10	–	5	10	10	10	5
*Roasted coffee*	10	–	3	5	5	5	3
*Spices*	30	20	15-20	15-20	15-20	20	10-20
*Chocolate/cocoa*	5-10	–	3	–	–	–	3
*Dried fruit*	10	–	2-8	10	10	10	2-8
*Nuts*	–	–	5-10	–	–	–	5-10
*Pulses*	10	–	–	–	–	–	5

*See **Table S1** for more details about individual food categories

The U.S. Food and Drug Administration (FDA) has not set a maximum level for OTA in foods; however, the agency provides a referral level of 20 µg/kg for FDA mycotoxin-servicing laboratories to identify findings they should submit for case-by-case safety assessment to determine if the OTA levels pose a human health concern. Collection of samples of foods of both domestic and import origin for OTA analysis occurs as part of the FDA Mycotoxins in Human Foods Compliance Program.^26^^)^ In addition to identifying samples of concern, the collection of occurrence data through the mycotoxin compliance program can serve to inform future decisions regarding future work on OTA in foods.

Separate from the mycotoxin compliance program sampling activities, FDA scientists and affiliates have also conducted research studies of OTA in retail food in the United States, as summarized in Table 2**.**^27^^,^^28^^,^^29^^)^ These studies focused on foods for infants and toddlers and found low levels of OTA in a small proportion of the samples (69 of 532 samples, 13%). Other surveys^30^^,^^31^^,^^32^^,^^33^^)^ of U.S. retail foods including breakfast cereals, dried fruits, nuts, and beverages, found OTA most frequently in oat-based cereals and raisins.^32^^)^ A 2017 risk assessment by Mitchell et al. of U.S. occurrence data concluded that there was negligible risk to the U.S. population from OTA exposure.^33^^)^

**Table 2.** Occurrence data of OTA in foods from selected national surveys and global systematic reviews

*Country*	*Foods*	*Detections of total samples*	*Range of OTA (µg/kg)*	*LOQ (µg/kg)*	*Reference*
**National Surveys**					
*USA*	Infant cereals	19 of 64	1.0 - 14.4	0.5	^27^
*USA*	Powdered infant formula (milk- and soy-based)	0 of 98	-	0.25	^28^
	Infant cereals	47 of 155	0.6 - 22.1	0.5	^28^
*USA*	Infant and toddler foods	1 of 147	2	0.6	^29^
	Breakfast cereals	2 of 68	0.5 - 2	0.6	^29^
*USA*	Breakfast cereal and snacks	75 of 144	0.10 - 7.43	0.032 - 0.10	^30^
*USA*	Breakfast cereals	205 of 489	0.10 - 9.30	0.03	^31^
*USA*	Dried fruits and nuts	57 of 665	0.28 - 890	0.25	^32^
*USA*	Wine, coffee, cocoa, and pork products	20 of 860	0.1 - 18.0	NR	^33^
*JAPAN*	Various retail foods	544 of 1358	up to 12.5	0.01 to 0.05	^34^
*JAPAN*	Various retail foods	120 of 192	0.010 - 12.5	0.004 - 0.1	^35^
*JAPAN*	Various retail foods	69 of 261	0.01 - 12.5	0.01 - 0.1	^36^
*JAPAN*	Wheat	329 of 782	<0.15 - 5.20	0.15	^37^
**Global Systematic Reviews**					
	Raw cereals	545 of 1896	0 - 1164		^38^
	Processed foods	758 of 1937	0.01 - 112		^38^
	Coffee	1778 of 3256	<0.03 - 136.9	0.04 - 3	^39^
	Spices	0 to 100%	up to 907.5		^40^

Table 2 also includes data from surveys of retail foods^34^^,^^35^^,^^36^^)^ and wheat^37^^)^ in Japan, as well as data from systematic global reviews.^38^^,^^39^^,^^40^^)^ While Japan has no regulatory levels for OTA in food, the levels reported across Japanese surveys were relatively low. Globally, OTA has been found in high levels in some foods, particularly in raw cereals and spices such as capsicums, highlighting the need for continued monitoring of OTA in food.

This paper will discuss 15 years of OTA data collected through FDA’s compliance program and how those data compare to international surveys of OTA in food. We also present information on international MLs for OTA in food and compare FDA findings to international MLs.

## Materials and Methods

A total of 3708 samples (1268 domestic and 2440 import) of OTA-susceptible food products were collected by FDA inspectors under the FDA mycotoxin compliance program from October 1, 2007, to September 30, 2022 (FY08-FY22). Samples (217) from one laboratory were excluded because records of LOQ values for older samples were not available. These samples were analyzed for OTA in FDA Office of Regulatory Affairs (ORA) laboratories following guidelines for OTA analysis in the Compliance Policy Guidance Manual for Mycotoxins in Domestic and Imported Foods, Part IV – Analytical.^26^^)^ Foods chosen for collection and analysis included baby foods (e.g., soya infant formula, dry infant cereals), dried beans, cereal grains and related processed foods (e.g., flour, cornmeal, bakery goods), dried fruits (e.g., raisins, dried figs), coffee beans, and spices (e.g., chili powder, paprika, ginger). Sampling for the compliance program is not designed to be statistically representative of the market share of these foods in the United States, or of the consumption patterns of the U.S. population; rather, sampling is designed to target commodities most susceptible to mycotoxin contamination as well as from geographical regions where mycotoxin contamination has been a known concern based on previous years’ data.

The data were uploaded to an Amazon Quicksight dashboard to assist in data analysis, data categorization, and preparation of visuals.^41^^)^ We analyzed the following characteristics by year and over the time span (15 years) of this retrospective study: percent quantified samples, mean/median quantified level, and range of quantified levels. Quantified samples are defined as samples with levels of mycotoxins above the level of quantification (LOQ). Trace samples are those with levels of mycotoxins above the level of detection (LOD) but below the LOQ. Non-detects are defined as a level of mycotoxins below the LOD. The LOQs ranged from 0.21 to 24.9 µg/kg; it should be noted that 96.7% of the LOQs were ≤ 5 µg/kg. Statistical analysis showed no trends in LOQs when compared to types of matrices, analytical laboratories, or year.

FDA analytical laboratories are ISO/IEC 17025 accredited and participate in analytical testing of externally issued quality control samples as required by ISO/IEC 17025.^42^^)^ Analytical methods are validated according to FDA guidelines prior to implementation.^43^^)^

## Results

During the 15-year period of this analysis, 3,708 food samples were analyzed for OTA and the findings are summarized in **Table 3****.** A total of 131 samples, or 3.5%, had levels of OTA above the LOQ. For these 131 samples, the LOQs ranged from 0.233 to 24.9 µg/kg.

**Table 3.** FDA results of ochratoxin A analysis of various commodities during FY08-FY22.

**Food Category**	**Commodity**	**N**	**> LOQ (%)**	**Mean (µg/kg)**	**Median (µg/kg)**	**Range(µg/kg)**	**Codex ML (µg/kg)**	**>Codex ML (%)**	**EU ML (µg/kg)**	**>EU ML (%)**
**Baby Food Products**	Baby Cereals	128	1 (0.8%)	1.20	1.20	1.20			0.5	1 (0.7%)
	Baby Formula	37	0						0.5	–
	Other Baby Foods	3	0						0.5	–
	**Total**	**168**	**1 (0.6%)**	**1.20**	**1.20**	**1.20**				**1 (0.6%)**
**Dried Beans and Other Pulses**	Black Beans	28	1 (3.6%)	4.35	4.35	4.35				
	Blackeye Peas	50	1 (2.0%)	3.60	3.60	3.60				
	Garbanzo Beans	82	1 (1.2%)	7.82	7.82	7.82				
	Lentils	39	0							
	Other Pulses	310	3 (1.0%)	13.7	12.6	3.20 - 25.2				
	Peas	34	3 (8.8%)	11.1	8.60	5.40 – 19.4				
	Pinto Beans	59	1 (1.7%)	6.00	6.00	6.00				
	Soybeans	83	0						5	–
	**Total**	**685**	**10 (1.5%)**	**9.62**	**6.91**	**3.20 – 25.2**				
**Candy**	**Total**	**13**	**0**							**–**
**Coffee**	**Total**	**514**	**14 (2.7%)**	**16.8**	**10.6**	**1.20 – 116**			3*	**10 (1.9%)**
**Fruit**	Dried Figs	12	1 (8.3%)	1.25	1.25	1.25			8	–
	Raisins	189	24 (12.7%)	10.2	5.22	1.10 - 49.2			8	10 (5.3%)
	Other Fruit Products	9	1 (11.1%)	42.4	42.4	42.4			2	1 (11.1%)
	**Total**	**210**	**26 (12.4%)**	**11.1**	**5.22**	**1.10 – 49.2**				**11 (5.2%)**
**Grains and Grain Products**	Barley	283	9 (3.2%)	10.8	6.90	1.10 - 44.0	5^	5 (1.8%)	3 or 5^#^	7 (2.5%)
	Buckwheat	100	7 (7.0%)	11.9	3.47	2.10 - 50.1			3 or 5^#^	4 (4.0%)
	Corn	238	6 (2.5%)	5.59	2.06	1.60 - 15.7			3 or 5^#^	2 (0.8%)
	Oats	164	13 (7.9%)	7.42	6.26	1.90 - 17.4			3 or 5^#^	9 (5.5%)
	Other Grains	37	0						3 or 5^#^	–
	Rice	21	0						3 or 5^#^	–
	Rye	71	2 (2.8%)	5.31	5.31	5.01 - 5.61	5^	–	3 or 5^#^	2 (2.8%)
	Wheat	602	14 (2.3%)	9.72	4.85	1.81 - 35.8	5^	3 (0.5%)	3 or 5^#^	9 (1.5%)
	**Total**	**1516**	**51 (3.4%)**	**8.97**	**5.70**	**1.10 – 50.1**		**8 (0.5%)**		**33 (2.2%)**
**Nuts and Edible Seeds**	Pistachios	42	1 (2.4%)	9.50	9.50	9.50			5	1 (2.4%)
	Other Nuts and Edible Seeds	14	0						5	–
	**Total**	**56**	**1 (1.8%)**	**9.50**	**9.50**	**9.50**				**1 (1.8%)**
**Processed Food Products**	Bread	20	0						2	–
	Breakfast Foods	216	2 (0.9%)	2.37	2.37	1.20 – 3.54			2	1 (0.5%)
	Other Bakery Products	42	1 (2.4%)	1.70	1.70	1.70			2	–
	Other Processed Foods	196	1 (0.5%)	1.03	1.03	1.03			3	–
	**Total**	**474**	**4 (0.8%)**	**1.87**	**1.45**	**1.03 – 3.54**				**1 (0.2%)**
**Spices**	Capsicums, including Paprika	18	10 (55.6%)	15.0	9.98	1.30 - 49.7	20	2 (11.1%)	20	2 (11.1%)
	Ginger	20	6 (30.0%)	5.17	5.74	1.50 - 8.10			15	–
	Nutmeg	4	3 (75.0%)	25.9	20.2	4.68 – 52.9	20	2 (50%)	15	2 (50%)
	Other Spices	16	4 (25.0%)	10.7	10.8	2.20 – 19.1			15	1 (6.3%)
	**Total**	**58**	**23 (39.7%)**	**13.1**	**8.10**	**1.30 – 52.9**		**4 (26.7%)**		**5 (8.6%)**
**Other products**	**Other**	**14**	**1 (7.1%)**	**1.43**	**1.43**	**1.43**				
**TOTAL**		**3708**	**131 (3.5%)**	**10.67**	**6.00**	**1.03 - 116**		**12 (0.3%)**		**62 (1.7%)**

* The EU ML of 3 µg/kg in coffee applies specifically to roasted coffee (not including instant coffee). FDA collects green, unroasted coffee beans for sampling. ^The Codex MLs for barley, rye, and wheat applies only to the raw whole commodity. ^#^The EU has a ML of 3 µg/kg for all products, derived/processed from unprocessed cereals and for cereals placed on the market for the final consumer, with a higher ML of 5 µg/kg for unprocessed cereal.

### Baby Food Products

Out of 168 baby food products analyzed for OTA, including dry infant cereal, soya baby formula, and other baby foods such as teething biscuits and mixed ingredient baby purees, only 1 sample (0.6%) - a multigrain (wheat, oat, rice, rye, and barley) baby cereal - had a quantifiable level of OTA, 1.20 µg/kg. The EU has a ML of 0.5 µg/kg for baby food and processed cereal-based food for infants and young children, which this sample would exceed. Codex Alimentarius has no MLs for OTA in foods intended for infants.

### Dried Beans and Other Pulses

There were 685 total samples of dried beans and other pulses collected for OTA analysis. Of the 10 samples where OTA was detected at quantifiable levels, there were black beans (1), blackeye peas (1), garbanzo beans (1), peas (3), pinto beans (1), and other pulses (3). The levels of OTA in these samples ranged from 3.20 to 25.2 µg/kg, with a mean level of 9.62 µg/kg. The only commodity in this category for which Codex or the EU has set an ML is soybeans, for which the EU has an ML of 5 µg/kg. Of the 83 soybean samples collected, none had a quantifiable level of OTA.

### Candy

There were 13 candy samples collected in the 15-year time span of the study. No samples had a level of OTA above the LOQ. No Codex or EU ML exists for candy, with the exception of licorice candy in the EU. U.S. sampling focused on the collection of cocoa containing products.

### Coffee Beans

Of 514 samples of coffee beans analyzed for OTA, 14 had levels of OTA above the LOQ. The levels in these 14 samples ranged from 1.20 to 116 µg/kg, with a mean level of 16.8 µg/kg. Codex has not set an ML for OTA in coffee. The EU has an ML for OTA in roasted coffee of 3 µg/kg and in instant coffee of 5 µg/kg. FDA collection of coffee for OTA analysis primarily focused on the collection of green coffee beans rather than finished product. While 10 of the 14 quantified samples were above the EU ML of 3 µg/kg for roasted coffee, it is reported that roasting of green coffee beans may reduce the levels of OTA by 65 to 100%.^14^^)^

### Fruit

Of the 210 samples of fruit products collected for OTA analysis, 189 were raisins, 12 were dried figs, and the remainder consisted of other fruit products, such as grape juice, dried currants, prunes, and dried oleasters. All but 2 of the 26 samples with quantifiable levels of OTA were raisins. The levels of OTA measured in these 26 samples ranged from 1.10 to 49.2 µg/kg, with a mean level of 11.1 µg/kg. The EU has set a ML of 8 µg/kg for dried vine fruit, such as raisins, and for dried figs, and an ML of 2 µg/kg for other dried fruits. Ten of the 24 FDA raisin samples with quantifiable OTA levels had levels above the EU ML of 8 µg/kg. One sample of dried oleasters (a fruit similar to olives) had a level of OTA above the 2 µg/kg ML for other dried fruits.

### Grains and Grain Products

The grains and grain products category, which includes whole raw grains, processed grains (e.g., husked or polished rice), and milled grain products like flours, cracked grains, and grain germ, consisted of 1,516 samples. Of those 1,516 samples, 602 (39.7%) were wheat, 283 (18.7%) were barley, 238 (15.7%) were corn, 164 (10.8%) were oats, and the remainder were buckwheat, rice, rye, and other grains. Of the grain samples, 540 were whole grain, while 976 were processed or milled grains. Quantifiable levels of OTA were found in all of the grain categories except for the category of rice and the category of other grains. There were 51 samples with levels of OTA above the LOQ. For whole grain samples (25), measured levels ranged from 1.10 to 44.0 µg/kg, with a mean of 9.47 µg/kg. For processed/milled grains (26 samples), levels ranged from 1.60 to 50.1 µg/kg, with a mean level of 8.48 µg/kg. Both Codex and the EU have set levels for OTA in grains. Codex has an ML of 5 µg/kg for raw wheat, barley, and rye; 8 of 12 quantified samples of wheat, barley, and rye were above the Codex ML. The EU has an ML of 3 µg/kg for all products, derived/processed from unprocessed cereals and for cereals placed on the market for the final consumer, with a higher ML of 5 µg/kg for unprocessed cereal. Of the 26 quantified samples of processed/milled grains, 16 were above the EU ML of 3 µg/kg, while 17 of the 25 quantified samples of whole grains were above the EU ML of 5 µg/kg.

### Nuts and Edible Seeds

Of the 56 nuts and edible seeds collected for OTA analysis, only 1 sample (pistachios) had OTA levels above the LOQ, at 9.50 µg/kg. Overall, 42 of the nut samples were pistachios. There is no Codex ML for OTA in nuts, but the EU has an ML of 5 µg/kg for certain edible seeds and pistachios on the market for the final consumer. The single quantified sample was above the EU ML.

### Processed Food Products

The processed food products category consists of processed foods such as bread, other bakery products, breakfast foods (e.g., breakfast cereal, flavored oatmeal), and other processed foods, such as pasta, corn chips, tortillas, and pretzels. There were 474 foods sampled in this category, with only 4 having quantifiable levels of OTA. These levels ranged from 1.03 to 3.54 µg/kg, with a mean level of 1.87 µg/kg. Codex has no ML set for OTA in these types of commodities, but the EU has set MLs of 2-3 µg/kg for OTA in bakery wares, cereal snacks, and breakfast cereals. Of the 4 quantified samples, only 1 breakfast food sample had an OTA level above the 2 µg/kg ML, in an overnight oats mix sample; the 3 other foods (muffin, corn flakes, and instant oatmeal) had levels below 2 µg/kg.

### Spices

Of the 58 spice samples collected, 18 were capsicums (including paprika), 20 were ginger, 4 were nutmeg, and the remainder were other spices such as pepper and turmeric. These 58 samples consisted of both powdered (46) and whole (12) spices and were primarily imported (38 imported; 20 domestic). Domestic samples primarily consisted of capsicums (10) and ginger (7). There were 23 samples with OTA detected above the LOQ. These ranged from 1.30 to 52.9 µg/kg, with a mean level of 13.1 µg/kg. Codex has an ML of 20 µg/kg OTA for chili pepper, paprika, and nutmeg; there were 2 capsicums and 2 nutmeg samples – all ground – above the Codex ML. Likewise, the EU has an ML of 20 µg/kg for capsicums and an ML of 15 µg/kg for other spices and spice mixtures. There were 2 capsicums, 2 nutmeg, and 1 turmeric sample above the EU MLs.

### Other Products

There are an additional 14 samples that do not fall in any other category. Most (13) did not contain OTA above the LOQ; one sample, a soy-based alternative protein, had an OTA level above the LOQ at 1.43 µg/kg.

## Discussion

With the exception of a small percentage (3.5%) of products, the samples collected and analyzed for OTA by the FDA during this 15-year period have been either trace or non-detect. One limitation of the analysis is that the remaining 96.5% of non-detect and trace samples were associated with LOQs ranging from 0.21 to 24.9 µg/kg (96.7% were below 5 µg/kg). It is possible that the rate of detection would have been higher if the LOQs were uniformly low. Adoption of a multi-mycotoxin method with an LOQ of 2.5 µg/kg starting in FY25 should provide a more accurate picture of OTA exposure from U.S. foods.

Of those samples with quantifiable levels (131 samples), sample findings ranged from 1.03 to 116 µg/kg, broken down as follows: dried beans and other pulses, 3.20 – 25.2 µg/kg; fruit, 1.10-49.2 µg/kg; grains and grain products, 1.10-50.1 µg/kg; coffee, 1.20-116 µg/kg; processed food products, 1.03-3.54 µg/kg; and spices, 1.30 – 52.9 µg/kg. For product categories with single detections (baby food products, candy, and nuts and edible seeds), findings ranged from 1.20 µg/kg to 9.50 µg/kg. In addition, 47.3% of samples with quantifiable concentrations (62 samples, 1.7% of all samples) had OTA levels above the relevant EU ML, and 9.2% of samples with quantifiable concentrations (12 samples, 0.3% of all samples) had OTA levels above the relevant Codex ML; however, not all foods with quantified levels had a Codex or EU level for comparison. Only 17 samples (0.4%) had levels above the FDA referral level of 20 µg/kg; 15 of the 17 samples were imported products. Of these 15 imported products, 6 samples were determined to be violative based on safety assessments and shipments were refused entry.

As with global data (Table 2.), our data show similar trends with certain food categories and commodities having higher levels of detection, notably fruit (e.g., raisins) (12.7%), oats (7.9%), and spices (39.7%). The highest levels detected in spices for FDA were for capsicums (49.7 µg/kg) and nutmeg (52.9 µg/kg), which aligns with the findings reported in Pickova et al (2020), where the highest level of OTA in spices were for capsicum chili (907.5 µg/kg) and nutmeg (60.7 µg/kg).^40^^)^

In terms of types of samples collected, FDA’s compliance program focused on those foods most susceptible to OTA contamination. The largest category collected was grains and grain products, with 1516 samples (40.9% of all samples). The second and third largest categories were dried beans and other pulses with 685 samples (18.5% of all samples) and coffee beans with 514 samples (13.9% of all samples), respectively. This breakdown aligns with foods most often identified as OTA-susceptible in other surveys, with grains and grain-based products being the most frequently sampled food type.^27^^,^^28^^,^^29^^,^^30^^,^^31^^,^^32^^,^^33^^,^^34^^,^^35^^,^^36^^,^^37^^,^^38^^,^^39^^,^^40^^)^

Overall, there were roughly twice as many imported foods collected as domestic foods (2,440 imported, 1,268 domestic). The decision to collect more imported foods than domestic foods is not representative of the market share of foods in the United States or the consumption pattern of the average U.S. consumer; rather it reflects observations over time of imported foods being more likely to have higher levels of mycotoxin contamination. The rate of samples with OTA levels above the LOQ was similar for import and domestic: 3.5% for imports, 3.6% for domestic. The two categories with the highest rates of OTA levels above the LOQ were spices (39.7%) and fruit (12.4%), with only fruit having a higher rate for imports (14.3% vs 10.2%).

## Conclusion

Over the 15-year period from October 1, 2008, to September 30, 2022, FDA collected and analyzed 3,708 foods for OTA as part of its mycotoxin compliance program. This is the first time a large set of data for OTA findings in foods in the United States has been published by FDA. The vast majority of the foods sampled (96.5%) had no quantifiable levels of OTA. Of the 3.5% of samples with detectable levels, 9.2% had levels above the relevant Codex ML (12 samples, 0.3% of all samples) and 47.3% had levels above the relevant EU MLs (62 samples, 1.7% of all samples). Overall, the FDA findings appear similar to those seen in other U.S.-based and global surveys of OTA in susceptible commodities.

The OTA occurrence data presented here highlight that while most foods have no quantifiable levels of OTA, there is a need to continue monitoring the food supply for these contaminants. OTA is unfortunately an unavoidable contaminant for agriculture, but there are steps to control the growth of fungi and limit mycotoxin load, such as pre-harvest measures like the use of fungicides and post-harvest measures like sorting harvested crops to remove moldy or damaged grains and nuts and seeds. This OTA dataset could be useful in future work to determine whether it is appropriate to identify an ML for OTA in the United States. Typically, FDA has used action levels in guidance documents^44^^)^ to recommend MLs for mycotoxins.^45^^,^^46^^,^^47^^,^^48^^)^

In addition, as research into mycotoxins continues to reveal new and emerging mycotoxins and new data become available on co-occurrence of mycotoxins in a single commodity, the FDA looks to modernize its regulatory strategy and methods. Many of the foods susceptible to ochratoxin A are also susceptible to other types of mycotoxins, such as aflatoxins. The FDA recently updated its Compliance Policy Guide Manual (CPGM) to reflect the adoption of a new multi-mycotoxin analytical method by FDA laboratories,^26^^)^ which is published in the Foods Program Compendium of Analytical Laboratory Methods: Chemical Analytical Manual (CAM) on the FDA website.^49^^,^^50^^)^ It will allow FDA to monitor foods for co-occurrence of ochratoxin, aflatoxin, deoxynivalenol, T-2 and HT-2 toxins, and zearalenone. Co-occurrence data of OTA and other mycotoxins in foods will allow FDA to better understand mycotoxin exposure from the U.S. food supply and to make better informed decisions to protect public health.

## Supplementary materials
