# Supplementary material for: U.S. FDA Regulatory Monitoring of Ochratoxin A in Human Foods: 2008-2022
Source: Food Saf (Tokyo). 2026 Jun 26;14(2):48–57. doi: 10.14252/foodsafetyfscj.D-25-00002 (PMC13310607; doi:10.14252/foodsafetyfscj.D-25-00002)
Supplement: Supplementary file 1 [file foodsafetyfscj-14-2-48-s001.pdf]

U.S. FDA Regulatory Monitoring of Ochratoxin A in Human Foods: 2008-2022

Tabitha J. Miller*, Anthony Adeuya, Lauren P. Robin

Office of Food Chemical Safety, Dietary Supplements, and Innovation; Human Foods Program; US Food and Drug Administration, 5001 Campus Drive, College Park Maryland 20740, USA

*Corresponding author: Tabitha J. Miller, US Food and Drug Administration, 5001 Campus Drive, College Park Maryland 20740, USA
tabitha.miller@fda.hhs.gov

Table S1. Global regulatory limits for ochratoxin A in foods.

Country or Organization	Foods Applicable to	Regulatory Limit (µg/kg)
Codex Alimentarius[1]	Wheat	5
	Barley	5
	Rye	5
	Chili pepper, paprika, nutmeg	20
European Union[2]	Unprocessed cereals	5
	All products, derived/processed from unprocessed cereals, with the exception of foodstuffs listed elsewhere	3
	Cereals placed on the market for the final consumer	3
	Bakery wares, cereal snacks, and breakfast cereals	
	products not containing oilseeds, nuts, or dried fruit	2
	products containing at least 20% dried vine fruit and/or dried figs	4
	other products containing oilseeds, nuts, and/or dried fruit	3
	Non-alcoholic malt beverages	3
	Wheat gluten not placed on the market for the final consumer	8
	Dried fruit	
	dried vine fruit (currants, raisins, and sultanas) and dried figs	8
	other dried fruit	2
	Date syrup	15
	Roasted coffee	

	roasted coffee beans and ground roasted coffee, excluding soluble coffee	3
	soluble coffee (instant coffee)	5
	Wine (including sparking wine, excluding liqueur wine and wine with an alcoholic strength of not less than 15% vol.) and fruit wine	2
	Aromatized wine, aromatized wine-based drinks, and aromatized wine-product cocktails	2
	Grape juice, concentrated grape juice as reconstituted, grape nectar, grape must, and concentrated grape must as reconstituted, placed on the market for the final consumer	2
	Processed cereal-based foods for infants and young children and baby foods	0.50
	Dietary foods for special medical purposes intended for infants and young children	0.50
	Spices, including dried spices, except *Capsicum spp.*	15
	Capsicum spp. (dried fruits thereof, whole or ground, including chillies, chilli powder, cayenne, or paprika)	20
	Mixtures of spices	15
	Liquorice (*Glycyrrhiza glabra, Glycyrrhiza inflate* and other species)	
	liquorice root, including as an ingredient in herbal infusions	20
	liquorice extract for use in food in particular beverages and confectionary	80
	liquorice confectionary containing ≥97% liquorice extract on dry basis	50
	Other liquorice confectionary	10
	Dried herbs	10
	Ginger roots for use in herbal infusions	15
	Marshmallow roots, dandelion roots, and orange blossoms for use in herbal infusions or in coffee substitutes	20
	Sunflower seeds, pumpkin seeds, (water) melon seeds, hempseeds, soybeans	5
	Pistachios to be subjected to sorting, or other physical treatment, before placing on the market for final consumer or use as ingredient in food	10
	Pistachios placed on the market for final consumer or use as ingredient in foodstuffs	5
	Cocoa powder	3
***Brazil*[a,3]**	Cereals and cereal products, including malted barley	10
	Beans	10
	Roasted coffee (ground or beans) and instant coffee	10
	Wine and its derivatives	2

	Grape juice and grape pulp	2
	Spices: *Capsicum spp.* (the fruit dried, whole or crushed, including peppers, chili powder, chili cayenne and sweet pepper), *Piper spp.* (the fruit, including white pepper and black pepper), *Myristica fragrans* (nutmeg), *Zingiber officinale* (ginger), *Curcuma longa* (turmeric), spice mixes that contain one or more of the spices listed above	30
	Cereal-based foods for infant nutrition (infants and early childhood children)	2
	Cocoa and chocolate products	5
	Cocoa beans	10
	Dried and dehydrated fruits	10
Switzerland[b,4]	Non-alcoholic malt beverages	3
	Instant coffee	5
	Roasted coffee (ground or whole)	3
	Cereals, raw	5
	Hempseed	5
	Licorice-based confectionary, others	10
	Licorice-based confectionary containing ≥97% licorice extract on a dry matter basis	50
	Foodstuffs intended for special medical purposes for infants and children	0.5
	Spices and mixed spices, except paprika and chili powder derived from *Capsicum* spp., cayenne pepper and paprika; including dried spices	15
	Licorice extract	80
	Soybeans	5
	Dried figs	8
	Dried fruits	2
	Wheat gluten not on the market for final consumer	8
	Pumpkin seeds	5
	Watermelon and melon seeds	5
	Sunflower seeds	5
	Dried herbs	10
	Grape juice, reconstituted concentrated grape juice, and grape nectar	2
	Paprika and chili powder	20

	Pistachios, intended to be subjected to sorting or other physical treatment prior to direct human consumption or use as food ingredients	10
	Pistachios, intended for direct human consumption or for use as food ingredients	5
	Cocoa powder	3
	Cereal-based preparations and foods for babies intended for infants and children in young age	0.5
	Baked goods, cereal snacks and breakfast cereals not containing oilseeds, nuts or dried fruits	2
	Baked goods, cereal snacks and breakfast cereals containing at least 20% raisins or dried figs	4
	Baked goods, cereal snacks and breakfast cereals containing oilseeds, nuts or dried fruits	3
	Products derived from raw cereals	3
	Ginger root	15
	Marshmallow roots, dandelion roots, and orange blossoms	20
	Licorice root	20
	Raisins	8
	Date syrup	15
	Wine	2
United Kingdom [c,5]	Unprocessed cereals	5
	All products derived from unprocessed cereals, including processed cereal products and cereals intended for direct human consumption with the exception of foodstuffs listed elsewhere	3
	Dried vine fruit (currants, raisins, and sultanas)	10
	Roasted coffee beans and ground roasted coffee, excluding soluble coffee	5
	Soluble coffee (instant coffee)	10
	Wine (including sparkling wine, excluding liqueur wine and wine with an alcoholic strength of not less than 15% vol) and fruit wine	2
	Aromatized wine, aromatized wine-based drinks, and aromatized wine-product cocktails	2
	Grape juice, concentrated grape juice as reconstituted, grape nectar, grape must and concentrated grape must as reconstituted, intended for direct human consumption	2

	Processed cereal-based foods and baby foods for infants and young children	0.5
	Dietary foods for special medical purposes intended specifically for infants	0.5
	Spices, including dried spices	
	Piper spp. (fruits thereof, including white and black pepper), *Myristica fragrans* (nutmeg), *Zingiber officinale* (ginger), *Curcuma longa* (turmeric)	15
	Capsicum spp. (dried fruits thereof, whole or ground, including chilies, chili powder, cayenne, and paprika)	20
	Mixtures of spices containing one of the abovementioned spices	15
	Licorice (*Glycyrrhiza glabra*, *Glycyrrhiza inflate* and other species)	
	Licorice root, ingredient for herbal infusion	20
	Licorice extract, for use in food in particular beverages and confectionary	80
	Wheat gluten not sold directly to the consumer	8
Morocco[d,6]	Raw cereals	5
	All products derived from raw cereals, including processed cereal products and cereals intended for human consumption, with the exception of food products listed elsewhere	3
	Raisins (currants, sultanas, and other raisins)	10
	Roasted coffee beans and ground roasted coffee, except instant soluble coffee	5
	Soluble coffee (instant coffee)	10
	Wines (including sparkling wines, but excluding liqueur wines and wines with a minimum alcoholic strength by volume of 15%) and fruit wines	2
	Aromatized wines, aromatized wine-based drinks, and cocktails flavored with wine products	2
	Grape juice, reconstituted concentrated grape juice, grape nectar, grape must and reconstituted concentrated grape must, intended for direct human consumption	2
	Cereal-based preparations and baby foods intended for infants and young children	0.5
	Dietary foods for special medical purposes, specifically for infants	0.5
	Spices, including dried:<br>*Piper spp.* (fruits derived from it, including white pepper and black pepper)<br>*Myristica fragrans* (nutmeg)<br>*Zingiber officinale* (ginger)<br>*Curcuma longa* (Indian saffron)	15
	Capsicum spp. (dried fruits, whole or powder, including chili peppers, chili powder, cayenne pepper, and paprika)	20

	Spice blends containing any of the above spices	15
	Licorice root (*Glycyrrhiza glabra, Glycyrrhiza inflate* and other species), ingredient for infusion	20
	Licorice extract (*Glycyrrhiza glabra, Glycyrrhiza inflate* and other species), for use in food products, in particular beverages and confectionary	80
	Wheat gluten not sold directly to the consumer	8
Singapore[7]	Unprocessed cereal grain	5
	Dried vine fruit (currants, raisins and sultanas)	10
	Roasted coffee beans and ground roasted coffee, excluding soluble coffee	5
	Soluble coffee (instant coffee)	10
	Food for infants and young children	0.5
	Spices, including dried spices	20
	Wine and fruit wine	2
	All food derived from cereals except food for infants and young children	3

[a]Translated from the original Portuguese. [b]Translated from the original French. [c]The United Kingdom retained the EU legislation regarding contaminants in foods upon leaving the EU. [d]Translated from the original French.

References

(1) Codex Alimentarius Commission (CAC). *General Standard for Contaminants and Toxins in Food and Feed*. CXS 193-1995. CAC; 2023. Accessed January 15, 2025. https://www.fao.org/fao-who-codexalimentarius/sh-proxy/en/?lnk=1&url=https%253A%252F%252Fworkspace.fao.org%252Fsites%252Fcodex%252FStandards%252FCXS%2B193-1995%252FCXS_193e.pdf

(2) European Commission (EC). *Commission Regulation (EU) 2023/915 of 25 April 2023 on maximum levels for certain contaminants in food and repealing Regulation (EC) No 1881/2006.* Doc no 32023R0915. EC; 2023. Accessed January 15, 2023. https://eur-lex.europa.eu/legal-content/EN/TXT/?uri=CELEX:32023R0915

(3) Brazilian Health Regulatory Agency (ANVISA). *Resolução da Diretoria Colegiada n. 7 - Regulamento Técnico sobre limites máximos tolerados (LMT) para micotoxinas em alimentos [Board Resolution RDC No. 7 on the Technical Regulation on Maximum Acceptable Limits for mycotoxins in food].* ANVISA; 2017. Accessed January 16, 2025. https://www.gov.br/agricultura/pt-br/assuntos/inspecao/produtos-animal/plano-de-nacional-de-controle-de-residuos-e-contaminantes/rdc-anvisa-2011_07.pdf

(4) Swiss Federal Department of the Interior (DFI). *Ordonnance du DFI sur les teneurs maximales en contaminants [DFI Ordinance on maximum levels of contaminants].* DFI; 2024. Accessed January 16, 2025. https://www.fedlex.admin.ch/eli/cc/2017/156/fr

(5) EC. *Commission Regulation (EC) No 1881/2006 of 19 December 2006 setting maximum levels for certain contaminants in foodstuffs (Text with EEA relevance).* EC; 2006. Accessed January 16, 2025. https://www.legislation.gov.uk/eur/2006/1881/introduction

(6) National Office for Sanitary Safety of Food Products (ONSSA); Ministry of Health of Morocco. *Joint Decree of the Minister of Agriculture and Marine Fisheries and the Minister of Health No. 1643-16 of 23 Chaabane 1437 (30 May 2016) setting the maximum permissible levels of contaminants in primary products and foodstuffs*. ONSSA; Ministry of Health of Morocco; 2016. Accessed January 16, 2025. https://www.onssa.gov.ma/wp-content/uploads/2024/02/ARR.1643-16.ENG_.c2.pdf

(7) Singapore Food Agency (SFA*). Maximum Residue Limits Established for Mercury, Bromate and Mycotoxins in Food*. SFA; 2020. Accessed January 16, 2025. https://www.sfa.gov.sg/docs/default-source/regulatory-standards-frameworks-and-guidelines/mycotoxins-and-marine-biotoxins.pdf
