# Supplementary material for: U.S. FDA Regulatory Monitoring of Ochratoxin A in Human Foods: 2008-2022
Source: Food Saf (Tokyo). 2026 Jun 26;14(2):48–57. doi: 10.14252/foodsafetyfscj.D-25-00002 (PMC13310607; doi:10.14252/foodsafetyfscj.D-25-00002)
Supplement: Supplementary file 2 [file foodsafetyfscj-14-2-48-s002.pdf]

**FY2008 Data**

| Sample Number | Fiscal Year | Collection Date | Product Name | Food Category | Food Subcategory | Origin Type | Country of Origin | Non-Detect, Trace, or Positive (>LOQ) | LOQ (ppb) | Amount found (ppb) |
|---|---|---|---|---|---|---|---|---|---|---|
| 441564 | 2008 | 10/3/2007 | PINTO BEAN | Beans and Legumes | Pinto Beans | IMPORT | Canada | Non-Detect | 2.3 | 0 |
| 442177 | 2008 | 10/10/2007 | WHEAT FLOUR, ENRICHED (ALL PURPOSE) | Grains and Grain Products | Wheat | IMPORT | Canada | Non-Detect | 0.233 | 0 |
| 442491 | 2008 | 10/15/2007 | SEMOLINA, WHEAT | Grains and Grain Products | Wheat | IMPORT | Canada | Trace | 1 | |
| 430260 | 2008 | 10/17/2007 | WHEAT FLOUR, ENRICHED (ALL PURPOSE) | Grains and Grain Products | Wheat | DOMESTIC | United States | Non-Detect | 0.233 | |
| 442946 | 2008 | 10/17/2007 | WHEAT FLOUR, ENRICHED (ALL PURPOSE) | Grains and Grain Products | Wheat | IMPORT | Mexico | Non-Detect | 4.99 | 0 |
| 442965 | 2008 | 10/17/2007 | SPAGHETTI | Processed Food Products | Pasta | IMPORT | Mexico | Non-Detect | 4.99 | 0 |
| 430265 | 2008 | 10/18/2007 | WHEAT FLOUR, ENRICHED (ALL PURPOSE) | Grains and Grain Products | Wheat | DOMESTIC | United States | Non-Detect | 0.233 | 0 |
| 443273 | 2008 | 10/18/2007 | WHEAT FLOUR, WHOLE | Grains and Grain Products | Wheat | IMPORT | Mexico | Non-Detect | 5 | 0 |
| 443294 | 2008 | 10/18/2007 | COFFEE, BEANS | Non-Juice Beverages | Coffee | IMPORT | Ecuador | Non-Detect | 2.3 | 0 |
| 443372 | 2008 | 10/18/2007 | WHEAT FLOUR, WHOLE | Grains and Grain Products | Wheat | IMPORT | India | Non-Detect | 5 | 0 |
| 443376 | 2008 | 10/18/2007 | WHEAT FLOUR, WHOLE | Grains and Grain Products | Wheat | IMPORT | India | Non-Detect | 5 | 0 |
| 443566 | 2008 | 10/19/2007 | PINTO BEAN, DRIED OR PASTE | Beans and Legumes | Pinto Beans | IMPORT | Mexico | Non-Detect | 5 | 0 |
| 444070 | 2008 | 10/24/2007 | OAT FLAKES, ROLLED OATS, PUFFS, KRISPIES, LOOPS READY TO EAT | Processed Food Products | Breakfast Foods | IMPORT | Canada | Non-Detect | 2.3 | 0 |
| 444344 | 2008 | 10/25/2007 | RAISINS (DRIED GRAPES) (BERRY) | Fruit | Raisins | IMPORT | Iran | Non-Detect | 0.233 | 0 |
| 444667 | 2008 | 10/29/2007 | WHEAT FLAKES, PUFFS, KRISPIES, LOOPS, SHREDDED, WHEAT GERM READY TO EAT | Processed Food Products | Breakfast Foods | IMPORT | United Kingdom | Non-Detect | 1 | 0 |
| 444826 | 2008 | 10/29/2007 | GARBANZO BEAN, DRIED OR PASTE | Beans and Legumes | Garbanzo Beans | IMPORT | Turkey | Non-Detect | 2.3 | 0 |
| 444843 | 2008 | 10/30/2007 | COFFEE, BEANS | Non-Juice Beverages | Coffee | IMPORT | Vietnam | Non-Detect | 2.3 | 0 |
| 444994 | 2008 | 10/31/2007 | GARBANZO BEAN | Beans and Legumes | Garbanzo Beans | IMPORT | Canada | Non-Detect | 2.3 | 0 |
| 444943 | 2008 | 10/31/2007 | OATMEAL, REGULAR, FRUIT OR SPICE ADDED, QUICK OR INSTANT COOKING | Processed Food Products | Breakfast Foods | DOMESTIC | United States | Positive | 1 | 1.2 |
| 442052 | 2008 | 11/5/2007 | CORN, ENRICHED MEAL | Grains and Grain Products | Corn | DOMESTIC | United States | Non-Detect | 2.3 | 0 |
| 442053 | 2008 | 11/5/2007 | CORN, ENRICHED MEAL | Grains and Grain Products | Corn | DOMESTIC | United States | Non-Detect | 2.3 | 0 |
| 445547 | 2008 | 11/5/2007 | WHEAT, WHOLE GRAIN | Grains and Grain Products | Wheat | IMPORT | Canada | Non-Detect | 1 | 0 |
| 445692 | 2008 | 11/6/2007 | WHEAT FLOUR, WHOLE | Grains and Grain Products | Wheat | DOMESTIC | United States | Non-Detect | 1 | 0 |
| 445693 | 2008 | 11/6/2007 | CORN, BOLTED MEAL OR FLOUR | Grains and Grain Products | Corn | DOMESTIC | United States | Non-Detect | 1 | 0 |
| 446594 | 2008 | 11/13/2007 | BEAN, DRIED OR PASTE, N.E.C. (VEGETABLE) | Beans and Legumes | Other Beans and Legumes | IMPORT | Mexico | Non-Detect | 0.233 | 0 |
| 446729 | 2008 | 11/14/2007 | MALT, BARLEY | Grains and Grain Products | Barley | IMPORT | Canada | Non-Detect | 1 | 0 |
| 444591 | 2008 | 11/19/2007 | CORN, BOLTED MEAL OR FLOUR | Grains and Grain Products | Corn | DOMESTIC | United States | Non-Detect | 1 | 0 |
| 444593 | 2008 | 11/19/2007 | CORN, BOLTED MEAL OR FLOUR | Grains and Grain Products | Corn | DOMESTIC | United States | Non-Detect | 1 | 0 |
| 444594 | 2008 | 11/19/2007 | CORN, BOLTED MEAL OR FLOUR | Grains and Grain Products | Corn | DOMESTIC | United States | Non-Detect | 1 | 0 |
| 444595 | 2008 | 11/19/2007 | CORN, BOLTED MEAL OR FLOUR | Grains and Grain Products | Corn | DOMESTIC | United States | Non-Detect | 1 | 0 |
| 447315 | 2008 | 11/19/2007 | BUCKWHEAT FLOUR | Grains and Grain Products | Buckwheat | IMPORT | Korea (the Republic of) | Non-Detect | 1 | 0 |
| 447318 | 2008 | 11/19/2007 | BARLEY, WHOLE GRAIN | Grains and Grain Products | Barley | IMPORT | Korea (the Republic of) | Non-Detect | 1 | 0 |
| 444592 | 2008 | 11/19/2007 | CORN, BOLTED MEAL OR FLOUR | Grains and Grain Products | Corn | DOMESTIC | United States | Trace | 1 | |
| 447945 | 2008 | 11/23/2007 | COFFEE, BEANS | Non-Juice Beverages | Coffee | IMPORT | Rwanda | Non-Detect | 1 | 0 |
| 300754 | 2008 | 11/28/2007 | WHEAT FLOUR, ENRICHED SELF RISING | Grains and Grain Products | Wheat | DOMESTIC | United States | Non-Detect | 2.3 | 0 |
| 448798 | 2008 | 11/29/2007 | COFFEE, BEANS | Non-Juice Beverages | Coffee | IMPORT | Peru | Non-Detect | 1 | 0 |
| 448704 | 2008 | 11/29/2007 | MALT, BARLEY | Grains and Grain Products | Barley | IMPORT | Canada | Trace | 1 | |
| 438238 | 2008 | 12/3/2007 | NAVY (WHITE) BEANS (BAKED BEANS, PORK AND BEANS), DRIED OR PASTE | Beans and Legumes | Other Beans and Legumes | DOMESTIC | United States | Non-Detect | 2.3 | 0 |
| 450446 | 2008 | 12/13/2007 | BLACK BEAN, DRIED OR PASTE | Beans and Legumes | Black Beans | IMPORT | China | Non-Detect | 1 | 0 |
| 450473 | 2008 | 12/13/2007 | WHEAT, WHOLE GRAIN | Grains and Grain Products | Wheat | IMPORT | Canada | Trace | 1 | |
| 443120 | 2008 | 12/18/2007 | WHEAT FLOUR, WHOLE | Grains and Grain Products | Wheat | DOMESTIC | United States | Non-Detect | 0.233 | 0 |
| 440545 | 2008 | 12/19/2007 | WHEAT FLOUR, WHOLE | Grains and Grain Products | Wheat | DOMESTIC | United States | Non-Detect | 2.3 | 0 |
| 451098 | 2008 | 12/19/2007 | COFFEE, BEANS | Non-Juice Beverages | Coffee | IMPORT | Mexico | Non-Detect | 0.233 | 0 |
| 451679 | 2008 | 12/27/2007 | BARLEY, WHOLE GRAIN | Grains and Grain Products | Barley | IMPORT | Canada | Non-Detect | 2.3 | 0 |
| 451990 | 2008 | 1/2/2008 | COFFEE, BEANS | Non-Juice Beverages | Coffee | IMPORT | Japan | Positive | 1 | 1.8 |
| 452069 | 2008 | 1/3/2008 | WHEAT, MILLED (CRUSHED, COARSE GROUND OR CRACKED) | Grains and Grain Products | Wheat | IMPORT | Canada | Non-Detect | 2.3 | 0 |
| 452152 | 2008 | 1/3/2008 | OAT FLAKES, ROLLED OATS, PUFFS, KRISPIES, LOOPS READY TO EAT | Processed Food Products | Breakfast Foods | IMPORT | Mexico | Non-Detect | 0.233 | 0 |
| 451853 | 2008 | 1/4/2008 | CORN, BOLTED MEAL OR FLOUR | Grains and Grain Products | Corn | DOMESTIC | United States | Non-Detect | 1 | 0 |
| 451854 | 2008 | 1/4/2008 | CORN, BOLTED MEAL OR FLOUR | Grains and Grain Products | Corn | DOMESTIC | United States | Non-Detect | 1 | 0 |
| 451855 | 2008 | 1/4/2008 | CORN, BOLTED MEAL OR FLOUR | Grains and Grain Products | Corn | DOMESTIC | United States | Non-Detect | 1 | 0 |
| 452219 | 2008 | 1/4/2008 | RAISINS, DRIED OR PASTE | Fruit | Raisins | IMPORT | Chile | Non-Detect | 2.3 | 0 |
| 451857 | 2008 | 1/4/2008 | CORN, BOLTED MEAL OR FLOUR | Grains and Grain Products | Corn | DOMESTIC | United States | Positive | 1 | 2.01 |
| 451856 | 2008 | 1/4/2008 | CORN, BOLTED MEAL OR FLOUR | Grains and Grain Products | Corn | DOMESTIC | United States | Trace | 1 | |
| 452402 | 2008 | 1/7/2008 | LENTILS | Beans and Legumes | Lentils | IMPORT | Mexico | Non-Detect | 0.233 | 0 |
| 452406 | 2008 | 1/7/2008 | GARBANZO BEAN | Beans and Legumes | Garbanzo Beans | IMPORT | Mexico | Non-Detect | 0.233 | 0 |
| 452409 | 2008 | 1/7/2008 | PINTO BEAN | Beans and Legumes | Pinto Beans | IMPORT | Mexico | Non-Detect | 0.233 | 0 |
| 452412 | 2008 | 1/7/2008 | MAYOCOBA BEANS | Beans and Legumes | Other Beans and Legumes | IMPORT | Mexico | Non-Detect | 0.233 | 0 |
| 393390 | 2008 | 1/15/2008 | BARLEY, WHOLE GRAIN | Grains and Grain Products | Barley | DOMESTIC | United States | Non-Detect | 2.3 | 0 |
| 393392 | 2008 | 1/15/2008 | BARLEY, WHOLE GRAIN | Grains and Grain Products | Barley | DOMESTIC | United States | Non-Detect | 2.3 | 0 |
| 393394 | 2008 | 1/15/2008 | BARLEY, WHOLE GRAIN | Grains and Grain Products | Barley | DOMESTIC | United States | Non-Detect | 2.3 | 0 |
| 393395 | 2008 | 1/15/2008 | BARLEY, WHOLE GRAIN | Grains and Grain Products | Barley | DOMESTIC | United States | Non-Detect | 2.3 | 0 |
| 453447 | 2008 | 1/16/2008 | MALT, BARLEY | Grains and Grain Products | Barley | IMPORT | Canada | Non-Detect | 2.3 | 0 |
| 453634 | 2008 | 1/16/2008 | FLOR DE MAYO BEAN | Beans and Legumes | Other Beans and Legumes | IMPORT | Mexico | Non-Detect | 4.99 | 0 |
| 453731 | 2008 | 1/16/2008 | RICE MEAL PROCESSED (PACKAGED) | Grains and Grain Products | Rice | IMPORT | Mexico | Non-Detect | 4.99 | 0 |
| 453432 | 2008 | 1/16/2008 | RAISINS (DRIED GRAPES) (BERRY) | Fruit | Raisins | IMPORT | South Africa | Trace | 7.57 | |
| 453617 | 2008 | 1/16/2008 | FAVA BEAN | Beans and Legumes | Other Beans and Legumes | IMPORT | Mexico | Trace | 5.01 | |
| 453640 | 2008 | 1/16/2008 | BLACK BEANS | Beans and Legumes | Black Beans | IMPORT | Mexico | Trace | 5 | |
| 300755 | 2008 | 1/17/2008 | WHEAT FLOUR, ENRICHED SELF RISING | Grains and Grain Products | Wheat | DOMESTIC | United States | Non-Detect | 2.3 | 0 |
| 454817 | 2008 | 1/28/2008 | PEAS | Beans and Legumes | Peas | IMPORT | China | Non-Detect | 2.3 | 0 |
| 449982 | 2008 | 1/30/2008 | CORN, DEGERMINATED MEAL | Grains and Grain Products | Corn | DOMESTIC | United States | Non-Detect | 2.3 | 0 |
| 449983 | 2008 | 1/30/2008 | CORN, DEGERMINATED MEAL | Grains and Grain Products | Corn | DOMESTIC | United States | Non-Detect | 2.3 | 0 |
| 455315 | 2008 | 1/30/2008 | COFFEE, BEANS | Non-Juice Beverages | Coffee | IMPORT | Ethiopia | Non-Detect | 2.3 | 0 |
| 455442 | 2008 | 1/31/2008 | GRAM FLOUR | Beans and Legumes | Garbanzo Beans | IMPORT | Canada | Non-Detect | 2.3 | 0 |
| 456084 | 2008 | 2/5/2008 | WHEAT FLOUR, GLUTEN | Grains and Grain Products | Wheat | IMPORT | United States | Trace | 1 | |
| 300756 | 2008 | 2/6/2008 | WHEAT FLOUR, GLUTEN | Grains and Grain Products | Wheat | DOMESTIC | United States | Non-Detect | 2.3 | 0 |
| 456546 | 2008 | 2/8/2008 | WHEAT FLOUR, GLUTEN | Grains and Grain Products | Wheat | DOMESTIC | United States | Trace | 5 | |
| 457271 | 2008 | 2/11/2008 | COFFEE, BEANS | Non-Juice Beverages | Coffee | IMPORT | Canada | Non-Detect | 2.3 | 0 |
| 457682 | 2008 | 2/18/2008 | RYE, WHOLE GRAIN | Grains and Grain Products | Rye | IMPORT | Canada | Non-Detect | 2.3 | 0 |
| 458071 | 2008 | 2/20/2008 | WHEAT, WHOLE GRAIN | Grains and Grain Products | Wheat | IMPORT | Lebanon | Non-Detect | 2.3 | 0 |
| 458383 | 2008 | 2/20/2008 | MALT, BARLEY | Grains and Grain Products | Barley | IMPORT | Canada | Non-Detect | 2.3 | 0 |
| 446861 | 2008 | 2/25/2008 | WHEAT FLOUR, BROMATED | Grains and Grain Products | Wheat | DOMESTIC | United States | Non-Detect | 0.233 | 0 |
| 446802 | 2008 | 3/3/2008 | WHEAT, WHOLE GRAIN | Grains and Grain Products | Wheat | DOMESTIC | United States | Non-Detect | 4.99 | 0 |

| | | | | | | | | | | |
|---|---|---|---|---|---|---|---|---|---|---|
| 446803 | 2008 | 3/3/2008 | OAT FLAKES, ROLLED OATS, PUFFS, KRISPIES, LOOPS READY TO EAT | Processed Food Products | Breakfast Foods | DOMESTIC | United States | Non-Detect | 4.99 | 0 |
| 459998 | 2008 | 3/4/2008 | RAISINS (DRIED GRAPES) (BERRY) | Fruit | Raisins | DOMESTIC | United States | Non-Detect | 2.3 | 0 |
| 460685 | 2008 | 3/7/2008 | OATMEAL, REGULAR, FRUIT OR SPICE ADDED, QUICK OR INSTANT COOKING | Processed Food Products | Breakfast Foods | IMPORT | Canada | Non-Detect | 2.3 | 0 |
| 375997 | 2008 | 3/11/2008 | CORN, PLAIN MEAL | Grains and Grain Products | Corn | DOMESTIC | United States | Non-Detect | 2.3 | 0 |
| 437253 | 2008 | 3/11/2008 | WHEAT FLOUR, ENRICHED (ALL PURPOSE) | Grains and Grain Products | Wheat | DOMESTIC | United States | Non-Detect | 2.3 | 0 |
| 461076 | 2008 | 3/12/2008 | RICE FLAKES, PUFFS, KRISPIES, LOOPS READY TO EAT | Processed Food Products | Breakfast Foods | DOMESTIC | United States | Non-Detect | 1 | 0 |
| 462525 | 2008 | 3/19/2008 | RAISINS, DRIED OR PASTE | Fruit | Raisins | IMPORT | South Africa | Non-Detect | 2.3 | 0 |
| 463409 | 2008 | 3/25/2008 | RAISINS (DRIED GRAPES) (BERRY) | Fruit | Raisins | IMPORT | China | Non-Detect | 2.3 | 0 |
| 305670 | 2008 | 3/31/2008 | WHEAT FLOUR, ENRICHED (ALL PURPOSE) | Grains and Grain Products | Wheat | DOMESTIC | United States | Non-Detect | 2.3 | 0 |
| 443318 | 2008 | 3/31/2008 | BEAN, DRIED OR PASTE, N.E.C. (VEGETABLE) | Beans and Legumes | Other Beans and Legumes | DOMESTIC | Unknown | Non-Detect | 2.3 | 0 |
| 443319 | 2008 | 3/31/2008 | PINTO BEAN, DRIED OR PASTE | Beans and Legumes | Pinto Beans | DOMESTIC | Unknown | Non-Detect | 2.3 | 0 |
| 454256 | 2008 | 3/31/2008 | COFFEE, BEANS | Non-Juice Beverages | Coffee | DOMESTIC | Unknown | Non-Detect | 2.3 | 0 |
| 461490 | 2008 | 4/1/2008 | CORN, DEGERMINATED MEAL | Grains and Grain Products | Corn | DOMESTIC | United States | Non-Detect | 4.99 | 0 |
| 464546 | 2008 | 4/3/2008 | COFFEE, BEANS | Non-Juice Beverages | Coffee | IMPORT | Singapore | Non-Detect | 1 | 0 |
| 464552 | 2008 | 4/3/2008 | COFFEE, BEANS | Non-Juice Beverages | Coffee | IMPORT | Tanzania, United Republic Of | Non-Detect | 1 | 0 |
| 464554 | 2008 | 4/3/2008 | COFFEE, BEANS | Non-Juice Beverages | Coffee | IMPORT | Burundi | Non-Detect | 1 | 0 |
| 465771 | 2008 | 4/10/2008 | WHEAT, MILLED (CRUSHED, COARSE GROUND OR CRACKED) | Grains and Grain Products | Wheat | IMPORT | Mexico | Non-Detect | 4.99 | 0 |
| 465980 | 2008 | 4/14/2008 | LENTILS | Beans and Legumes | Lentils | IMPORT | Turkey | Non-Detect | 2.3 | 0 |
| 466119 | 2008 | 4/14/2008 | COFFEE, BEANS | Non-Juice Beverages | Coffee | IMPORT | Tanzania, United Republic Of | Non-Detect | 1 | 0 |
| 466121 | 2008 | 4/14/2008 | COFFEE, BEANS | Non-Juice Beverages | Coffee | IMPORT | Kenya | Non-Detect | 1 | 0 |
| 452307 | 2008 | 4/17/2008 | RAISINS, DRIED OR PASTE | Fruit | Raisins | DOMESTIC | United States | Non-Detect | 1 | 0 |
| 460556 | 2008 | 4/17/2008 | WHEAT FLOUR, BROMATED | Grains and Grain Products | Wheat | DOMESTIC | United States | Non-Detect | 2.3 | 0 |
| 467217 | 2008 | 4/22/2008 | COFFEE, BEANS | Non-Juice Beverages | Coffee | IMPORT | Ethiopia | Positive | 2.6 | 5.2 |
| 467582 | 2008 | 4/24/2008 | CORN FLAKES, PUFFS, KRISPIES, LOOPS READY TO EAT | Processed Food Products | Other Corn Products | IMPORT | United States | Non-Detect | 4.99 | 0 |
| 466535 | 2008 | 4/29/2008 | CORN, BOLTED MEAL OR FLOUR | Grains and Grain Products | Corn | DOMESTIC | United States | Non-Detect | 1 | 0 |
| 466536 | 2008 | 4/29/2008 | CORN, BOLTED MEAL OR FLOUR | Grains and Grain Products | Corn | DOMESTIC | United States | Non-Detect | 1 | 0 |
| 466537 | 2008 | 4/29/2008 | CORN, BOLTED MEAL OR FLOUR | Grains and Grain Products | Corn | DOMESTIC | United States | Non-Detect | 1 | 0 |
| 468338 | 2008 | 5/1/2008 | OATMEAL, REGULAR, FRUIT OR SPICE ADDED, QUICK OR INSTANT COOKING | Processed Food Products | Breakfast Foods | IMPORT | Guatemala | Non-Detect | 5 | 0 |
| 469378 | 2008 | 5/8/2008 | RICE CAKES | Processed Food Products | Other Bakery Products | IMPORT | Canada | Non-Detect | 1 | 0 |
| 469996 | 2008 | 5/13/2008 | CEREAL GRAIN SOUP, DRIED | Processed Food Products | Breakfast Foods | IMPORT | Italy | Non-Detect | 0.233 | 0 |
| 470082 | 2008 | 5/13/2008 | WHEAT FLOUR, DURUM | Grains and Grain Products | Wheat | IMPORT | Canada | Non-Detect | 2.6 | 0 |
| 470084 | 2008 | 5/13/2008 | FARINA, WHEAT | Grains and Grain Products | Wheat | IMPORT | Canada | Positive | 2.8 | 4 |
| 470852 | 2008 | 5/19/2008 | MIXED CEREAL, QUICK OR INSTANT COOKING | Processed Food Products | Breakfast Foods | IMPORT | Guatemala | Non-Detect | 1 | 0 |
| 463702 | 2008 | 5/20/2008 | OAT CEREAL (BABY) | Baby Food Products | Baby Cereals | DOMESTIC | United States | Non-Detect | 1 | 0 |
| 470968 | 2008 | 5/20/2008 | BEAN, DRIED OR PASTE, N.E.C. (VEGETABLE) | Beans and Legumes | Other Beans and Legumes | IMPORT | Mexico | Non-Detect | 0.233 | 0 |
| 452639 | 2008 | 5/22/2008 | CORN, PLAIN MEAL | Grains and Grain Products | Corn | DOMESTIC | United States | Non-Detect | 1 | 0 |
| 472667 | 2008 | 6/3/2008 | WHEAT, WHOLE GRAIN | Grains and Grain Products | Wheat | IMPORT | Canada | Non-Detect | 2.3 | 0 |
| 472669 | 2008 | 6/3/2008 | WHEAT, WHOLE GRAIN | Grains and Grain Products | Wheat | IMPORT | Canada | Non-Detect | 2.3 | 0 |
| 472671 | 2008 | 6/3/2008 | WHEAT, WHOLE GRAIN | Grains and Grain Products | Wheat | DOMESTIC | United States | Non-Detect | 2.3 | 0 |
| 472680 | 2008 | 6/3/2008 | WHEAT FLOUR, WHOLE DURUM | Grains and Grain Products | Wheat | DOMESTIC | United States | Non-Detect | 2.7 | 0 |
| 472674 | 2008 | 6/3/2008 | WHEAT FLOUR, ENRICHED (ALL PURPOSE) | Grains and Grain Products | Wheat | DOMESTIC | United States | Positive | 2.7 | 3.8 |
| 472677 | 2008 | 6/3/2008 | WHEAT FLOUR, WHOLE DURUM | Grains and Grain Products | Wheat | DOMESTIC | United States | Trace | 2.7 | |
| 473215 | 2008 | 6/5/2008 | CORN CHIPS, FRIED | Processed Food Products | Other Corn Products | IMPORT | Philippines | Non-Detect | 1 | 0 |
| 473217 | 2008 | 6/5/2008 | CORN CHIPS, FRIED | Processed Food Products | Other Corn Products | IMPORT | Philippines | Non-Detect | 1 | 0 |
| 300759 | 2008 | 6/6/2008 | SEMOLINA, WHEAT | Grains and Grain Products | Wheat | IMPORT | Mexico | Non-Detect | 2.3 | 0 |
| 473602 | 2008 | 6/10/2008 | KIDNEY BEAN, DRIED OR PASTE | Beans and Legumes | Other Beans and Legumes | IMPORT | China | Non-Detect | 1 | 0 |
| 300758 | 2008 | 6/18/2008 | WHEAT FLOUR, GLUTEN | Grains and Grain Products | Wheat | IMPORT | United States | Non-Detect | 2.3 | 0 |
| 472683 | 2008 | 6/24/2008 | WHEAT FLOUR, WHOLE | Grains and Grain Products | Wheat | DOMESTIC | United States | Non-Detect | 2.7 | 0 |
| 472686 | 2008 | 6/24/2008 | WHEAT FLOUR, WHOLE | Grains and Grain Products | Wheat | DOMESTIC | United States | Non-Detect | 2.7 | 0 |
| 472688 | 2008 | 6/24/2008 | RYE FLOUR | Grains and Grain Products | Rye | DOMESTIC | United States | Non-Detect | 2.7 | 0 |
| 475253 | 2008 | 6/25/2008 | COFFEE, BEANS | Non-Juice Beverages | Coffee | IMPORT | Indonesia | Non-Detect | 1 | 0 |
| 300760 | 2008 | 6/27/2008 | FLOURS AND MEALS N.E.C. | Grains and Grain Products | Other Grains | IMPORT | United States | Non-Detect | 2.3 | 0 |
| 475833 | 2008 | 7/1/2008 | COFFEE, BEANS | Non-Juice Beverages | Coffee | IMPORT | Italy | Trace | 2.3 | |
| 475837 | 2008 | 7/1/2008 | COFFEE, BEANS | Non-Juice Beverages | Coffee | IMPORT | Italy | Trace | 2.3 | |
| 475838 | 2008 | 7/1/2008 | COFFEE, BEANS | Non-Juice Beverages | Coffee | IMPORT | Italy | Trace | 2.3 | |
| 477470 | 2008 | 7/9/2008 | GARBANZO BEAN | Beans and Legumes | Garbanzo Beans | IMPORT | Canada | Non-Detect | 0.233 | 0 |
| 477630 | 2008 | 7/9/2008 | FAVA BEAN, DRIED OR PASTE | Beans and Legumes | Other Beans and Legumes | IMPORT | Canada | Non-Detect | 0.233 | 0 |
| 477817 | 2008 | 7/15/2008 | WHEAT FLOUR, WHOLE | Grains and Grain Products | Wheat | IMPORT | United Arab Emirates | Non-Detect | 1 | 0 |
| 478164 | 2008 | 7/18/2008 | GARBANZO BEAN, DRIED OR PASTE | Beans and Legumes | Garbanzo Beans | IMPORT | Turkey | Non-Detect | 1 | 0 |
| 478310 | 2008 | 7/21/2008 | GARBANZO BEAN | Beans and Legumes | Garbanzo Beans | IMPORT | Iran | Non-Detect | 1 | 0 |
| 478664 | 2008 | 7/23/2008 | WHEAT FLAKES, PUFFS, KRISPIES, LOOPS, SHREDDED, WHEAT GERM READY TO EAT | Processed Food Products | Breakfast Foods | IMPORT | South Africa | Non-Detect | 1 | 0 |
| 478673 | 2008 | 7/23/2008 | WHEAT FLAKES, PUFFS, KRISPIES, LOOPS, SHREDDED, WHEAT GERM READY TO EAT | Processed Food Products | Breakfast Foods | IMPORT | South Africa | Non-Detect | 1 | 0 |
| 478675 | 2008 | 7/23/2008 | WHEAT FLAKES, PUFFS, KRISPIES, LOOPS, SHREDDED, WHEAT GERM READY TO EAT | Processed Food Products | Breakfast Foods | IMPORT | South Africa | Non-Detect | 1 | 0 |
| 478737 | 2008 | 7/24/2008 | MALT FLOUR | Grains and Grain Products | Other Grains | IMPORT | Korea (the Republic of) | Non-Detect | 1 | 0 |
| 443078 | 2008 | 7/25/2008 | OAT FLAKES, ROLLED OATS, PUFFS, KRISPIES, LOOPS READY TO EAT | Processed Food Products | Breakfast Foods | DOMESTIC | United States | Non-Detect | 2.3 | 0 |
| 479378 | 2008 | 7/30/2008 | BLACK BEAN, DRIED OR PASTE | Beans and Legumes | Black Beans | IMPORT | Thailand | Non-Detect | 0.233 | 0 |
| 479548 | 2008 | 7/31/2008 | COFFEE, BEANS | Non-Juice Beverages | Coffee | IMPORT | Vietnam | Non-Detect | 1 | 0 |
| 480244 | 2008 | 8/6/2008 | WHEAT FLOUR, WHOLE | Grains and Grain Products | Wheat | IMPORT | United Arab Emirates | Non-Detect | 2.3 | 0 |
| 480676 | 2008 | 8/8/2008 | MUNG BEAN, DRIED OR PASTE | Beans and Legumes | Other Beans and Legumes | IMPORT | Thailand | Non-Detect | 2.3 | 0 |
| 480748 | 2008 | 8/8/2008 | MUNG BEAN, DRIED OR PASTE | Beans and Legumes | Other Beans and Legumes | IMPORT | India | Non-Detect | 2.3 | |
| 480844 | 2008 | 8/8/2008 | MUNG BEAN, DRIED OR PASTE | Beans and Legumes | Other Beans and Legumes | IMPORT | India | Non-Detect | 2.3 | 0 |
| 478611 | 2008 | 8/13/2008 | WHEAT, WHOLE GRAIN | Grains and Grain Products | Wheat | DOMESTIC | United States | Non-Detect | 2.3 | 0 |
| 481504 | 2008 | 8/15/2008 | COFFEE, BEANS | Non-Juice Beverages | Coffee | IMPORT | Ethiopia | Non-Detect | 1 | 0 |
| 462977 | 2008 | 8/21/2008 | BLACK BEANS | Beans and Legumes | Black Beans | IMPORT | Canada | Non-Detect | 2.3 | 0 |
| 482717 | 2008 | 8/21/2008 | NAVY (WHITE) BEANS, (BAKED BEANS, PORK AND BEANS) | Beans and Legumes | Other Beans and Legumes | DOMESTIC | Unknown | Non-Detect | 2.3 | 0 |
| 482858 | 2008 | 8/21/2008 | PINTO BEAN | Beans and Legumes | Pinto Beans | DOMESTIC | United States | Non-Detect | 2.3 | 0 |
| 462645 | 2008 | 8/26/2008 | WHEAT, WHOLE GRAIN | Grains and Grain Products | Wheat | DOMESTIC | United States | Non-Detect | 2.3 | 0 |
| 462647 | 2008 | 8/26/2008 | WHEAT, WHOLE GRAIN | Grains and Grain Products | Wheat | DOMESTIC | United States | Non-Detect | 2.3 | 0 |
| 462649 | 2008 | 8/26/2008 | WHEAT, WHOLE GRAIN | Grains and Grain Products | Wheat | DOMESTIC | United States | Non-Detect | 2.3 | 0 |
| 483772 | 2008 | 8/27/2008 | BARLEY, WHOLE GRAIN | Grains and Grain Products | Barley | IMPORT | Korea (the Republic of) | Trace | 1 | |
| 478625 | 2008 | 9/4/2008 | WHEAT, WHOLE GRAIN | Grains and Grain Products | Wheat | DOMESTIC | United States | Non-Detect | 2.3 | 0 |
| 478627 | 2008 | 9/4/2008 | WHEAT, WHOLE GRAIN | Grains and Grain Products | Wheat | DOMESTIC | United States | Non-Detect | 2.3 | 0 |
| 478631 | 2008 | 9/4/2008 | WHEAT, WHOLE GRAIN | Grains and Grain Products | Wheat | DOMESTIC | United States | Non-Detect | 2.3 | 0 |

| | | | | | | | | | | |
|---|---|---|---|---|---|---|---|---|---|---|
| 483043 | 2008 | 9/4/2008 | WHEAT, WHOLE GRAIN | Grains and Grain Products | Wheat | DOMESTIC | United States | Non-Detect | 2.3 | 0 |
| 483045 | 2008 | 9/4/2008 | WHEAT, WHOLE GRAIN | Grains and Grain Products | Wheat | DOMESTIC | United States | Non-Detect | 2.3 | 0 |
| 483047 | 2008 | 9/4/2008 | WHEAT, WHOLE GRAIN | Grains and Grain Products | Wheat | DOMESTIC | United States | Non-Detect | 2.3 | 0 |
| 483049 | 2008 | 9/4/2008 | WHEAT, WHOLE GRAIN | Grains and Grain Products | Wheat | DOMESTIC | United States | Non-Detect | 2.3 | 0 |
| 482744 | 2008 | 9/8/2008 | CORN, PLAIN MEAL | Grains and Grain Products | Corn | DOMESTIC | United States | Non-Detect | 2.3 | 0 |
| 482745 | 2008 | 9/8/2008 | WHEAT FLOUR, ENRICHED BROMATED | Grains and Grain Products | Wheat | DOMESTIC | United States | Non-Detect | 2.3 | 0 |
| 457583 | 2008 | 9/9/2008 | RAISINS, DRIED OR PASTE | Fruit | Raisins | DOMESTIC | United States | Non-Detect | 0.233 | 0 |
| 483063 | 2008 | 9/10/2008 | WHEAT FLOUR, ENRICHED (ALL PURPOSE) | Grains and Grain Products | Wheat | DOMESTIC | United States | Non-Detect | 2.3 | 0 |
| 483067 | 2008 | 9/10/2008 | FARINA, WHEAT | Grains and Grain Products | Wheat | DOMESTIC | United States | Non-Detect | 2.3 | 0 |
| 487032 | 2008 | 9/12/2008 | BARLEY, WHOLE GRAIN | Grains and Grain Products | Barley | IMPORT | Korea (the Republic of) | Non-Detect | 1 | 0 |
| 482747 | 2008 | 9/15/2008 | WHEAT FLOUR, ENRICHED (ALL PURPOSE) | Grains and Grain Products | Wheat | DOMESTIC | United States | Non-Detect | 2.3 | 0 |
| 482748 | 2008 | 9/15/2008 | WHEAT FLOUR, ENRICHED (ALL PURPOSE) | Grains and Grain Products | Wheat | DOMESTIC | United States | Non-Detect | 2.3 | 0 |
| 480832 | 2008 | 9/17/2008 | CORN, HOMINY GRITS | Grains and Grain Products | Corn | DOMESTIC | Unknown | Non-Detect | 1 | 0 |
| 486981 | 2008 | 9/17/2008 | CORN, DEGERMINATED MEAL | Grains and Grain Products | Corn | DOMESTIC | Unknown | Non-Detect | 1 | 0 |
| 482584 | 2008 | 9/29/2008 | RAISINS, DRIED OR PASTE | Fruit | Raisins | DOMESTIC | United States | Non-Detect | 2.3 | 0 |
| 485506 | 2008 | 9/29/2008 | WHEAT FLOUR, ENRICHED (ALL PURPOSE) | Grains and Grain Products | Wheat | DOMESTIC | United States | Non-Detect | 2.3 | 0 |

**FY2009 Data**

| Sample Number | Fiscal Year | Collection Date | Product Name | Food Category | Food Subcategory | Origin Type | Country of Origin | Non-Detect, Trace, or Positive (>LOQ) | LOQ (ppb) | Amount found (ppb) |
|---|---|---|---|---|---|---|---|---|---|---|
| 491654 | 2009 | 10/1/2008 | COFFEE, BEANS | Non-Juice Beverages | Coffee | IMPORT | Colombia | Non-Detect | 2.3 | 0 |
| 493714 | 2009 | 10/8/2008 | GARBANZO BEAN | Beans and Legumes | Garbanzo Beans | IMPORT | Canada | Non-Detect | 2.3 | 0 |
| 305671 | 2009 | 10/9/2008 | WHEAT FLOUR, BROMATED | Grains and Grain Products | Wheat | DOMESTIC | United States | Non-Detect | 2.3 | 0 |
| 494100 | 2009 | 10/14/2008 | WHEAT FLOUR, DURUM | Grains and Grain Products | Wheat | IMPORT | Canada | Non-Detect | 2.3 | 0 |
| 496311 | 2009 | 10/22/2008 | SOBA NOODLE (JAPANESE NOODLES MADE FROM BUCKWHEAT WHEAT FLOUR AND WATER) | Processed Food Products | Pasta | IMPORT | Korea (the Republic of) | Non-Detect | 1 | 0 |
| 497597 | 2009 | 10/29/2008 | WHEAT, WHOLE GRAIN | Grains and Grain Products | Wheat | IMPORT | Canada | Non-Detect | 2.3 | 0 |
| 498485 | 2009 | 10/30/2008 | CHANA DAL, DRIED OR PASTE | Beans and Legumes | Garbanzo Beans | IMPORT | Thailand | Non-Detect | 1 | 0 |
| 498487 | 2009 | 10/31/2008 | CHANA DAL, DRIED OR PASTE | Beans and Legumes | Garbanzo Beans | IMPORT | Thailand | Non-Detect | 2.3 | 0 |
| 498491 | 2009 | 10/31/2008 | PIGEON PEAS, DRIED OR PASTE | Beans and Legumes | Peas | IMPORT | Thailand | Non-Detect | 2.3 | 0 |
| 498523 | 2009 | 11/3/2008 | BLACK BEAN, DRIED OR PASTE | Beans and Legumes | Black Beans | IMPORT | Thailand | Non-Detect | 2.3 | 0 |
| 481829 | 2009 | 11/18/2008 | RAISINS, DRIED OR PASTE | Fruit | Raisins | DOMESTIC | United States | Non-Detect | 1 | 0 |
| 481830 | 2009 | 11/18/2008 | RAISINS, DRIED OR PASTE | Fruit | Raisins | DOMESTIC | United States | Non-Detect | 1 | 0 |
| 503962 | 2009 | 12/1/2008 | CORN, BOLTED MEAL OR FLOUR | Grains and Grain Products | Corn | DOMESTIC | United States | Non-Detect | 1 | 0 |
| 503963 | 2009 | 12/1/2008 | CORN, BOLTED MEAL OR FLOUR | Grains and Grain Products | Corn | DOMESTIC | United States | Non-Detect | 1 | 0 |
| 503964 | 2009 | 12/1/2008 | CORN, BOLTED MEAL OR FLOUR | Grains and Grain Products | Corn | DOMESTIC | United States | Non-Detect | 1 | 0 |
| 503965 | 2009 | 12/1/2008 | CORN, DEGERMINATED MEAL | Grains and Grain Products | Corn | DOMESTIC | United States | Non-Detect | 1 | 0 |
| 503966 | 2009 | 12/1/2008 | CORN, BOLTED MEAL OR FLOUR | Grains and Grain Products | Corn | DOMESTIC | United States | Non-Detect | 1 | 0 |
| 495586 | 2009 | 12/2/2008 | WHEAT FLOUR, DURUM | Grains and Grain Products | Wheat | DOMESTIC | United States | Non-Detect | 2.3 | 0 |
| 495588 | 2009 | 12/2/2008 | WHEAT FLOUR, ENRICHED (ALL PURPOSE) | Grains and Grain Products | Wheat | DOMESTIC | United States | Non-Detect | 2.3 | 0 |
| 501576 | 2009 | 12/4/2008 | WHEAT FLOUR, ENRICHED (ALL PURPOSE) | Grains and Grain Products | Wheat | DOMESTIC | United States | Non-Detect | 2.3 | 0 |
| 504237 | 2009 | 12/5/2008 | MALT, BARLEY | Grains and Grain Products | Barley | DOMESTIC | United States | Non-Detect | 0.233 | 0 |
| 506031 | 2009 | 12/5/2008 | MIXED CEREAL, QUICK OR INSTANT COOKING | Processed Food Products | Breakfast Foods | IMPORT | Guatemala | Non-Detect | 0.233 | 0 |
| 505813 | 2009 | 12/5/2008 | WHEAT, WHOLE GRAIN | Grains and Grain Products | Wheat | IMPORT | Canada | Non-Detect | 1 | 0 |
| 506131 | 2009 | 12/8/2008 | OATMEAL, REGULAR, FRUIT OR SPICE ADDED, QUICK OR INSTANT COOKING | Processed Food Products | Breakfast Foods | IMPORT | Mexico | Non-Detect | 0.233 | 0 |
| 506262 | 2009 | 12/8/2008 | WHEAT, WHOLE GRAIN | Grains and Grain Products | Wheat | IMPORT | Canada | Non-Detect | 1 | 0 |
| 502244 | 2009 | 12/15/2008 | WHEAT FLOUR, BROMATED | Grains and Grain Products | Wheat | DOMESTIC | United States | Non-Detect | 2.3 | 0 |
| 510528 | 2009 | 1/6/2009 | COFFEE, BEANS, DECAFFEINATED | Non-Juice Beverages | Coffee | IMPORT | Germany | Non-Detect | 1.7 | 0 |
| 512184 | 2009 | 1/12/2009 | MALT, BARLEY | Grains and Grain Products | Barley | IMPORT | Korea (the Republic of) | Non-Detect | 1.7 | 0 |
| 511727 | 2009 | 1/12/2009 | GARBANZO BEAN | Beans and Legumes | Garbanzo Beans | IMPORT | Mexico | Non-Detect | 0.233 | 0 |
| 511740 | 2009 | 1/12/2009 | LIMA BEAN, DRIED OR PASTE | Beans and Legumes | Other Beans and Legumes | IMPORT | Mexico | Non-Detect | 0.233 | 0 |
| 512021 | 2009 | 1/13/2009 | LENTILS, DRIED OR PASTE | Beans and Legumes | Lentils | IMPORT | Mexico | Non-Detect | 1.7 | 0 |
| 505336 | 2009 | 1/14/2009 | RYE FLOUR | Grains and Grain Products | Rye | DOMESTIC | United States | Non-Detect | 1.7 | 0 |
| 505337 | 2009 | 1/14/2009 | WHEAT FLOUR, WHOLE | Grains and Grain Products | Wheat | DOMESTIC | United States | Non-Detect | 1.7 | 0 |
| 513021 | 2009 | 1/15/2009 | CORN, DRIED OR PASTE | Grains and Grain Products | Corn | IMPORT | Peru | Non-Detect | 1 | 0 |
| 513022 | 2009 | 1/15/2009 | WHEAT, WHOLE GRAIN | Grains and Grain Products | Wheat | IMPORT | Peru | Non-Detect | 1 | 0 |
| 513372 | 2009 | 1/15/2009 | PINTO BEAN | Beans and Legumes | Pinto Beans | IMPORT | China | Non-Detect | 1 | 0 |
| 511320 | 2009 | 1/16/2009 | RAISINS, DRIED OR PASTE | Fruit | Raisins | DOMESTIC | United States | Non-Detect | 1 | 0 |
| 511321 | 2009 | 1/16/2009 | RAISINS, DRIED OR PASTE | Fruit | Raisins | DOMESTIC | United States | Non-Detect | 1 | 0 |
| 514021 | 2009 | 1/22/2009 | PINTO BEAN, DRIED OR PASTE | Beans and Legumes | Pinto Beans | IMPORT | Mexico | Non-Detect | 1.7 | 0 |
| 499776 | 2009 | 1/23/2009 | WHEAT, WHOLE GRAIN | Grains and Grain Products | Wheat | DOMESTIC | United States | Non-Detect | 1.7 | 0 |
| 514226 | 2009 | 1/23/2009 | COFFEE, BEANS | Non-Juice Beverages | Coffee | IMPORT | Yemen | Non-Detect | 1 | 0 |
| 519511 | 2009 | 2/10/2009 | WHEAT FLOUR, WHOLE | Grains and Grain Products | Wheat | IMPORT | Japan | Non-Detect | 1.7 | 0 |
| 519977 | 2009 | 2/18/2009 | OAT FLOUR | Grains and Grain Products | Oats | IMPORT | United States | Trace | 10.08 | |
| 521000 | 2009 | 2/23/2009 | OATS, WHOLE GRAIN | Grains and Grain Products | Oats | IMPORT | Canada | Non-Detect | 1.7 | 0 |
| 512779 | 2009 | 2/26/2009 | WHEAT, WHOLE GRAIN | Grains and Grain Products | Wheat | DOMESTIC | United States | Non-Detect | 1.7 | 0 |
| 522457 | 2009 | 2/26/2009 | WHEAT FLOUR, WHOLE DURUM | Grains and Grain Products | Wheat | IMPORT | Canada | Trace | 1 | |
| 522754 | 2009 | 2/27/2009 | WHEAT, MILLED (CRUSHED, COARSE GROUND OR CRACKED) | Grains and Grain Products | Wheat | IMPORT | Peru | Non-Detect | 1.7 | 0 |
| 522917 | 2009 | 2/27/2009 | WHEAT, WHOLE GRAIN | Grains and Grain Products | Wheat | IMPORT | Canada | Non-Detect | 1 | 0 |
| 503576 | 2009 | 3/4/2009 | WHEAT, WHOLE GRAIN | Grains and Grain Products | Wheat | DOMESTIC | United States | Non-Detect | 1.7 | 0 |
| 503578 | 2009 | 3/4/2009 | WHEAT, WHOLE GRAIN | Grains and Grain Products | Wheat | DOMESTIC | United States | Non-Detect | 1.7 | 0 |
| 524334 | 2009 | 3/4/2009 | RICE CAKES | Processed Food Products | Other Bakery Products | IMPORT | China | Trace | 1 | |
| 524281 | 2009 | 3/5/2009 | PIZZA CRUST | Processed Food Products | Other Bakery Products | IMPORT | Mexico | Non-Detect | 1.7 | 0 |
| 524337 | 2009 | 3/5/2009 | RICE CAKES | Processed Food Products | Other Bakery Products | IMPORT | China | Trace | 1 | |
| 506701 | 2009 | 3/9/2009 | WHEAT FLAKES, PUFFS, KRISPIES, LOOPS, SHREDDED, WHEAT GERM READY TO EAT | Processed Food Products | Breakfast Foods | DOMESTIC | United States | Trace | 5.02 | |
| 527530 | 2009 | 3/16/2009 | COFFEE, BEANS | Non-Juice Beverages | Coffee | IMPORT | Honduras | Non-Detect | 1.7 | 0 |
| 527884 | 2009 | 3/17/2009 | COFFEE, BEANS | Non-Juice Beverages | Coffee | IMPORT | Indonesia | Non-Detect | 1.7 | 0 |
| 527899 | 2009 | 3/17/2009 | COFFEE, BEANS | Non-Juice Beverages | Coffee | IMPORT | Vietnam | Non-Detect | 1.7 | 0 |
| 494750 | 2009 | 3/19/2009 | BLACKEYE PEAS, DRIED OR PASTE | Beans and Legumes | Blackeye Peas | DOMESTIC | United States | Non-Detect | 0.233 | 0 |
| 529248 | 2009 | 3/23/2009 | PINTO BEAN | Beans and Legumes | Pinto Beans | IMPORT | Mexico | Non-Detect | 1.7 | 0 |
| 529464 | 2009 | 3/24/2009 | COFFEE, BEANS | Non-Juice Beverages | Coffee | IMPORT | Guatemala | Non-Detect | 1.7 | 0 |
| 485317 | 2009 | 4/2/2009 | BLACK BEAN, DRIED OR PASTE | Beans and Legumes | Black Beans | DOMESTIC | Unknown | Non-Detect | 0.233 | 0 |
| 531727 | 2009 | 4/2/2009 | WHEAT FLOUR, ENRICHED (ALL PURPOSE) | Grains and Grain Products | Wheat | IMPORT | Korea (the Republic of) | Non-Detect | 1 | 0 |
| 532111 | 2009 | 4/3/2009 | BARLEY, WHOLE GRAIN | Grains and Grain Products | Barley | IMPORT | Korea (the Republic of) | Non-Detect | 1 | 0 |
| 532116 | 2009 | 4/3/2009 | BARLEY, WHOLE GRAIN | Grains and Grain Products | Barley | IMPORT | Korea (the Republic of) | Non-Detect | 1 | 0 |
| 532175 | 2009 | 4/3/2009 | BARLEY, WHOLE GRAIN | Grains and Grain Products | Barley | IMPORT | Korea (the Republic of) | Non-Detect | 1 | 0 |
| 532177 | 2009 | 4/3/2009 | BARLEY, WHOLE GRAIN | Grains and Grain Products | Barley | IMPORT | Korea (the Republic of) | Non-Detect | 1 | 0 |
| 532196 | 2009 | 4/6/2009 | GRANOLA BARS | Processed Food Products | Other Processed Foods | IMPORT | Mexico | Non-Detect | 0.233 | 0 |
| 532478 | 2009 | 4/7/2009 | MALT, BARLEY | Grains and Grain Products | Barley | IMPORT | Canada | Non-Detect | 1 | 0 |
| 505199 | 2009 | 4/15/2009 | PEA, DRIED OR PASTE | Beans and Legumes | Peas | IMPORT | Spain | Non-Detect | 1.7 | 0 |
| 535170 | 2009 | 4/16/2009 | WHEAT FLOUR, WHOLE | Grains and Grain Products | Wheat | IMPORT | Canada | Non-Detect | 1.7 | 0 |
| 532542 | 2009 | 4/16/2009 | PINTO BEAN | Beans and Legumes | Pinto Beans | DOMESTIC | United States | Non-Detect | 0.233 | 0 |
| 535480 | 2009 | 4/17/2009 | COFFEE, BEANS | Non-Juice Beverages | Coffee | IMPORT | Brazil | Non-Detect | 1.7 | 0 |
| 537995 | 2009 | 4/27/2009 | CORN, PLAIN MEAL | Grains and Grain Products | Corn | DOMESTIC | United States | Non-Detect | 1.7 | 0 |
| 522199 | 2009 | 4/29/2009 | BREADING, BREADING MIXES. ETC. | Processed Food Products | Bread | DOMESTIC | United States | Non-Detect | 1.7 | 0 |
| 539473 | 2009 | 5/1/2009 | BARLEY, WHOLE GRAIN | Grains and Grain Products | Barley | IMPORT | Poland | Non-Detect | 1.7 | 0 |
| 539486 | 2009 | 5/1/2009 | BARLEY, WHOLE GRAIN | Grains and Grain Products | Barley | IMPORT | Poland | Non-Detect | 1.7 | 0 |
| 539514 | 2009 | 5/1/2009 | BUCKWHEAT, WHOLE GRAIN | Grains and Grain Products | Buckwheat | IMPORT | Poland | Non-Detect | 1.7 | 0 |
| 539575 | 2009 | 5/1/2009 | FARINA, WHEAT | Grains and Grain Products | Wheat | IMPORT | Poland | Non-Detect | 1.7 | 0 |
| 541473 | 2009 | 5/11/2009 | GRAM FLOUR | Beans and Legumes | Garbanzo Beans | IMPORT | Canada | Non-Detect | 1.7 | 0 |
| 541695 | 2009 | 5/12/2009 | COFFEE, BEANS | Non-Juice Beverages | Coffee | IMPORT | Papua New Guinea | Non-Detect | 1.7 | 0 |
| 541879 | 2009 | 5/12/2009 | CORN, BOLTED MEAL OR FLOUR | Grains and Grain Products | Corn | IMPORT | Argentina | Non-Detect | 1.7 | 0 |
| 525889 | 2009 | 5/14/2009 | BEAN SOUP, DRIED | Processed Food Products | Other Processed Foods | DOMESTIC | United States | Non-Detect | 1.7 | 0 |
| 543898 | 2009 | 5/15/2009 | GARBANZO BEAN | Beans and Legumes | Garbanzo Beans | IMPORT | Canada | Non-Detect | 1.7 | 0 |

| | | | | | | | | | | |
|---|---|---|---|---|---|---|---|---|---|---|
| 520840 | 2009 | 5/20/2009 | BARLEY, WHOLE GRAIN | Grains and Grain Products | Barley | DOMESTIC | United States | Non-Detect | 1.7 | 0 |
| 520842 | 2009 | 5/20/2009 | WHEAT, WHOLE GRAIN | Grains and Grain Products | Wheat | DOMESTIC | United States | Non-Detect | 3.1 | |
| 543869 | 2009 | 5/20/2009 | FAVA BEAN | Beans and Legumes | Other Beans and Legumes | IMPORT | Canada | Non-Detect | 1.7 | 0 |
| 521541 | 2009 | 5/27/2009 | WHEAT FLOUR, ENRICHED (ALL PURPOSE) | Grains and Grain Products | Wheat | DOMESTIC | United States | Non-Detect | 1 | 0 |
| 526284 | 2009 | 5/27/2009 | CORN, WHOLE GRAIN | Grains and Grain Products | Corn | DOMESTIC | United States | Non-Detect | 1 | 0 |
| 526286 | 2009 | 5/27/2009 | CORN, WHOLE GRAIN | Grains and Grain Products | Corn | DOMESTIC | United States | Non-Detect | 1 | 0 |
| 545527 | 2009 | 5/28/2009 | MUNG BEAN | Beans and Legumes | Other Beans and Legumes | IMPORT | India | Non-Detect | 1.7 | 0 |
| 545471 | 2009 | 5/28/2009 | MAYOCOBA BEANS, DRIED OR PASTE | Beans and Legumes | Other Beans and Legumes | IMPORT | Mexico | Non-Detect | 0.233 | 0 |
| 521542 | 2009 | 5/28/2009 | CORN, BOLTED MEAL OR FLOUR | Grains and Grain Products | Corn | DOMESTIC | United States | Non-Detect | 1 | 0 |
| 521543 | 2009 | 6/2/2009 | SEMOLINA, WHEAT | Grains and Grain Products | Wheat | DOMESTIC | United States | Non-Detect | 1.7 | 0 |
| 546862 | 2009 | 6/3/2009 | RICE FLOUR | Grains and Grain Products | Rice | IMPORT | Mexico | Non-Detect | 1.7 | 0 |
| 543375 | 2009 | 6/4/2009 | WHEAT FLOUR, BROMATED | Grains and Grain Products | Wheat | DOMESTIC | United States | Non-Detect | 1 | 0 |
| 543374 | 2009 | 6/4/2009 | RYE FLOUR | Grains and Grain Products | Rye | DOMESTIC | United States | Trace | 1 | |
| 547754 | 2009 | 6/8/2009 | COCOA BEAN N.E.C. | Candy and Chocolate Products | Other Chocolate Products | IMPORT | Indonesia | Non-Detect | 1.7 | 0 |
| 548042 | 2009 | 6/9/2009 | FAVA BEAN, DRIED OR PASTE | Beans and Legumes | Other Beans and Legumes | IMPORT | Mexico | Non-Detect | 1.7 | 0 |
| 548032 | 2009 | 6/9/2009 | PERUANO BEANS, DRIED OR PASTE | Beans and Legumes | Other Beans and Legumes | IMPORT | Mexico | Non-Detect | 0.233 | 0 |
| 548745 | 2009 | 6/11/2009 | FAVA BEAN, DRIED OR PASTE | Beans and Legumes | Other Beans and Legumes | IMPORT | Mexico | Non-Detect | 0.233 | 0 |
| 549789 | 2009 | 6/16/2009 | FAVA BEAN | Beans and Legumes | Other Beans and Legumes | IMPORT | Mexico | Non-Detect | 1.7 | 0 |
| 549766 | 2009 | 6/16/2009 | FLOR DE MAYO BEAN, DRIED OR PASTE | Beans and Legumes | Other Beans and Legumes | IMPORT | Mexico | Non-Detect | 0.233 | 0 |
| 552220 | 2009 | 6/24/2009 | WHEAT FLOUR, WHOLE | Grains and Grain Products | Wheat | IMPORT | Canada | Non-Detect | 1 | 0 |
| 490371 | 2009 | 6/25/2009 | MIXED CEREAL, WITH FRUIT (BABY) | Baby Food Products | Baby Cereals | DOMESTIC | United States | Non-Detect | 0.233 | 0 |
| 534880 | 2009 | 6/26/2009 | PLUM, DRIED OR PASTE | Fruit | Other Dried Fruits | DOMESTIC | United States | Non-Detect | 0.233 | 0 |
| 534881 | 2009 | 6/29/2009 | RAISINS, DRIED OR PASTE | Fruit | Raisins | DOMESTIC | United States | Non-Detect | 0.233 | 0 |
| 554256 | 2009 | 7/1/2009 | WHEAT FLAKES, PUFFS, KRISPIES, LOOPS, SHREDDED, WHEAT GERM READY TO EAT | Processed Food Products | Breakfast Foods | IMPORT | Canada | Non-Detect | 1.7 | 0 |
| 555398 | 2009 | 7/8/2009 | GARBANZO BEAN, DRIED OR PASTE | Beans and Legumes | Garbanzo Beans | IMPORT | Mexico | Non-Detect | 0.233 | 0 |
| 555630 | 2009 | 7/9/2009 | MALT, WHEAT | Grains and Grain Products | Wheat | IMPORT | Germany | Non-Detect | 0.233 | 0 |
| 555880 | 2009 | 7/9/2009 | WHEAT FLAKES, PUFFS, KRISPIES, LOOPS, SHREDDED, WHEAT GERM READY TO EAT | Processed Food Products | Breakfast Foods | IMPORT | Mexico | Non-Detect | 0.233 | 0 |
| 485431 | 2009 | 7/10/2009 | OATS, WHOLE GRAIN | Grains and Grain Products | Oats | IMPORT | United States | Non-Detect | 0.233 | 0 |
| 556530 | 2009 | 7/13/2009 | BRAN FLAKES, SHREDDED AND OTHER FORMS READY TO EAT | Processed Food Products | Breakfast Foods | IMPORT | Mexico | Non-Detect | 0.233 | 0 |
| 556430 | 2009 | 7/15/2009 | WHEAT, WHOLE GRAIN | Grains and Grain Products | Wheat | DOMESTIC | United States | Non-Detect | 1.7 | 0 |
| 556432 | 2009 | 7/15/2009 | WHEAT, WHOLE GRAIN | Grains and Grain Products | Wheat | DOMESTIC | United States | Non-Detect | 1.7 | 0 |
| 556436 | 2009 | 7/15/2009 | WHEAT, WHOLE GRAIN | Grains and Grain Products | Wheat | DOMESTIC | United States | Non-Detect | 1.7 | 0 |
| 556434 | 2009 | 7/15/2009 | WHEAT, WHOLE GRAIN | Grains and Grain Products | Wheat | DOMESTIC | United States | Positive | 1.7 | 6.8 |
| 520863 | 2009 | 7/15/2009 | RYE FLOUR | Grains and Grain Products | Rye | DOMESTIC | United States | Trace | 1.7 | |
| 520866 | 2009 | 7/15/2009 | WHEAT FLOUR, ENRICHED (ALL PURPOSE) | Grains and Grain Products | Wheat | DOMESTIC | United States | Trace | 1.7 | |
| 557953 | 2009 | 7/20/2009 | CORN CHIPS, FRIED | Processed Food Products | Other Corn Products | IMPORT | Mexico | Non-Detect | 0.233 | 0 |
| 557790 | 2009 | 7/21/2009 | BREADS, ROLLS, BUNS, ETC N.E.C. PREPARED DRY MIX WITHOUT MILK OR EGG | Processed Food Products | Bread | DOMESTIC | United States | Trace | 12.3 | |
| 557793 | 2009 | 7/21/2009 | BREADS, ROLLS, BUNS, ETC N.E.C. PREPARED DRY MIX WITHOUT MILK OR EGG | Processed Food Products | Bread | DOMESTIC | United States | Trace | 12.3 | |
| 557795 | 2009 | 7/21/2009 | WHEAT, WHOLE GRAIN | Grains and Grain Products | Wheat | DOMESTIC | United States | Trace | 12.3 | |
| 554770 | 2009 | 7/27/2009 | OATS, WHOLE GRAIN | Grains and Grain Products | Oats | IMPORT | Canada | Non-Detect | 1.7 | |
| 554771 | 2009 | 7/27/2009 | RAISINS, DRIED OR PASTE | Fruit | Raisins | DOMESTIC | United States | Trace | 1.7 | |
| 560842 | 2009 | 7/28/2009 | CHANA DAL, DRIED OR PASTE | Beans and Legumes | Garbanzo Beans | IMPORT | India | Non-Detect | 1.7 | 0 |
| 562517 | 2009 | 8/5/2009 | COFFEE, BEANS, DECAFFEINATED | Non-Juice Beverages | Coffee | IMPORT | Germany | Non-Detect | 1.7 | 0 |
| 562991 | 2009 | 8/6/2009 | OAT FLAKES, ROLLED OATS, PUFFS, KRISPIES, LOOPS READY TO EAT | Processed Food Products | Breakfast Foods | IMPORT | Mexico | Non-Detect | 0.233 | 0 |
| 564724 | 2009 | 8/12/2009 | BUCKWHEAT, WHOLE GRAIN | Grains and Grain Products | Buckwheat | IMPORT | Russia | Non-Detect | 1.7 | 0 |
| 565072 | 2009 | 8/13/2009 | CORN, BOLTED MEAL OR FLOUR | Grains and Grain Products | Corn | IMPORT | Mexico | Non-Detect | 0.233 | 0 |
| 566153 | 2009 | 8/18/2009 | BARLEY, WHOLE GRAIN | Grains and Grain Products | Barley | IMPORT | India | Non-Detect | 1.7 | 0 |
| 563251 | 2009 | 8/25/2009 | RAISINS, DRIED OR PASTE | Fruit | Raisins | DOMESTIC | United States | Non-Detect | 0.233 | 0 |
| 494398 | 2009 | 8/26/2009 | WHEAT FLOUR, ENRICHED (ALL PURPOSE) | Grains and Grain Products | Wheat | DOMESTIC | United States | Non-Detect | 2.3 | 0 |
| 570344 | 2009 | 9/1/2009 | RAISINS, DRIED OR PASTE | Fruit | Raisins | IMPORT | Mexico | Non-Detect | 0.233 | 0 |
| 556454 | 2009 | 9/3/2009 | FARINA, WHEAT | Grains and Grain Products | Wheat | DOMESTIC | United States | Non-Detect | 1.7 | 0 |
| 556457 | 2009 | 9/3/2009 | WHEAT FLOUR, ENRICHED (ALL PURPOSE) | Grains and Grain Products | Wheat | DOMESTIC | United States | Non-Detect | 1.7 | 0 |
| 564185 | 2009 | 9/4/2009 | WHEAT FLOUR, ENRICHED (ALL PURPOSE) | Grains and Grain Products | Wheat | DOMESTIC | United States | Trace | 0.233 | |
| 571228 | 2009 | 9/4/2009 | COOKIE, BISCUIT, WAFER DOUGH, N.E.C. | Processed Food Products | Other Bakery Products | IMPORT | Mexico | Trace | 0.5 | |
| 556399 | 2009 | 9/18/2009 | CORN, WHOLE GRAIN | Grains and Grain Products | Corn | DOMESTIC | United States | Non-Detect | 0.233 | 0 |
| 556401 | 2009 | 9/18/2009 | CORN, WHOLE GRAIN | Grains and Grain Products | Corn | DOMESTIC | United States | Non-Detect | 0.233 | 0 |
| 564186 | 2009 | 9/21/2009 | COFFEE, BEANS | Non-Juice Beverages | Coffee | IMPORT | Guatemala | Non-Detect | 1 | 0 |
| 558006 | 2009 | 9/22/2009 | WHEAT FLOUR, WHOLE | Grains and Grain Products | Wheat | DOMESTIC | Unknown | Trace | 1 | |
| 558007 | 2009 | 9/22/2009 | WHEAT FLOUR, ENRICHED (ALL PURPOSE) | Grains and Grain Products | Wheat | DOMESTIC | Unknown | Trace | 1 | |
| 539385 | 2009 | 9/24/2009 | WHEAT FLOUR, BROMATED | Grains and Grain Products | Wheat | DOMESTIC | United States | Non-Detect | 1.7 | 0 |
| 575804 | 2009 | 9/25/2009 | CORN, BOLTED MEAL OR FLOUR | Grains and Grain Products | Corn | IMPORT | Ghana | Trace | 1 | |
| 547303 | 2009 | 9/29/2009 | RICE CEREAL (BABY) | Baby Food Products | Baby Cereals | DOMESTIC | United States | Non-Detect | 1.7 | 0 |
| 575438 | 2009 | 9/29/2009 | SOYBEANS, WHOLE GRAIN | Beans and Legumes | Soybeans | DOMESTIC | United States | Non-Detect | 1.7 | 0 |
| 575440 | 2009 | 9/29/2009 | SOYBEANS, WHOLE GRAIN | Beans and Legumes | Soybeans | DOMESTIC | United States | Non-Detect | 1.7 | 0 |
| 575442 | 2009 | 9/29/2009 | SOYBEANS, WHOLE GRAIN | Beans and Legumes | Soybeans | DOMESTIC | United States | Non-Detect | 1.7 | 0 |
| 575444 | 2009 | 9/29/2009 | WHEAT, WHOLE GRAIN | Grains and Grain Products | Wheat | DOMESTIC | United States | Non-Detect | 1.7 | 0 |

## FY2010 Data

| Sample Number | Fiscal Year | Collection Date | Product Name | Food Category | Food Subcategory | Origin Type | Country of Origin | Non-Detect, Trace, or Positive (>LOQ) | LOQ (ppb) | Amount found (ppb) |
|---|---|---|---|---|---|---|---|---|---|---|
| 499831 | 2010 | 10/7/2009 | WHEAT FLOUR, BROMATED | Grains and Grain Products | Wheat | DOMESTIC | Unknown | Non-Detect | 1.7 | 0 |
| 527654 | 2010 | 10/7/2009 | WHEAT FLOUR, ENRICHED (ALL PURPOSE) | Grains and Grain Products | Wheat | DOMESTIC | United States | Non-Detect | 1.7 | 0 |
| 578829 | 2010 | 10/7/2009 | WHEAT FLAKES, PUFFS, KRISPIES, LOOPS, SHREDDED, WHEAT GERM READY TO EAT | Processed Food Products | Breakfast Foods | IMPORT | United Kingdom | Trace | 1 | |
| 579696 | 2010 | 10/13/2009 | RICE FLAKES, PUFFS, KRISPIES, LOOPS READY TO EAT | Processed Food Products | Breakfast Foods | IMPORT | Mexico | Non-Detect | 1.7 | 0 |
| 579824 | 2010 | 10/13/2009 | BUTTER/BUTTER FLAVORED, PLAIN COOKIES, BISCUITS AND WAFERS | Processed Food Products | Other Bakery Products | IMPORT | Mexico | Non-Detect | 1.7 | 0 |
| 521240 | 2010 | 10/14/2009 | CORN, HOMINY GRITS | Grains and Grain Products | Corn | DOMESTIC | United States | Non-Detect | 1.7 | 0 |
| 564180 | 2010 | 10/14/2009 | CORN, PLAIN MEAL | Grains and Grain Products | Corn | DOMESTIC | United States | Non-Detect | 1.7 | 0 |
| 578219 | 2010 | 10/14/2009 | CORN, BOLTED MEAL OR FLOUR | Grains and Grain Products | Corn | DOMESTIC | United States | Non-Detect | 1.7 | 0 |
| 490379 | 2010 | 10/16/2009 | OATS, WHOLE GRAIN | Grains and Grain Products | Oats | DOMESTIC | United States | Non-Detect | 1.7 | 0 |
| 490380 | 2010 | 10/16/2009 | RICE CEREAL (BABY) | Baby Food Products | Baby Cereals | DOMESTIC | United States | Non-Detect | 1.7 | 0 |
| 581088 | 2010 | 10/19/2009 | VERMICELLI | Processed Food Products | Pasta | IMPORT | Mexico | Non-Detect | 1.7 | 0 |
| 581093 | 2010 | 10/19/2009 | SPAGHETTI | Processed Food Products | Pasta | IMPORT | Mexico | Non-Detect | 1.7 | 0 |
| 581421 | 2010 | 10/20/2009 | YELLOW BEANS, DRIED OR PASTE | Beans and Legumes | Other Beans and Legumes | IMPORT | Mexico | Non-Detect | 1.7 | 0 |
| 583236 | 2010 | 10/25/2009 | BLACKEYE PEAS, DRIED OR PASTE | Beans and Legumes | Blackeye Peas | IMPORT | Peru | Non-Detect | 1.7 | 0 |
| 583613 | 2010 | 10/27/2009 | COFFEE, BEANS, DECAFFEINATED | Non-Juice Beverages | Coffee | IMPORT | Italy | Non-Detect | 1.7 | 0 |
| 583685 | 2010 | 10/27/2009 | OATS, WHOLE GRAIN | Grains and Grain Products | Oats | IMPORT | Canada | Non-Detect | 1.7 | 0 |
| 584678 | 2010 | 10/27/2009 | CORN, BOLTED MEAL OR FLOUR | Grains and Grain Products | Corn | IMPORT | France | Trace | 1 | |
| 584073 | 2010 | 10/28/2009 | WHEAT FLOUR, ENRICHED (ALL PURPOSE) | Grains and Grain Products | Wheat | IMPORT | Canada | Non-Detect | 1.7 | 0 |
| 584075 | 2010 | 10/28/2009 | ZWEIBACK, TOAST | Processed Food Products | Other Bakery Products | IMPORT | Mexico | Non-Detect | 1.7 | 0 |
| 583006 | 2010 | 10/29/2009 | CORN GRITS, BREWERS ENRICHED WHITE OR YELLOW CORN GRITS, CORN MEAL MUSH, QUICK OR INSTANT COOKING | Processed Food Products | Other Corn Products | DOMESTIC | United States | Non-Detect | 1.7 | 0 |
| 584575 | 2010 | 10/29/2009 | MACARONI | Processed Food Products | Pasta | IMPORT | Mexico | Non-Detect | 1.7 | 0 |
| 546171 | 2010 | 10/29/2009 | WHEAT FLOUR, ENRICHED (ALL PURPOSE) | Grains and Grain Products | Wheat | DOMESTIC | United States | Trace | 1 | |
| 584572 | 2010 | 10/29/2009 | MACARONI | Processed Food Products | Pasta | IMPORT | Mexico | Trace | 1 | |
| 584847 | 2010 | 10/30/2009 | CORN, DRIED OR PASTE | Grains and Grain Products | Corn | IMPORT | Vietnam | Trace | 1 | |
| 585044 | 2010 | 10/30/2009 | GRAM FLOUR | Beans and Legumes | Garbanzo Beans | IMPORT | India | Trace | 1 | |
| 495189 | 2010 | 11/2/2009 | MIXED CEREAL FLAKES, SHREDDED AND OTHER FORMS READY TO EAT | Processed Food Products | Breakfast Foods | DOMESTIC | United States | Non-Detect | 1.7 | 0 |
| 585271 | 2010 | 11/2/2009 | WHEAT FLOUR, WHOLE | Grains and Grain Products | Wheat | IMPORT | Canada | Trace | 1 | |
| 585516 | 2010 | 11/3/2009 | WHEAT, WHOLE GRAIN | Grains and Grain Products | Wheat | IMPORT | Canada | Non-Detect | 1.7 | 0 |
| 587027 | 2010 | 11/4/2009 | WHEAT FLOUR, ENRICHED (ALL PURPOSE) | Grains and Grain Products | Wheat | IMPORT | India | Non-Detect | 1.7 | 0 |
| 586274 | 2010 | 11/4/2009 | COFFEE, BEANS | Non-Juice Beverages | Coffee | IMPORT | Guatemala | Non-Detect | 1 | 0 |
| 490860 | 2010 | 11/5/2009 | CORN, PLAIN MEAL | Grains and Grain Products | Corn | DOMESTIC | United States | Non-Detect | 1 | 0 |
| 586745 | 2010 | 11/6/2009 | BLACK BEAN, DRIED OR PASTE | Beans and Legumes | Black Beans | IMPORT | Canada | Non-Detect | 1.7 | 0 |
| 485417 | 2010 | 11/6/2009 | WHEAT FLOUR, WHOLE | Grains and Grain Products | Wheat | DOMESTIC | United States | Trace | 1 | |
| 587335 | 2010 | 11/9/2009 | OATS, WHOLE GRAIN | Grains and Grain Products | Oats | IMPORT | Canada | Positive | 1.7 | 2.4 |
| 586432 | 2010 | 11/12/2009 | CORN, BOLTED MEAL OR FLOUR | Grains and Grain Products | Corn | DOMESTIC | United States | Non-Detect | 1.7 | 0 |
| 588021 | 2010 | 11/12/2009 | GARBANZO BEAN, DRIED OR PASTE | Beans and Legumes | Garbanzo Beans | IMPORT | Mexico | Non-Detect | 1.7 | 0 |
| 584809 | 2010 | 11/12/2009 | MUFFIN (BLUEBERRY, ETC) WITHOUT CUSTARD OR CREAM FILLING | Processed Food Products | Other Bakery Products | DOMESTIC | United States | Positive | 1.7 | 1.7 |
| 588289 | 2010 | 11/13/2009 | PINTO BEAN, DRIED OR PASTE | Beans and Legumes | Pinto Beans | IMPORT | Mexico | Non-Detect | 1.7 | 0 |
| 588318 | 2010 | 11/13/2009 | CORN, WHOLE GRAIN | Grains and Grain Products | Corn | IMPORT | Mexico | Non-Detect | 1.7 | 0 |
| 588700 | 2010 | 11/16/2009 | GARBANZO BEAN, DRIED OR PASTE | Beans and Legumes | Garbanzo Beans | IMPORT | Canada | Non-Detect | 1.7 | 0 |
| 588837 | 2010 | 11/16/2009 | GARBANZO BEAN, DRIED OR PASTE | Beans and Legumes | Garbanzo Beans | IMPORT | Mexico | Non-Detect | 1.7 | 0 |
| 589408 | 2010 | 11/17/2009 | TORTILLAS | Processed Food Products | Other Corn Products | IMPORT | Mexico | Non-Detect | 1.7 | 0 |
| 589887 | 2010 | 11/17/2009 | PINTO BEAN | Beans and Legumes | Pinto Beans | IMPORT | Canada | Non-Detect | 1.7 | 0 |
| 589885 | 2010 | 11/18/2009 | PERUANO BEANS, DRIED OR PASTE | Beans and Legumes | Other Beans and Legumes | IMPORT | Mexico | Non-Detect | 1.7 | 0 |
| 541033 | 2010 | 11/19/2009 | WHEAT FLOUR, ENRICHED (ALL PURPOSE) | Grains and Grain Products | Wheat | DOMESTIC | United States | Non-Detect | 1.7 | 0 |
| 590416 | 2010 | 11/19/2009 | WHEAT, MILLED (CRUSHED, COARSE GROUND OR CRACKED) | Grains and Grain Products | Wheat | IMPORT | Turkey | Non-Detect | 1 | 0 |
| 576273 | 2010 | 11/24/2009 | WHEAT FLOUR, BROMATED | Grains and Grain Products | Wheat | DOMESTIC | United States | Non-Detect | 1 | 0 |
| 576275 | 2010 | 11/24/2009 | WHEAT FLOUR, BROMATED | Grains and Grain Products | Wheat | DOMESTIC | United States | Positive | 1 | 1.81 |
| 591251 | 2010 | 11/25/2009 | WHEAT, WHOLE GRAIN | Grains and Grain Products | Wheat | IMPORT | Canada | Non-Detect | 1 | 0 |
| 488827 | 2010 | 12/1/2009 | CORN GRITS, BREWERS ENRICHED WHITE OR YELLOW CORN GRITS, CORN MEAL MUSH, QUICK OR INSTANT COOKING | Processed Food Products | Other Corn Products | DOMESTIC | United States | Non-Detect | 1 | 0 |
| 572932 | 2010 | 12/1/2009 | WHEAT FLOUR, WHOLE | Grains and Grain Products | Wheat | DOMESTIC | United States | Non-Detect | 1 | 0 |
| 583834 | 2010 | 12/2/2009 | RAISINS (DRIED GRAPES) (BERRY) | Fruit | Raisins | DOMESTIC | United States | Positive | 1 | 1.78 |
| 593365 | 2010 | 12/4/2009 | WHEAT FLOUR, ENRICHED (ALL PURPOSE) | Grains and Grain Products | Wheat | IMPORT | Korea (the Republic of) | Trace | 1 | |
| 484936 | 2010 | 12/7/2009 | WHEAT FLOUR, WHOLE | Grains and Grain Products | Wheat | DOMESTIC | United States | Non-Detect | 1 | 0 |
| 593998 | 2010 | 12/8/2009 | ZWEIBACK, TOAST | Processed Food Products | Other Bakery Products | IMPORT | Mexico | Non-Detect | 1 | 0 |
| 594082 | 2010 | 12/8/2009 | FARINA, WHEAT | Grains and Grain Products | Wheat | IMPORT | India | Non-Detect | 1 | 0 |
| 594083 | 2010 | 12/8/2009 | WHEAT FLOUR, ENRICHED (ALL PURPOSE) | Grains and Grain Products | Wheat | IMPORT | India | Trace | 1 | |
| 595103 | 2010 | 12/14/2009 | WHEAT FLOUR, GLUTEN | Grains and Grain Products | Wheat | IMPORT | Italy | Non-Detect | 1 | 0 |
| 595429 | 2010 | 12/15/2009 | WHEAT FLOUR, WHOLE | Grains and Grain Products | Wheat | IMPORT | India | Non-Detect | 1 | 0 |
| 595620 | 2010 | 12/16/2009 | WHEAT FLOUR, ENRICHED (ALL PURPOSE) | Grains and Grain Products | Wheat | IMPORT | Canada | Trace | 1 | |
| 596157 | 2010 | 12/18/2009 | BEANS, CORN, AND PEA, N.E.C. (VEGETABLE) | Beans and Legumes | Other Beans and Legumes | IMPORT | Mexico | Non-Detect | 0.233 | 0 |
| 596122 | 2010 | 12/18/2009 | VANILLA PLAIN COOKIE, BISCUIT AND WAFER | Processed Food Products | Other Bakery Products | IMPORT | Thailand | Non-Detect | 1 | 0 |
| 597375 | 2010 | 12/29/2009 | GARBANZO BEAN | Beans and Legumes | Garbanzo Beans | IMPORT | India | Non-Detect | 1 | 0 |
| 437736 | 2010 | 1/11/2010 | SOYBEANS, WHOLE GRAIN | Beans and Legumes | Soybeans | DOMESTIC | United States | Non-Detect | 2.9 | 0 |
| 504482 | 2010 | 1/11/2010 | SOYBEAN FLOUR | Beans and Legumes | Soybeans | DOMESTIC | United States | Non-Detect | 2.9 | 0 |
| 504483 | 2010 | 1/11/2010 | SOYBEAN FLOUR | Beans and Legumes | Soybeans | DOMESTIC | United States | Non-Detect | 2.9 | 0 |
| 504484 | 2010 | 1/11/2010 | CORN, BOLTED MEAL OR FLOUR | Grains and Grain Products | Corn | DOMESTIC | United States | Non-Detect | 2.9 | 0 |
| 504486 | 2010 | 1/11/2010 | FLOURS AND MEALS N.E.C. | Grains and Grain Products | Other Grains | DOMESTIC | United States | Non-Detect | 2.9 | 0 |
| 574290 | 2010 | 1/11/2010 | SOYBEANS, WHOLE GRAIN | Beans and Legumes | Soybeans | DOMESTIC | United States | Non-Detect | 2.9 | 0 |
| 504481 | 2010 | 1/12/2010 | CORN, BOLTED MEAL OR FLOUR | Grains and Grain Products | Corn | DOMESTIC | United States | Non-Detect | 2.9 | 0 |
| 504485 | 2010 | 1/12/2010 | FLOURS AND MEALS N.E.C. | Grains and Grain Products | Other Grains | DOMESTIC | United States | Non-Detect | 2.9 | 0 |
| 600310 | 2010 | 1/14/2010 | COFFEE, BEANS | Non-Juice Beverages | Coffee | IMPORT | Canada | Non-Detect | 2.6 | 0 |
| 600566 | 2010 | 1/19/2010 | SOY FIBER (MEAT EXTENDERS | Other | Other | IMPORT | Mexico | Positive | 0.233 | 1.43 |
| 601132 | 2010 | 1/21/2010 | OAT CEREAL (BABY) | Baby Food Products | Baby Cereals | DOMESTIC | United States | Non-Detect | 2.6 | 0 |
| 548727 | 2010 | 1/21/2010 | PINTO BEAN | Beans and Legumes | Pinto Beans | DOMESTIC | United States | Positive | 2.9 | 6 |
| 601911 | 2010 | 1/22/2010 | OAT FLAKES, ROLLED OATS, PUFFS, KRISPIES, LOOPS READY TO EAT | Processed Food Products | Breakfast Foods | IMPORT | Mexico | Non-Detect | 0.233 | 0 |
| 603446 | 2010 | 1/26/2010 | NUT FILLED/ICED COOKIES, BISCUITS AND WAFERS | Processed Food Products | Other Bakery Products | IMPORT | Taiwan | Non-Detect | 2.6 | 0 |
| 300765 | 2010 | 1/27/2010 | PINTO BEAN | Beans and Legumes | Pinto Beans | IMPORT | Canada | Trace | 2.9 | |
| 515776 | 2010 | 1/28/2010 | RYE FLOUR | Grains and Grain Products | Rye | DOMESTIC | United States | Non-Detect | 2.9 | 0 |
| 605126 | 2010 | 2/2/2010 | OAT FLOUR | Grains and Grain Products | Oats | IMPORT | Canada | Non-Detect | 2.6 | 0 |
| 566897 | 2010 | 2/2/2010 | CORN, DEGERMINATED MEAL | Grains and Grain Products | Corn | DOMESTIC | United States | Non-Detect | 1 | 0 |
| 605503 | 2010 | 2/2/2010 | CORN, SELF RISING BOLTED MEAL | Grains and Grain Products | Corn | IMPORT | India | Positive | 1 | 1.9 |
| 605792 | 2010 | 2/5/2010 | TEMPURA FLOUR MIX | Grains and Grain Products | Other Grains | IMPORT | Canada | Non-Detect | 1 | 0 |
| 603111 | 2010 | 2/8/2010 | OATS, WHOLE GRAIN | Grains and Grain Products | Oats | DOMESTIC | United States | Non-Detect | 2.6 | 0 |
| 606480 | 2010 | 2/9/2010 | COFFEE, BEANS | Non-Juice Beverages | Coffee | IMPORT | Colombia | Non-Detect | 2.6 | 0 |
| 518625 | 2010 | 2/11/2010 | PINTO BEAN | Beans and Legumes | Pinto Beans | DOMESTIC | United States | Non-Detect | 2.9 | 0 |
| 607101 | 2010 | 2/11/2010 | OATS, WHOLE GRAIN | Grains and Grain Products | Oats | IMPORT | Ireland | Non-Detect | 2.6 | 0 |
| 607444 | 2010 | 2/12/2010 | LIMA BEAN, DRIED OR PASTE | Beans and Legumes | Other Beans and Legumes | IMPORT | Canada | Non-Detect | 2.6 | 0 |

| | | | | | | | | | | |
|---|---|---|---|---|---|---|---|---|---|---|
| 607906 | 2010 | 2/16/2010 | COFFEE, BEANS | Non-Juice Beverages | Coffee | IMPORT | El Salvador | Non-Detect | 2.6 | 0 |
| 601356 | 2010 | 2/17/2010 | RICE CEREAL (BABY) | Baby Food Products | Baby Cereals | DOMESTIC | United States | Non-Detect | 2.6 | 0 |
| 609548 | 2010 | 2/18/2010 | NAVY (WHITE) BEANS (BAKED BEANS, PORK AND BEANS), DRIED OR PASTE | Beans and Legumes | Other Beans and Legumes | IMPORT | Canada | Non-Detect | 2.6 | 0 |
| 609603 | 2010 | 2/22/2010 | OATMEAL, REGULAR, FRUIT OR SPICE ADDED, QUICK OR INSTANT COOKING | Processed Food Products | Breakfast Foods | IMPORT | Dominican Republic (the) | Non-Detect | 2.6 | 0 |
| 609533 | 2010 | 2/22/2010 | CORN FLAKES, PUFFS, KRISPIES, LOOPS READY TO EAT | Processed Food Products | Other Corn Products | IMPORT | Canada | Non-Detect | 1 | 0 |
| 610256 | 2010 | 2/23/2010 | MUNG BEAN, DRIED OR PASTE | Beans and Legumes | Other Beans and Legumes | IMPORT | Thailand | Non-Detect | 2.6 | 0 |
| 599977 | 2010 | 2/26/2010 | BLACK BEAN, DRIED OR PASTE | Beans and Legumes | Black Beans | DOMESTIC | United States | Non-Detect | 1 | 0 |
| 599978 | 2010 | 2/26/2010 | RAISINS, DRIED OR PASTE | Fruit | Raisins | DOMESTIC | United States | Trace | 1 | |
| 612156 | 2010 | 3/2/2010 | WHEAT FLAKES, PUFFS, KRISPIES, LOOPS, SHREDDED, WHEAT GERM READY TO EAT | Processed Food Products | Breakfast Foods | IMPORT | Canada | Non-Detect | 2.6 | 0 |
| 612481 | 2010 | 3/3/2010 | BRAN FLAKES, SHREDDED AND OTHER FORMS READY TO EAT | Processed Food Products | Breakfast Foods | IMPORT | Canada | Non-Detect | 2.6 | 0 |
| 613796 | 2010 | 3/9/2010 | MIXED CEREAL, QUICK OR INSTANT COOKING | Processed Food Products | Breakfast Foods | IMPORT | Guatemala | Trace | 1 | |
| 556625 | 2010 | 3/10/2010 | WHEAT FLOUR, WHOLE | Grains and Grain Products | Wheat | DOMESTIC | United States | Non-Detect | 1 | 0 |
| 614347 | 2010 | 3/10/2010 | GARBANZO BEAN | Beans and Legumes | Garbanzo Beans | IMPORT | India | Non-Detect | 1 | 0 |
| 615382 | 2010 | 3/17/2010 | MALT, BARLEY | Grains and Grain Products | Barley | IMPORT | Canada | Non-Detect | 1 | 0 |
| 615574 | 2010 | 3/18/2010 | OATMEAL FILLED/ICED COOKIES, BISCUITS AND WAFERS | Processed Food Products | Other Bakery Products | IMPORT | Canada | Trace | 2.2 | |
| 615584 | 2010 | 3/18/2010 | OATMEAL PLAIN COOKIES, BISCUITS, AND WAFERS | Processed Food Products | Other Bakery Products | IMPORT | Canada | Trace | 2.2 | |
| 616402 | 2010 | 3/23/2010 | COFFEE, BEANS | Non-Juice Beverages | Coffee | IMPORT | Italy | Non-Detect | 2.6 | 0 |
| 616419 | 2010 | 3/23/2010 | COFFEE, BEANS | Non-Juice Beverages | Coffee | IMPORT | Italy | Non-Detect | 2.6 | 0 |
| 616552 | 2010 | 3/23/2010 | OATMEAL COOKIE, BISCUIT, WAFER DOUGH | Processed Food Products | Other Bakery Products | IMPORT | Canada | Non-Detect | 2.6 | 0 |
| 616895 | 2010 | 3/25/2010 | OAT FLAKES, ROLLED OATS, PUFFS, KRISPIES, LOOPS READY TO EAT | Processed Food Products | Breakfast Foods | IMPORT | Dominican Republic (the) | Non-Detect | 2.6 | 0 |
| 576702 | 2010 | 3/26/2010 | CORN GRITS, BREWERS ENRICHED WHITE OR YELLOW CORN GRITS, CORN MEAL MUSH, QUICK OR INSTANT COOKING | Processed Food Products | Other Corn Products | DOMESTIC | United States | Non-Detect | 1 | 0 |
| 617334 | 2010 | 3/28/2010 | MUNG BEAN, DRIED OR PASTE | Beans and Legumes | Other Beans and Legumes | IMPORT | Canada | Non-Detect | 2.6 | 0 |
| 617336 | 2010 | 3/28/2010 | MUNG BEAN, DRIED OR PASTE | Beans and Legumes | Other Beans and Legumes | IMPORT | Canada | Non-Detect | 2.6 | 0 |
| 617344 | 2010 | 3/28/2010 | MUNG BEAN, DRIED OR PASTE | Beans and Legumes | Other Beans and Legumes | IMPORT | Canada | Non-Detect | 2.6 | 0 |
| 617347 | 2010 | 3/28/2010 | LENTILS, DRIED OR PASTE | Beans and Legumes | Lentils | IMPORT | Canada | Non-Detect | 2.6 | 0 |
| 617358 | 2010 | 3/28/2010 | GARBANZO BEAN, DRIED OR PASTE | Beans and Legumes | Garbanzo Beans | IMPORT | Canada | Non-Detect | 2.6 | 0 |
| 617364 | 2010 | 3/28/2010 | GARBANZO BEAN, DRIED OR PASTE | Beans and Legumes | Garbanzo Beans | IMPORT | Canada | Non-Detect | 2.6 | 0 |
| 617366 | 2010 | 3/28/2010 | BEAN, CORN, PEA, DRIED OR PASTE, N.E.C. (VEGETABLE) | Beans and Legumes | Other Beans and Legumes | IMPORT | Canada | Non-Detect | 2.6 | 0 |
| 617556 | 2010 | 3/29/2010 | COFFEE, BEANS | Non-Juice Beverages | Coffee | IMPORT | Vietnam | Non-Detect | 2.6 | 0 |
| 576704 | 2010 | 3/31/2010 | NAVY (WHITE) BEANS (BAKED BEANS, PORK AND BEANS), DRIED OR PASTE | Beans and Legumes | Other Beans and Legumes | DOMESTIC | United States | Non-Detect | 2.6 | 0 |
| 618077 | 2010 | 3/31/2010 | COFFEE, BEANS | Non-Juice Beverages | Coffee | IMPORT | Dominican Republic (the) | Non-Detect | 2.6 | 0 |
| 618414 | 2010 | 3/31/2010 | GARBANZO BEAN, DRIED OR PASTE | Beans and Legumes | Garbanzo Beans | IMPORT | Turkey | Non-Detect | 2.6 | 0 |
| 618473 | 2010 | 3/31/2010 | GARBANZO BEAN, DRIED OR PASTE | Beans and Legumes | Garbanzo Beans | IMPORT | Turkey | Non-Detect | 2.6 | 0 |
| 618526 | 2010 | 3/31/2010 | GARBANZO BEAN, DRIED OR PASTE | Beans and Legumes | Garbanzo Beans | IMPORT | Turkey | Non-Detect | 2.6 | 0 |
| 618533 | 2010 | 3/31/2010 | GARBANZO BEAN, DRIED OR PASTE | Beans and Legumes | Garbanzo Beans | IMPORT | Turkey | Non-Detect | 2.6 | 0 |
| 619135 | 2010 | 4/7/2010 | CORN FLAKES, PUFFS, KRISPIES, LOOPS READY TO EAT | Processed Food Products | Other Corn Products | IMPORT | Canada | Positive | 1 | 1.03 |
| 619916 | 2010 | 4/9/2010 | BUCKWHEAT, WHOLE GRAIN | Grains and Grain Products | Buckwheat | IMPORT | Russia | Trace | 1 | |
| 600377 | 2010 | 4/12/2010 | WHEAT FLOUR, BROMATED | Grains and Grain Products | Wheat | DOMESTIC | United States | Non-Detect | 2.6 | 0 |
| 620243 | 2010 | 4/13/2010 | COFFEE, BEANS | Non-Juice Beverages | Coffee | IMPORT | Honduras | Non-Detect | 2.6 | 0 |
| 620873 | 2010 | 4/13/2010 | BEAN, CORN, PEA, DRIED OR PASTE, N.E.C. (VEGETABLE) | Beans and Legumes | Other Beans and Legumes | IMPORT | Nigeria | Non-Detect | 0.233 | 0 |
| 621496 | 2010 | 4/20/2010 | COFFEE, BEANS | Non-Juice Beverages | Coffee | IMPORT | Tanzania, United Republic Of | Non-Detect | 2.6 | 0 |
| 623142 | 2010 | 4/21/2010 | MIXED CEREAL, QUICK OR INSTANT COOKING | Processed Food Products | Breakfast Foods | IMPORT | United Kingdom | Non-Detect | 0.233 | 0 |
| 622148 | 2010 | 4/23/2010 | CORN, PLAIN MEAL | Grains and Grain Products | Corn | IMPORT | China | Non-Detect | 2.6 | 0 |
| 622317 | 2010 | 4/26/2010 | COFFEE, BEANS | Non-Juice Beverages | Coffee | IMPORT | India | Non-Detect | 2.6 | 0 |
| 481570 | 2010 | 4/29/2010 | WHEAT FLOUR, GLUTEN | Grains and Grain Products | Wheat | DOMESTIC | United States | Non-Detect | 1 | 0 |
| 623555 | 2010 | 5/1/2010 | WHEAT FLOUR, ENRICHED (ALL PURPOSE) | Grains and Grain Products | Wheat | IMPORT | Canada | Non-Detect | 1 | 0 |
| 623744 | 2010 | 5/3/2010 | BARLEY, WHOLE GRAIN | Grains and Grain Products | Barley | IMPORT | India | Non-Detect | 1 | 0 |
| 624945 | 2010 | 5/10/2010 | LUPIN BEAN, DRIED OR PASTE | Beans and Legumes | Other Beans and Legumes | IMPORT | Portugal | Non-Detect | 2.6 | 0 |
| 624962 | 2010 | 5/10/2010 | PEA, DRIED OR PASTE | Beans and Legumes | Peas | IMPORT | Canada | Non-Detect | 2.6 | 0 |
| 625550 | 2010 | 5/12/2010 | CORN MUFFIN PREPARED DRY MIX WITH MILK OR EGG | Processed Food Products | Other Corn Products | IMPORT | Canada | Non-Detect | 2.6 | 0 |
| 626190 | 2010 | 5/18/2010 | LENTILS | Beans and Legumes | Lentils | IMPORT | Turkey | Non-Detect | 2.6 | 0 |
| 531799 | 2010 | 5/18/2010 | WHEAT FLOUR, ENRICHED (ALL PURPOSE) | Grains and Grain Products | Wheat | DOMESTIC | United States | Non-Detect | 0.233 | 0 |
| 627322 | 2010 | 5/21/2010 | WHEAT FLAKES, PUFFS, KRISPIES, LOOPS, SHREDDED, WHEAT GERM READY TO EAT | Processed Food Products | Breakfast Foods | IMPORT | Canada | Non-Detect | 2.6 | 0 |
| 630512 | 2010 | 6/9/2010 | BEVERAGE BASE OF NON FRUIT ORIGIN, N.E.C. | Non-Juice Beverages | Other Beverages | IMPORT | El Salvador | Non-Detect | 2.6 | 0 |
| 630623 | 2010 | 6/9/2010 | BARLEY, WHOLE GRAIN | Grains and Grain Products | Barley | IMPORT | Korea (the Republic of) | Non-Detect | 2.6 | 0 |
| 630446 | 2010 | 6/9/2010 | SEMOLINA, WHEAT | Grains and Grain Products | Wheat | IMPORT | Israel | Trace | 1 | |
| 630457 | 2010 | 6/9/2010 | MACARONI, WHOLE WHEAT | Processed Food Products | Pasta | IMPORT | Israel | Trace | 1 | |
| 630474 | 2010 | 6/9/2010 | SPAGHETTI | Processed Food Products | Pasta | IMPORT | Israel | Trace | 1 | |
| 630931 | 2010 | 6/15/2010 | RAISINS, DRIED OR PASTE | Fruit | Raisins | IMPORT | Afghanistan | Non-Detect | 0.233 | 0 |
| 632375 | 2010 | 6/22/2010 | COFFEE, BEANS | Non-Juice Beverages | Coffee | IMPORT | Nicaragua | Non-Detect | 2.6 | 0 |
| 599761 | 2010 | 6/29/2010 | CORN, BOLTED MEAL OR FLOUR | Grains and Grain Products | Corn | DOMESTIC | United States | Non-Detect | 1 | 0 |
| 599762 | 2010 | 6/29/2010 | CORN, BOLTED MEAL OR FLOUR | Grains and Grain Products | Corn | DOMESTIC | United States | Non-Detect | 1 | 0 |
| 599763 | 2010 | 6/29/2010 | CORN, BOLTED MEAL OR FLOUR | Grains and Grain Products | Corn | DOMESTIC | United States | Non-Detect | 1 | 0 |
| 599764 | 2010 | 6/29/2010 | CORN, BOLTED MEAL OR FLOUR | Grains and Grain Products | Corn | DOMESTIC | United States | Non-Detect | 1 | 0 |
| 626158 | 2010 | 6/29/2010 | WHEAT FLOUR, GLUTEN | Grains and Grain Products | Wheat | DOMESTIC | United States | Positive | 1 | 2.18 |
| 634071 | 2010 | 7/1/2010 | BARLEY, WHOLE GRAIN | Grains and Grain Products | Barley | IMPORT | Ethiopia | Non-Detect | 2.6 | 0 |
| 512530 | 2010 | 7/2/2010 | MIXED CEREAL FLAKES, SHREDDED AND OTHER FORMS READY TO EAT | Processed Food Products | Breakfast Foods | DOMESTIC | United States | Non-Detect | 2.9 | 0 |
| 512531 | 2010 | 7/2/2010 | CORN FLAKES, PUFFS, KRISPIES, LOOPS READY TO EAT | Processed Food Products | Other Corn Products | DOMESTIC | United States | Non-Detect | 2.9 | 0 |
| 629125 | 2010 | 7/7/2010 | WHEAT FLOUR, BROMATED | Grains and Grain Products | Wheat | DOMESTIC | United States | Non-Detect | 2.6 | 0 |
| 635134 | 2010 | 7/9/2010 | OAT FLAKES, ROLLED OATS, PUFFS, KRISPIES, LOOPS READY TO EAT | Processed Food Products | Breakfast Foods | IMPORT | Canada | Non-Detect | 1 | 0 |
| 635141 | 2010 | 7/10/2010 | GARBANZO BEAN, DRIED OR PASTE | Beans and Legumes | Garbanzo Beans | IMPORT | Canada | Non-Detect | 2.6 | 0 |
| 635150 | 2010 | 7/10/2010 | KIDNEY BEAN, DRIED OR PASTE | Beans and Legumes | Other Beans and Legumes | IMPORT | Thailand | Non-Detect | 2.6 | 0 |
| 635923 | 2010 | 7/12/2010 | COFFEE, BEANS | Non-Juice Beverages | Coffee | IMPORT | Guatemala | Non-Detect | 2.6 | 0 |
| 635924 | 2010 | 7/12/2010 | COFFEE, BEANS | Non-Juice Beverages | Coffee | IMPORT | El Salvador | Non-Detect | 2.6 | 0 |
| 635657 | 2010 | 7/13/2010 | BARLEY, WHOLE GRAIN | Grains and Grain Products | Barley | IMPORT | Korea (the Republic of) | Non-Detect | 2.6 | 0 |
| 635655 | 2010 | 7/13/2010 | BARLEY, WHOLE GRAIN | Grains and Grain Products | Barley | IMPORT | Korea (the Republic of) | Non-Detect | 1 | 0 |
| 633946 | 2010 | 7/14/2010 | BARLEY, WHOLE GRAIN | Grains and Grain Products | Barley | DOMESTIC | United States | Non-Detect | 2.6 | 0 |
| 633947 | 2010 | 7/14/2010 | BARLEY, WHOLE GRAIN | Grains and Grain Products | Barley | DOMESTIC | United States | Non-Detect | 2.6 | 0 |
| 633948 | 2010 | 7/14/2010 | BARLEY, WHOLE GRAIN | Grains and Grain Products | Barley | DOMESTIC | United States | Non-Detect | 2.6 | 0 |
| 633945 | 2010 | 7/14/2010 | BARLEY, WHOLE GRAIN | Grains and Grain Products | Barley | DOMESTIC | United States | Positive | 2.6 | 11.67 |
| 624013 | 2010 | 7/15/2010 | WHEAT FLOUR, ENRICHED (ALL PURPOSE) | Grains and Grain Products | Wheat | DOMESTIC | United States | Non-Detect | 2.6 | 0 |
| 636453 | 2010 | 7/19/2010 | RICE FLAKES, PUFFS, KRISPIES, LOOPS READY TO EAT | Processed Food Products | Breakfast Foods | IMPORT | Mexico | Non-Detect | 0.233 | 0 |
| 636694 | 2010 | 7/20/2010 | HORCHATA (BEVERAGE BASE N.E.C.) | Non-Juice Beverages | Other Beverages | IMPORT | El Salvador | Non-Detect | 2.6 | 0 |
| 636729 | 2010 | 7/22/2010 | WHEAT FLOUR, BROMATED | Grains and Grain Products | Wheat | DOMESTIC | United States | Non-Detect | 2.6 | 0 |
| 636545 | 2010 | 7/22/2010 | BARLEY, WHOLE GRAIN | Grains and Grain Products | Barley | DOMESTIC | United States | Non-Detect | 0.233 | 0 |
| 599448 | 2010 | 7/29/2010 | RAISINS, DRIED OR PASTE | Fruit | Raisins | DOMESTIC | United States | Non-Detect | 2.6 | 0 |
| 621808 | 2010 | 8/2/2010 | COFFEE, BEANS | Non-Juice Beverages | Coffee | IMPORT | Colombia | Non-Detect | 2.6 | 0 |
| 578120 | 2010 | 8/4/2010 | COFFEE, BEANS | Non-Juice Beverages | Coffee | IMPORT | Guatemala | Non-Detect | 0.233 | 0 |
| 637955 | 2010 | 8/4/2010 | WHEAT, WHOLE GRAIN | Grains and Grain Products | Wheat | DOMESTIC | United States | Non-Detect | 0.233 | 0 |

| | | | | | | | | | | |
|---|---|---|---|---|---|---|---|---|---|---|
| 630570 | 2010 | 8/4/2010 | CORN, BOLTED MEAL OR FLOUR | Grains and Grain Products | Corn | DOMESTIC | United States | Non-Detect | 1 | 0 |
| 624187 | 2010 | 8/16/2010 | OAT FLAKES, ROLLED OATS, PUFFS, KRISPIES, LOOPS READY TO EAT | Processed Food Products | Breakfast Foods | IMPORT | United States | Non-Detect | 2.6 | 0 |
| 624189 | 2010 | 8/16/2010 | WHEAT, MILLED (CRUSHED, COARSE GROUND OR CRACKED) | Grains and Grain Products | Wheat | IMPORT | United States | Non-Detect | 0.233 | 0 |
| 626048 | 2010 | 8/17/2010 | OAT FLAKES, ROLLED OATS, PUFFS, KRISPIES, LOOPS READY TO EAT | Processed Food Products | Breakfast Foods | DOMESTIC | United States | Non-Detect | 0.233 | 0 |
| 600770 | 2010 | 8/18/2010 | CORN GRITS, BREWERS ENRICHED WHITE OR YELLOW CORN GRITS, CORN MEAL MUSH, QUICK OR INSTANT COOKING | Processed Food Products | Other Corn Products | DOMESTIC | United States | Non-Detect | 2.6 | 0 |
| 643620 | 2010 | 8/26/2010 | GRAM FLOUR | Beans and Legumes | Garbanzo Beans | IMPORT | Canada | Non-Detect | 2.6 | 0 |
| 599788 | 2010 | 8/30/2010 | CORN, BOLTED MEAL OR FLOUR | Grains and Grain Products | Corn | DOMESTIC | United States | Non-Detect | 1 | 0 |
| 599789 | 2010 | 8/30/2010 | CORN, BOLTED MEAL OR FLOUR | Grains and Grain Products | Corn | DOMESTIC | United States | Non-Detect | 1 | 0 |
| 599790 | 2010 | 8/30/2010 | CORN, BOLTED MEAL OR FLOUR | Grains and Grain Products | Corn | DOMESTIC | United States | Non-Detect | 1 | 0 |
| 599791 | 2010 | 8/30/2010 | CORN, BOLTED MEAL OR FLOUR | Grains and Grain Products | Corn | DOMESTIC | United States | Non-Detect | 1 | 0 |
| 599792 | 2010 | 8/30/2010 | CORN, BOLTED MEAL OR FLOUR | Grains and Grain Products | Corn | DOMESTIC | United States | Non-Detect | 1 | 0 |
| 637957 | 2010 | 9/7/2010 | COFFEE, BEANS | Non-Juice Beverages | Coffee | DOMESTIC | United States | Non-Detect | 0.233 | 0 |
| 637684 | 2010 | 9/8/2010 | RAISINS, DRIED OR PASTE | Fruit | Raisins | DOMESTIC | United States | Non-Detect | 2.6 | 0 |
| 643586 | 2010 | 9/8/2010 | OATMEAL, REGULAR, FRUIT OR SPICE ADDED, QUICK OR INSTANT COOKING | Processed Food Products | Breakfast Foods | DOMESTIC | United States | Non-Detect | 2.6 | 0 |
| 643587 | 2010 | 9/9/2010 | RICE CEREAL (BABY) | Baby Food Products | Baby Cereals | DOMESTIC | United States | Non-Detect | 2.6 | 0 |
| 643588 | 2010 | 9/9/2010 | RICE CEREAL (BABY) | Baby Food Products | Baby Cereals | DOMESTIC | United States | Non-Detect | 2.6 | 0 |
| 646268 | 2010 | 9/10/2010 | COFFEE, BEANS | Non-Juice Beverages | Coffee | IMPORT | Colombia | Non-Detect | 0.233 | 0 |
| 607517 | 2010 | 9/21/2010 | CORN, BOLTED MEAL OR FLOUR | Grains and Grain Products | Corn | DOMESTIC | United States | Non-Detect | 2.6 | 0 |
| 648304 | 2010 | 9/22/2010 | WHEAT FLOUR, ENRICHED (ALL PURPOSE) | Grains and Grain Products | Wheat | IMPORT | Canada | Non-Detect | 2.6 | 0 |
| 599125 | 2010 | 9/22/2010 | KIDNEY BEAN, DRIED OR PASTE | Beans and Legumes | Other Beans and Legumes | DOMESTIC | United States | Non-Detect | 1 | 0 |
| 599124 | 2010 | 9/23/2010 | PINTO BEAN, DRIED OR PASTE | Beans and Legumes | Pinto Beans | DOMESTIC | United States | Non-Detect | 1 | 0 |
| 649576 | 2010 | 9/28/2010 | BARLEY, WHOLE GRAIN | Grains and Grain Products | Barley | IMPORT | Ethiopia | Positive | 1 | 1.1 |
| 649706 | 2010 | 9/29/2010 | CORN, DRIED OR PASTE | Grains and Grain Products | Corn | IMPORT | Mexico | Non-Detect | 1 | 0 |
| 649823 | 2010 | 9/30/2010 | SOYBEANS, WHOLE GRAIN | Beans and Legumes | Soybeans | DOMESTIC | United States | Non-Detect | 2.6 | 0 |
| 649825 | 2010 | 9/30/2010 | SOYBEANS, WHOLE GRAIN | Beans and Legumes | Soybeans | DOMESTIC | United States | Non-Detect | 2.6 | 0 |
| 649829 | 2010 | 9/30/2010 | SOYBEANS, WHOLE GRAIN | Beans and Legumes | Soybeans | DOMESTIC | United States | Non-Detect | 2.6 | 0 |
| 634894 | 2010 | 9/30/2010 | SOYBEANS, WHOLE GRAIN | Beans and Legumes | Soybeans | DOMESTIC | United States | Non-Detect | 1 | 0 |
| 649814 | 2010 | 9/30/2010 | SOYBEANS, WHOLE GRAIN | Beans and Legumes | Soybeans | DOMESTIC | United States | Non-Detect | 1 | 0 |
| 649819 | 2010 | 9/30/2010 | SOYBEANS, WHOLE GRAIN | Beans and Legumes | Soybeans | DOMESTIC | United States | Non-Detect | 1 | 0 |
| 649821 | 2010 | 9/30/2010 | SOYBEANS, WHOLE GRAIN | Beans and Legumes | Soybeans | DOMESTIC | United States | Non-Detect | 1 | 0 |
| 649827 | 2010 | 9/30/2010 | SOYBEANS, WHOLE GRAIN | Beans and Legumes | Soybeans | DOMESTIC | United States | Non-Detect | 1 | 0 |

**FY2011 Data**

| Sample Number | Fiscal Year | Collection Date | Product Name | Food Category | Food Subcategory | Origin Type | Country of Origin | Non-Detect, Trace, or Positive (>LOQ) | LOQ (ppb) | Amount found (ppb) |
|---|---|---|---|---|---|---|---|---|---|---|
| 562709 | 2011 | 10/1/2010 | CORN, PLAIN MEAL | Grains and Grain Products | Corn | DOMESTIC | United States | Non-Detect | 1 | 0 |
| 650533 | 2011 | 10/5/2010 | COFFEE, BEANS | Non-Juice Beverages | Coffee | IMPORT | Nicaragua | Non-Detect | 1 | 0 |
| 638280 | 2011 | 10/6/2010 | WHEAT FLOUR, BROMATED | Grains and Grain Products | Wheat | DOMESTIC | United States | Non-Detect | 2.6 | 0 |
| 651059 | 2011 | 10/7/2010 | WHEAT FLOUR, WHOLE | Grains and Grain Products | Wheat | IMPORT | Canada | Non-Detect | 1 | 0 |
| 651425 | 2011 | 10/8/2010 | OATS, WHOLE GRAIN | Grains and Grain Products | Oats | IMPORT | China | Non-Detect | 0.233 | 0 |
| 651428 | 2011 | 10/8/2010 | WHEAT, WHOLE GRAIN | Grains and Grain Products | Wheat | IMPORT | China | Non-Detect | 0.233 | 0 |
| 609231 | 2011 | 10/18/2010 | WHEAT FLOUR, ENRICHED (ALL PURPOSE) | Grains and Grain Products | Wheat | DOMESTIC | United States | Non-Detect | 2.6 | 0 |
| 609232 | 2011 | 10/18/2010 | WHEAT CEREAL (BABY) | Baby Food Products | Baby Cereals | DOMESTIC | United States | Non-Detect | 2.6 | 0 |
| 633752 | 2011 | 10/18/2010 | MALT, BARLEY | Grains and Grain Products | Barley | DOMESTIC | United States | Non-Detect | 2.6 | 0 |
| 649075 | 2011 | 10/18/2010 | CORN GRITS, BREWERS ENRICHED WHITE OR YELLOW CORN GRITS, CORN MEAL MUSH, QUICK OR INSTANT COOKING | Processed Food Products | Other Corn Products | DOMESTIC | United States | Non-Detect | 2.6 | 0 |
| 653299 | 2011 | 10/20/2010 | WHEAT, WHOLE GRAIN | Grains and Grain Products | Wheat | IMPORT | United States | Non-Detect | 1 | 0 |
| 653315 | 2011 | 10/20/2010 | WHEAT, WHOLE GRAIN | Grains and Grain Products | Wheat | IMPORT | United States | Non-Detect | 1 | 0 |
| 653331 | 2011 | 10/20/2010 | WHEAT, WHOLE GRAIN | Grains and Grain Products | Wheat | IMPORT | United States | Trace | 1 | |
| 654292 | 2011 | 10/22/2010 | WHEAT FLOUR, WHOLE DURUM | Grains and Grain Products | Wheat | IMPORT | Canada | Non-Detect | 2.6 | 0 |
| 655258 | 2011 | 10/26/2010 | GARBANZO BEAN | Beans and Legumes | Garbanzo Beans | IMPORT | Mexico | Non-Detect | 1 | 0 |
| 655400 | 2011 | 10/27/2010 | CORN STARCH | Grains and Grain Products | Corn | IMPORT | Korea (the Republic of) | Non-Detect | 1 | 0 |
| 655786 | 2011 | 10/29/2010 | MUNG BEAN, DRIED OR PASTE | Beans and Legumes | Other Beans and Legumes | IMPORT | Thailand | Non-Detect | 0.233 | 0 |
| 656950 | 2011 | 11/2/2010 | CORN GRITS, BREWERS ENRICHED WHITE OR YELLOW CORN GRITS, CORN MEAL MUSH, QUICK OR INSTANT COOKING | Processed Food Products | Other Corn Products | IMPORT | Brazil | Non-Detect | 1 | 0 |
| 659319 | 2011 | 11/3/2010 | BARLEY, WHOLE GRAIN | Grains and Grain Products | Barley | IMPORT | Russia | Non-Detect | 2.6 | 0 |
| 656685 | 2011 | 11/3/2010 | CORN, WHOLE GRAIN | Grains and Grain Products | Corn | IMPORT | Mexico | Non-Detect | 1 | 0 |
| 658214 | 2011 | 11/6/2010 | WHEAT FLAKES, PUFFS, KRISPIES, LOOPS, SHREDDED, WHEAT GERM READY TO EAT | Processed Food Products | Breakfast Foods | IMPORT | Canada | Non-Detect | 1 | 0 |
| 636717 | 2011 | 11/8/2010 | COFFEE, GROUND, DECAFFEINATED | Non-Juice Beverages | Coffee | DOMESTIC | United States | Non-Detect | 2.6 | 0 |
| 658054 | 2011 | 11/9/2010 | QUINOA, WHOLE GRAIN | Grains and Grain Products | Other Grains | IMPORT | Bolivia | Non-Detect | 1 | 0 |
| 658427 | 2011 | 11/10/2010 | PINOLE (CORN BEVERAGE BASE) | Non-Juice Beverages | Other Beverages | IMPORT | Mexico | Non-Detect | 1 | 0 |
| 659755 | 2011 | 11/17/2010 | BARLEY, WHOLE GRAIN | Grains and Grain Products | Barley | IMPORT | Peru | Non-Detect | 2.6 | 0 |
| 659732 | 2011 | 11/17/2010 | WHEAT FLAKES, PUFFS, KRISPIES, LOOPS, SHREDDED, WHEAT GERM READY TO EAT | Processed Food Products | Breakfast Foods | IMPORT | Germany | Non-Detect | 1 | 0 |
| 638282 | 2011 | 11/18/2010 | WHEAT FLOUR, BROMATED | Grains and Grain Products | Wheat | DOMESTIC | United States | Non-Detect | 1 | 0 |
| 661000 | 2011 | 11/22/2010 | ORIENTAL NOODLES (FLAVORED WITH SHRIMP, CHICKEN, BEEF, LOBSTER, CRAB, PLAIN, ETC.) | Processed Food Products | Pasta | IMPORT | Philippines | Non-Detect | 1 | 0 |
| 661261 | 2011 | 11/23/2010 | BARLEY, WHOLE GRAIN | Grains and Grain Products | Barley | IMPORT | Canada | Non-Detect | 2.6 | 0 |
| 660997 | 2011 | 11/23/2010 | CORN, BOLTED MEAL OR FLOUR | Grains and Grain Products | Corn | IMPORT | Guatemala | Trace | 1 | |
| 661410 | 2011 | 11/24/2010 | KIDNEY BEAN | Beans and Legumes | Other Beans and Legumes | IMPORT | Canada | Non-Detect | 2.6 | 0 |
| 661443 | 2011 | 11/26/2010 | GRAM FLOUR | Beans and Legumes | Garbanzo Beans | IMPORT | Canada | Non-Detect | 2.6 | 0 |
| 663778 | 2011 | 12/7/2010 | BAKED SNACK FOOD, N.E.C. | Processed Food Products | Other Snack Foods | IMPORT | India | Non-Detect | 0.233 | 0 |
| 663653 | 2011 | 12/8/2010 | WHEAT FLOUR, WHOLE | Grains and Grain Products | Wheat | IMPORT | United Arab Emirates | Non-Detect | 1 | 0 |
| 664824 | 2011 | 12/15/2010 | BARLEY, WHOLE GRAIN | Grains and Grain Products | Barley | IMPORT | Peru | Non-Detect | 1 | 0 |
| 563956 | 2011 | 12/21/2010 | WHEAT FLOUR, BROMATED | Grains and Grain Products | Wheat | DOMESTIC | United States | Non-Detect | 2.9 | 0 |
| 666509 | 2011 | 12/21/2010 | BARLEY, WHOLE GRAIN | Grains and Grain Products | Barley | IMPORT | Korea (the Republic of) | Non-Detect | 1 | 0 |
| 666343 | 2011 | 12/22/2010 | CORN CHIPS, FRIED | Processed Food Products | Other Corn Products | IMPORT | Mexico | Non-Detect | 1 | 0 |
| 668017 | 2011 | 1/4/2011 | CORN, PLAIN MEAL | Grains and Grain Products | Corn | IMPORT | Mexico | Trace | 1 | |
| 668225 | 2011 | 1/5/2011 | BISCUITS | Processed Food Products | Other Bakery Products | IMPORT | India | Trace | 1 | |
| 659599 | 2011 | 1/6/2011 | BRAN FLAKES, SHREDDED AND OTHER FORMS READY TO EAT | Processed Food Products | Breakfast Foods | DOMESTIC | United States | Non-Detect | 2.6 | 0 |
| 660979 | 2011 | 1/7/2011 | WHEAT FLOUR, BROMATED | Grains and Grain Products | Wheat | DOMESTIC | United States | Trace | 1 | |
| 669353 | 2011 | 1/11/2011 | LIMA BEAN | Beans and Legumes | Other Beans and Legumes | IMPORT | Mexico | Non-Detect | 1 | 0 |
| 558135 | 2011 | 1/12/2011 | CORN, BOLTED MEAL OR FLOUR | Grains and Grain Products | Corn | DOMESTIC | United States | Non-Detect | 1 | 0 |
| 629785 | 2011 | 1/13/2011 | BEAN, DRIED OR PASTE, N.E.C. (VEGETABLE) | Beans and Legumes | Other Beans and Legumes | DOMESTIC | United States | Non-Detect | 2.6 | 0 |
| 652461 | 2011 | 1/18/2011 | RAISINS, DRIED OR PASTE | Fruit | Raisins | DOMESTIC | United States | Non-Detect | 2.6 | 0 |
| 670402 | 2011 | 1/19/2011 | COFFEE, BEANS | Non-Juice Beverages | Coffee | IMPORT | Colombia | Non-Detect | 0.233 | 0 |
| 672092 | 2011 | 1/26/2011 | JALAPENO PEPPER, HOT (FRUIT USED AS VEGETABLE | Vegetables | Vegetables | IMPORT | Mexico | Non-Detect | 0.233 | 0 |
| 636189 | 2011 | 1/27/2011 | FLOURS AND MEALS N.E.C. | Grains and Grain Products | Other Grains | DOMESTIC | United States | Non-Detect | 0.233 | 0 |
| 672815 | 2011 | 1/27/2011 | CUSTARD PUDDING (PIE) MIX, N.E.C. | Other | Other | IMPORT | Pakistan | Non-Detect | 1 | 0 |
| 637784 | 2011 | 2/2/2011 | SOYBEAN FLOUR | Beans and Legumes | Soybeans | DOMESTIC | United States | Non-Detect | 0.233 | 0 |
| 673847 | 2011 | 2/4/2011 | OATMEAL, REGULAR, FRUIT OR SPICE ADDED, QUICK OR INSTANT COOKING | Processed Food Products | Breakfast Foods | IMPORT | Dominican Republic (the) | Non-Detect | 2.6 | 0 |
| 674545 | 2011 | 2/8/2011 | CORN, PLAIN MEAL | Grains and Grain Products | Corn | IMPORT | United States | Non-Detect | 1 | 0 |
| 674764 | 2011 | 2/9/2011 | OATMEAL, REGULAR, FRUIT OR SPICE ADDED, QUICK OR INSTANT COOKING | Processed Food Products | Breakfast Foods | IMPORT | Canada | Non-Detect | 2.6 | 0 |
| 674792 | 2011 | 2/9/2011 | CORN, MILLED PRODUCT N.E.C. | Grains and Grain Products | Corn | IMPORT | Italy | Non-Detect | 1 | 0 |
| 674927 | 2011 | 2/9/2011 | CORN, BOLTED MEAL OR FLOUR | Grains and Grain Products | Corn | IMPORT | Nicaragua | Non-Detect | 1 | 0 |
| 674928 | 2011 | 2/9/2011 | CORN, BOLTED MEAL OR FLOUR | Grains and Grain Products | Corn | IMPORT | Colombia | Non-Detect | 1 | 0 |
| 675237 | 2011 | 2/10/2011 | OATMEAL, REGULAR, FRUIT OR SPICE ADDED, QUICK OR INSTANT COOKING | Processed Food Products | Breakfast Foods | IMPORT | Finland | Non-Detect | 1 | 0 |
| 677253 | 2011 | 2/16/2011 | BUCKWHEAT FLOUR | Grains and Grain Products | Buckwheat | IMPORT | Poland | Positive | 20 | 50.1 |
| 676724 | 2011 | 2/17/2011 | RAISINS (DRIED GRAPES) (BERRY) | Fruit | Raisins | IMPORT | China | Non-Detect | 2.6 | 0 |
| 676762 | 2011 | 2/17/2011 | KIDNEY BEAN | Beans and Legumes | Other Beans and Legumes | IMPORT | Honduras | Non-Detect | 2.6 | 0 |
| 643625 | 2011 | 2/17/2011 | WHEAT FLOUR, WHOLE | Grains and Grain Products | Wheat | DOMESTIC | United States | Non-Detect | 1 | 0 |
| 677417 | 2011 | 2/22/2011 | MALT, BARLEY | Grains and Grain Products | Barley | IMPORT | Canada | Non-Detect | 1 | 0 |
| 677942 | 2011 | 2/24/2011 | CORN, BOLTED MEAL OR FLOUR | Grains and Grain Products | Corn | IMPORT | Canada | Non-Detect | 1 | 0 |
| 533771 | 2011 | 2/28/2011 | WHEAT FLOUR, ENRICHED BROMATED | Grains and Grain Products | Wheat | DOMESTIC | United States | Non-Detect | 2.6 | 0 |
| 678956 | 2011 | 3/1/2011 | ORIENTAL NOODLES (FLAVORED WITH SHRIMP, CHICKEN, BEEF, LOBSTER, CRAB, PLAIN, ETC.) | Processed Food Products | Pasta | IMPORT | Korea (the Republic of) | Non-Detect | 1 | 0 |
| 679052 | 2011 | 3/1/2011 | GARBANZO BEAN | Beans and Legumes | Garbanzo Beans | IMPORT | India | Non-Detect | 1 | 0 |
| 679422 | 2011 | 3/3/2011 | MALT, BARLEY | Grains and Grain Products | Barley | IMPORT | Belgium | Non-Detect | 2.6 | 0 |
| 679442 | 2011 | 3/3/2011 | MALT, BARLEY | Grains and Grain Products | Barley | IMPORT | Belgium | Non-Detect | 2.6 | 0 |
| 679629 | 2011 | 3/4/2011 | MALT, BARLEY | Grains and Grain Products | Barley | IMPORT | United Kingdom | Non-Detect | 2.6 | 0 |
| 679711 | 2011 | 3/7/2011 | OATS, WHOLE GRAIN | Grains and Grain Products | Oats | IMPORT | Canada | Non-Detect | 1 | 0 |
| 670166 | 2011 | 3/10/2011 | BARLEY, WHOLE GRAIN | Grains and Grain Products | Barley | DOMESTIC | United States | Non-Detect | 2.6 | 0 |
| 680917 | 2011 | 3/11/2011 | OATMEAL, REGULAR, FRUIT OR SPICE ADDED, QUICK OR INSTANT COOKING | Processed Food Products | Breakfast Foods | IMPORT | Dominican Republic (the) | Non-Detect | 2.6 | 0 |
| 681168 | 2011 | 3/14/2011 | COFFEE, BEANS | Non-Juice Beverages | Coffee | IMPORT | Peru | Non-Detect | 1 | 0 |
| 681817 | 2011 | 3/17/2011 | BLACK EYE BEANS | Beans and Legumes | Blackeye Peas | IMPORT | China | Non-Detect | 0.233 | 0 |
| 682058 | 2011 | 3/17/2011 | OAT FLAKES, ROLLED OATS, PUFFS, KRISPIES, LOOPS READY TO EAT | Processed Food Products | Breakfast Foods | IMPORT | Mexico | Non-Detect | 0.233 | 0 |
| 682348 | 2011 | 3/21/2011 | GRAM FLOUR | Beans and Legumes | Garbanzo Beans | IMPORT | Canada | Trace | 1 | |
| 682624 | 2011 | 3/22/2011 | OAT FLAKES, ROLLED OATS, PUFFS, KRISPIES, LOOPS READY TO EAT | Processed Food Products | Breakfast Foods | IMPORT | Canada | Non-Detect | 1 | 0 |
| 682730 | 2011 | 3/22/2011 | CORN CHIPS, FRIED | Processed Food Products | Other Corn Products | IMPORT | Philippines | Non-Detect | 1 | 0 |
| 683551 | 2011 | 3/25/2011 | HEMP SEED (EDIBLE SEED) | Nuts and Edible Seeds | Other Nuts and Edible Seeds | IMPORT | Romania | Non-Detect | 2.6 | 0 |
| 684582 | 2011 | 4/4/2011 | COFFEE, BEANS | Non-Juice Beverages | Coffee | IMPORT | Brazil | Non-Detect | 2.6 | 0 |
| 684855 | 2011 | 4/5/2011 | QUINOA SEED (EDIBLE SEED) | Nuts and Edible Seeds | Other Nuts and Edible Seeds | IMPORT | Peru | Non-Detect | 0.233 | 0 |
| 685052 | 2011 | 4/5/2011 | BARLEY, WHOLE GRAIN | Grains and Grain Products | Barley | IMPORT | Peru | Non-Detect | 0.233 | 0 |
| 653673 | 2011 | 4/7/2011 | SOYBEANS, WHOLE GRAIN | Beans and Legumes | Soybeans | DOMESTIC | United States | Non-Detect | 2.6 | 0 |
| 671309 | 2011 | 4/12/2011 | CORN GRITS, BREWERS ENRICHED WHITE OR YELLOW CORN GRITS, CORN MEAL MUSH, QUICK OR INSTANT COOKING | Processed Food Products | Other Corn Products | DOMESTIC | United States | Non-Detect | 1 | 0 |
| 623405 | 2011 | 4/14/2011 | BARLEY, WHOLE GRAIN | Grains and Grain Products | Barley | DOMESTIC | Unknown | Non-Detect | 2.6 | 0 |
| 623407 | 2011 | 4/14/2011 | BARLEY, WHOLE GRAIN | Grains and Grain Products | Barley | DOMESTIC | Unknown | Non-Detect | 2.6 | 0 |
| 623409 | 2011 | 4/14/2011 | MALT, BARLEY | Grains and Grain Products | Barley | DOMESTIC | Unknown | Non-Detect | 2.6 | 0 |

| | | | | | | | | | | |
|---|---|---|---|---|---|---|---|---|---|---|
| 623411 | 2011 | 4/14/2011 | MALT, BARLEY | Grains and Grain Products | Barley | DOMESTIC | Unknown | Non-Detect | 2.6 | 0 |
| 645240 | 2011 | 4/14/2011 | RICE FLOUR | Grains and Grain Products | Rice | DOMESTIC | United States | Non-Detect | 2.6 | 0 |
| 645241 | 2011 | 4/14/2011 | RICE GLUTEN | Grains and Grain Products | Rice | DOMESTIC | United States | Non-Detect | 2.6 | 0 |
| 661541 | 2011 | 4/18/2011 | BARLEY FLOUR | Grains and Grain Products | Barley | DOMESTIC | United States | Non-Detect | 2.6 | 0 |
| 661543 | 2011 | 4/18/2011 | WHEAT, WHOLE GRAIN | Grains and Grain Products | Wheat | DOMESTIC | United States | Non-Detect | 2.6 | 0 |
| 661546 | 2011 | 4/18/2011 | WHEAT, WHOLE GRAIN | Grains and Grain Products | Wheat | DOMESTIC | United States | Non-Detect | 2.6 | 0 |
| 661547 | 2011 | 4/20/2011 | WHEAT, WHOLE GRAIN | Grains and Grain Products | Wheat | DOMESTIC | United States | Non-Detect | 2.6 | 0 |
| 661548 | 2011 | 4/20/2011 | WHEAT FLOUR, ENRICHED (ALL PURPOSE) | Grains and Grain Products | Wheat | DOMESTIC | United States | Non-Detect | 2.6 | 0 |
| 661549 | 2011 | 4/20/2011 | WHEAT GERM | Grains and Grain Products | Wheat | DOMESTIC | United States | Non-Detect | 2.6 | 0 |
| 663226 | 2011 | 4/20/2011 | WHEAT, WHOLE GRAIN | Grains and Grain Products | Wheat | DOMESTIC | United States | Non-Detect | 2.6 | 0 |
| 688767 | 2011 | 4/26/2011 | COFFEE, BEANS | Non-Juice Beverages | Coffee | IMPORT | Honduras | Non-Detect | 2.6 | 0 |
| 678567 | 2011 | 4/26/2011 | WHEAT FLOUR, ENRICHED (ALL PURPOSE) | Grains and Grain Products | Wheat | DOMESTIC | United States | Non-Detect | 1 | 0 |
| 688756 | 2011 | 4/26/2011 | FLAVORED OR PARTY CRACKERS | Processed Food Products | Other Bakery Products | IMPORT | Philippines | Non-Detect | 1 | 0 |
| 688966 | 2011 | 4/27/2011 | COFFEE, BEANS | Non-Juice Beverages | Coffee | IMPORT | Brazil | Non-Detect | 2.6 | 0 |
| 690033 | 2011 | 5/2/2011 | WHEAT FLOUR, WHOLE | Grains and Grain Products | Wheat | IMPORT | India | Non-Detect | 1 | 0 |
| 690041 | 2011 | 5/2/2011 | WHEAT FLOUR, WHOLE | Grains and Grain Products | Wheat | IMPORT | India | Non-Detect | 1 | 0 |
| 689086 | 2011 | 5/3/2011 | RICE FLAKES, PUFFS, KRISPIES, LOOPS READY TO EAT | Processed Food Products | Breakfast Foods | DOMESTIC | United States | Non-Detect | 2.6 | 0 |
| 689087 | 2011 | 5/3/2011 | WHEAT BRAN (HUMAN USE) | Grains and Grain Products | Wheat | DOMESTIC | United States | Non-Detect | 1 | 0 |
| 690515 | 2011 | 5/3/2011 | CORN, BOLTED MEAL OR FLOUR | Grains and Grain Products | Corn | IMPORT | Argentina | Non-Detect | 1 | 0 |
| 555504 | 2011 | 5/5/2011 | WHEAT FLOUR, WHOLE | Grains and Grain Products | Wheat | DOMESTIC | United States | Non-Detect | 1 | 0 |
| 690898 | 2011 | 5/5/2011 | RICE BEAN, DRIED OR PASTE | Beans and Legumes | Other Beans and Legumes | IMPORT | China | Non-Detect | 1 | 0 |
| 637488 | 2011 | 5/10/2011 | COFFEE, BEANS | Non-Juice Beverages | Coffee | IMPORT | Colombia | Non-Detect | 2.6 | 0 |
| 692310 | 2011 | 5/11/2011 | NAVY (WHITE) BEANS (BAKED BEANS, PORK AND BEANS), DRIED OR PASTE | Beans and Legumes | Other Beans and Legumes | IMPORT | Lebanon | Non-Detect | 1 | 0 |
| 692247 | 2011 | 5/12/2011 | WHEAT FLAKES, PUFFS, KRISPIES, LOOPS, SHREDDED, WHEAT GERM READY TO EAT | Processed Food Products | Breakfast Foods | IMPORT | Mexico | Non-Detect | 0.233 | 0 |
| 693304 | 2011 | 5/18/2011 | OATS, WHOLE GRAIN | Grains and Grain Products | Oats | IMPORT | Canada | Non-Detect | 0.233 | 0 |
| 674105 | 2011 | 5/24/2011 | MIXED GRAIN CEREAL (BABY) | Baby Food Products | Baby Cereals | DOMESTIC | United States | Non-Detect | 2.6 | 0 |
| 672910 | 2011 | 5/25/2011 | CORN, ENRICHED MEAL | Grains and Grain Products | Corn | DOMESTIC | United States | Non-Detect | 1 | 0 |
| 573849 | 2011 | 6/1/2011 | WHEAT FLOUR, BROMATED | Grains and Grain Products | Wheat | IMPORT | Canada | Non-Detect | 2.6 | 0 |
| 653509 | 2011 | 6/2/2011 | BARLEY, WHOLE GRAIN | Grains and Grain Products | Barley | DOMESTIC | United States | Non-Detect | 1 | 0 |
| 653510 | 2011 | 6/2/2011 | CORN, ENRICHED MEAL | Grains and Grain Products | Corn | DOMESTIC | United States | Non-Detect | 1 | 0 |
| 696777 | 2011 | 6/3/2011 | OAT FLAKES, ROLLED OATS, PUFFS, KRISPIES, LOOPS READY TO EAT | Processed Food Products | Breakfast Foods | IMPORT | Mexico | Non-Detect | 0.233 | 0 |
| 696472 | 2011 | 6/7/2011 | WHEAT FLAKES, PUFFS, KRISPIES, LOOPS, SHREDDED, WHEAT GERM READY TO EAT | Processed Food Products | Breakfast Foods | IMPORT | Canada | Non-Detect | 2.6 | 0 |
| 693269 | 2011 | 6/8/2011 | SOYBEANS, WHOLE GRAIN | Beans and Legumes | Soybeans | DOMESTIC | United States | Non-Detect | 2.6 | 0 |
| 693270 | 2011 | 6/8/2011 | SOYBEANS, WHOLE GRAIN | Beans and Legumes | Soybeans | DOMESTIC | United States | Non-Detect | 2.6 | 0 |
| 693274 | 2011 | 6/8/2011 | SOYBEANS, WHOLE GRAIN | Beans and Legumes | Soybeans | DOMESTIC | United States | Non-Detect | 2.6 | 0 |
| 671300 | 2011 | 6/8/2011 | CORN GRITS, BREWERS ENRICHED WHITE OR YELLOW CORN GRITS,CORN MEAL MUSH, QUICK OR INSTANT COOKING | Processed Food Products | Other Corn Products | DOMESTIC | Unknown | Non-Detect | 1 | 0 |
| 693276 | 2011 | 6/8/2011 | SOYBEANS, WHOLE GRAIN | Beans and Legumes | Soybeans | DOMESTIC | United States | Trace | 2.6 | |
| 696776 | 2011 | 6/8/2011 | CORN, DRIED OR PASTE | Grains and Grain Products | Corn | IMPORT | Peru | Trace | 1 | |
| 696919 | 2011 | 6/8/2011 | WHEAT FLOUR, WHOLE DURUM | Grains and Grain Products | Wheat | IMPORT | Canada | Trace | 1 | |
| 697684 | 2011 | 6/13/2011 | QUINOA SEED (EDIBLE SEED) | Nuts and Edible Seeds | Other Nuts and Edible Seeds | IMPORT | Bolivia | Non-Detect | 2.6 | 0 |
| 698302 | 2011 | 6/15/2011 | OAT FLAKES, ROLLED OATS, PUFFS, KRISPIES, LOOPS READY TO EAT | Processed Food Products | Breakfast Foods | IMPORT | Canada | Non-Detect | 2.6 | 0 |
| 652466 | 2011 | 6/21/2011 | WHEAT FLOUR, BROMATED | Grains and Grain Products | Wheat | DOMESTIC | United States | Non-Detect | 2.6 | 0 |
| 699053 | 2011 | 6/21/2011 | RAISINS, DRIED OR PASTE | Fruit | Raisins | IMPORT | Chile | Non-Detect | 2.6 | 0 |
| 699085 | 2011 | 6/21/2011 | CORN FLAKES, PUFFS, KRISPIES, LOOPS READY TO EAT | Processed Food Products | Other Corn Products | IMPORT | United States | Non-Detect | 2.6 | 0 |
| 699075 | 2011 | 6/21/2011 | RAISINS (DRIED GRAPES) (BERRY) | Fruit | Raisins | IMPORT | Argentina | Trace | 5 | |
| 692236 | 2011 | 6/22/2011 | WHEAT FLOUR, ENRICHED (ALL PURPOSE) | Grains and Grain Products | Wheat | DOMESTIC | United States | Non-Detect | 2.6 | 0 |
| 699849 | 2011 | 6/23/2011 | MAYOCOBA BEANS | Beans and Legumes | Other Beans and Legumes | IMPORT | Mexico | Non-Detect | 0.233 | 0 |
| 572743 | 2011 | 6/27/2011 | COFFEE, BEANS | Non-Juice Beverages | Coffee | DOMESTIC | United States | Non-Detect | 2.6 | 0 |
| 703497 | 2011 | 7/5/2011 | MUNG BEAN | Beans and Legumes | Other Beans and Legumes | IMPORT | Canada | Non-Detect | 2.6 | 0 |
| 406275 | 2011 | 7/6/2011 | WHEAT FLOUR, WHOLE | Grains and Grain Products | Wheat | DOMESTIC | United States | Non-Detect | 0.233 | 0 |
| 682300 | 2011 | 7/11/2011 | BREADING, BREADING MIXES. ETC. | Processed Food Products | Bread | DOMESTIC | United States | Non-Detect | 2.6 | 0 |
| 654346 | 2011 | 7/13/2011 | CORN, BOLTED MEAL OR FLOUR | Grains and Grain Products | Corn | DOMESTIC | United States | Non-Detect | 1 | 0 |
| 704182 | 2011 | 7/14/2011 | WHEAT FLAKES, PUFFS, KRISPIES, LOOPS, SHREDDED, WHEAT GERM READY TO EAT | Processed Food Products | Breakfast Foods | IMPORT | Mexico | Non-Detect | 0.233 | 0 |
| 706653 | 2011 | 7/26/2011 | COFFEE, BEANS | Non-Juice Beverages | Coffee | IMPORT | Kenya | Trace | 1 | |
| 706911 | 2011 | 7/27/2011 | COFFEE, BEANS | Non-Juice Beverages | Coffee | IMPORT | Nicaragua | Non-Detect | 1 | 0 |
| 706883 | 2011 | 7/27/2011 | CORN, BOLTED MEAL OR FLOUR | Grains and Grain Products | Corn | IMPORT | Guatemala | Trace | 1 | |
| 707245 | 2011 | 7/28/2011 | OATS, WHOLE GRAIN | Grains and Grain Products | Oats | IMPORT | Ireland | Non-Detect | 2.6 | 0 |
| 670909 | 2011 | 8/2/2011 | WHEAT, WHOLE GRAIN | Grains and Grain Products | Wheat | DOMESTIC | United States | Non-Detect | 0.233 | 0 |
| 544064 | 2011 | 8/9/2011 | CORN, BOLTED MEAL OR FLOUR | Grains and Grain Products | Corn | DOMESTIC | United States | Non-Detect | 1 | 0 |
| 709982 | 2011 | 8/9/2011 | CORN, BOLTED MEAL OR FLOUR | Grains and Grain Products | Corn | IMPORT | Mexico | Non-Detect | 1 | 0 |
| 710447 | 2011 | 8/11/2011 | BARLEY GRAIN SNACK (WHOLE, SPLIT), BAKED | Processed Food Products | Other Snack Foods | IMPORT | Korea (the Republic of) | Non-Detect | 1 | 0 |
| 710756 | 2011 | 8/15/2011 | FARINA, WHEAT | Grains and Grain Products | Wheat | IMPORT | India | Non-Detect | 1 | 0 |
| 712381 | 2011 | 8/17/2011 | LENTILS, DRIED OR PASTE | Beans and Legumes | Lentils | IMPORT | Spain | Non-Detect | 1 | 0 |
| 712400 | 2011 | 8/17/2011 | GARBANZO BEAN, DRIED OR PASTE | Beans and Legumes | Garbanzo Beans | IMPORT | Spain | Non-Detect | 1 | 0 |
| 712408 | 2011 | 8/17/2011 | KIDNEY BEAN, DRIED OR PASTE | Beans and Legumes | Other Beans and Legumes | IMPORT | Spain | Non-Detect | 1 | 0 |
| 657717 | 2011 | 8/22/2011 | WHEAT FLOUR, BROMATED | Grains and Grain Products | Wheat | DOMESTIC | United States | Non-Detect | 1 | 0 |
| 582936 | 2011 | 8/31/2011 | WHEAT FLOUR, ENRICHED (ALL PURPOSE) | Grains and Grain Products | Wheat | DOMESTIC | United States | Non-Detect | 2.6 | 0 |
| 680177 | 2011 | 8/31/2011 | BARLEY CEREAL (BABY) | Baby Food Products | Baby Cereals | DOMESTIC | United States | Non-Detect | 2.6 | 0 |
| 531805 | 2011 | 8/31/2011 | RYE FLOUR | Grains and Grain Products | Rye | DOMESTIC | United States | Non-Detect | 0.233 | 0 |
| 714129 | 2011 | 9/1/2011 | CORN FLAKES, PUFFS, KRISPIES, LOOPS READY TO EAT | Processed Food Products | Other Corn Products | IMPORT | Mexico | Non-Detect | 0.233 | 0 |
| 714239 | 2011 | 9/2/2011 | CORN FLAKES, PUFFS, KRISPIES, LOOPS READY TO EAT | Processed Food Products | Other Corn Products | IMPORT | Mexico | Non-Detect | 0.233 | 0 |
| 714246 | 2011 | 9/2/2011 | MIXED CEREAL FLAKES, SHREDDED AND OTHER FORMS READY TO EAT | Processed Food Products | Breakfast Foods | IMPORT | Mexico | Non-Detect | 0.233 | 0 |
| 713514 | 2011 | 9/7/2011 | WHEAT FLOUR, ENRICHED (ALL PURPOSE) | Grains and Grain Products | Wheat | DOMESTIC | United States | Non-Detect | 2.6 | 0 |
| 712513 | 2011 | 9/8/2011 | OATS, WHOLE GRAIN | Grains and Grain Products | Oats | DOMESTIC | United States | Non-Detect | 2.6 | 0 |
| 714661 | 2011 | 9/9/2011 | WHEAT FLOUR, ENRICHED (ALL PURPOSE) | Grains and Grain Products | Wheat | DOMESTIC | United States | Non-Detect | 0.233 | 0 |
| 714663 | 2011 | 9/9/2011 | WHEAT FLOUR, ENRICHED (ALL PURPOSE) | Grains and Grain Products | Wheat | DOMESTIC | United States | Non-Detect | 0.233 | 0 |
| 714665 | 2011 | 9/9/2011 | WHEAT FLOUR, ENRICHED (ALL PURPOSE) | Grains and Grain Products | Wheat | DOMESTIC | United States | Trace | 5 | |
| 587465 | 2011 | 9/12/2011 | CORN, ENRICHED MEAL | Grains and Grain Products | Corn | DOMESTIC | United States | Non-Detect | 0.233 | 0 |
| 715349 | 2011 | 9/12/2011 | OATS, WHOLE GRAIN | Grains and Grain Products | Oats | IMPORT | Russia | Non-Detect | 1 | 0 |
| 624657 | 2011 | 9/14/2011 | BARLEY, WHOLE GRAIN | Grains and Grain Products | Barley | DOMESTIC | United States | Non-Detect | 2.6 | 0 |
| 682522 | 2011 | 9/15/2011 | BARLEY, WHOLE GRAIN | Grains and Grain Products | Barley | DOMESTIC | Unknown | Non-Detect | 2.6 | 0 |
| 710897 | 2011 | 9/15/2011 | WHEAT FLOUR, BROMATED | Grains and Grain Products | Wheat | DOMESTIC | United States | Trace | 1 | |
| 716155 | 2011 | 9/16/2011 | BARLEY FLOUR | Grains and Grain Products | Barley | IMPORT | El Salvador | Non-Detect | 2.6 | 0 |
| 700709 | 2011 | 9/20/2011 | WHEAT FLOUR, ENRICHED (ALL PURPOSE) | Grains and Grain Products | Wheat | DOMESTIC | United States | Non-Detect | 0.233 | 0 |
| 691811 | 2011 | 9/22/2011 | PINTO BEAN, DRIED OR PASTE | Beans and Legumes | Pinto Beans | DOMESTIC | United States | Non-Detect | 0.233 | 0 |
| 716974 | 2011 | 9/22/2011 | BLACK EYE BEANS | Beans and Legumes | Blackeye Peas | IMPORT | China | Non-Detect | 1 | 0 |
| 670629 | 2011 | 9/23/2011 | CORN, ENRICHED MEAL | Grains and Grain Products | Corn | DOMESTIC | United States | Non-Detect | 1 | 0 |
| 670630 | 2011 | 9/23/2011 | CORN, ENRICHED MEAL | Grains and Grain Products | Corn | DOMESTIC | United States | Non-Detect | 1 | 0 |

## FY2012 Data

| Sample Number | Fiscal Year | Collection Date | Product Name | Food Category | Food Subcategory | Origin Type | Country of Origin | Non-Detect, Trace, or Positive (>LOQ) | LOQ (ppb) | Amount found (ppb) |
|---|---|---|---|---|---|---|---|---|---|---|
| 718808 | 2012 | 10/3/2011 | COUSCOUS | Processed Food Products | Pasta | IMPORT | Israel | Non-Detect | 2.6 | 0 |
| 718399 | 2012 | 10/4/2011 | KIDNEY BEAN | Beans and Legumes | Other Beans and Legumes | IMPORT | Honduras | Non-Detect | 2.4 | 0 |
| 718452 | 2012 | 10/4/2011 | OAT FLAKES, ROLLED OATS, PUFFS, KRISPIES, LOOPS READY TO EAT | Processed Food Products | Breakfast Foods | IMPORT | Guatemala | Non-Detect | 2.6 | 0 |
| 718463 | 2012 | 10/4/2011 | PANCAKE PREPARED DRY MIX WITHOUT CUSTARD OR CREAM FILLING | Processed Food Products | Other Bakery Products | IMPORT | Mexico | Non-Detect | 0.233 | 0 |
| 718639 | 2012 | 10/5/2011 | COFFEE, BEANS, DECAFFEINATED | Non-Juice Beverages | Coffee | IMPORT | Costa Rica | Non-Detect | 2.6 | 0 |
| 718745 | 2012 | 10/5/2011 | COFFEE, BEANS | Non-Juice Beverages | Coffee | IMPORT | Guatemala | Non-Detect | 2.6 | 0 |
| 718711 | 2012 | 10/5/2011 | BEANS, CORN, AND PEA, N.E.C. (VEGETABLE) | Beans and Legumes | Other Beans and Legumes | IMPORT | Mexico | Non-Detect | 0.233 | 0 |
| 718920 | 2012 | 10/6/2011 | KIDNEY BEAN | Beans and Legumes | Other Beans and Legumes | IMPORT | Honduras | Non-Detect | 2.6 | 0 |
| 718976 | 2012 | 10/6/2011 | COFFEE, BEANS | Non-Juice Beverages | Coffee | IMPORT | Haiti | Non-Detect | 2.6 | 0 |
| 719573 | 2012 | 10/6/2011 | CORN, PLAIN MEAL | Grains and Grain Products | Corn | IMPORT | United States | Non-Detect | 1 | 0 |
| 719594 | 2012 | 10/6/2011 | CORN, PLAIN MEAL | Grains and Grain Products | Corn | IMPORT | Canada | Non-Detect | 1 | 0 |
| 719144 | 2012 | 10/7/2011 | KIDNEY BEAN | Beans and Legumes | Other Beans and Legumes | IMPORT | Nicaragua | Non-Detect | 2.6 | 0 |
| 719266 | 2012 | 10/10/2011 | RICE FLAKES, PUFFS, KRISPIES, LOOPS READY TO EAT | Processed Food Products | Breakfast Foods | IMPORT | Mexico | Non-Detect | 1 | 0 |
| 719391 | 2012 | 10/11/2011 | BLACK EYE BEANS | Beans and Legumes | Blackeye Peas | IMPORT | China | Non-Detect | 1 | 0 |
| 719960 | 2012 | 10/12/2011 | TAMALE (MULTIPLE FOOD SPECIALITIES, SIDE DISHES AND DESSERTS) | Processed Food Products | Other Processed Foods | IMPORT | Mexico | Non-Detect | 0.233 | 0 |
| 719701 | 2012 | 10/12/2011 | COFFEE, BEANS | Non-Juice Beverages | Coffee | IMPORT | Brazil | Non-Detect | 1 | 0 |
| 719734 | 2012 | 10/12/2011 | CORN CHIPS, FRIED | Processed Food Products | Other Corn Products | IMPORT | Mexico | Non-Detect | 1 | 0 |
| 719868 | 2012 | 10/12/2011 | CORN, DRIED OR PASTE | Grains and Grain Products | Corn | IMPORT | Peru | Non-Detect | 1 | 0 |
| 719870 | 2012 | 10/12/2011 | CORN, DRIED OR PASTE | Grains and Grain Products | Corn | IMPORT | Peru | Non-Detect | 1 | 0 |
| 719876 | 2012 | 10/12/2011 | CORN, DRIED OR PASTE | Grains and Grain Products | Corn | IMPORT | Peru | Non-Detect | 1 | 0 |
| 720355 | 2012 | 10/14/2011 | FAVA BEAN | Beans and Legumes | Other Beans and Legumes | IMPORT | Taiwan | Non-Detect | 2.6 | 0 |
| 720520 | 2012 | 10/17/2011 | OAT FLAKES, ROLLED OATS, PUFFS, KRISPIES, LOOPS READY TO EAT | Processed Food Products | Breakfast Foods | IMPORT | Haiti | Non-Detect | 2.6 | 0 |
| 720861 | 2012 | 10/17/2011 | BLACK EYE BEANS | Beans and Legumes | Blackeye Peas | IMPORT | China | Non-Detect | 2.6 | 0 |
| 687682 | 2012 | 10/19/2011 | RAISINS, DRIED OR PASTE | Fruit | Raisins | DOMESTIC | United States | Non-Detect | 2.6 | 0 |
| 721248 | 2012 | 10/20/2011 | COFFEE, BEANS | Non-Juice Beverages | Coffee | IMPORT | Costa Rica | Non-Detect | 2.6 | 0 |
| 722314 | 2012 | 10/24/2011 | BARLEY, WHOLE GRAIN | Grains and Grain Products | Barley | IMPORT | Ethiopia | Non-Detect | 1 | 0 |
| 722375 | 2012 | 10/27/2011 | WHEAT, MILLED (CRUSHED, COARSE GROUND OR CRACKED) | Grains and Grain Products | Wheat | IMPORT | Turkey | Non-Detect | 2.6 | 0 |
| 722549 | 2012 | 10/27/2011 | WHEAT, MILLED (CRUSHED, COARSE GROUND OR CRACKED) | Grains and Grain Products | Wheat | IMPORT | Turkey | Non-Detect | 2.6 | 0 |
| 723055 | 2012 | 10/27/2011 | CORN GRITS, BREWERS ENRICHED WHITE OR YELLOW CORN GRITS,CORN MEAL MUSH, QUICK OR INSTANT COOKING | Processed Food Products | Other Corn Products | IMPORT | South Africa | Non-Detect | 2.6 | 0 |
| 723074 | 2012 | 10/27/2011 | CREAM WHEAT, QUICK OR INSTANT COOKING | Processed Food Products | Breakfast Foods | IMPORT | South Africa | Non-Detect | 2.6 | 0 |
| 723043 | 2012 | 10/27/2011 | CORN GRITS, BREWERS ENRICHED WHITE OR YELLOW CORN GRITS,CORN MEAL MUSH, QUICK OR INSTANT COOKING | Processed Food Products | Other Corn Products | IMPORT | South Africa | Non-Detect | 0.233 | 0 |
| 723168 | 2012 | 10/27/2011 | NON-STANDARD BREAD/ROLLS/BUNS | Processed Food Products | Bread | IMPORT | South Africa | Non-Detect | 0.233 | 0 |
| 722513 | 2012 | 10/27/2011 | CORN, PLAIN MEAL | Grains and Grain Products | Corn | IMPORT | Italy | Non-Detect | 1 | 0 |
| 722849 | 2012 | 10/27/2011 | CREAM WHEAT, QUICK OR INSTANT COOKING | Processed Food Products | Breakfast Foods | IMPORT | South Africa | Trace | 1 | |
| 722867 | 2012 | 10/27/2011 | CORN, PLAIN MEAL | Grains and Grain Products | Corn | IMPORT | South Africa | Trace | 1 | |
| 645249 | 2012 | 10/28/2011 | STANDARDIZED MIX NUT PRODUCT | Nuts and Edible Seeds | Other Nuts and Edible Seeds | IMPORT | United States | Non-Detect | 2.6 | 0 |
| 722714 | 2012 | 10/28/2011 | CORN, PLAIN MEAL | Grains and Grain Products | Corn | IMPORT | South Africa | Trace | 1 | |
| 723155 | 2012 | 10/31/2011 | CORN BRAN | Grains and Grain Products | Corn | IMPORT | Canada | Non-Detect | 2.6 | 0 |
| 723475 | 2012 | 11/2/2011 | BLACK EYE BEANS | Beans and Legumes | Blackeye Peas | IMPORT | China | Non-Detect | 2.6 | 0 |
| 723987 | 2012 | 11/4/2011 | KIDNEY BEAN | Beans and Legumes | Other Beans and Legumes | IMPORT | Nicaragua | Non-Detect | 2.6 | 0 |
| 724379 | 2012 | 11/7/2011 | WHEAT, MILLED (CRUSHED, COARSE GROUND OR CRACKED) | Grains and Grain Products | Wheat | IMPORT | Turkey | Non-Detect | 2.6 | 0 |
| 724975 | 2012 | 11/7/2011 | WHEAT, MILLED (CRUSHED, COARSE GROUND OR CRACKED) | Grains and Grain Products | Wheat | IMPORT | Turkey | Trace | 2.6 | |
| 688153 | 2012 | 11/10/2011 | WHEAT FLOUR, WHOLE | Grains and Grain Products | Wheat | DOMESTIC | United States | Non-Detect | 1 | 0 |
| 528595 | 2012 | 11/10/2011 | WHEAT FLOUR, BROMATED | Grains and Grain Products | Wheat | DOMESTIC | United States | Trace | 2.6 | |
| 687683 | 2012 | 11/14/2011 | OAT FLAKES, ROLLED OATS, PUFFS, KRISPIES, LOOPS READY TO EAT | Processed Food Products | Breakfast Foods | DOMESTIC | United States | Trace | 2.6 | |
| 725354 | 2012 | 11/15/2011 | CORN, ENRICHED MEAL | Grains and Grain Products | Corn | IMPORT | Colombia | Non-Detect | 1 | 0 |
| 727181 | 2012 | 11/23/2011 | MIXED CEREAL, QUICK OR INSTANT COOKING | Processed Food Products | Breakfast Foods | IMPORT | Guatemala | Trace | 1 | |
| 727793 | 2012 | 11/30/2011 | COFFEE, BEANS | Non-Juice Beverages | Coffee | IMPORT | Brazil | Non-Detect | 2.6 | 0 |
| 728685 | 2012 | 12/6/2011 | OTHER BEAN/SEED SPROUTS, N.E.C., BEAN AND PEA SPROUTS | Beans and Legumes | Other Beans and Legumes | IMPORT | Spain | Non-Detect | 2.6 | 0 |
| 670156 | 2012 | 12/6/2011 | BARLEY CEREAL (BABY) | Baby Food Products | Baby Cereals | DOMESTIC | United States | Non-Detect | 1 | 0 |
| 670157 | 2012 | 12/6/2011 | OAT CEREAL (BABY) | Baby Food Products | Baby Cereals | DOMESTIC | United States | Trace | 1 | |
| 727657 | 2012 | 12/6/2011 | WHEAT FLOUR, ENRICHED SELF-RISING | Grains and Grain Products | Wheat | DOMESTIC | United States | Trace | 1 | |
| 714265 | 2012 | 12/7/2011 | RAISINS (DRIED GRAPES) (BERRY) | Fruit | Raisins | DOMESTIC | United States | Trace | 1 | |
| 729412 | 2012 | 12/9/2011 | COFFEE, BEANS | Non-Juice Beverages | Coffee | IMPORT | Brazil | Non-Detect | 2.6 | 0 |
| 729659 | 2012 | 12/12/2011 | WHEAT FLOUR, WHOLE | Grains and Grain Products | Wheat | IMPORT | Canada | Non-Detect | 2.6 | 0 |
| 729682 | 2012 | 12/12/2011 | COFFEE, BEANS | Non-Juice Beverages | Coffee | IMPORT | Colombia | Non-Detect | 2.6 | 0 |
| 731405 | 2012 | 12/13/2011 | RICE CEREAL (BABY) | Baby Food Products | Baby Cereals | IMPORT | United States | Non-Detect | 2.6 | 0 |
| 731414 | 2012 | 12/13/2011 | OAT CEREAL (BABY) | Baby Food Products | Baby Cereals | IMPORT | United States | Non-Detect | 2.6 | 0 |
| 731470 | 2012 | 12/13/2011 | MIXED GRAIN CEREAL (BABY) | Baby Food Products | Baby Cereals | IMPORT | United States | Non-Detect | 2.6 | 0 |
| 715124 | 2012 | 12/15/2011 | RAISINS, DRIED OR PASTE | Fruit | Raisins | DOMESTIC | Unknown | Non-Detect | 2.6 | 0 |
| 730462 | 2012 | 12/15/2011 | CREAM RICE, QUICK OR INSTANT COOKING | Processed Food Products | Breakfast Foods | IMPORT | Guatemala | Non-Detect | 1 | 0 |
| 670153 | 2012 | 12/20/2011 | RICE CEREAL (BABY) | Baby Food Products | Baby Cereals | DOMESTIC | United States | Non-Detect | 1 | 0 |
| 670158 | 2012 | 12/20/2011 | RICE CEREAL (BABY) | Baby Food Products | Baby Cereals | DOMESTIC | United States | Non-Detect | 1 | 0 |
| 671765 | 2012 | 12/21/2011 | WHEAT, WHOLE GRAIN | Grains and Grain Products | Wheat | DOMESTIC | United States | Non-Detect | 2.6 | 0 |
| 671766 | 2012 | 12/21/2011 | BUCKWHEAT FLOUR | Grains and Grain Products | Buckwheat | DOMESTIC | United States | Non-Detect | 1 | 0 |
| 696081 | 2012 | 12/21/2011 | BUCKWHEAT FLOUR | Grains and Grain Products | Buckwheat | DOMESTIC | United States | Non-Detect | 1 | 0 |
| 731637 | 2012 | 12/22/2011 | WHEAT STARCH SNACKS, FRIED/OIL COOK | Processed Food Products | Other Snack Foods | IMPORT | Mexico | Non-Detect | 0.233 | 0 |
| 671764 | 2012 | 12/22/2011 | WHEAT, WHOLE GRAIN | Grains and Grain Products | Wheat | DOMESTIC | United States | Non-Detect | 1 | 0 |
| 731837 | 2012 | 12/27/2011 | GARBANZO BEAN | Beans and Legumes | Garbanzo Beans | IMPORT | Philippines | Non-Detect | 2.6 | 0 |
| 668278 | 2012 | 12/29/2011 | RAISINS (DRIED GRAPES) (BERRY) | Fruit | Raisins | DOMESTIC | United States | Non-Detect | 1 | 0 |
| 732541 | 2012 | 1/4/2012 | CORN FLAKES, PUFFS, KRISPIES, LOOPS READY TO EAT | Processed Food Products | Other Corn Products | IMPORT | Mexico | Trace | 1 | |
| 732871 | 2012 | 1/5/2012 | WHEAT FLOUR, WHOLE | Grains and Grain Products | Wheat | IMPORT | India | Non-Detect | 2.4 | 0 |
| 733116 | 2012 | 1/6/2012 | FAVA BEAN | Beans and Legumes | Other Beans and Legumes | IMPORT | Canada | Non-Detect | 2.4 | 0 |
| 733361 | 2012 | 1/9/2012 | CORN, DRIED OR PASTE | Grains and Grain Products | Corn | IMPORT | China | Non-Detect | 1 | 0 |
| 717316 | 2012 | 1/10/2012 | WHEAT, WHOLE GRAIN | Grains and Grain Products | Wheat | DOMESTIC | United States | Non-Detect | 2.4 | 0 |
| 733754 | 2012 | 1/10/2012 | CREAM RICE, QUICK OR INSTANT COOKING | Processed Food Products | Breakfast Foods | IMPORT | Guatemala | Non-Detect | 1 | 0 |
| 733758 | 2012 | 1/10/2012 | CREAM RICE, QUICK OR INSTANT COOKING | Processed Food Products | Breakfast Foods | IMPORT | Guatemala | Non-Detect | 1 | 0 |
| 734292 | 2012 | 1/12/2012 | OAT FLAKES, ROLLED OATS, PUFFS, KRISPIES, LOOPS READY TO EAT | Processed Food Products | Breakfast Foods | IMPORT | Mexico | Non-Detect | 0.233 | 0 |
| 734284 | 2012 | 1/12/2012 | PINTO BEAN, DRIED OR PASTE | Beans and Legumes | Pinto Beans | IMPORT | Canada | Trace | 1 | |
| 734926 | 2012 | 1/17/2012 | KIDNEY BEAN | Beans and Legumes | Other Beans and Legumes | IMPORT | Nicaragua | Non-Detect | 2.4 | 0 |
| 735332 | 2012 | 1/17/2012 | VERMICELLI | Processed Food Products | Pasta | IMPORT | United Arab Emirates | Non-Detect | 2.4 | 0 |
| 714887 | 2012 | 1/24/2012 | RAISINS, DRIED OR PASTE | Fruit | Raisins | DOMESTIC | United States | Non-Detect | 2.4 | 0 |
| 737085 | 2012 | 1/25/2012 | COFFEE, GROUND | Non-Juice Beverages | Coffee | IMPORT | Netherlands | Non-Detect | 0.233 | 0 |
| 736889 | 2012 | 1/26/2012 | BARLEY, WHOLE GRAIN | Grains and Grain Products | Barley | IMPORT | Canada | Non-Detect | 2.4 | 0 |
| 737556 | 2012 | 1/30/2012 | CORN, PLAIN MEAL | Grains and Grain Products | Corn | IMPORT | France | Non-Detect | 1 | 0 |
| 737786 | 2012 | 1/31/2012 | TORTILLAS | Processed Food Products | Other Corn Products | IMPORT | Mexico | Non-Detect | 1 | 0 |
| 737846 | 2012 | 2/1/2012 | CEREAL BASE MEAT EXTENDERS, WHEAT PROTEIN, ETC. | Other | Other | IMPORT | United States | Trace | 1 | |
| 738124 | 2012 | 2/2/2012 | OATS, WHOLE GRAIN | Grains and Grain Products | Oats | IMPORT | Canada | Non-Detect | 2.4 | 0 |

| | | | | | | | | | | |
|---|---|---|---|---|---|---|---|---|---|---|
| 738121 | 2012 | 2/2/2012 | OATS, WHOLE GRAIN | Grains and Grain Products | Oats | IMPORT | Canada | Positive | 2.5 | 13.8 |
| 738362 | 2012 | 2/3/2012 | OATS, WHOLE GRAIN | Grains and Grain Products | Oats | IMPORT | Canada | Positive | 2.5 | 8.2 |
| 738790 | 2012 | 2/6/2012 | PEA, DRIED OR PASTE | Beans and Legumes | Peas | IMPORT | Canada | Non-Detect | 2.4 | 0 |
| 739277 | 2012 | 2/7/2012 | OATS, WHOLE GRAIN | Grains and Grain Products | Oats | IMPORT | China | Non-Detect | 1 | 0 |
| 739288 | 2012 | 2/7/2012 | CORN, DRIED OR PASTE | Grains and Grain Products | Corn | IMPORT | China | Non-Detect | 1 | 0 |
| 739148 | 2012 | 2/8/2012 | OATS, WHOLE GRAIN | Grains and Grain Products | Oats | IMPORT | Canada | Non-Detect | 2.4 | 0 |
| 739303 | 2012 | 2/8/2012 | CORN, BOLTED MEAL OR FLOUR | Grains and Grain Products | Corn | IMPORT | China | Non-Detect | 1 | 0 |
| 739369 | 2012 | 2/9/2012 | OATS, WHOLE GRAIN | Grains and Grain Products | Oats | IMPORT | Canada | Non-Detect | 2.4 | 0 |
| 739704 | 2012 | 2/9/2012 | BEANS, CORN, AND PEA, N.E.C. (VEGETABLE) | Beans and Legumes | Other Beans and Legumes | IMPORT | Mexico | Non-Detect | 2.4 | 0 |
| 740733 | 2012 | 2/9/2012 | CORN, MILLED PRODUCT N.E.C. | Grains and Grain Products | Corn | IMPORT | China | Non-Detect | 1 | 0 |
| 739368 | 2012 | 2/9/2012 | OATS, WHOLE GRAIN | Grains and Grain Products | Oats | IMPORT | Canada | Positive | 2.6 | 2.9 |
| 739672 | 2012 | 2/10/2012 | SOYBEANS (SEE INDUSTRY 37 FOR 'BEAN CURD') | Beans and Legumes | Soybeans | IMPORT | Canada | Non-Detect | 2.4 | 0 |
| 714325 | 2012 | 2/14/2012 | WHEAT, WHOLE GRAIN | Grains and Grain Products | Wheat | DOMESTIC | United States | Non-Detect | 2.4 | 0 |
| 688152 | 2012 | 2/14/2012 | COFFEE, BEANS | Non-Juice Beverages | Coffee | IMPORT | Colombia | Non-Detect | 1 | 0 |
| 740023 | 2012 | 2/14/2012 | CORN, BOLTED MEAL OR FLOUR | Grains and Grain Products | Corn | IMPORT | Mexico | Non-Detect | 1 | 0 |
| 729026 | 2012 | 2/15/2012 | OAT CEREAL (BABY) | Baby Food Products | Baby Cereals | DOMESTIC | Unknown | Non-Detect | 2.4 | 0 |
| 740617 | 2012 | 2/15/2012 | BLACK EYE BEANS | Beans and Legumes | Blackeye Peas | IMPORT | China | Non-Detect | 0.233 | 0 |
| 729025 | 2012 | 2/15/2012 | WHEAT CEREAL (BABY) | Baby Food Products | Baby Cereals | DOMESTIC | Unknown | Non-Detect | 1 | 0 |
| 740312 | 2012 | 2/15/2012 | CORN, BOLTED MEAL OR FLOUR | Grains and Grain Products | Corn | IMPORT | Mexico | Non-Detect | 1 | 0 |
| 740735 | 2012 | 2/16/2012 | CORN, BOLTED MEAL OR FLOUR | Grains and Grain Products | Corn | IMPORT | Canada | Non-Detect | 1 | 0 |
| 741079 | 2012 | 2/21/2012 | OATS, WHOLE GRAIN | Grains and Grain Products | Oats | IMPORT | Canada | Non-Detect | 2.4 | 0 |
| 741128 | 2012 | 2/21/2012 | SOYBEANS (SEE INDUSTRY 37 FOR 'BEAN CURD') | Beans and Legumes | Soybeans | IMPORT | Canada | Non-Detect | 2.4 | 0 |
| 741832 | 2012 | 2/22/2012 | COFFEE, BEANS | Non-Juice Beverages | Coffee | IMPORT | Taiwan | Non-Detect | 2.4 | 0 |
| 741677 | 2012 | 2/23/2012 | WHEAT, WHOLE GRAIN | Grains and Grain Products | Wheat | IMPORT | Canada | Non-Detect | 2.4 | 0 |
| 741717 | 2012 | 2/23/2012 | WHEAT, WHOLE GRAIN | Grains and Grain Products | Wheat | IMPORT | Canada | Non-Detect | 2.4 | 0 |
| 741916 | 2012 | 2/24/2012 | SOYBEANS (SEE INDUSTRY 37 FOR 'BEAN CURD') | Beans and Legumes | Soybeans | IMPORT | Canada | Non-Detect | 2.4 | 0 |
| 741934 | 2012 | 2/24/2012 | ADZUKI BEANS, DRIED OR PASTE | Beans and Legumes | Other Beans and Legumes | IMPORT | China | Non-Detect | 2.4 | 0 |
| 741947 | 2012 | 2/24/2012 | MUNG BEAN, DRIED OR PASTE | Beans and Legumes | Other Beans and Legumes | IMPORT | China | Non-Detect | 2.4 | 0 |
| 742397 | 2012 | 2/27/2012 | MATZOTH CRACKERS | Processed Food Products | Other Bakery Products | IMPORT | Israel | Non-Detect | 2.4 | 0 |
| 742411 | 2012 | 2/27/2012 | CORN CHIPS, FRIED | Processed Food Products | Other Corn Products | IMPORT | Mexico | Non-Detect | 1 | 0 |
| 742509 | 2012 | 2/27/2012 | CORN, HOMINY GRITS | Grains and Grain Products | Corn | IMPORT | Peru | Non-Detect | 1 | 0 |
| 721677 | 2012 | 2/29/2012 | CORN CEREAL (BABY) | Baby Food Products | Baby Cereals | DOMESTIC | United States | Non-Detect | 2.4 | 0 |
| 742971 | 2012 | 2/29/2012 | KIDNEY BEAN, DRIED OR PASTE | Beans and Legumes | Other Beans and Legumes | IMPORT | Canada | Non-Detect | 2.4 | 0 |
| 742868 | 2012 | 2/29/2012 | CORN, PLAIN MEAL | Grains and Grain Products | Corn | IMPORT | Mexico | Non-Detect | 1 | 0 |
| 742908 | 2012 | 2/29/2012 | CORN, BOLTED MEAL OR FLOUR | Grains and Grain Products | Corn | IMPORT | Mexico | Non-Detect | 1 | 0 |
| 743212 | 2012 | 3/1/2012 | COFFEE, GROUND | Non-Juice Beverages | Coffee | IMPORT | Saudi Arabia | Non-Detect | 2.4 | 0 |
| 743829 | 2012 | 3/6/2012 | WHEAT, WHOLE GRAIN | Grains and Grain Products | Wheat | IMPORT | Canada | Non-Detect | 2.4 | 0 |
| 743861 | 2012 | 3/6/2012 | WHEAT, WHOLE GRAIN | Grains and Grain Products | Wheat | IMPORT | Canada | Non-Detect | 2.4 | 0 |
| 730283 | 2012 | 3/6/2012 | CORN, ENRICHED MEAL | Grains and Grain Products | Corn | IMPORT | Canada | Non-Detect | 1 | 0 |
| 743870 | 2012 | 3/6/2012 | CORN, ENRICHED MEAL | Grains and Grain Products | Corn | IMPORT | Italy | Non-Detect | 1 | 0 |
| 744196 | 2012 | 3/6/2012 | CORN CHIPS, FRIED | Processed Food Products | Other Corn Products | IMPORT | Philippines | Non-Detect | 1 | 0 |
| 722553 | 2012 | 3/7/2012 | MIXED GRAIN CEREAL (BABY) | Baby Food Products | Baby Cereals | DOMESTIC | United States | Non-Detect | 2.4 | 0 |
| 744146 | 2012 | 3/7/2012 | RYE FLOUR | Grains and Grain Products | Rye | IMPORT | France | Non-Detect | 2.4 | 0 |
| 744271 | 2012 | 3/7/2012 | MILLED GRAIN PRODUCTS, N.E.C. | Grains and Grain Products | Other Grains | IMPORT | Mexico | Non-Detect | 0.233 | 0 |
| 744182 | 2012 | 3/7/2012 | KIDNEY BEAN | Beans and Legumes | Other Beans and Legumes | IMPORT | Nicaragua | Non-Detect | 1 | 0 |
| 744262 | 2012 | 3/7/2012 | CORN, BOLTED MEAL OR FLOUR | Grains and Grain Products | Corn | IMPORT | Mexico | Non-Detect | 1 | 0 |
| 744533 | 2012 | 3/8/2012 | BLACKEYE PEAS, DRIED OR PASTE | Beans and Legumes | Blackeye Peas | IMPORT | Mexico | Non-Detect | 2.4 | 0 |
| 744537 | 2012 | 3/8/2012 | GARBANZO BEAN, DRIED OR PASTE | Beans and Legumes | Garbanzo Beans | IMPORT | Mexico | Non-Detect | 2.4 | 0 |
| 719203 | 2012 | 3/8/2012 | COFFEE, BEANS | Non-Juice Beverages | Coffee | IMPORT | Brazil | Non-Detect | 0.233 | 0 |
| 719204 | 2012 | 3/8/2012 | COFFEE, BEANS | Non-Juice Beverages | Coffee | IMPORT | Papua New Guinea | Non-Detect | 0.233 | 0 |
| 693896 | 2012 | 3/12/2012 | WHEAT FLOUR, ENRICHED (ALL PURPOSE) | Grains and Grain Products | Wheat | DOMESTIC | United States | Non-Detect | 2.4 | 0 |
| 731029 | 2012 | 3/12/2012 | CORN FLAKES, PUFFS, KRISPIES, LOOPS READY TO EAT | Processed Food Products | Other Corn Products | DOMESTIC | United States | Non-Detect | 1 | 0 |
| 745074 | 2012 | 3/13/2012 | CEREAL PREPARATIONS NOT ELSEWHERE MENTIONED, N.E.C. | Processed Food Products | Breakfast Foods | IMPORT | Brazil | Non-Detect | 2.4 | 0 |
| 473151 | 2012 | 3/14/2012 | WHEAT FLOUR, ENRICHED (ALL PURPOSE) | Grains and Grain Products | Wheat | DOMESTIC | United States | Non-Detect | 2.4 | 0 |
| 451393 | 2012 | 3/14/2012 | WHEAT CEREAL (BABY) | Baby Food Products | Baby Cereals | DOMESTIC | United States | Non-Detect | 1 | 0 |
| 731846 | 2012 | 3/14/2012 | CORN, ENRICHED MEAL | Grains and Grain Products | Corn | DOMESTIC | Unknown | Non-Detect | 1 | 0 |
| 745412 | 2012 | 3/14/2012 | CORN, SELF-RISING MEAL | Grains and Grain Products | Corn | DOMESTIC | United States | Non-Detect | 1 | 0 |
| 745504 | 2012 | 3/14/2012 | COFFEE, BEANS | Non-Juice Beverages | Coffee | IMPORT | Honduras | Non-Detect | 1 | 0 |
| 709971 | 2012 | 3/15/2012 | RYE FLOUR | Grains and Grain Products | Rye | DOMESTIC | United States | Trace | 1 | |
| 744732 | 2012 | 3/16/2012 | OATS, WHOLE GRAIN | Grains and Grain Products | Oats | IMPORT | Canada | Non-Detect | 1 | 0 |
| 744733 | 2012 | 3/16/2012 | OATS, WHOLE GRAIN | Grains and Grain Products | Oats | IMPORT | Canada | Trace | 1 | |
| 746707 | 2012 | 3/22/2012 | COFFEE, BEANS | Non-Juice Beverages | Coffee | IMPORT | France | Non-Detect | 2.4 | 0 |
| 719207 | 2012 | 3/29/2012 | WHEAT FLOUR, BROMATED | Grains and Grain Products | Wheat | DOMESTIC | United States | Non-Detect | 2.4 | 0 |
| 748135 | 2012 | 3/30/2012 | LENTILS, DRIED OR PASTE | Beans and Legumes | Lentils | IMPORT | Turkey | Non-Detect | 2.4 | 0 |
| 748086 | 2012 | 3/30/2012 | CORN CHIPS, FRIED | Processed Food Products | Other Corn Products | IMPORT | Mexico | Non-Detect | 1 | 0 |
| 748254 | 2012 | 4/2/2012 | CORN CHIPS, FRIED | Processed Food Products | Other Corn Products | IMPORT | Mexico | Non-Detect | 1 | 0 |
| 748573 | 2012 | 4/3/2012 | COFFEE, BEANS | Non-Juice Beverages | Coffee | IMPORT | Canada | Non-Detect | 2.4 | 0 |
| 627458 | 2012 | 4/3/2012 | CORN FLAKES, PUFFS, KRISPIES, LOOPS READY TO EAT | Processed Food Products | Other Corn Products | DOMESTIC | United States | Non-Detect | 1 | 0 |
| 719208 | 2012 | 4/3/2012 | GARBANZO BEAN, DRIED OR PASTE | Beans and Legumes | Garbanzo Beans | DOMESTIC | United States | Non-Detect | 1 | 0 |
| 748572 | 2012 | 4/3/2012 | COFFEE, BEANS | Non-Juice Beverages | Coffee | IMPORT | India | Non-Detect | 1 | 0 |
| 748571 | 2012 | 4/3/2012 | COFFEE, BEANS, DECAFFEINATED | Non-Juice Beverages | Coffee | IMPORT | Germany | Trace | 4.99 | |
| 716995 | 2012 | 4/5/2012 | CORN GRITS, BREWERS ENRICHED WHITE OR YELLOW CORN GRITS,CORN MEAL MUSH, QUICK OR INSTANT COOKING | Processed Food Products | Other Corn Products | DOMESTIC | United States | Non-Detect | 2.4 | 0 |
| 716996 | 2012 | 4/5/2012 | CORN GRITS, BREWERS ENRICHED WHITE OR YELLOW CORN GRITS,CORN MEAL MUSH, QUICK OR INSTANT COOKING | Processed Food Products | Other Corn Products | DOMESTIC | United States | Non-Detect | 2.4 | 0 |
| 750000 | 2012 | 4/11/2012 | COFFEE, INSTANT | Non-Juice Beverages | Coffee | IMPORT | United Kingdom | Non-Detect | 0.233 | 0 |
| 750021 | 2012 | 4/12/2012 | SOYBEANS (SEE INDUSTRY 37 FOR 'BEAN CURD') | Beans and Legumes | Soybeans | IMPORT | Canada | Non-Detect | 2.4 | 0 |
| 750045 | 2012 | 4/12/2012 | OATS, WHOLE GRAIN | Grains and Grain Products | Oats | IMPORT | Canada | Non-Detect | 2.4 | 0 |
| 750099 | 2012 | 4/12/2012 | BLACK BEAN, DRIED OR PASTE | Beans and Legumes | Black Beans | IMPORT | China | Non-Detect | 2.4 | 0 |
| 750757 | 2012 | 4/17/2012 | OAT FLOUR | Grains and Grain Products | Oats | IMPORT | Canada | Non-Detect | 1 | 0 |
| 750906 | 2012 | 4/17/2012 | CORN, BOLTED MEAL OR FLOUR | Grains and Grain Products | Corn | IMPORT | El Salvador | Non-Detect | 1 | 0 |
| 750927 | 2012 | 4/17/2012 | FARINA, WHEAT | Grains and Grain Products | Wheat | IMPORT | India | Non-Detect | 1 | 0 |
| 751344 | 2012 | 4/19/2012 | BARLEY FLOUR | Grains and Grain Products | Barley | IMPORT | Mexico | Non-Detect | 0.233 | 0 |
| 701577 | 2012 | 4/24/2012 | BARLEY, WHOLE GRAIN | Grains and Grain Products | Barley | DOMESTIC | United States | Non-Detect | 0.233 | 0 |
| 751787 | 2012 | 4/24/2012 | SOYBEAN FLOUR, MEAL, OR POWDER | Beans and Legumes | Soybeans | IMPORT | Argentina | Non-Detect | 1 | 0 |
| 752780 | 2012 | 4/27/2012 | COFFEE, BEANS | Non-Juice Beverages | Coffee | IMPORT | Indonesia | Non-Detect | 2.4 | 0 |
| 754055 | 2012 | 5/7/2012 | CORN CHIPS, FRIED | Processed Food Products | Other Corn Products | IMPORT | Canada | Non-Detect | 1 | 0 |
| 754241 | 2012 | 5/7/2012 | WHEAT FLOUR, DURUM | Grains and Grain Products | Wheat | IMPORT | Italy | Trace | 1 | |
| 754495 | 2012 | 5/8/2012 | OATMEAL, REGULAR, FRUIT OR SPICE ADDED, QUICK OR INSTANT COOKING | Processed Food Products | Breakfast Foods | IMPORT | United Kingdom | Non-Detect | 2.4 | 0 |
| 754226 | 2012 | 5/8/2012 | COFFEE, BEANS | Non-Juice Beverages | Coffee | IMPORT | Vietnam | Non-Detect | 1 | 0 |
| 754709 | 2012 | 5/10/2012 | WHEAT MEAL | Grains and Grain Products | Wheat | IMPORT | Canada | Non-Detect | 1 | 0 |

| | | | | | | | | | | |
|---|---|---|---|---|---|---|---|---|---|---|
| 755058 | 2012 | 5/10/2012 | WHEAT FLOUR, WHOLE | Grains and Grain Products | Wheat | IMPORT | India | Non-Detect | 1 | 0 |
| 756996 | 2012 | 5/21/2012 | RICE CEREAL (BABY) | Baby Food Products | Baby Cereals | IMPORT | Chile | Non-Detect | 2.4 | 0 |
| 757294 | 2012 | 5/22/2012 | WHEAT FLOUR, WHOLE | Grains and Grain Products | Wheat | IMPORT | India | Non-Detect | 1 | 0 |
| 757323 | 2012 | 5/22/2012 | WHEAT FLOUR, WHOLE | Grains and Grain Products | Wheat | IMPORT | India | Non-Detect | 1 | 0 |
| 757607 | 2012 | 5/24/2012 | BUCKWHEAT, WHOLE GRAIN | Grains and Grain Products | Buckwheat | IMPORT | China | Non-Detect | 1 | 0 |
| 758375 | 2012 | 5/30/2012 | CORN CHIPS, FRIED | Processed Food Products | Other Corn Products | IMPORT | Philippines | Trace | 1 | |
| 758169 | 2012 | 5/31/2012 | OATMEAL, REGULAR, FRUIT OR SPICE ADDED, QUICK OR INSTANT COOKING | Processed Food Products | Breakfast Foods | IMPORT | Dominican Republic (the) | Non-Detect | 2.4 | 0 |
| 758400 | 2012 | 6/1/2012 | MUNG BEAN | Beans and Legumes | Other Beans and Legumes | IMPORT | Canada | Non-Detect | 2.4 | 0 |
| 758406 | 2012 | 6/1/2012 | MUNG BEAN | Beans and Legumes | Other Beans and Legumes | IMPORT | Canada | Non-Detect | 2.4 | 0 |
| 758410 | 2012 | 6/1/2012 | MUNG BEAN | Beans and Legumes | Other Beans and Legumes | IMPORT | Canada | Non-Detect | 2.4 | 0 |
| 758417 | 2012 | 6/1/2012 | PEAS | Beans and Legumes | Peas | IMPORT | Canada | Non-Detect | 2.4 | 0 |
| 758421 | 2012 | 6/1/2012 | GARBANZO BEAN | Beans and Legumes | Garbanzo Beans | IMPORT | Canada | Non-Detect | 2.4 | 0 |
| 758484 | 2012 | 6/1/2012 | WHEAT FLOUR, DURUM | Grains and Grain Products | Wheat | IMPORT | India | Non-Detect | 1 | 0 |
| 758722 | 2012 | 6/5/2012 | WHEAT, WHOLE GRAIN | Grains and Grain Products | Wheat | IMPORT | Canada | Non-Detect | 2.4 | 0 |
| 758998 | 2012 | 6/5/2012 | OATS, WHOLE GRAIN | Grains and Grain Products | Oats | IMPORT | Canada | Non-Detect | 2.4 | 0 |
| 759000 | 2012 | 6/5/2012 | OATS, WHOLE GRAIN | Grains and Grain Products | Oats | IMPORT | Canada | Non-Detect | 2.4 | 0 |
| 759098 | 2012 | 6/6/2012 | BARLEY, WHOLE GRAIN | Grains and Grain Products | Barley | IMPORT | Canada | Non-Detect | 2.4 | 0 |
| 759103 | 2012 | 6/6/2012 | OATS, WHOLE GRAIN | Grains and Grain Products | Oats | IMPORT | Canada | Non-Detect | 2.4 | 0 |
| 759740 | 2012 | 6/11/2012 | MACARONI | Processed Food Products | Pasta | IMPORT | Mexico | Non-Detect | 1 | 0 |
| 759743 | 2012 | 6/11/2012 | SPAGHETTI | Processed Food Products | Pasta | IMPORT | Mexico | Non-Detect | 1 | 0 |
| 760416 | 2012 | 6/13/2012 | LIMA BEAN | Beans and Legumes | Other Beans and Legumes | IMPORT | Peru | Non-Detect | 0.233 | 0 |
| 760539 | 2012 | 6/13/2012 | FAVA BEAN, BEAN AND PEA SPROUTS | Beans and Legumes | Other Beans and Legumes | IMPORT | Canada | Non-Detect | 1 | 0 |
| 751668 | 2012 | 6/18/2012 | SOYBEANS, WHOLE GRAIN | Beans and Legumes | Soybeans | DOMESTIC | United States | Non-Detect | 0.233 | 0 |
| 761101 | 2012 | 6/19/2012 | BARLEY, WHOLE GRAIN | Grains and Grain Products | Barley | IMPORT | Canada | Non-Detect | 0.233 | 0 |
| 762234 | 2012 | 6/27/2012 | OATMEAL, REGULAR, FRUIT OR SPICE ADDED, QUICK OR INSTANT COOKING | Processed Food Products | Breakfast Foods | IMPORT | Dominican Republic (the) | Non-Detect | 2.4 | 0 |
| 762287 | 2012 | 6/27/2012 | OATMEAL, REGULAR, FRUIT OR SPICE ADDED, QUICK OR INSTANT COOKING | Processed Food Products | Breakfast Foods | IMPORT | Dominican Republic (the) | Non-Detect | 2.4 | 0 |
| 760184 | 2012 | 7/2/2012 | PEA, DRIED OR PASTE | Beans and Legumes | Peas | DOMESTIC | United States | Non-Detect | 2.4 | 0 |
| 763296 | 2012 | 7/5/2012 | BARLEY FLOUR | Grains and Grain Products | Barley | IMPORT | Canada | Non-Detect | 2.4 | 0 |
| 763457 | 2012 | 7/6/2012 | COFFEE, BEANS | Non-Juice Beverages | Coffee | IMPORT | Dominican Republic (the) | Non-Detect | 2.4 | 0 |
| 764438 | 2012 | 7/16/2012 | RICE CEREAL (BABY) | Baby Food Products | Baby Cereals | IMPORT | Chile | Non-Detect | 2.4 | 0 |
| 764453 | 2012 | 7/16/2012 | RICE CEREAL (BABY) | Baby Food Products | Baby Cereals | IMPORT | Chile | Non-Detect | 2.4 | 0 |
| 765521 | 2012 | 7/24/2012 | WHEAT, WHOLE GRAIN | Grains and Grain Products | Wheat | IMPORT | Canada | Non-Detect | 2.4 | 0 |
| 723270 | 2012 | 7/31/2012 | OATS, WHOLE GRAIN | Grains and Grain Products | Oats | DOMESTIC | United States | Positive | 2.6 | 6.26 |
| 723271 | 2012 | 8/1/2012 | OATS, WHOLE GRAIN | Grains and Grain Products | Oats | DOMESTIC | United States | Non-Detect | 2.4 | 0 |
| 761872 | 2012 | 8/9/2012 | RAISINS, DRIED OR PASTE | Fruit | Raisins | DOMESTIC | United States | Trace | 5.2 | |
| 769056 | 2012 | 8/15/2012 | OATS, WHOLE GRAIN | Grains and Grain Products | Oats | IMPORT | United Kingdom | Non-Detect | 2.4 | 0 |
| 723272 | 2012 | 8/16/2012 | WHEAT, WHOLE GRAIN | Grains and Grain Products | Wheat | DOMESTIC | United States | Non-Detect | 2.4 | 0 |
| 768477 | 2012 | 8/16/2012 | WHEAT, WHOLE GRAIN | Grains and Grain Products | Wheat | DOMESTIC | United States | Non-Detect | 2.4 | 0 |
| 769960 | 2012 | 8/21/2012 | CORN, WHOLE GRAIN | Grains and Grain Products | Corn | IMPORT | Mexico | Non-Detect | 1 | 0 |
| 770494 | 2012 | 8/22/2012 | GRAIN, WHOLE, N.E.C. | Grains and Grain Products | Other Grains | IMPORT | Taiwan | Non-Detect | 1 | 0 |
| 770525 | 2012 | 8/22/2012 | GRAIN, WHOLE, N.E.C. | Grains and Grain Products | Other Grains | IMPORT | Taiwan | Non-Detect | 1 | 0 |
| 743708 | 2012 | 9/7/2012 | WHEAT, WHOLE GRAIN | Grains and Grain Products | Wheat | DOMESTIC | United States | Non-Detect | 2.4 | 0 |
| 543864 | 2012 | 9/25/2012 | WHEAT, WHOLE GRAIN | Grains and Grain Products | Wheat | DOMESTIC | United States | Non-Detect | 0.233 | 0 |
| 543865 | 2012 | 9/25/2012 | WHEAT, WHOLE GRAIN | Grains and Grain Products | Wheat | DOMESTIC | United States | Non-Detect | 0.233 | 0 |
| 622707 | 2012 | 9/25/2012 | SOY BASE FORMULA PRODUCT, POWDER FORMULA | Baby Food Products | Baby Formula | DOMESTIC | United States | Non-Detect | 0.233 | 0 |
| 622708 | 2012 | 9/25/2012 | SOY BASE FORMULA PRODUCT, POWDER FORMULA | Baby Food Products | Baby Formula | DOMESTIC | United States | Non-Detect | 0.233 | 0 |
| 755476 | 2012 | 9/25/2012 | OAT CEREAL (BABY) | Baby Food Products | Baby Cereals | DOMESTIC | United States | Non-Detect | 0.233 | 0 |
| 755477 | 2012 | 9/25/2012 | RICE CEREAL (BABY) | Baby Food Products | Baby Cereals | DOMESTIC | United States | Non-Detect | 0.233 | 0 |
| 755478 | 2012 | 9/25/2012 | WHEAT CEREAL (BABY) | Baby Food Products | Baby Cereals | DOMESTIC | United States | Non-Detect | 0.233 | 0 |
| 758047 | 2012 | 9/25/2012 | WHEAT FLOUR, ENRICHED (ALL PURPOSE) | Grains and Grain Products | Wheat | IMPORT | Canada | Non-Detect | 1 | 0 |
| 775089 | 2012 | 9/25/2012 | GARBANZO BEAN | Beans and Legumes | Garbanzo Beans | IMPORT | Pakistan | Non-Detect | 1 | 0 |
| 749395 | 2012 | 9/26/2012 | RICE CEREAL (BABY) | Baby Food Products | Baby Cereals | DOMESTIC | United States | Non-Detect | 2.4 | 0 |
| 528596 | 2012 | 9/27/2012 | MIXED CEREAL FLAKES, SHREDDED AND OTHER FORMS READY TO EAT | Processed Food Products | Breakfast Foods | DOMESTIC | United States | Non-Detect | 2.8 | 0 |
| 741897 | 2012 | 9/27/2012 | CORN, ENRICHED MEAL | Grains and Grain Products | Corn | DOMESTIC | United States | Non-Detect | 0.233 | 0 |
| 528597 | 2012 | 9/28/2012 | WHEAT BRAN (HUMAN USE) | Grains and Grain Products | Wheat | DOMESTIC | United States | Non-Detect | 2.8 | 0 |

## FY2013 Data

| Sample Number | Fiscal Year | Collection Date | Product Name | Food Category | Food Subcategory | Origin Type | Country of Origin | Non-Detect, Trace, or Positive (>LOQ) | LOQ (ppb) | Amount found (ppb) |
|---|---|---|---|---|---|---|---|---|---|---|
| 776038 | 2013 | 10/3/2012 | MUNG BEAN, DRIED OR PASTE | Beans and Legumes | Other Beans and Legumes | IMPORT | India | Non-Detect | 2.4 | 0 |
| 777477 | 2013 | 10/11/2012 | WHEAT, MILLED (CRUSHED, COARSE GROUND OR CRACKED) | Grains and Grain Products | Wheat | IMPORT | Canada | Non-Detect | 1 | 0 |
| 777872 | 2013 | 10/15/2012 | GARBANZO BEAN, DRIED OR PASTE | Beans and Legumes | Garbanzo Beans | IMPORT | Mexico | Non-Detect | 2.4 | 0 |
| 777874 | 2013 | 10/15/2012 | NOODLES, WHEAT AND SOY | Processed Food Products | Pasta | IMPORT | Vietnam | Non-Detect | 1 | 0 |
| 777875 | 2013 | 10/15/2012 | CORN, WHOLE GRAIN | Grains and Grain Products | Corn | IMPORT | Vietnam | Non-Detect | 1 | 0 |
| 779590 | 2013 | 10/16/2012 | SOYBEANS (SEE INDUSTRY 37 FOR 'BEAN CURD') | Beans and Legumes | Soybeans | DOMESTIC | United States | Non-Detect | 2.4 | 0 |
| 777892 | 2013 | 10/16/2012 | CORN FLAKES, PUFFS, KRISPIES, LOOPS READY TO EAT | Processed Food Products | Other Corn Products | IMPORT | Mexico | Non-Detect | 0.233 | 0 |
| 778024 | 2013 | 10/16/2012 | OATMEAL PLAIN COOKIES, BISCUITS, AND WAFERS | Processed Food Products | Other Bakery Products | IMPORT | Mexico | Non-Detect | 0.233 | 0 |
| 778051 | 2013 | 10/16/2012 | PEAS | Beans and Legumes | Peas | IMPORT | Canada | Trace | 1 | |
| 779363 | 2013 | 10/23/2012 | SPAGHETTI | Processed Food Products | Pasta | IMPORT | Italy | Non-Detect | 1 | 0 |
| 779367 | 2013 | 10/23/2012 | SPAGHETTI | Processed Food Products | Pasta | IMPORT | Italy | Trace | 1 | |
| 779671 | 2013 | 10/24/2012 | MALT, BARLEY | Grains and Grain Products | Barley | IMPORT | Germany | Non-Detect | 0.233 | 0 |
| 779485 | 2013 | 10/24/2012 | GARBANZO BEAN, DRIED OR PASTE | Beans and Legumes | Garbanzo Beans | IMPORT | Canada | Trace | 1 | |
| 779829 | 2013 | 10/25/2012 | COFFEE, BEANS | Non-Juice Beverages | Coffee | IMPORT | Mexico | Non-Detect | 0.233 | 0 |
| 780190 | 2013 | 10/25/2012 | FLAVORED OR PARTY CRACKERS | Processed Food Products | Other Bakery Products | IMPORT | Mexico | Non-Detect | 0.233 | 0 |
| 780202 | 2013 | 10/28/2012 | BARLEY, WHOLE GRAIN | Grains and Grain Products | Barley | IMPORT | Canada | Non-Detect | 0.233 | 0 |
| 712025 | 2013 | 10/29/2012 | CANNELLINI BEAN, DRIED OR PASTE | Beans and Legumes | Other Beans and Legumes | DOMESTIC | United States | Non-Detect | 0.233 | 0 |
| 781124 | 2013 | 11/1/2012 | COFFEE, BEANS | Non-Juice Beverages | Coffee | IMPORT | Peru | Non-Detect | 0.233 | 0 |
| 781190 | 2013 | 11/1/2012 | BARLEY, WHOLE GRAIN | Grains and Grain Products | Barley | IMPORT | Thailand | Positive | 1 | 5.58 |
| 781343 | 2013 | 11/2/2012 | SPAGHETTI | Processed Food Products | Pasta | IMPORT | Korea (the Republic of) | Non-Detect | 1 | 0 |
| 712026 | 2013 | 11/5/2012 | NAVY (WHITE) BEANS (BAKED BEANS, PORK AND BEANS), DRIED OR PASTE | Beans and Legumes | Other Beans and Legumes | DOMESTIC | United States | Non-Detect | 0.233 | 0 |
| 781788 | 2013 | 11/6/2012 | COFFEE, BEANS | Non-Juice Beverages | Coffee | IMPORT | Peru | Non-Detect | 1 | 0 |
| 781846 | 2013 | 11/6/2012 | SPAGHETTI | Processed Food Products | Pasta | IMPORT | Italy | Non-Detect | 1 | 0 |
| 773508 | 2013 | 11/8/2012 | CORN FLAKES, PUFFS, KRISPIES, LOOPS READY TO EAT | Processed Food Products | Other Corn Products | DOMESTIC | United States | Non-Detect | 2.4 | 0 |
| 782107 | 2013 | 11/8/2012 | COFFEE, BEANS | Non-Juice Beverages | Coffee | IMPORT | Indonesia | Non-Detect | 1 | 0 |
| 782321 | 2013 | 11/8/2012 | SPAGHETTI | Processed Food Products | Pasta | IMPORT | Italy | Non-Detect | 1 | 0 |
| 782388 | 2013 | 11/8/2012 | MIXED CEREAL, QUICK OR INSTANT COOKING | Processed Food Products | Breakfast Foods | IMPORT | Guatemala | Non-Detect | 1 | 0 |
| 783263 | 2013 | 11/8/2012 | SEMOLINA, WHEAT | Grains and Grain Products | Wheat | IMPORT | Argentina | Non-Detect | 1 | 0 |
| 783282 | 2013 | 11/8/2012 | CORN, BOLTED MEAL OR FLOUR | Grains and Grain Products | Corn | IMPORT | Argentina | Non-Detect | 1 | 0 |
| 782406 | 2013 | 11/9/2012 | CORN, PLAIN MEAL | Grains and Grain Products | Corn | IMPORT | Nicaragua | Non-Detect | 0.233 | 0 |
| 782664 | 2013 | 11/9/2012 | WHEAT, MILLED (CRUSHED, COARSE GROUND OR CRACKED) | Grains and Grain Products | Wheat | IMPORT | Turkey | Non-Detect | 1 | 0 |
| 782821 | 2013 | 11/13/2012 | CORN NUTS SNACK | Processed Food Products | Other Corn Products | IMPORT | Philippines | Non-Detect | 1 | 0 |
| 783608 | 2013 | 11/14/2012 | SPAGHETTI | Processed Food Products | Pasta | IMPORT | Italy | Non-Detect | 1 | 0 |
| 783394 | 2013 | 11/16/2012 | RAISINS, DRIED OR PASTE | Fruit | Raisins | IMPORT | United States | Positive | 1 | 2.42 |
| 754949 | 2013 | 11/20/2012 | CORN FLAKES, PUFFS, KRISPIES, LOOPS READY TO EAT | Processed Food Products | Other Corn Products | DOMESTIC | United States | Non-Detect | 1 | 0 |
| 783827 | 2013 | 11/20/2012 | CORN, BOLTED MEAL OR FLOUR | Grains and Grain Products | Corn | IMPORT | India | Non-Detect | 1 | 0 |
| 665439 | 2013 | 11/21/2012 | WHEAT FLOUR, N.E.C. | Grains and Grain Products | Wheat | DOMESTIC | United States | Non-Detect | 2.6 | 0 |
| 754948 | 2013 | 11/21/2012 | CORN, ENRICHED MEAL | Grains and Grain Products | Corn | DOMESTIC | United States | Non-Detect | 1 | 0 |
| 784082 | 2013 | 11/23/2012 | COFFEE, BEANS | Non-Juice Beverages | Coffee | IMPORT | France | Non-Detect | 0.233 | 0 |
| 784574 | 2013 | 11/26/2012 | WHEAT FLOUR, WHOLE | Grains and Grain Products | Wheat | IMPORT | India | Non-Detect | 1 | 0 |
| 784706 | 2013 | 11/27/2012 | WHEAT, WHOLE GRAIN | Grains and Grain Products | Wheat | IMPORT | Canada | Non-Detect | 2.4 | 0 |
| 784492 | 2013 | 11/27/2012 | COFFEE, BEANS | Non-Juice Beverages | Coffee | IMPORT | Guatemala | Non-Detect | 1 | 0 |
| 784819 | 2013 | 11/28/2012 | CORN FLAKES, PUFFS, KRISPIES, LOOPS READY TO EAT | Processed Food Products | Other Corn Products | IMPORT | Canada | Non-Detect | 2.4 | 0 |
| 785263 | 2013 | 11/29/2012 | BLACK BEAN, DRIED OR PASTE | Beans and Legumes | Black Beans | IMPORT | China | Non-Detect | 2.4 | 0 |
| 785238 | 2013 | 11/29/2012 | WHEAT FLOUR, WHOLE BROMATED | Grains and Grain Products | Wheat | IMPORT | India | Non-Detect | 1 | 0 |
| 785275 | 2013 | 11/30/2012 | SOYBEANS (EDIBLE SEED) | Beans and Legumes | Soybeans | IMPORT | China | Non-Detect | 2.4 | 0 |
| 785244 | 2013 | 11/30/2012 | COFFEE, BEANS | Non-Juice Beverages | Coffee | IMPORT | Haiti | Non-Detect | 0.233 | 0 |
| 785502 | 2013 | 12/3/2012 | WHEAT FLOUR, WHOLE | Grains and Grain Products | Wheat | IMPORT | Canada | Trace | 1 | |
| 785759 | 2013 | 12/5/2012 | WHEAT, WHOLE GRAIN | Grains and Grain Products | Wheat | IMPORT | Canada | Non-Detect | 2.4 | 0 |
| 785887 | 2013 | 12/5/2012 | VEGETABLE MACARONI PRODUCTS | Processed Food Products | Pasta | IMPORT | Romania | Non-Detect | 1 | 0 |
| 786691 | 2013 | 12/11/2012 | COFFEE, BEANS | Non-Juice Beverages | Coffee | IMPORT | Ecuador | Non-Detect | 2.4 | 0 |
| 787331 | 2013 | 12/12/2012 | RAISINS, DRIED OR PASTE | Fruit | Raisins | IMPORT | Pakistan | Non-Detect | 1 | 0 |
| 787556 | 2013 | 12/13/2012 | MILLED GRAIN PRODUCTS, N.E.C. | Grains and Grain Products | Other Grains | IMPORT | Italy | Non-Detect | 1 | 0 |
| 787766 | 2013 | 12/14/2012 | RYE BREAD, ROLLS, BUNS, ETC PREPARED DRY MIX WITHOUT MILK OR EGG | Processed Food Products | Bread | IMPORT | Canada | Trace | 1 | |
| 788005 | 2013 | 12/18/2012 | COFFEE, BEANS | Non-Juice Beverages | Coffee | IMPORT | Nicaragua | Non-Detect | 1 | 0 |
| 788260 | 2013 | 12/19/2012 | COFFEE, BEANS | Non-Juice Beverages | Coffee | IMPORT | Italy | Non-Detect | 2.4 | 0 |
| 782788 | 2013 | 1/3/2013 | WHEAT FLOUR, WHOLE | Grains and Grain Products | Wheat | DOMESTIC | Unknown | Non-Detect | 2.3 | 0 |
| 789886 | 2013 | 1/9/2013 | MALT, BARLEY | Grains and Grain Products | Barley | IMPORT | United Kingdom | Non-Detect | 2.3 | 0 |
| 777592 | 2013 | 1/9/2013 | OATS, WHOLE GRAIN | Grains and Grain Products | Oats | IMPORT | Canada | Positive | 2.6 | 3.77 |
| 790082 | 2013 | 1/10/2013 | OATS, WHOLE GRAIN | Grains and Grain Products | Oats | IMPORT | Canada | Non-Detect | 2.3 | 0 |
| 760199 | 2013 | 1/15/2013 | CORN, DEGERMINATED MEAL | Grains and Grain Products | Corn | DOMESTIC | United States | Non-Detect | 2.3 | 0 |
| 760200 | 2013 | 1/15/2013 | CORN FLAKES, PUFFS, KRISPIES, LOOPS READY TO EAT | Processed Food Products | Other Corn Products | DOMESTIC | United States | Non-Detect | 2.4 | 0 |
| 791405 | 2013 | 1/17/2013 | CORN, (VEGETABLE) | Grains and Grain Products | Corn | IMPORT | China | Non-Detect | 1 | 0 |
| 791456 | 2013 | 1/17/2013 | CORN, MILLED PRODUCT N.E.C. | Grains and Grain Products | Corn | IMPORT | India | Non-Detect | 1 | 0 |
| 791745 | 2013 | 1/22/2013 | WHEAT, WHOLE GRAIN | Grains and Grain Products | Wheat | IMPORT | Canada | Non-Detect | 1 | 0 |
| 791962 | 2013 | 1/22/2013 | CORN CHIPS, FRIED | Processed Food Products | Other Corn Products | IMPORT | Guatemala | Non-Detect | 1 | 0 |
| 792001 | 2013 | 1/22/2013 | CORN CHIPS, FRIED | Processed Food Products | Other Corn Products | IMPORT | Guatemala | Non-Detect | 1 | 0 |
| 792297 | 2013 | 1/24/2013 | COFFEE, BEANS | Non-Juice Beverages | Coffee | IMPORT | Indonesia | Non-Detect | 2.3 | 0 |
| 792478 | 2013 | 1/24/2013 | KIDNEY BEAN | Beans and Legumes | Other Beans and Legumes | IMPORT | China | Non-Detect | 1 | 0 |
| 793164 | 2013 | 1/29/2013 | COFFEE, BEANS | Non-Juice Beverages | Coffee | IMPORT | Vietnam | Non-Detect | 2.3 | 0 |
| 793317 | 2013 | 1/29/2013 | CORN, WHOLE GRAIN | Grains and Grain Products | Corn | IMPORT | Mexico | Non-Detect | 1 | 0 |
| 794046 | 2013 | 1/31/2013 | PINTO BEAN | Beans and Legumes | Pinto Beans | IMPORT | Mexico | Non-Detect | 0.233 | 0 |
| 794614 | 2013 | 2/5/2013 | LIMA BEAN, DRIED OR PASTE | Beans and Legumes | Other Beans and Legumes | IMPORT | Peru | Non-Detect | 2.3 | 0 |
| 794618 | 2013 | 2/5/2013 | LENTILS, DRIED OR PASTE | Beans and Legumes | Lentils | IMPORT | Canada | Non-Detect | 2.3 | 0 |
| 794631 | 2013 | 2/5/2013 | LENTILS, DRIED OR PASTE | Beans and Legumes | Lentils | IMPORT | Canada | Non-Detect | 2.3 | 0 |
| 794642 | 2013 | 2/5/2013 | KIDNEY BEAN, DRIED OR PASTE | Beans and Legumes | Other Beans and Legumes | IMPORT | Canada | Non-Detect | 2.3 | 0 |
| 794647 | 2013 | 2/5/2013 | KIDNEY BEAN, DRIED OR PASTE | Beans and Legumes | Other Beans and Legumes | IMPORT | Argentina | Non-Detect | 2.3 | 0 |
| 794652 | 2013 | 2/5/2013 | MIXED BEAN/SEED SPROUTS, DRIED OR PASTE | Beans and Legumes | Other Beans and Legumes | IMPORT | Canada | Non-Detect | 2.3 | 0 |
| 794876 | 2013 | 2/5/2013 | LENTILS, DRIED OR PASTE | Beans and Legumes | Lentils | IMPORT | Canada | Non-Detect | 2.3 | 0 |
| 786665 | 2013 | 2/6/2013 | SOYBEANS, WHOLE GRAIN | Beans and Legumes | Soybeans | DOMESTIC | United States | Non-Detect | 2.3 | 0 |
| 795096 | 2013 | 2/7/2013 | OATS, WHOLE GRAIN | Grains and Grain Products | Oats | IMPORT | Mexico | Non-Detect | 0.233 | 0 |
| 795446 | 2013 | 2/11/2013 | COFFEE, BEANS, DECAFFEINATED | Non-Juice Beverages | Coffee | IMPORT | Canada | Non-Detect | 1 | 0 |
| 795458 | 2013 | 2/11/2013 | COFFEE, BEANS, DECAFFEINATED | Non-Juice Beverages | Coffee | IMPORT | Canada | Non-Detect | 1 | 0 |
| 780731 | 2013 | 2/12/2013 | OATMEAL, REGULAR, FRUIT OR SPICE ADDED, QUICK OR INSTANT COOKING | Processed Food Products | Breakfast Foods | DOMESTIC | United States | Non-Detect | 0.233 | 0 |
| 796566 | 2013 | 2/14/2013 | WHEAT MEAL | Grains and Grain Products | Wheat | IMPORT | El Salvador | Non-Detect | 1 | 0 |
| 796622 | 2013 | 2/14/2013 | OATMEAL, REGULAR, FRUIT OR SPICE ADDED, QUICK OR INSTANT COOKING | Processed Food Products | Breakfast Foods | IMPORT | El Salvador | Non-Detect | 1 | 0 |
| 796631 | 2013 | 2/14/2013 | OATMEAL, REGULAR, FRUIT OR SPICE ADDED, QUICK OR INSTANT COOKING | Processed Food Products | Breakfast Foods | IMPORT | El Salvador | Non-Detect | 1 | 0 |
| 796860 | 2013 | 2/20/2013 | PEAS | Beans and Legumes | Peas | IMPORT | Canada | Non-Detect | 1 | 0 |

| | | | | | | | | | | |
|---|---|---|---|---|---|---|---|---|---|---|
| 797225 | 2013 | 2/21/2013 | WHEAT FLOUR, BROMATED | Grains and Grain Products | Wheat | IMPORT | Mexico | Non-Detect | 2.3 | 0 |
| 797482 | 2013 | 2/25/2013 | LENTILS | Beans and Legumes | Lentils | IMPORT | Canada | Non-Detect | 1 | 0 |
| 797630 | 2013 | 2/25/2013 | OATMEAL, REGULAR, FRUIT OR SPICE ADDED, QUICK OR INSTANT COOKING | Processed Food Products | Breakfast Foods | IMPORT | Russia | Non-Detect | 1 | 0 |
| 797730 | 2013 | 2/25/2013 | WHEAT, MILLED PRODUCT, N.E.C. | Grains and Grain Products | Wheat | IMPORT | Mexico | Non-Detect | 1 | 0 |
| 798330 | 2013 | 2/28/2013 | CORN, BOLTED MEAL OR FLOUR | Grains and Grain Products | Corn | IMPORT | Italy | Non-Detect | 1 | 0 |
| 798340 | 2013 | 2/28/2013 | CORN, BOLTED MEAL OR FLOUR | Grains and Grain Products | Corn | IMPORT | Italy | Non-Detect | 1 | 0 |
| 798358 | 2013 | 2/28/2013 | BREAKFAST FOOD QUICK OR INSTANT COOKING, N.E.C. | Processed Food Products | Breakfast Foods | IMPORT | Guatemala | Non-Detect | 1 | 0 |
| 798423 | 2013 | 3/1/2013 | OAT FLOUR | Grains and Grain Products | Oats | IMPORT | Canada | Non-Detect | 1 | 0 |
| 743381 | 2013 | 3/4/2013 | MALT, BARLEY | Grains and Grain Products | Barley | DOMESTIC | United States | Non-Detect | 1 | 0 |
| 798607 | 2013 | 3/5/2013 | WHEAT FLOUR, WHOLE | Grains and Grain Products | Wheat | DOMESTIC | United States | Non-Detect | 1 | 0 |
| 798608 | 2013 | 3/5/2013 | WHEAT FLOUR, BROMATED | Grains and Grain Products | Wheat | DOMESTIC | United States | Non-Detect | 1 | 0 |
| 798609 | 2013 | 3/5/2013 | WHEAT FLOUR, WHOLE | Grains and Grain Products | Wheat | DOMESTIC | United States | Non-Detect | 1 | 0 |
| 798610 | 2013 | 3/5/2013 | WHEAT, MILLED (CRUSHED, COARSE GROUND OR CRACKED) | Grains and Grain Products | Wheat | DOMESTIC | United States | Non-Detect | 1 | 0 |
| 799617 | 2013 | 3/6/2013 | MIXED CEREAL, QUICK OR INSTANT COOKING | Processed Food Products | Breakfast Foods | IMPORT | Malaysia | Non-Detect | 1 | 0 |
| 799729 | 2013 | 3/6/2013 | MIXED CEREAL, QUICK OR INSTANT COOKING | Processed Food Products | Breakfast Foods | IMPORT | Malaysia | Non-Detect | 1 | 0 |
| 799732 | 2013 | 3/6/2013 | MIXED CEREAL, QUICK OR INSTANT COOKING | Processed Food Products | Breakfast Foods | IMPORT | Malaysia | Non-Detect | 1 | 0 |
| 782803 | 2013 | 3/8/2013 | WHEAT, WHOLE GRAIN | Grains and Grain Products | Wheat | DOMESTIC | United States | Non-Detect | 0.233 | 0 |
| 782804 | 2013 | 3/8/2013 | WHEAT, WHOLE GRAIN | Grains and Grain Products | Wheat | DOMESTIC | United States | Non-Detect | 0.233 | 0 |
| 782805 | 2013 | 3/12/2013 | WHEAT, WHOLE GRAIN | Grains and Grain Products | Wheat | DOMESTIC | United States | Non-Detect | 0.233 | 0 |
| 782806 | 2013 | 3/12/2013 | WHEAT, WHOLE GRAIN | Grains and Grain Products | Wheat | DOMESTIC | United States | Non-Detect | 0.233 | 0 |
| 789506 | 2013 | 3/13/2013 | OAT CEREAL (BABY) | Baby Food Products | Baby Cereals | DOMESTIC | United States | Non-Detect | 0.233 | 0 |
| 713291 | 2013 | 3/14/2013 | LENTILS, DRIED OR PASTE | Beans and Legumes | Lentils | DOMESTIC | United States | Non-Detect | 0.233 | 0 |
| 798611 | 2013 | 3/14/2013 | WHEAT, MILLED (CRUSHED, COARSE GROUND OR CRACKED) | Grains and Grain Products | Wheat | DOMESTIC | United States | Non-Detect | 1 | 0 |
| 798612 | 2013 | 3/14/2013 | MALT, BARLEY | Grains and Grain Products | Barley | DOMESTIC | United States | Non-Detect | 1 | 0 |
| 802254 | 2013 | 3/15/2013 | BARLEY, WHOLE GRAIN | Grains and Grain Products | Barley | IMPORT | Korea (the Republic of) | Non-Detect | 2.3 | 0 |
| 801369 | 2013 | 3/15/2013 | WHEAT FLOUR, WHOLE | Grains and Grain Products | Wheat | IMPORT | India | Non-Detect | 1 | 0 |
| 802596 | 2013 | 3/20/2013 | OATMEAL, REGULAR, FRUIT OR SPICE ADDED, QUICK OR INSTANT COOKING | Processed Food Products | Breakfast Foods | IMPORT | Canada | Non-Detect | 2.3 | 0 |
| 802552 | 2013 | 3/20/2013 | COFFEE, BEANS | Non-Juice Beverages | Coffee | IMPORT | Vietnam | Non-Detect | 1 | 0 |
| 802734 | 2013 | 3/20/2013 | VEGETABLE SPAGHETTI PRODUCTS | Processed Food Products | Pasta | IMPORT | Italy | Non-Detect | 1 | 0 |
| 792390 | 2013 | 3/21/2013 | BARLEY, WHOLE GRAIN | Grains and Grain Products | Barley | DOMESTIC | United States | Non-Detect | 0.233 | 0 |
| 759824 | 2013 | 3/27/2013 | MALT, BARLEY | Grains and Grain Products | Barley | DOMESTIC | United States | Non-Detect | 0.233 | 0 |
| 759825 | 2013 | 3/27/2013 | MALT, BARLEY | Grains and Grain Products | Barley | IMPORT | United Kingdom | Non-Detect | 0.233 | 0 |
| 780093 | 2013 | 3/28/2013 | WHEAT, WHOLE GRAIN | Grains and Grain Products | Wheat | DOMESTIC | United States | Non-Detect | 2.3 | 0 |
| 804467 | 2013 | 4/1/2013 | WHEAT, WHOLE GRAIN | Grains and Grain Products | Wheat | IMPORT | Canada | Non-Detect | 2.3 | 0 |
| 804464 | 2013 | 4/2/2013 | GARBANZO BEAN | Beans and Legumes | Garbanzo Beans | IMPORT | Canada | Non-Detect | 2.3 | 0 |
| 804595 | 2013 | 4/2/2013 | WHEAT FLOUR, DURUM | Grains and Grain Products | Wheat | IMPORT | India | Non-Detect | 1 | 0 |
| 799976 | 2013 | 4/4/2013 | OAT CEREAL (BABY) | Baby Food Products | Baby Cereals | DOMESTIC | United States | Non-Detect | 0.233 | 0 |
| 744811 | 2013 | 4/9/2013 | CORN, HOMINY GRITS | Grains and Grain Products | Corn | DOMESTIC | Unknown | Non-Detect | 1 | 0 |
| 744812 | 2013 | 4/9/2013 | CORN, HOMINY GRITS | Grains and Grain Products | Corn | DOMESTIC | Unknown | Non-Detect | 1 | 0 |
| 806711 | 2013 | 4/16/2013 | COFFEE, BEANS | Non-Juice Beverages | Coffee | IMPORT | Rwanda | Non-Detect | 2.3 | 0 |
| 807173 | 2013 | 4/17/2013 | WHEAT STARCH SNACKS, FRIED/OIL COOK | Processed Food Products | Other Snack Foods | IMPORT | Mexico | Non-Detect | 0.233 | 0 |
| 808224 | 2013 | 4/24/2013 | COFFEE, BEANS | Non-Juice Beverages | Coffee | IMPORT | Nicaragua | Non-Detect | 2.3 | 0 |
| 809563 | 2013 | 5/2/2013 | WHEAT FLOUR, WHOLE | Grains and Grain Products | Wheat | IMPORT | Canada | Positive | 1 | 35.8 |
| 809055 | 2013 | 5/2/2013 | SOYBEANS, WHOLE GRAIN | Beans and Legumes | Soybeans | DOMESTIC | United States | Non-Detect | 2.3 | 0 |
| 806268 | 2013 | 5/2/2013 | WHEAT FLAKES, PUFFS, KRISPIES, LOOPS, SHREDDED, WHEAT GERM READY TO EAT | Processed Food Products | Breakfast Foods | DOMESTIC | United States | Non-Detect | 0.233 | 0 |
| 809057 | 2013 | 5/3/2013 | SOYBEANS, WHOLE GRAIN | Beans and Legumes | Soybeans | DOMESTIC | United States | Non-Detect | 2.3 | 0 |
| 810406 | 2013 | 5/8/2013 | FLOURS AND MEALS N.E.C. | Grains and Grain Products | Other Grains | IMPORT | India | Non-Detect | 1 | 0 |
| 810350 | 2013 | 5/8/2013 | GRAM FLOUR | Beans and Legumes | Garbanzo Beans | IMPORT | India | Trace | 1 | |
| 810777 | 2013 | 5/13/2013 | COFFEE, BEANS | Non-Juice Beverages | Coffee | IMPORT | Haiti | Non-Detect | 0.233 | 0 |
| 812354 | 2013 | 5/14/2013 | GARBANZO BEAN, DRIED OR PASTE | Beans and Legumes | Garbanzo Beans | IMPORT | India | Positive | 2.7 | 7.82 |
| 811711 | 2013 | 5/15/2013 | FARINA, WHEAT | Grains and Grain Products | Wheat | IMPORT | Canada | Non-Detect | 1 | 0 |
| 811712 | 2013 | 5/15/2013 | WHEAT FLOUR, DURUM | Grains and Grain Products | Wheat | IMPORT | Canada | Non-Detect | 1 | 0 |
| 812315 | 2013 | 5/20/2013 | SPAGHETTI | Processed Food Products | Pasta | IMPORT | Mexico | Non-Detect | 0.233 | 0 |
| 812466 | 2013 | 5/21/2013 | BARLEY, WHOLE GRAIN | Grains and Grain Products | Barley | IMPORT | Canada | Non-Detect | 1 | 0 |
| 812675 | 2013 | 5/22/2013 | COFFEE, BEANS | Non-Juice Beverages | Coffee | IMPORT | Kenya | Positive | 24.9 | 116 |
| 813014 | 2013 | 5/23/2013 | WHEAT FLOUR, ENRICHED (ALL PURPOSE) | Grains and Grain Products | Wheat | IMPORT | India | Non-Detect | 1 | 0 |
| 811739 | 2013 | 5/24/2013 | RAISINS, DRIED OR PASTE | Fruit | Raisins | DOMESTIC | United States | Non-Detect | 1 | 0 |
| 811740 | 2013 | 5/24/2013 | RAISINS, DRIED OR PASTE | Fruit | Raisins | DOMESTIC | United States | Non-Detect | 1 | 0 |
| 813965 | 2013 | 5/30/2013 | PEANUT, SHELLED | Nuts and Edible Seeds | Peanuts | IMPORT | Thailand | Non-Detect | 0.233 | 0 |
| 814114 | 2013 | 5/31/2013 | ENGLISH MUFFINS | Processed Food Products | Other Bakery Products | IMPORT | Canada | Non-Detect | 1 | 0 |
| 814592 | 2013 | 6/4/2013 | WHEAT FLOUR, ENRICHED (ALL PURPOSE) | Grains and Grain Products | Wheat | IMPORT | Mexico | Non-Detect | 0.233 | 0 |
| 814629 | 2013 | 6/4/2013 | VANILLA PLAIN COOKIE, BISCUIT AND WAFER | Processed Food Products | Other Bakery Products | IMPORT | Mexico | Non-Detect | 1 | 0 |
| 814902 | 2013 | 6/5/2013 | WHEAT, WHOLE GRAIN | Grains and Grain Products | Wheat | IMPORT | Canada | Non-Detect | 1 | 0 |
| 815046 | 2013 | 6/5/2013 | WHEAT FLAKES, PUFFS, KRISPIES, LOOPS, SHREDDED, WHEAT GERM READY TO EAT | Processed Food Products | Breakfast Foods | IMPORT | India | Non-Detect | 1 | 0 |
| 815086 | 2013 | 6/6/2013 | OATMEAL, REGULAR, FRUIT OR SPICE ADDED, QUICK OR INSTANT COOKING | Processed Food Products | Breakfast Foods | IMPORT | Canada | Non-Detect | 1 | 0 |
| 815114 | 2013 | 6/6/2013 | OATS, WHOLE GRAIN | Grains and Grain Products | Oats | IMPORT | Canada | Non-Detect | 1 | 0 |
| 734892 | 2013 | 6/10/2013 | MALT, BARLEY | Grains and Grain Products | Barley | IMPORT | Unknown | Non-Detect | 2.3 | 0 |
| 734893 | 2013 | 6/10/2013 | MALT, BARLEY | Grains and Grain Products | Barley | IMPORT | Unknown | Trace | 2.3 | |
| 734894 | 2013 | 6/11/2013 | MALT, BARLEY | Grains and Grain Products | Barley | IMPORT | Germany | Non-Detect | 2.3 | 0 |
| 734895 | 2013 | 6/11/2013 | MALT, BARLEY | Grains and Grain Products | Barley | IMPORT | Germany | Non-Detect | 2.3 | 0 |
| 783686 | 2013 | 6/13/2013 | WHEAT, WHOLE GRAIN | Grains and Grain Products | Wheat | DOMESTIC | United States | Non-Detect | 2.3 | 0 |
| 816174 | 2013 | 6/13/2013 | CEREAL PREPARATIONS NOT ELSEWHERE MENTIONED, N.E.C. | Processed Food Products | Breakfast Foods | IMPORT | Canada | Non-Detect | 2.3 | 0 |
| 816738 | 2013 | 6/18/2013 | KIDNEY BEAN, DRIED OR PASTE | Beans and Legumes | Other Beans and Legumes | IMPORT | Canada | Non-Detect | 0.233 | 0 |
| 817628 | 2013 | 6/24/2013 | OAT FLAKES, ROLLED OATS, PUFFS, KRISPIES, LOOPS READY TO EAT | Processed Food Products | Breakfast Foods | IMPORT | Canada | Non-Detect | 1 | 0 |
| 763612 | 2013 | 6/25/2013 | OATMEAL, REGULAR, FRUIT OR SPICE ADDED, QUICK OR INSTANT COOKING | Processed Food Products | Breakfast Foods | DOMESTIC | United States | Non-Detect | 2.3 | 0 |
| 818105 | 2013 | 6/25/2013 | BEANS, CORN, AND PEA, N.E.C. (VEGETABLE) | Beans and Legumes | Other Beans and Legumes | IMPORT | Mexico | Non-Detect | 0.233 | 0 |
| 818522 | 2013 | 6/27/2013 | WHEAT FLOUR, WHOLE | Grains and Grain Products | Wheat | IMPORT | United Arab Emirates | Non-Detect | 1 | 0 |
| 816995 | 2013 | 7/1/2013 | MALT, BARLEY | Grains and Grain Products | Barley | IMPORT | United Kingdom | Non-Detect | 2.3 | 0 |
| 816996 | 2013 | 7/1/2013 | MALT, BARLEY | Grains and Grain Products | Barley | IMPORT | United Kingdom | Non-Detect | 2.3 | 0 |
| 818840 | 2013 | 7/1/2013 | COFFEE, BEANS | Non-Juice Beverages | Coffee | IMPORT | Haiti | Non-Detect | 0.233 | 0 |
| 816997 | 2013 | 7/2/2013 | MALT, BARLEY | Grains and Grain Products | Barley | IMPORT | United States | Non-Detect | 2.3 | 0 |
| 816998 | 2013 | 7/2/2013 | MALT, BARLEY | Grains and Grain Products | Barley | IMPORT | United States | Non-Detect | 2.3 | 0 |
| 819094 | 2013 | 7/2/2013 | WHEAT FLOUR, WHOLE | Grains and Grain Products | Wheat | IMPORT | India | Non-Detect | 2.3 | 0 |
| 819178 | 2013 | 7/2/2013 | WHEAT FLOUR, WHOLE | Grains and Grain Products | Wheat | IMPORT | India | Non-Detect | 2.3 | 0 |
| 819183 | 2013 | 7/2/2013 | WHEAT FLOUR, WHOLE | Grains and Grain Products | Wheat | IMPORT | India | Non-Detect | 2.3 | 0 |
| 819293 | 2013 | 7/3/2013 | KIDNEY BEAN | Beans and Legumes | Other Beans and Legumes | IMPORT | Nicaragua | Non-Detect | 0.233 | 0 |
| 819376 | 2013 | 7/3/2013 | SOYBEANS (SEE INDUSTRY 37 FOR 'BEAN CURD') | Beans and Legumes | Soybeans | IMPORT | China | Non-Detect | 1 | 0 |
| 809413 | 2013 | 7/10/2013 | CORN, HOMINY GRITS | Grains and Grain Products | Corn | DOMESTIC | United States | Non-Detect | 1 | 0 |
| 820224 | 2013 | 7/11/2013 | BEANS, CORN, AND PEA, N.E.C. (VEGETABLE) | Beans and Legumes | Other Beans and Legumes | IMPORT | Mexico | Non-Detect | 0.233 | 0 |

| | | | | | | | | | | |
|---|---|---|---|---|---|---|---|---|---|---|
| 820384 | 2013 | 7/12/2013 | GARBANZO BEAN | Beans and Legumes | Garbanzo Beans | IMPORT | Canada | Non-Detect | 2.3 | 0 |
| 820401 | 2013 | 7/12/2013 | FLOR DE MAYO BEAN, DRIED OR PASTE | Beans and Legumes | Other Beans and Legumes | IMPORT | Mexico | Non-Detect | 0.233 | 0 |
| 821369 | 2013 | 7/15/2013 | RED BEAN, DRIED OR PASTE | Beans and Legumes | Other Beans and Legumes | IMPORT | Guatemala | Non-Detect | 0.233 | 0 |
| 820735 | 2013 | 7/16/2013 | COFFEE, BEANS | Non-Juice Beverages | Coffee | IMPORT | Honduras | Non-Detect | 0.233 | 0 |
| 813513 | 2013 | 7/17/2013 | WHEAT, WHOLE GRAIN | Grains and Grain Products | Wheat | DOMESTIC | United States | Non-Detect | 2.3 | 0 |
| 820917 | 2013 | 7/17/2013 | VANILLA PLAIN COOKIE, BISCUIT AND WAFER | Processed Food Products | Other Bakery Products | IMPORT | Mexico | Non-Detect | 0.233 | 0 |
| 821208 | 2013 | 7/18/2013 | WHEAT FLOUR, BROMATED | Grains and Grain Products | Wheat | IMPORT | Korea (the Republic of) | Non-Detect | 2.3 | 0 |
| 821400 | 2013 | 7/23/2013 | SOYBEANS, WHOLE GRAIN | Beans and Legumes | Soybeans | DOMESTIC | United States | Non-Detect | 2.3 | 0 |
| 821402 | 2013 | 7/23/2013 | WHEAT, WHOLE GRAIN | Grains and Grain Products | Wheat | DOMESTIC | United States | Non-Detect | 2.3 | 0 |
| 821408 | 2013 | 7/23/2013 | WHEAT, WHOLE GRAIN | Grains and Grain Products | Wheat | DOMESTIC | United States | Non-Detect | 2.3 | 0 |
| 821409 | 2013 | 7/23/2013 | WHEAT, WHOLE GRAIN | Grains and Grain Products | Wheat | DOMESTIC | United States | Non-Detect | 2.3 | 0 |
| 821646 | 2013 | 7/23/2013 | CORN CHIPS, FRIED | Processed Food Products | Other Corn Products | IMPORT | Philippines | Non-Detect | 1 | 0 |
| 821406 | 2013 | 7/23/2013 | WHEAT, WHOLE GRAIN | Grains and Grain Products | Wheat | DOMESTIC | United States | Positive | 2.3 | 2.9 |
| 777381 | 2013 | 7/24/2013 | SOYBEANS (EDIBLE SEED) | Beans and Legumes | Soybeans | DOMESTIC | United States | Non-Detect | 2.4 | 0 |
| 822605 | 2013 | 7/25/2013 | WHEAT FLAKES, PUFFS, KRISPIES, LOOPS, SHREDDED, WHEAT GERM READY TO EAT | Processed Food Products | Breakfast Foods | DOMESTIC | United States | Non-Detect | 2.3 | 0 |
| 822527 | 2013 | 7/29/2013 | SOYBEANS (SEE INDUSTRY 37 FOR BEAN CURD), DRIED OR PASTE | Beans and Legumes | Soybeans | IMPORT | Korea (the Republic of) | Non-Detect | 1 | 0 |
| 822687 | 2013 | 7/29/2013 | WHEAT FLOUR, WHOLE | Grains and Grain Products | Wheat | IMPORT | Sri Lanka | Non-Detect | 1 | 0 |
| 823108 | 2013 | 7/31/2013 | WHEAT, MILLED (CRUSHED, COARSE GROUND OR CRACKED) | Grains and Grain Products | Wheat | IMPORT | Turkey | Non-Detect | 2.3 | 0 |
| 823114 | 2013 | 7/31/2013 | WHEAT, WHOLE GRAIN | Grains and Grain Products | Wheat | IMPORT | Turkey | Non-Detect | 2.3 | 0 |
| 823462 | 2013 | 8/1/2013 | OAT FLAKES, ROLLED OATS, PUFFS, KRISPIES, LOOPS READY TO EAT | Processed Food Products | Breakfast Foods | IMPORT | Germany | Non-Detect | 2.3 | 0 |
| 823529 | 2013 | 8/1/2013 | MIXED CEREAL FLAKES, SHREDDED AND OTHER FORMS READY TO EAT | Processed Food Products | Breakfast Foods | IMPORT | Germany | Non-Detect | 2.3 | 0 |
| 823534 | 2013 | 8/1/2013 | MIXED CEREAL FLAKES, SHREDDED AND OTHER FORMS READY TO EAT | Processed Food Products | Breakfast Foods | IMPORT | Poland | Non-Detect | 2.3 | 0 |
| 823190 | 2013 | 8/1/2013 | COFFEE, BEANS | Non-Juice Beverages | Coffee | IMPORT | Honduras | Non-Detect | 1 | 0 |
| 823723 | 2013 | 8/2/2013 | MILLED GRAIN PRODUCTS, N.E.C. | Grains and Grain Products | Other Grains | IMPORT | China | Non-Detect | 1 | 0 |
| 823836 | 2013 | 8/2/2013 | WHOLE WHEAT BREAD/ROLLS/BUNS | Processed Food Products | Bread | IMPORT | Taiwan | Non-Detect | 1 | 0 |
| 823690 | 2013 | 8/5/2013 | COFFEE, BEANS | Non-Juice Beverages | Coffee | IMPORT | Brazil | Non-Detect | 1 | 0 |
| 823716 | 2013 | 8/5/2013 | COFFEE, BEANS | Non-Juice Beverages | Coffee | IMPORT | Brazil | Non-Detect | 1 | 0 |
| 825036 | 2013 | 8/6/2013 | LENTILS, DRIED OR PASTE | Beans and Legumes | Lentils | IMPORT | India | Non-Detect | 2.3 | 0 |
| 825044 | 2013 | 8/6/2013 | LENTILS, DRIED OR PASTE | Beans and Legumes | Lentils | IMPORT | United Arab Emirates | Non-Detect | 2.3 | 0 |
| 823162 | 2013 | 8/8/2013 | CORN GRITS, BREWERS ENRICHED WHITE OR YELLOW CORN GRITS, CORN MEAL MUSH, QUICK OR INSTANT COOKING | Processed Food Products | Other Corn Products | DOMESTIC | United States | Non-Detect | 2.3 | 0 |
| 682011 | 2013 | 8/9/2013 | WHEAT FLOUR, GLUTEN | Grains and Grain Products | Wheat | DOMESTIC | United States | Non-Detect | 0.233 | 0 |
| 788112 | 2013 | 8/12/2013 | WHEAT FLOUR, GLUTEN | Grains and Grain Products | Wheat | DOMESTIC | United States | Non-Detect | 2.3 | 0 |
| 825401 | 2013 | 8/12/2013 | BLACKEYE PEAS, DRIED OR PASTE | Beans and Legumes | Blackeye Peas | IMPORT | Brazil | Non-Detect | 2.3 | 0 |
| 825408 | 2013 | 8/12/2013 | LENTILS, DRIED OR PASTE | Beans and Legumes | Lentils | IMPORT | Canada | Non-Detect | 2.3 | 0 |
| 825411 | 2013 | 8/12/2013 | CHANA DAL, DRIED OR PASTE | Beans and Legumes | Garbanzo Beans | IMPORT | Australia | Non-Detect | 2.3 | 0 |
| 825415 | 2013 | 8/12/2013 | CHANA DAL, DRIED OR PASTE | Beans and Legumes | Garbanzo Beans | IMPORT | Australia | Non-Detect | 2.3 | 0 |
| 825428 | 2013 | 8/12/2013 | KIDNEY BEAN, DRIED OR PASTE | Beans and Legumes | Other Beans and Legumes | IMPORT | Canada | Non-Detect | 2.3 | 0 |
| 825432 | 2013 | 8/12/2013 | PEA, DRIED OR PASTE | Beans and Legumes | Peas | IMPORT | Canada | Trace | 2.8 | |
| 788114 | 2013 | 8/13/2013 | NON STANDARD BREAD, ROLLS, BUNS, ETC PREPARED DRY MIX WITH MILK OR EGG | Processed Food Products | Bread | DOMESTIC | United States | Non-Detect | 2.3 | 0 |
| 825249 | 2013 | 8/13/2013 | OATS, WHOLE GRAIN | Grains and Grain Products | Oats | IMPORT | Canada | Non-Detect | 2.3 | 0 |
| 825318 | 2013 | 8/13/2013 | KIDNEY BEAN | Beans and Legumes | Other Beans and Legumes | IMPORT | Canada | Non-Detect | 2.3 | 0 |
| 825320 | 2013 | 8/13/2013 | MUNG BEAN, DRIED OR PASTE | Beans and Legumes | Other Beans and Legumes | IMPORT | Australia | Non-Detect | 2.3 | 0 |
| 825323 | 2013 | 8/13/2013 | PIGEON PEAS | Beans and Legumes | Peas | IMPORT | India | Non-Detect | 2.3 | 0 |
| 825327 | 2013 | 8/13/2013 | ADZUKI BEANS, DRIED OR PASTE | Beans and Legumes | Other Beans and Legumes | IMPORT | India | Non-Detect | 2.3 | 0 |
| 825375 | 2013 | 8/13/2013 | BLACK EYE BEANS | Beans and Legumes | Blackeye Peas | IMPORT | Canada | Non-Detect | 2.3 | 0 |
| 825364 | 2013 | 8/13/2013 | BLACK EYE BEANS | Beans and Legumes | Blackeye Peas | IMPORT | Canada | Positive | 2.5 | 3.6 |
| 825710 | 2013 | 8/14/2013 | BARLEY, WHOLE GRAIN | Grains and Grain Products | Barley | IMPORT | Canada | Positive | 2.6 | 10.8 |
| 783696 | 2013 | 8/15/2013 | MALT, BARLEY | Grains and Grain Products | Barley | DOMESTIC | United States | Non-Detect | 2.3 | 0 |
| 825698 | 2013 | 8/15/2013 | COFFEE, BEANS | Non-Juice Beverages | Coffee | IMPORT | Brazil | Non-Detect | 2.3 | 0 |
| 825847 | 2013 | 8/15/2013 | PEA, DRIED OR PASTE | Beans and Legumes | Peas | IMPORT | Canada | Non-Detect | 2.3 | 0 |
| 825590 | 2013 | 8/15/2013 | OATS, WHOLE GRAIN | Grains and Grain Products | Oats | IMPORT | Canada | Positive | 2.6 | 10 |
| 825718 | 2013 | 8/15/2013 | OATS, WHOLE GRAIN | Grains and Grain Products | Oats | IMPORT | Canada | Trace | 2.3 | |
| 826043 | 2013 | 8/16/2013 | RYE, WHOLE GRAIN | Grains and Grain Products | Rye | IMPORT | Canada | Non-Detect | 2.3 | 0 |
| 826040 | 2013 | 8/19/2013 | FAVA BEAN | Beans and Legumes | Other Beans and Legumes | IMPORT | Canada | Non-Detect | 2.3 | 0 |
| 826348 | 2013 | 8/19/2013 | SOYBEANS (SEE INDUSTRY 37 FOR 'BEAN CURD') | Beans and Legumes | Soybeans | IMPORT | Canada | Non-Detect | 2.3 | 0 |
| 826106 | 2013 | 8/19/2013 | OATS, WHOLE GRAIN | Grains and Grain Products | Oats | IMPORT | Canada | Positive | 2.3 | 5.6 |
| 826326 | 2013 | 8/19/2013 | BARLEY, WHOLE GRAIN | Grains and Grain Products | Barley | IMPORT | Canada | Positive | 2.3 | 44 |
| 826301 | 2013 | 8/20/2013 | BARLEY, WHOLE GRAIN | Grains and Grain Products | Barley | IMPORT | Canada | Non-Detect | 2.3 | 0 |
| 826417 | 2013 | 8/20/2013 | SOYBEANS, WHOLE GRAIN | Beans and Legumes | Soybeans | IMPORT | Canada | Non-Detect | 2.3 | 0 |
| 826672 | 2013 | 8/21/2013 | MALT, BARLEY | Grains and Grain Products | Barley | IMPORT | Canada | Non-Detect | 2.3 | 0 |
| 826697 | 2013 | 8/21/2013 | SOYBEANS (SEE INDUSTRY 37 FOR 'BEAN CURD') | Beans and Legumes | Soybeans | IMPORT | Canada | Non-Detect | 2.3 | 0 |
| 826569 | 2013 | 8/21/2013 | BEAN, CORN, PEA, DRIED OR PASTE, N.E.C. (VEGETABLE) | Beans and Legumes | Other Beans and Legumes | IMPORT | Mexico | Non-Detect | 0.233 | 0 |
| 826578 | 2013 | 8/21/2013 | LENTILS, DRIED OR PASTE | Beans and Legumes | Lentils | IMPORT | Mexico | Non-Detect | 0.233 | 0 |
| 826701 | 2013 | 8/22/2013 | SOYBEANS (SEE INDUSTRY 37 FOR 'BEAN CURD') | Beans and Legumes | Soybeans | IMPORT | Canada | Non-Detect | 2.3 | 0 |
| 826703 | 2013 | 8/22/2013 | GARBANZO BEAN, DRIED OR PASTE | Beans and Legumes | Garbanzo Beans | IMPORT | Spain | Trace | 2.3 | |
| 722447 | 2013 | 8/26/2013 | WHEAT, WHOLE GRAIN | Grains and Grain Products | Wheat | DOMESTIC | United States | Non-Detect | 0.233 | 0 |
| 827287 | 2013 | 8/26/2013 | CORN, DRIED OR PASTE | Grains and Grain Products | Corn | IMPORT | China | Trace | 1 | |
| 827537 | 2013 | 8/27/2013 | OATS, WHOLE GRAIN | Grains and Grain Products | Oats | IMPORT | Latvia | Non-Detect | 2.3 | 0 |
| 827317 | 2013 | 8/27/2013 | PERUANO BEANS, DRIED OR PASTE | Beans and Legumes | Other Beans and Legumes | IMPORT | Mexico | Non-Detect | 0.233 | 0 |
| 827257 | 2013 | 8/28/2013 | BUCKWHEAT, WHOLE GRAIN | Grains and Grain Products | Buckwheat | DOMESTIC | United States | Non-Detect | 0.233 | 0 |
| 828062 | 2013 | 8/29/2013 | WHEAT CEREAL (BABY) | Baby Food Products | Baby Cereals | IMPORT | United Kingdom | Non-Detect | 2.3 | 0 |
| 828067 | 2013 | 8/29/2013 | WHEAT CEREAL (BABY) | Baby Food Products | Baby Cereals | IMPORT | United Kingdom | Non-Detect | 2.3 | 0 |
| 827258 | 2013 | 8/29/2013 | WHEAT FLAKES, PUFFS, KRISPIES, LOOPS, SHREDDED, WHEAT GERM READY TO EAT | Processed Food Products | Breakfast Foods | DOMESTIC | United States | Non-Detect | 0.233 | 0 |
| 827259 | 2013 | 8/29/2013 | WHEAT FLAKES, PUFFS, KRISPIES, LOOPS, SHREDDED, WHEAT GERM READY TO EAT | Processed Food Products | Breakfast Foods | DOMESTIC | United States | Non-Detect | 0.233 | 0 |
| 827260 | 2013 | 8/29/2013 | WHEAT FLAKES, PUFFS, KRISPIES, LOOPS, SHREDDED, WHEAT GERM READY TO EAT | Processed Food Products | Breakfast Foods | DOMESTIC | United States | Non-Detect | 0.233 | 0 |
| 828164 | 2013 | 8/30/2013 | COFFEE, BEANS | Non-Juice Beverages | Coffee | IMPORT | Vietnam | Non-Detect | 0.233 | 0 |
| 634613 | 2013 | 9/4/2013 | CORN GRITS, BREWERS ENRICHED WHITE OR YELLOW CORN GRITS, CORN MEAL MUSH, QUICK OR INSTANT COOKING | Processed Food Products | Other Corn Products | DOMESTIC | United States | Non-Detect | 1 | 0 |
| 829269 | 2013 | 9/4/2013 | COFFEE, BEANS | Non-Juice Beverages | Coffee | IMPORT | Saudi Arabia | Positive | 2.3 | 21.3 |
| 634614 | 2013 | 9/5/2013 | CORN, ENRICHED MEAL | Grains and Grain Products | Corn | DOMESTIC | United States | Non-Detect | 1 | 0 |
| 828881 | 2013 | 9/5/2013 | BEAN, CORN, PEA, DRIED OR PASTE, N.E.C. (VEGETABLE) | Beans and Legumes | Other Beans and Legumes | IMPORT | Mexico | Positive | 5 | 25.2 |
| 828918 | 2013 | 9/6/2013 | WHEAT, WHOLE GRAIN | Grains and Grain Products | Wheat | IMPORT | Canada | Non-Detect | 1 | 0 |
| 829416 | 2013 | 9/9/2013 | COFFEE, BEANS | Non-Juice Beverages | Coffee | IMPORT | Jamaica | Non-Detect | 2.2 | 0 |
| 829682 | 2013 | 9/10/2013 | GARBANZO BEAN, DRIED OR PASTE | Beans and Legumes | Garbanzo Beans | IMPORT | Mexico | Non-Detect | 0.233 | 0 |
| 780876 | 2013 | 9/11/2013 | COFFEE, BEANS | Non-Juice Beverages | Coffee | IMPORT | Brazil | Non-Detect | 2.3 | 0 |
| 780877 | 2013 | 9/11/2013 | COFFEE, BEANS | Non-Juice Beverages | Coffee | IMPORT | Colombia | Non-Detect | 2.3 | 0 |
| 780878 | 2013 | 9/11/2013 | COFFEE, BEANS | Non-Juice Beverages | Coffee | IMPORT | Ethiopia | Non-Detect | 2.3 | 0 |
| 780879 | 2013 | 9/11/2013 | COFFEE, BEANS | Non-Juice Beverages | Coffee | IMPORT | Honduras | Non-Detect | 2.3 | 0 |
| 780880 | 2013 | 9/11/2013 | COFFEE, BEANS | Non-Juice Beverages | Coffee | IMPORT | Mexico | Non-Detect | 2.3 | 0 |
| 828040 | 2013 | 9/11/2013 | WHEAT FLOUR, ENRICHED (ALL PURPOSE) | Grains and Grain Products | Wheat | DOMESTIC | United States | Non-Detect | 2.3 | 0 |

| | | | | | | | | | | |
|---|---|---|---|---|---|---|---|---|---|---|
| 830313 | 2013 | 9/12/2013 | WHEAT FLAKES, PUFFS, KRISPIES, LOOPS, SHREDDED, WHEAT GERM READY TO EAT | Processed Food Products | Breakfast Foods | IMPORT | Taiwan | Non-Detect | 1 | 0 |
| 695929 | 2013 | 9/16/2013 | WHEAT FLOUR, WHOLE | Grains and Grain Products | Wheat | DOMESTIC | United States | Non-Detect | 2.3 | 0 |
| 695931 | 2013 | 9/16/2013 | WHEAT FLOUR, WHOLE | Grains and Grain Products | Wheat | DOMESTIC | United States | Non-Detect | 2.3 | 0 |
| 695932 | 2013 | 9/16/2013 | WHEAT FLOUR, WHOLE | Grains and Grain Products | Wheat | DOMESTIC | United States | Non-Detect | 2.3 | 0 |
| 830250 | 2013 | 9/16/2013 | WHEAT FLOUR, PLAIN | Grains and Grain Products | Wheat | DOMESTIC | United States | Non-Detect | 2.3 | 0 |
| 830251 | 2013 | 9/16/2013 | WHEAT FLOUR, N.E.C. | Grains and Grain Products | Wheat | DOMESTIC | United States | Non-Detect | 2.3 | 0 |
| 830253 | 2013 | 9/16/2013 | WHEAT FLOUR, PLAIN | Grains and Grain Products | Wheat | DOMESTIC | United States | Non-Detect | 2.3 | 0 |
| 695934 | 2013 | 9/17/2013 | WHEAT FLOUR, WHOLE | Grains and Grain Products | Wheat | DOMESTIC | United States | Non-Detect | 2.3 | 0 |
| 831038 | 2013 | 9/19/2013 | WHEAT FLOUR, WHOLE | Grains and Grain Products | Wheat | IMPORT | India | Non-Detect | 2.3 | 0 |
| 831045 | 2013 | 9/19/2013 | CREAM WHEAT, QUICK OR INSTANT COOKING | Processed Food Products | Breakfast Foods | IMPORT | India | Non-Detect | 2.3 | 0 |
| 831359 | 2013 | 9/20/2013 | WHEAT FLOUR, WHOLE | Grains and Grain Products | Wheat | IMPORT | India | Non-Detect | 2.3 | 0 |
| 831361 | 2013 | 9/20/2013 | WHEAT FLOUR, WHOLE | Grains and Grain Products | Wheat | IMPORT | India | Non-Detect | 2.3 | 0 |
| 831416 | 2013 | 9/20/2013 | WHEAT, WHOLE GRAIN | Grains and Grain Products | Wheat | DOMESTIC | United States | Non-Detect | 2.3 | 0 |
| 831499 | 2013 | 9/20/2013 | WHEAT, WHOLE GRAIN | Grains and Grain Products | Wheat | DOMESTIC | United States | Non-Detect | 2.3 | 0 |
| 832158 | 2013 | 9/20/2013 | PINTO BEAN, DRIED OR PASTE | Beans and Legumes | Pinto Beans | DOMESTIC | United States | Non-Detect | 2.3 | 0 |
| 801594 | 2013 | 9/20/2013 | OAT FLAKES, ROLLED OATS, PUFFS, KRISPIES, LOOPS READY TO EAT | Processed Food Products | Breakfast Foods | DOMESTIC | United States | Non-Detect | 1 | 0 |
| 829151 | 2013 | 9/20/2013 | WHEAT, WHOLE GRAIN | Grains and Grain Products | Wheat | DOMESTIC | Unknown | Non-Detect | 1 | 0 |
| 829152 | 2013 | 9/20/2013 | WHEAT, WHOLE GRAIN | Grains and Grain Products | Wheat | DOMESTIC | Unknown | Non-Detect | 1 | 0 |
| 832237 | 2013 | 9/23/2013 | NAVY (WHITE) BEANS (BAKED BEANS, PORK AND BEANS), DRIED OR PASTE | Beans and Legumes | Other Beans and Legumes | DOMESTIC | United States | Non-Detect | 2.3 | 0 |
| 832279 | 2013 | 9/23/2013 | WHEAT, WHOLE GRAIN | Grains and Grain Products | Wheat | DOMESTIC | United States | Non-Detect | 2.3 | 0 |
| 832299 | 2013 | 9/23/2013 | WHEAT, WHOLE GRAIN | Grains and Grain Products | Wheat | DOMESTIC | United States | Non-Detect | 2.6 | 0 |
| 832784 | 2013 | 9/23/2013 | PINTO BEAN | Beans and Legumes | Pinto Beans | DOMESTIC | United States | Non-Detect | 2.3 | 0 |
| 832806 | 2013 | 9/23/2013 | NAVY (WHITE) BEANS (BAKED BEANS, PORK AND BEANS), DRIED OR PASTE | Beans and Legumes | Other Beans and Legumes | DOMESTIC | United States | Non-Detect | 2.3 | 0 |
| 832807 | 2013 | 9/23/2013 | WHEAT, WHOLE GRAIN | Grains and Grain Products | Wheat | DOMESTIC | United States | Non-Detect | 2.3 | 0 |
| 751317 | 2013 | 9/23/2013 | WHEAT FLAKES, PUFFS, KRISPIES, LOOPS, SHREDDED, WHEAT GERM READY TO EAT | Processed Food Products | Breakfast Foods | DOMESTIC | United States | Non-Detect | 1 | 0 |
| 832797 | 2013 | 9/23/2013 | WHEAT, WHOLE GRAIN | Grains and Grain Products | Wheat | DOMESTIC | United States | Positive | 2.6 | 32.7 |
| 770083 | 2013 | 9/24/2013 | PEAS | Beans and Legumes | Peas | DOMESTIC | Unknown | Non-Detect | 0.233 | 0 |
| 770084 | 2013 | 9/24/2013 | PINTO BEAN | Beans and Legumes | Pinto Beans | DOMESTIC | Unknown | Non-Detect | 0.233 | 0 |
| 827026 | 2013 | 9/24/2013 | WHEAT, WHOLE GRAIN | Grains and Grain Products | Wheat | DOMESTIC | United States | Non-Detect | 0.233 | 0 |
| 751318 | 2013 | 9/24/2013 | RAISINS, DRIED OR PASTE | Fruit | Raisins | DOMESTIC | United States | Non-Detect | 1 | 0 |
| 794116 | 2013 | 9/25/2013 | POPCORN (NOT POPPED), BULK, WHOLE GRAIN | Grains and Grain Products | Corn | DOMESTIC | Unknown | Non-Detect | 1 | 0 |
| 827318 | 2013 | 9/26/2013 | RYE FLOUR | Grains and Grain Products | Rye | DOMESTIC | United States | Non-Detect | 2.3 | 0 |
| 827319 | 2013 | 9/26/2013 | WHEAT FLOUR, WHOLE DURUM | Grains and Grain Products | Wheat | DOMESTIC | United States | Non-Detect | 2.3 | 0 |

## FY2014 Data

| Sample Number | Fiscal Year | Collection Date | Product Name | Food Category | Food Subcategory | Origin Type | Country of Origin | Non-Detect, Trace, or Positive (>LOQ) | LOQ (ppb) | Amount found (ppb) |
|---|---|---|---|---|---|---|---|---|---|---|
| 833749 | 2014 | 10/29/2013 | OATMEAL COOKIE, BISCUIT, WAFER DOUGH | Processed Food Products | Other Bakery Products | IMPORT | Canada | Non-Detect | 2.3 | 0 |
| 833738 | 2014 | 10/29/2013 | OAT FLAKES, ROLLED OATS, PUFFS, KRISPIES, LOOPS READY TO EAT | Processed Food Products | Breakfast Foods | IMPORT | Canada | Non-Detect | 1 | 0 |
| 834086 | 2014 | 10/31/2013 | OAT FLAKES, ROLLED OATS, PUFFS, KRISPIES, LOOPS READY TO EAT | Processed Food Products | Breakfast Foods | IMPORT | Canada | Non-Detect | 1 | 0 |
| 834245 | 2014 | 10/31/2013 | WHEAT, WHOLE GRAIN | Grains and Grain Products | Wheat | IMPORT | Italy | Non-Detect | 1 | 0 |
| 834286 | 2014 | 11/1/2013 | OTHER BAKERY PRODUCTS, N.E.C. | Processed Food Products | Other Bakery Products | IMPORT | Korea (the Republic of) | Trace | 1 | |
| 834545 | 2014 | 11/4/2013 | WHEAT FLOUR, PLAIN | Grains and Grain Products | Wheat | IMPORT | Korea (the Republic of) | Non-Detect | 1 | 0 |
| 834623 | 2014 | 11/5/2013 | FLOURS AND MEALS N.E.C. | Grains and Grain Products | Other Grains | IMPORT | Mexico | Non-Detect | 0.233 | 0 |
| 829370 | 2014 | 11/6/2013 | WHEAT, WHOLE GRAIN | Grains and Grain Products | Wheat | DOMESTIC | United States | Trace | 5 | |
| 835470 | 2014 | 11/7/2013 | WHEAT FLOUR, WHOLE | Grains and Grain Products | Wheat | IMPORT | India | Non-Detect | 2.3 | 0 |
| 835283 | 2014 | 11/7/2013 | COFFEE, BEANS | Non-Juice Beverages | Coffee | IMPORT | Colombia | Non-Detect | 0.233 | 0 |
| 835500 | 2014 | 11/7/2013 | GRANOLA BARS | Processed Food Products | Other Processed Foods | IMPORT | Mexico | Non-Detect | 0.233 | 0 |
| 835287 | 2014 | 11/7/2013 | CORN CHIPS, FRIED | Processed Food Products | Other Corn Products | IMPORT | Korea (the Republic of) | Non-Detect | 1 | 0 |
| 835441 | 2014 | 11/7/2013 | CORN CHIPS, FRIED | Processed Food Products | Other Corn Products | IMPORT | Korea (the Republic of) | Non-Detect | 1 | 0 |
| 835646 | 2014 | 11/7/2013 | PEANUT, SHELLED | Nuts and Edible Seeds | Peanuts | IMPORT | Indonesia | Non-Detect | 1 | 0 |
| 835712 | 2014 | 11/12/2013 | SPAGHETTI | Processed Food Products | Pasta | IMPORT | Italy | Non-Detect | 1 | 0 |
| 835716 | 2014 | 11/12/2013 | MACARONI | Processed Food Products | Pasta | IMPORT | Italy | Non-Detect | 1 | 0 |
| 835860 | 2014 | 11/12/2013 | COFFEE, BEANS | Non-Juice Beverages | Coffee | IMPORT | Vietnam | Non-Detect | 1 | 0 |
| 836517 | 2014 | 11/12/2013 | CORN CHIPS, FRIED | Processed Food Products | Other Corn Products | IMPORT | Philippines | Non-Detect | 1 | 0 |
| 836570 | 2014 | 11/15/2013 | WHEAT MEAL | Grains and Grain Products | Wheat | IMPORT | Guatemala | Non-Detect | 2.3 | 0 |
| 836669 | 2014 | 11/15/2013 | MUNG BEAN, DRIED OR PASTE | Beans and Legumes | Other Beans and Legumes | IMPORT | Thailand | Non-Detect | 2.3 | 0 |
| 836675 | 2014 | 11/15/2013 | CHANA DAL | Beans and Legumes | Garbanzo Beans | IMPORT | Canada | Non-Detect | 2.3 | 0 |
| 836776 | 2014 | 11/15/2013 | ADZUKI BEANS | Beans and Legumes | Other Beans and Legumes | IMPORT | Canada | Non-Detect | 2.3 | 0 |
| 837101 | 2014 | 11/15/2013 | BLACKEYE PEAS, DRIED OR PASTE | Beans and Legumes | Blackeye Peas | IMPORT | Canada | Non-Detect | 2.3 | 0 |
| 837326 | 2014 | 11/15/2013 | KIDNEY BEAN | Beans and Legumes | Other Beans and Legumes | IMPORT | Canada | Non-Detect | 2.3 | 0 |
| 837338 | 2014 | 11/15/2013 | BLACKEYE PEAS, DRIED OR PASTE | Beans and Legumes | Blackeye Peas | IMPORT | Peru | Non-Detect | 2.3 | 0 |
| 836455 | 2014 | 11/15/2013 | COFFEE, BEANS | Non-Juice Beverages | Coffee | IMPORT | Colombia | Non-Detect | 0.233 | 0 |
| 836561 | 2014 | 11/15/2013 | RAISINS (DRIED GRAPES) (BERRY) | Fruit | Raisins | IMPORT | Argentina | Positive | 5 | 20.9 |
| 836688 | 2014 | 11/18/2013 | BEAN, DRIED OR PASTE, N.E.C. (VEGETABLE) | Beans and Legumes | Other Beans and Legumes | IMPORT | Canada | Non-Detect | 2.3 | 0 |
| 836690 | 2014 | 11/18/2013 | OATS, WHOLE GRAIN | Grains and Grain Products | Oats | IMPORT | Canada | Non-Detect | 2.3 | 0 |
| 836826 | 2014 | 11/18/2013 | BLACK EYE BEANS | Beans and Legumes | Blackeye Peas | IMPORT | Canada | Non-Detect | 2.3 | 0 |
| 836882 | 2014 | 11/18/2013 | SOYBEANS, WHOLE GRAIN | Beans and Legumes | Soybeans | IMPORT | Canada | Non-Detect | 2.3 | 0 |
| 836998 | 2014 | 11/18/2013 | SOYBEANS, WHOLE GRAIN | Beans and Legumes | Soybeans | IMPORT | Canada | Non-Detect | 2.3 | 0 |
| 836953 | 2014 | 11/18/2013 | WHEAT FLOUR, WHOLE | Grains and Grain Products | Wheat | IMPORT | India | Non-Detect | 1 | 0 |
| 837030 | 2014 | 11/19/2013 | RYE, WHOLE GRAIN | Grains and Grain Products | Rye | IMPORT | Canada | Non-Detect | 2.3 | 0 |
| 837195 | 2014 | 11/19/2013 | LENTILS, DRIED OR PASTE | Beans and Legumes | Lentils | IMPORT | Canada | Non-Detect | 2.3 | 0 |
| 837088 | 2014 | 11/19/2013 | COFFEE, BEANS | Non-Juice Beverages | Coffee | IMPORT | Haiti | Non-Detect | 0.233 | 0 |
| 837374 | 2014 | 11/19/2013 | PINTO BEAN, DRIED OR PASTE | Beans and Legumes | Pinto Beans | IMPORT | Mexico | Non-Detect | 0.233 | 0 |
| 837399 | 2014 | 11/19/2013 | HARD CANDY WITH NUT OR NUT PRODUCTS, N.E.C. (WITHOUT CHOCOLATE) | Candy and Chocolate Products | Candy with Nuts | IMPORT | India | Non-Detect | 1 | 0 |
| 837478 | 2014 | 11/19/2013 | WHEAT FLOUR, WHOLE | Grains and Grain Products | Wheat | IMPORT | India | Non-Detect | 1 | 0 |
| 837485 | 2014 | 11/19/2013 | PLAIN COOKIES, BISCUITS AND WAFERS, N.E.C. | Processed Food Products | Other Bakery Products | IMPORT | India | Non-Detect | 1 | 0 |
| 837433 | 2014 | 11/20/2013 | GRAIN, WHOLE, N.E.C. | Grains and Grain Products | Other Grains | IMPORT | Canada | Non-Detect | 2.3 | 0 |
| 837447 | 2014 | 11/20/2013 | WHEAT, WHOLE GRAIN | Grains and Grain Products | Wheat | IMPORT | Canada | Non-Detect | 2.3 | 0 |
| 837640 | 2014 | 11/20/2013 | CORN, DRIED OR PASTE | Grains and Grain Products | Corn | IMPORT | Vietnam | Non-Detect | 1 | 0 |
| 837642 | 2014 | 11/20/2013 | SPAGHETTI | Processed Food Products | Pasta | IMPORT | Italy | Non-Detect | 1 | 0 |
| 837606 | 2014 | 11/21/2013 | FLAX SEED (EDIBLE SEED) | Nuts and Edible Seeds | Other Nuts and Edible Seeds | IMPORT | Canada | Non-Detect | 2.3 | 0 |
| 837859 | 2014 | 11/21/2013 | WHEAT STARCH SNACKS, FRIED/OIL COOK | Processed Food Products | Other Snack Foods | IMPORT | Mexico | Non-Detect | 0.233 | 0 |
| 837701 | 2014 | 11/21/2013 | CORN CHIPS, FRIED | Processed Food Products | Other Corn Products | IMPORT | Philippines | Non-Detect | 1 | 0 |
| 837759 | 2014 | 11/22/2013 | WHEAT FLOUR, WHOLE | Grains and Grain Products | Wheat | IMPORT | Canada | Non-Detect | 1 | 0 |
| 837925 | 2014 | 11/22/2013 | CORN CHIPS, BAKED | Processed Food Products | Other Corn Products | IMPORT | Philippines | Non-Detect | 1 | 0 |
| 838072 | 2014 | 11/25/2013 | BARLEY, WHOLE GRAIN | Grains and Grain Products | Barley | IMPORT | Canada | Non-Detect | 2.3 | 0 |
| 838217 | 2014 | 11/25/2013 | WHEAT, WHOLE GRAIN | Grains and Grain Products | Wheat | IMPORT | Canada | Non-Detect | 2.3 | 0 |
| 838223 | 2014 | 11/25/2013 | SOYBEANS (SEE INDUSTRY 37 FOR 'BEAN CURD') | Beans and Legumes | Soybeans | IMPORT | Canada | Non-Detect | 2.3 | 0 |
| 838232 | 2014 | 11/25/2013 | SOYBEANS, WHOLE GRAIN | Beans and Legumes | Soybeans | IMPORT | Canada | Non-Detect | 2.3 | 0 |
| 838126 | 2014 | 11/25/2013 | COFFEE, BEANS | Non-Juice Beverages | Coffee | IMPORT | Colombia | Non-Detect | 0.233 | 0 |
| 831593 | 2014 | 11/25/2013 | RAISINS, DRIED OR PASTE | Fruit | Raisins | DOMESTIC | United States | Non-Detect | 1 | 0 |
| 838243 | 2014 | 11/26/2013 | MALT, BARLEY | Grains and Grain Products | Barley | IMPORT | Germany | Non-Detect | 2.3 | 0 |
| 838419 | 2014 | 11/26/2013 | SOYBEANS (EDIBLE SEED) | Beans and Legumes | Soybeans | IMPORT | Canada | Non-Detect | 2.3 | 0 |
| 838420 | 2014 | 11/26/2013 | SOYBEANS (EDIBLE SEED) | Beans and Legumes | Soybeans | IMPORT | Canada | Non-Detect | 2.3 | 0 |
| 838425 | 2014 | 11/26/2013 | SOYBEANS (SEE INDUSTRY 37 FOR 'BEAN CURD') | Beans and Legumes | Soybeans | IMPORT | Canada | Non-Detect | 2.3 | 0 |
| 838433 | 2014 | 11/26/2013 | SOYBEANS (SEE INDUSTRY 37 FOR 'BEAN CURD') | Beans and Legumes | Soybeans | IMPORT | Canada | Non-Detect | 2.3 | 0 |
| 838447 | 2014 | 11/26/2013 | BARLEY, WHOLE GRAIN | Grains and Grain Products | Barley | IMPORT | China | Non-Detect | 1 | 0 |
| 838810 | 2014 | 11/29/2013 | SOYBEANS (EDIBLE SEED) | Beans and Legumes | Soybeans | IMPORT | Canada | Non-Detect | 2.3 | 0 |
| 838818 | 2014 | 11/29/2013 | SOYBEANS (SEE INDUSTRY 37 FOR 'BEAN CURD') | Beans and Legumes | Soybeans | IMPORT | Canada | Non-Detect | 2.3 | 0 |
| 838831 | 2014 | 11/29/2013 | SOYBEANS, WHOLE GRAIN | Beans and Legumes | Soybeans | IMPORT | Canada | Non-Detect | 2.3 | 0 |
| 838893 | 2014 | 11/29/2013 | SOYBEANS (SEE INDUSTRY 37 FOR 'BEAN CURD') | Beans and Legumes | Soybeans | IMPORT | Canada | Non-Detect | 2.3 | 0 |
| 838680 | 2014 | 12/2/2013 | OATS, WHOLE GRAIN | Grains and Grain Products | Oats | IMPORT | Canada | Non-Detect | 2.3 | 0 |
| 838709 | 2014 | 12/2/2013 | SOYBEANS, WHOLE GRAIN | Beans and Legumes | Soybeans | IMPORT | Canada | Non-Detect | 2.3 | 0 |
| 838726 | 2014 | 12/2/2013 | SOYBEANS, WHOLE GRAIN | Beans and Legumes | Soybeans | IMPORT | Canada | Non-Detect | 2.3 | 0 |
| 838883 | 2014 | 12/2/2013 | BLACK EYE BEANS | Beans and Legumes | Blackeye Peas | IMPORT | Canada | Non-Detect | 2.3 | 0 |
| 831011 | 2014 | 12/2/2013 | WHEAT, WHOLE GRAIN | Grains and Grain Products | Wheat | DOMESTIC | United States | Non-Detect | 1 | 0 |
| 831012 | 2014 | 12/2/2013 | WHEAT, WHOLE GRAIN | Grains and Grain Products | Wheat | DOMESTIC | United States | Non-Detect | 1 | 0 |
| 838895 | 2014 | 12/3/2013 | COFFEE, BEANS | Non-Juice Beverages | Coffee | IMPORT | Uganda | Non-Detect | 0.233 | 0 |
| 830688 | 2014 | 12/3/2013 | RAISINS, DRIED OR PASTE | Fruit | Raisins | DOMESTIC | United States | Non-Detect | 1 | 0 |
| 839091 | 2014 | 12/4/2013 | WHEAT FLOUR, PLAIN | Grains and Grain Products | Wheat | IMPORT | Canada | Non-Detect | 2.3 | 0 |
| 839127 | 2014 | 12/4/2013 | OATS, WHOLE GRAIN | Grains and Grain Products | Oats | IMPORT | Canada | Non-Detect | 2.3 | 0 |
| 839379 | 2014 | 12/5/2013 | COFFEE, BEANS | Non-Juice Beverages | Coffee | IMPORT | Rwanda | Non-Detect | 2.3 | 0 |
| 839454 | 2014 | 12/5/2013 | COFFEE, BEANS | Non-Juice Beverages | Coffee | IMPORT | Burundi | Non-Detect | 2.3 | 0 |
| 839457 | 2014 | 12/5/2013 | OAT FLAKES, ROLLED OATS, PUFFS, KRISPIES, LOOPS READY TO EAT | Processed Food Products | Breakfast Foods | IMPORT | Canada | Non-Detect | 1 | 0 |
| 839618 | 2014 | 12/6/2013 | PEAS | Beans and Legumes | Peas | IMPORT | Canada | Non-Detect | 2.3 | 0 |
| 839769 | 2014 | 12/6/2013 | CORN, BOLTED MEAL OR FLOUR | Grains and Grain Products | Corn | IMPORT | India | Non-Detect | 1 | 0 |
| 839739 | 2014 | 12/9/2013 | RYE, WHOLE GRAIN | Grains and Grain Products | Rye | IMPORT | Canada | Non-Detect | 2.3 | 0 |
| 839868 | 2014 | 12/9/2013 | OATS, WHOLE GRAIN | Grains and Grain Products | Oats | IMPORT | Canada | Non-Detect | 2.3 | 0 |
| 839878 | 2014 | 12/9/2013 | SOYBEANS (SEE INDUSTRY 37 FOR 'BEAN CURD') | Beans and Legumes | Soybeans | IMPORT | Canada | Non-Detect | 2.3 | 0 |
| 839829 | 2014 | 12/9/2013 | WHEAT FLOUR, DURUM | Grains and Grain Products | Wheat | IMPORT | Canada | Non-Detect | 1 | 0 |
| 839888 | 2014 | 12/10/2013 | SOYBEANS, WHOLE GRAIN | Beans and Legumes | Soybeans | IMPORT | Canada | Non-Detect | 2.3 | 0 |
| 840250 | 2014 | 12/10/2013 | WHEAT FLOUR, ENRICHED (ALL PURPOSE) | Grains and Grain Products | Wheat | IMPORT | Canada | Non-Detect | 2.3 | 0 |
| 839886 | 2014 | 12/10/2013 | WHEAT FLOUR, WHOLE DURUM | Grains and Grain Products | Wheat | IMPORT | Dominican Republic (the) | Non-Detect | 0.233 | 0 |
| 839896 | 2014 | 12/10/2013 | KIDNEY BEAN | Beans and Legumes | Other Beans and Legumes | IMPORT | Nicaragua | Non-Detect | 0.233 | 0 |
| 840129 | 2014 | 12/11/2013 | VEGETABLE SPAGHETTI PRODUCTS | Processed Food Products | Pasta | IMPORT | Italy | Non-Detect | 1 | 0 |
| 840514 | 2014 | 12/12/2013 | PINTO BEAN | Beans and Legumes | Pinto Beans | IMPORT | Mexico | Non-Detect | 0.233 | 0 |
| 840526 | 2014 | 12/12/2013 | CORN CHIPS, BAKED | Processed Food Products | Other Corn Products | IMPORT | Philippines | Non-Detect | 1 | 0 |
| 840562 | 2014 | 12/15/2013 | WHEAT FLOUR, DURUM | Grains and Grain Products | Wheat | IMPORT | Canada | Non-Detect | 1 | 0 |

| | | | | | | | | | | |
|---|---|---|---|---|---|---|---|---|---|---|
| 840710 | 2014 | 12/16/2013 | BARLEY, WHOLE GRAIN | Grains and Grain Products | Barley | IMPORT | Morocco | Non-Detect | 1 | 0 |
| 840619 | 2014 | 12/16/2013 | PEABUTTER SPREAD | Beans and Legumes | Peas | IMPORT | Canada | Positive | 1 | 8.6 |
| 841086 | 2014 | 12/17/2013 | MALT, BARLEY | Grains and Grain Products | Barley | IMPORT | Canada | Non-Detect | 1 | 0 |
| 841209 | 2014 | 12/18/2013 | MALT, BARLEY | Grains and Grain Products | Barley | IMPORT | Germany | Non-Detect | 2.3 | 0 |
| 841318 | 2014 | 12/18/2013 | WHEAT FLOUR, N.E.C. | Grains and Grain Products | Wheat | IMPORT | Japan | Non-Detect | 2.3 | 0 |
| 841337 | 2014 | 12/18/2013 | COFFEE, BEANS | Non-Juice Beverages | Coffee | IMPORT | Guatemala | Non-Detect | 1 | 0 |
| 841716 | 2014 | 12/22/2013 | CORN, BOLTED MEAL OR FLOUR | Grains and Grain Products | Corn | IMPORT | Canada | Non-Detect | 1 | 0 |
| 827115 | 2014 | 12/23/2013 | CORN GRITS, BREWERS ENRICHED WHITE OR YELLOW CORN GRITS,CORN MEAL MUSH, QUICK OR INSTANT COOKING | Processed Food Products | Other Corn Products | DOMESTIC | United States | Non-Detect | 2.3 | 0 |
| 841724 | 2014 | 12/23/2013 | WHEAT GERM | Grains and Grain Products | Wheat | DOMESTIC | United States | Non-Detect | 2.3 | 0 |
| 808570 | 2014 | 12/26/2013 | RICE CEREAL (BABY) | Baby Food Products | Baby Cereals | DOMESTIC | United States | Non-Detect | 0.233 | 0 |
| 817014 | 2014 | 12/30/2013 | MALT, BARLEY | Grains and Grain Products | Barley | IMPORT | Unknown | Non-Detect | 2.3 | 0 |
| 817015 | 2014 | 12/30/2013 | MALT, BARLEY | Grains and Grain Products | Barley | IMPORT | United States | Non-Detect | 2.3 | 0 |
| 842143 | 2014 | 12/30/2013 | COFFEE, BEANS | Non-Juice Beverages | Coffee | IMPORT | Italy | Non-Detect | 2.3 | 0 |
| 840482 | 2014 | 12/31/2013 | WHEAT FLAKES, PUFFS, KRISPIES, LOOPS, SHREDDED, WHEAT GERM READY TO EAT | Processed Food Products | Breakfast Foods | DOMESTIC | United States | Non-Detect | 1 | 0 |
| 817016 | 2014 | 1/2/2014 | MALT, BARLEY | Grains and Grain Products | Barley | IMPORT | United States | Non-Detect | 2.2 | 0 |
| 822957 | 2014 | 1/2/2014 | BARLEY, WHOLE GRAIN | Grains and Grain Products | Barley | DOMESTIC | United States | Non-Detect | 0.233 | 0 |
| 765420 | 2014 | 1/2/2014 | RAISINS, DRIED OR PASTE | Fruit | Raisins | DOMESTIC | United States | Trace | 1 | |
| 817017 | 2014 | 1/3/2014 | MALT, BARLEY | Grains and Grain Products | Barley | IMPORT | United States | Non-Detect | 2.2 | 0 |
| 817018 | 2014 | 1/3/2014 | MALT PRODUCTS, N.E.C. | Grains and Grain Products | Other Grains | IMPORT | Germany | Non-Detect | 2.2 | 0 |
| 842612 | 2014 | 1/7/2014 | BEANS, N.E.C. (VEGETABLE) | Beans and Legumes | Other Beans and Legumes | IMPORT | Mexico | Non-Detect | 0.233 | 0 |
| 820275 | 2014 | 1/7/2014 | OATS, WHOLE GRAIN | Grains and Grain Products | Oats | DOMESTIC | United States | Non-Detect | 1 | 0 |
| 843692 | 2014 | 1/8/2014 | WHEAT, MILLED (CRUSHED, COARSE GROUND OR CRACKED) | Grains and Grain Products | Wheat | IMPORT | Turkey | Non-Detect | 2.2 | 0 |
| 843740 | 2014 | 1/8/2014 | WHEAT, MILLED (CRUSHED, COARSE GROUND OR CRACKED) | Grains and Grain Products | Wheat | IMPORT | Turkey | Non-Detect | 2.2 | 0 |
| 843767 | 2014 | 1/8/2014 | WHEAT, MILLED (CRUSHED, COARSE GROUND OR CRACKED) | Grains and Grain Products | Wheat | IMPORT | Turkey | Non-Detect | 2.2 | 0 |
| 843238 | 2014 | 1/9/2014 | BLACKEYE PEAS, DRIED OR PASTE | Beans and Legumes | Blackeye Peas | IMPORT | China | Non-Detect | 2.2 | 0 |
| 843251 | 2014 | 1/9/2014 | BLACKEYE PEAS, DRIED OR PASTE | Beans and Legumes | Blackeye Peas | IMPORT | Thailand | Non-Detect | 2.2 | 0 |
| 807207 | 2014 | 1/9/2014 | OATS, WHOLE GRAIN | Grains and Grain Products | Oats | DOMESTIC | United States | Non-Detect | 1 | 0 |
| 824990 | 2014 | 1/9/2014 | RAISINS (DRIED GRAPES) (BERRY) | Fruit | Raisins | DOMESTIC | United States | Non-Detect | 1 | 0 |
| 840483 | 2014 | 1/10/2014 | MIXED CEREAL FLAKES, SHREDDED AND OTHER FORMS READY TO EAT | Processed Food Products | Breakfast Foods | DOMESTIC | United States | Non-Detect | 1 | 0 |
| 822958 | 2014 | 1/13/2014 | BARLEY, WHOLE GRAIN | Grains and Grain Products | Barley | DOMESTIC | United States | Non-Detect | 4.96 | 0 |
| 843297 | 2014 | 1/13/2014 | RAISINS (DRIED GRAPES) (BERRY) | Fruit | Raisins | IMPORT | Argentina | Positive | 4.99 | 27.8 |
| 768651 | 2014 | 1/14/2014 | WHEAT FLOUR, ENRICHED (ALL PURPOSE) | Grains and Grain Products | Wheat | DOMESTIC | United States | Non-Detect | 2.2 | 0 |
| 843618 | 2014 | 1/14/2014 | PINTO BEAN, DRIED OR PASTE | Beans and Legumes | Pinto Beans | IMPORT | Mexico | Non-Detect | 4.99 | 0 |
| 843633 | 2014 | 1/14/2014 | BEAN, DRIED OR PASTE, N.E.C. (VEGETABLE) | Beans and Legumes | Other Beans and Legumes | IMPORT | Mexico | Non-Detect | 4.99 | 0 |
| 831164 | 2014 | 1/14/2014 | CORN CHIPS, FRIED | Processed Food Products | Other Corn Products | DOMESTIC | United States | Non-Detect | 1 | 0 |
| 843824 | 2014 | 1/15/2014 | CORN, HOMINY GRITS | Grains and Grain Products | Corn | IMPORT | Ethiopia | Non-Detect | 2.2 | 0 |
| 843775 | 2014 | 1/15/2014 | PINTO BEAN, DRIED OR PASTE | Beans and Legumes | Pinto Beans | IMPORT | Mexico | Non-Detect | 4.99 | 0 |
| 843749 | 2014 | 1/15/2014 | CORN, BOLTED MEAL OR FLOUR | Grains and Grain Products | Corn | IMPORT | China | Non-Detect | 1 | 0 |
| 837536 | 2014 | 1/16/2014 | WHEAT FLOUR, WHOLE | Grains and Grain Products | Wheat | DOMESTIC | United States | Non-Detect | 2.2 | 0 |
| 844314 | 2014 | 1/16/2014 | WHEAT, MILLED (CRUSHED, COARSE GROUND OR CRACKED) | Grains and Grain Products | Wheat | IMPORT | Turkey | Non-Detect | 2.2 | 0 |
| 844082 | 2014 | 1/16/2014 | BEAN, DRIED OR PASTE, N.E.C. (VEGETABLE) | Beans and Legumes | Other Beans and Legumes | IMPORT | Thailand | Non-Detect | 1 | 0 |
| 844446 | 2014 | 1/21/2014 | COFFEE, BEANS | Non-Juice Beverages | Coffee | IMPORT | Colombia | Non-Detect | 2.2 | 0 |
| 844459 | 2014 | 1/21/2014 | PINTO BEAN, DRIED OR PASTE | Beans and Legumes | Pinto Beans | IMPORT | Mexico | Non-Detect | 0.233 | 0 |
| 844521 | 2014 | 1/22/2014 | CORN FLAKES, PUFFS, KRISPIES, LOOPS READY TO EAT | Processed Food Products | Other Corn Products | IMPORT | Guatemala | Non-Detect | 2.2 | 0 |
| 844532 | 2014 | 1/22/2014 | CORN FLAKES, PUFFS, KRISPIES, LOOPS READY TO EAT | Processed Food Products | Other Corn Products | IMPORT | Guatemala | Non-Detect | 2.2 | 0 |
| 844612 | 2014 | 1/22/2014 | SOYBEANS (SEE INDUSTRY 37 FOR 'BEAN CURD') | Beans and Legumes | Soybeans | IMPORT | China | Non-Detect | 2.2 | 0 |
| 844658 | 2014 | 1/22/2014 | WHEAT FLOUR, ENRICHED (ALL PURPOSE) | Grains and Grain Products | Wheat | IMPORT | India | Non-Detect | 1 | 0 |
| 844693 | 2014 | 1/23/2014 | COFFEE, BEANS | Non-Juice Beverages | Coffee | IMPORT | Colombia | Non-Detect | 2.2 | 0 |
| 844944 | 2014 | 1/23/2014 | ADZUKI BEANS | Beans and Legumes | Other Beans and Legumes | IMPORT | China | Non-Detect | 2.2 | 0 |
| 845942 | 2014 | 1/23/2014 | WHEAT FLOUR, WHOLE | Grains and Grain Products | Wheat | IMPORT | Canada | Non-Detect | 0.233 | 0 |
| 763958 | 2014 | 1/23/2014 | RICE CEREAL (BABY) | Baby Food Products | Baby Cereals | DOMESTIC | United States | Trace | 4.99 | |
| 845004 | 2014 | 1/24/2014 | KIDNEY BEAN, DRIED OR PASTE | Beans and Legumes | Other Beans and Legumes | IMPORT | Argentina | Non-Detect | 2.2 | 0 |
| 845191 | 2014 | 1/24/2014 | CORN CHIPS, FRIED | Processed Food Products | Other Corn Products | IMPORT | Mexico | Non-Detect | 1 | 0 |
| 845071 | 2014 | 1/26/2014 | WHEAT, WHOLE GRAIN | Grains and Grain Products | Wheat | IMPORT | Canada | Non-Detect | 1 | 0 |
| 845074 | 2014 | 1/26/2014 | URAD DAL (INDIAN DRIED, HULLED AND SPLIT BLACK GRAM BEANS, IVORY WHITE IN COLOR) | Beans and Legumes | Lentils | IMPORT | United Arab Emirates | Non-Detect | 1 | 0 |
| 845227 | 2014 | 1/27/2014 | PINTO BEAN, DRIED OR PASTE | Beans and Legumes | Pinto Beans | IMPORT | Mexico | Non-Detect | 4.99 | 0 |
| 845548 | 2014 | 1/28/2014 | YELLOW BEANS, DRIED OR PASTE | Beans and Legumes | Other Beans and Legumes | IMPORT | Mexico | Non-Detect | 0.233 | 0 |
| 845424 | 2014 | 1/28/2014 | WHEAT BRAN (HUMAN USE) | Grains and Grain Products | Wheat | IMPORT | Canada | Non-Detect | 1 | 0 |
| 845654 | 2014 | 1/29/2014 | PINTO BEAN, DRIED OR PASTE | Beans and Legumes | Pinto Beans | IMPORT | Mexico | Non-Detect | 4.99 | 0 |
| 845761 | 2014 | 1/29/2014 | BEANS, N.E.C. (VEGETABLE) | Beans and Legumes | Other Beans and Legumes | IMPORT | Mexico | Non-Detect | 5 | 0 |
| 845878 | 2014 | 1/30/2014 | PINTO BEAN, DRIED OR PASTE | Beans and Legumes | Pinto Beans | IMPORT | Mexico | Non-Detect | 0.233 | 0 |
| 846072 | 2014 | 1/30/2014 | PERUANO BEANS, DRIED OR PASTE | Beans and Legumes | Other Beans and Legumes | IMPORT | Mexico | Non-Detect | 5 | 0 |
| 845784 | 2014 | 1/30/2014 | OATMEAL, REGULAR, FRUIT OR SPICE ADDED, QUICK OR INSTANT COOKING | Processed Food Products | Breakfast Foods | IMPORT | Canada | Non-Detect | 1 | 0 |
| 845786 | 2014 | 1/30/2014 | MALT, BARLEY | Grains and Grain Products | Barley | IMPORT | United States | Non-Detect | 1 | 0 |
| 845917 | 2014 | 1/30/2014 | OATMEAL, REGULAR, FRUIT OR SPICE ADDED, QUICK OR INSTANT COOKING | Processed Food Products | Breakfast Foods | IMPORT | Canada | Non-Detect | 1 | 0 |
| 846167 | 2014 | 1/31/2014 | BARLEY, WHOLE GRAIN | Grains and Grain Products | Barley | IMPORT | Ethiopia | Non-Detect | 2.2 | 0 |
| 750114 | 2014 | 1/31/2014 | WHEAT FLOUR, WHOLE | Grains and Grain Products | Wheat | DOMESTIC | United States | Non-Detect | 1 | 0 |
| 829341 | 2014 | 2/3/2014 | RICE CEREAL (BABY) | Baby Food Products | Baby Cereals | DOMESTIC | United States | Non-Detect | 2.2 | 0 |
| 829342 | 2014 | 2/3/2014 | OAT CEREAL (BABY) | Baby Food Products | Baby Cereals | IMPORT | Chile | Non-Detect | 2.3 | 0 |
| 846597 | 2014 | 2/3/2014 | GARBANZO BEAN | Beans and Legumes | Garbanzo Beans | IMPORT | Canada | Non-Detect | 1 | 0 |
| 846646 | 2014 | 2/3/2014 | CORN NUTS SNACK | Processed Food Products | Other Corn Products | IMPORT | Peru | Non-Detect | 1 | 0 |
| 823213 | 2014 | 2/4/2014 | WHEAT FLOUR, WHOLE | Grains and Grain Products | Wheat | DOMESTIC | United States | Non-Detect | 2.2 | 0 |
| 847066 | 2014 | 2/4/2014 | WHEAT STARCH SNACKS, FRIED/OIL COOK | Processed Food Products | Other Snack Foods | IMPORT | Indonesia | Non-Detect | 1 | 0 |
| 846669 | 2014 | 2/5/2014 | WHEAT, WHOLE GRAIN | Grains and Grain Products | Wheat | IMPORT | Canada | Non-Detect | 1 | 0 |
| 846968 | 2014 | 2/6/2014 | BRAN FLAKES, SHREDDED AND OTHER FORMS READY TO EAT | Processed Food Products | Breakfast Foods | IMPORT | Mexico | Non-Detect | 0.233 | 0 |
| 847361 | 2014 | 2/6/2014 | WHEAT, MILLED (CRUSHED, COARSE GROUND OR CRACKED) | Grains and Grain Products | Wheat | IMPORT | Canada | Non-Detect | 1 | 0 |
| 847418 | 2014 | 2/10/2014 | BUCKWHEAT, WHOLE GRAIN | Grains and Grain Products | Buckwheat | IMPORT | China | Non-Detect | 0.233 | 0 |
| 848208 | 2014 | 2/12/2014 | WHEAT, WHOLE GRAIN | Grains and Grain Products | Wheat | DOMESTIC | United States | Non-Detect | 2.2 | 0 |
| 847798 | 2014 | 2/12/2014 | COFFEE, BEANS | Non-Juice Beverages | Coffee | IMPORT | Peru | Non-Detect | 1 | 0 |
| 847629 | 2014 | 2/12/2014 | RAISINS (DRIED GRAPES) (BERRY) | Fruit | Raisins | IMPORT | Argentina | Positive | 5 | 16.5 |
| 848150 | 2014 | 2/18/2014 | BARLEY, WHOLE GRAIN | Grains and Grain Products | Barley | IMPORT | China | Non-Detect | 1 | 0 |
| 796691 | 2014 | 2/19/2014 | MIXED GRAIN CEREAL (BABY) | Baby Food Products | Baby Cereals | DOMESTIC | United States | Non-Detect | 0.233 | 0 |
| 848400 | 2014 | 2/19/2014 | PINTO BEAN | Beans and Legumes | Pinto Beans | IMPORT | Mexico | Non-Detect | 0.233 | 0 |
| 848481 | 2014 | 2/19/2014 | PINTO BEAN | Beans and Legumes | Pinto Beans | IMPORT | Mexico | Non-Detect | 0.233 | 0 |
| 835092 | 2014 | 2/19/2014 | WHEAT FLOUR, BROMATED | Grains and Grain Products | Wheat | DOMESTIC | United States | Non-Detect | 1 | 0 |
| 848646 | 2014 | 2/20/2014 | BUCKWHEAT, WHOLE GRAIN | Grains and Grain Products | Buckwheat | IMPORT | Russia | Non-Detect | 1 | 0 |
| 849026 | 2014 | 2/21/2014 | PINTO BEAN | Beans and Legumes | Pinto Beans | IMPORT | Mexico | Non-Detect | 0.233 | 0 |
| 849039 | 2014 | 2/23/2014 | PIGEON PEAS, DRIED OR PASTE | Beans and Legumes | Peas | IMPORT | Peru | Non-Detect | 1 | 0 |
| 803963 | 2014 | 2/25/2014 | WHEAT, WHOLE GRAIN | Grains and Grain Products | Wheat | DOMESTIC | United States | Non-Detect | 2.2 | 0 |
| 849477 | 2014 | 2/25/2014 | BEAN, DRIED OR PASTE, N.E.C. (VEGETABLE) | Beans and Legumes | Other Beans and Legumes | IMPORT | Mexico | Non-Detect | 0.233 | 0 |
| 849544 | 2014 | 2/25/2014 | CORN CHIPS, FRIED | Processed Food Products | Other Corn Products | IMPORT | Philippines | Non-Detect | 1 | 0 |
| 846333 | 2014 | 2/26/2014 | RYE FLOUR | Grains and Grain Products | Rye | DOMESTIC | United States | Non-Detect | 2.2 | 0 |
| 690212 | 2014 | 2/26/2014 | WHEAT FLOUR, ENRICHED (ALL PURPOSE) | Grains and Grain Products | Wheat | DOMESTIC | United States | Non-Detect | 1 | 0 |

| | | | | | | | | | | |
|---|---|---|---|---|---|---|---|---|---|---|
| 690213 | 2014 | 2/26/2014 | PRETZELS, BAKED | Processed Food Products | Other Snack Foods | DOMESTIC | United States | Non-Detect | 1 | 0 |
| 690214 | 2014 | 2/26/2014 | PRETZELS, BAKED | Processed Food Products | Other Snack Foods | DOMESTIC | United States | Non-Detect | 1 | 0 |
| 690215 | 2014 | 2/26/2014 | CREAM WHEAT, QUICK OR INSTANT COOKING | Processed Food Products | Breakfast Foods | DOMESTIC | United States | Non-Detect | 1 | 0 |
| 843315 | 2014 | 2/27/2014 | WHEAT CEREAL (BABY) | Baby Food Products | Baby Cereals | DOMESTIC | United States | Non-Detect | 0.233 | 0 |
| 849950 | 2014 | 2/27/2014 | OAT FLAKES, ROLLED OATS, PUFFS, KRISPIES, LOOPS READY TO EAT | Processed Food Products | Breakfast Foods | IMPORT | Mexico | Non-Detect | 0.233 | 0 |
| 849991 | 2014 | 2/27/2014 | COFFEE, BEANS | Non-Juice Beverages | Coffee | IMPORT | Vietnam | Non-Detect | 1 | 0 |
| 850340 | 2014 | 2/27/2014 | OATMEAL, REGULAR, FRUIT OR SPICE ADDED, QUICK OR INSTANT COOKING | Processed Food Products | Breakfast Foods | IMPORT | Canada | Non-Detect | 1 | 0 |
| 850497 | 2014 | 3/3/2014 | COFFEE, BEANS | Non-Juice Beverages | Coffee | IMPORT | Mexico | Non-Detect | 1 | 0 |
| 827083 | 2014 | 3/6/2014 | SPAGHETTI | Processed Food Products | Pasta | DOMESTIC | Unknown | Non-Detect | 1 | 0 |
| 851093 | 2014 | 3/6/2014 | LENTILS, DRIED OR PASTE | Beans and Legumes | Lentils | IMPORT | Canada | Non-Detect | 1 | 0 |
| 836267 | 2014 | 3/10/2014 | SPAGHETTI | Processed Food Products | Pasta | DOMESTIC | Unknown | Non-Detect | 1 | 0 |
| 851967 | 2014 | 3/11/2014 | PERUANO BEANS, DRIED OR PASTE | Beans and Legumes | Other Beans and Legumes | IMPORT | Mexico | Non-Detect | 0.233 | 0 |
| 852459 | 2014 | 3/17/2014 | BLACKEYE PEAS, DRIED OR PASTE | Beans and Legumes | Blackeye Peas | IMPORT | China | Non-Detect | 2.2 | 0 |
| 852512 | 2014 | 3/17/2014 | WHEAT, WHOLE GRAIN | Grains and Grain Products | Wheat | IMPORT | Canada | Non-Detect | 1 | 0 |
| 852529 | 2014 | 3/17/2014 | COFFEE, BEANS, DECAFFEINATED | Non-Juice Beverages | Coffee | IMPORT | Canada | Non-Detect | 1 | 0 |
| 852554 | 2014 | 3/17/2014 | COFFEE, BEANS | Non-Juice Beverages | Coffee | IMPORT | Guatemala | Non-Detect | 1 | 0 |
| 852643 | 2014 | 3/18/2014 | COFFEE, BEANS | Non-Juice Beverages | Coffee | IMPORT | Honduras | Non-Detect | 2.2 | 0 |
| 852830 | 2014 | 3/18/2014 | KIDNEY BEAN, DRIED OR PASTE | Beans and Legumes | Other Beans and Legumes | IMPORT | El Salvador | Non-Detect | 2.2 | 0 |
| 852509 | 2014 | 3/18/2014 | WHEAT FLAKES, PUFFS, KRISPIES, LOOPS, SHREDDED, WHEAT GERM READY TO EAT | Processed Food Products | Breakfast Foods | DOMESTIC | United States | Non-Detect | 0.233 | 0 |
| 852904 | 2014 | 3/19/2014 | MAYOCOBA BEANS, DRIED OR PASTE | Beans and Legumes | Other Beans and Legumes | IMPORT | Mexico | Non-Detect | 0.233 | 0 |
| 852806 | 2014 | 3/19/2014 | COFFEE, BEANS | Non-Juice Beverages | Coffee | IMPORT | Canada | Non-Detect | 1 | 0 |
| 853026 | 2014 | 3/20/2014 | COFFEE, BEANS | Non-Juice Beverages | Coffee | IMPORT | Dominican Republic (the) | Non-Detect | 2.2 | 0 |
| 853426 | 2014 | 3/21/2014 | CORN, WHOLE GRAIN | Grains and Grain Products | Corn | IMPORT | Peru | Non-Detect | 1 | 0 |
| 853429 | 2014 | 3/21/2014 | CORN, WHOLE GRAIN | Grains and Grain Products | Corn | IMPORT | Peru | Non-Detect | 1 | 0 |
| 853775 | 2014 | 3/25/2014 | COFFEE, BEANS | Non-Juice Beverages | Coffee | IMPORT | Mexico | Non-Detect | 2.2 | 0 |
| 853884 | 2014 | 3/25/2014 | BUCKWHEAT, WHOLE GRAIN | Grains and Grain Products | Buckwheat | IMPORT | China | Non-Detect | 2.2 | 0 |
| 853790 | 2014 | 3/25/2014 | PINTO BEAN | Beans and Legumes | Pinto Beans | IMPORT | Mexico | Non-Detect | 0.233 | 0 |
| 854118 | 2014 | 3/26/2014 | PERUANO BEANS, DRIED OR PASTE | Beans and Legumes | Other Beans and Legumes | IMPORT | Mexico | Non-Detect | 0.233 | 0 |
| 845325 | 2014 | 3/26/2014 | OAT FLOUR | Grains and Grain Products | Oats | DOMESTIC | United States | Trace | 1 | |
| 854245 | 2014 | 3/27/2014 | OATMEAL, REGULAR, FRUIT OR SPICE ADDED, QUICK OR INSTANT COOKING | Processed Food Products | Breakfast Foods | IMPORT | Canada | Non-Detect | 1 | 0 |
| 825141 | 2014 | 4/1/2014 | OAT FLAKES, ROLLED OATS, PUFFS, KRISPIES, LOOPS READY TO EAT | Processed Food Products | Breakfast Foods | DOMESTIC | United States | Non-Detect | 0.233 | 0 |
| 817031 | 2014 | 4/2/2014 | COFFEE, BEANS | Non-Juice Beverages | Coffee | IMPORT | Unknown | Non-Detect | 2.2 | 0 |
| 798298 | 2014 | 4/2/2014 | RAISINS, DRIED OR PASTE | Fruit | Raisins | DOMESTIC | United States | Non-Detect | 0.233 | 0 |
| 855906 | 2014 | 4/3/2014 | KIDNEY BEAN | Beans and Legumes | Other Beans and Legumes | IMPORT | Honduras | Non-Detect | 2.2 | 0 |
| 855938 | 2014 | 4/3/2014 | MALT, BARLEY | Grains and Grain Products | Barley | IMPORT | United Kingdom | Non-Detect | 2.2 | 0 |
| 855740 | 2014 | 4/3/2014 | OATS, WHOLE GRAIN | Grains and Grain Products | Oats | IMPORT | Canada | Non-Detect | 1 | 0 |
| 855861 | 2014 | 4/3/2014 | FLOURS AND MEALS N.E.C. | Grains and Grain Products | Other Grains | IMPORT | Bolivia | Non-Detect | 1 | 0 |
| 855963 | 2014 | 4/4/2014 | WHEAT, MILLED (CRUSHED, COARSE GROUND OR CRACKED) | Grains and Grain Products | Wheat | IMPORT | Germany | Non-Detect | 2.2 | 0 |
| 846341 | 2014 | 4/8/2014 | PEAS | Beans and Legumes | Peas | DOMESTIC | United States | Non-Detect | 2.2 | 0 |
| 856334 | 2014 | 4/8/2014 | BARLEY FLOUR | Grains and Grain Products | Barley | IMPORT | El Salvador | Non-Detect | 2.2 | 0 |
| 856425 | 2014 | 4/8/2014 | COFFEE, BEANS | Non-Juice Beverages | Coffee | IMPORT | Tanzania, United Republic Of | Non-Detect | 2.2 | 0 |
| 856438 | 2014 | 4/8/2014 | WHOLE WHEAT BREAD/ROLLS/BUNS | Processed Food Products | Bread | IMPORT | Germany | Non-Detect | 1 | 0 |
| 856959 | 2014 | 4/8/2014 | CORN, ENRICHED BOLTED MEAL | Grains and Grain Products | Corn | IMPORT | Italy | Non-Detect | 1 | 0 |
| 821416 | 2014 | 4/9/2014 | PINTO BEAN | Beans and Legumes | Pinto Beans | DOMESTIC | United States | Non-Detect | 2.2 | 0 |
| 856556 | 2014 | 4/9/2014 | COFFEE, BEANS | Non-Juice Beverages | Coffee | IMPORT | Indonesia | Non-Detect | 2.2 | 0 |
| 856682 | 2014 | 4/9/2014 | GLUTEN, WHEAT | Grains and Grain Products | Wheat | IMPORT | Australia | Non-Detect | 2.2 | 0 |
| 857136 | 2014 | 4/9/2014 | FAVA BEAN, DRIED OR PASTE | Beans and Legumes | Other Beans and Legumes | IMPORT | Egypt | Non-Detect | 2.2 | 0 |
| 857137 | 2014 | 4/9/2014 | LUPIN BEAN, DRIED OR PASTE | Beans and Legumes | Other Beans and Legumes | IMPORT | Egypt | Non-Detect | 2.2 | 0 |
| 857138 | 2014 | 4/9/2014 | NAVY (WHITE) BEANS (BAKED BEANS, PORK AND BEANS), DRIED OR PASTE | Beans and Legumes | Other Beans and Legumes | IMPORT | Egypt | Non-Detect | 2.2 | 0 |
| 857141 | 2014 | 4/9/2014 | LENTILS, DRIED OR PASTE | Beans and Legumes | Lentils | IMPORT | Egypt | Non-Detect | 2.2 | 0 |
| 857145 | 2014 | 4/9/2014 | GARBANZO BEAN, DRIED OR PASTE | Beans and Legumes | Garbanzo Beans | IMPORT | Egypt | Non-Detect | 2.2 | 0 |
| 856714 | 2014 | 4/9/2014 | FAVA BEAN | Beans and Legumes | Other Beans and Legumes | IMPORT | Mexico | Non-Detect | 0.233 | 0 |
| 856758 | 2014 | 4/9/2014 | FAVA BEAN | Beans and Legumes | Other Beans and Legumes | IMPORT | Mexico | Non-Detect | 0.233 | 0 |
| 857654 | 2014 | 4/9/2014 | OAT FLAKES, ROLLED OATS, PUFFS, KRISPIES, LOOPS READY TO EAT | Processed Food Products | Breakfast Foods | IMPORT | Mexico | Non-Detect | 0.233 | 0 |
| 855274 | 2014 | 4/10/2014 | RYE FLOUR | Grains and Grain Products | Rye | DOMESTIC | United States | Non-Detect | 0.233 | 0 |
| 857526 | 2014 | 4/13/2014 | RAISINS (DRIED GRAPES) (BERRY) | Fruit | Raisins | IMPORT | Afghanistan | Trace | 1 | |
| 857324 | 2014 | 4/14/2014 | COFFEE, BEANS | Non-Juice Beverages | Coffee | IMPORT | Indonesia | Non-Detect | 2.2 | 0 |
| 826138 | 2014 | 4/14/2014 | CORN FLAKES, PUFFS, KRISPIES, LOOPS READY TO EAT | Processed Food Products | Other Corn Products | DOMESTIC | Unknown | Non-Detect | 0.233 | 0 |
| 857406 | 2014 | 4/14/2014 | CORN, BOLTED MEAL OR FLOUR | Grains and Grain Products | Corn | IMPORT | Italy | Non-Detect | 1 | 0 |
| 857830 | 2014 | 4/15/2014 | SOYBEANS (SEE INDUSTRY 37 FOR 'BEAN CURD') | Beans and Legumes | Soybeans | IMPORT | Canada | Non-Detect | 2.2 | 0 |
| 857864 | 2014 | 4/15/2014 | WHEAT FLOUR, WHOLE | Grains and Grain Products | Wheat | IMPORT | India | Non-Detect | 2.2 | 0 |
| 857931 | 2014 | 4/15/2014 | WHEAT, WHOLE GRAIN | Grains and Grain Products | Wheat | IMPORT | Canada | Non-Detect | 2.2 | 0 |
| 857947 | 2014 | 4/15/2014 | WHEAT, WHOLE GRAIN | Grains and Grain Products | Wheat | IMPORT | Canada | Non-Detect | 2.2 | 0 |
| 857593 | 2014 | 4/15/2014 | KIDNEY BEAN | Beans and Legumes | Other Beans and Legumes | IMPORT | Spain | Non-Detect | 0.233 | 0 |
| 857850 | 2014 | 4/15/2014 | MIXED CEREAL, QUICK OR INSTANT COOKING | Processed Food Products | Breakfast Foods | IMPORT | Senegal | Non-Detect | 1 | 0 |
| 857998 | 2014 | 4/15/2014 | RAISINS (DRIED GRAPES) (BERRY) | Fruit | Raisins | IMPORT | Pakistan | Non-Detect | 1 | 0 |
| 858059 | 2014 | 4/16/2014 | SOYBEANS (EDIBLE SEED) | Beans and Legumes | Soybeans | IMPORT | Canada | Non-Detect | 2.2 | 0 |
| 858061 | 2014 | 4/16/2014 | SOYBEANS (EDIBLE SEED) | Beans and Legumes | Soybeans | IMPORT | Canada | Non-Detect | 2.2 | 0 |
| 858070 | 2014 | 4/16/2014 | WHEAT, WHOLE GRAIN | Grains and Grain Products | Wheat | IMPORT | Canada | Non-Detect | 2.2 | 0 |
| 858073 | 2014 | 4/16/2014 | SOYBEANS (SEE INDUSTRY 37 FOR 'BEAN CURD') | Beans and Legumes | Soybeans | IMPORT | Canada | Non-Detect | 2.2 | 0 |
| 857856 | 2014 | 4/16/2014 | COFFEE, BEANS | Non-Juice Beverages | Coffee | IMPORT | Nicaragua | Non-Detect | 0.233 | 0 |
| 839297 | 2014 | 4/17/2014 | BARLEY, WHOLE GRAIN | Grains and Grain Products | Barley | DOMESTIC | United States | Non-Detect | 2.2 | 0 |
| 839298 | 2014 | 4/17/2014 | BARLEY, WHOLE GRAIN | Grains and Grain Products | Barley | DOMESTIC | United States | Non-Detect | 2.2 | 0 |
| 839299 | 2014 | 4/17/2014 | BARLEY, WHOLE GRAIN | Grains and Grain Products | Barley | DOMESTIC | United States | Non-Detect | 2.2 | 0 |
| 858479 | 2014 | 4/21/2014 | SOYBEANS (SEE INDUSTRY 37 FOR 'BEAN CURD') | Beans and Legumes | Soybeans | IMPORT | Canada | Non-Detect | 2.2 | 0 |
| 858514 | 2014 | 4/21/2014 | OATS, WHOLE GRAIN | Grains and Grain Products | Oats | IMPORT | Canada | Non-Detect | 2.2 | 0 |
| 858590 | 2014 | 4/21/2014 | COFFEE, BEANS | Non-Juice Beverages | Coffee | IMPORT | Honduras | Non-Detect | 1 | 0 |
| 858600 | 2014 | 4/22/2014 | OAT CEREAL (BABY) | Baby Food Products | Baby Cereals | IMPORT | Chile | Non-Detect | 2.2 | 0 |
| 858602 | 2014 | 4/22/2014 | WHEAT CEREAL (BABY) | Baby Food Products | Baby Cereals | IMPORT | Chile | Non-Detect | 2.2 | 0 |
| 858665 | 2014 | 4/22/2014 | COFFEE, BEANS | Non-Juice Beverages | Coffee | IMPORT | Colombia | Non-Detect | 2.2 | 0 |
| 858679 | 2014 | 4/22/2014 | COFFEE, BEANS | Non-Juice Beverages | Coffee | IMPORT | Brazil | Non-Detect | 2.2 | 0 |
| 858814 | 2014 | 4/22/2014 | WHEAT, MILLED (CRUSHED, COARSE GROUND OR CRACKED) | Grains and Grain Products | Wheat | IMPORT | Turkey | Non-Detect | 2.2 | 0 |
| 858691 | 2014 | 4/22/2014 | CORN, MILLED PRODUCT N.E.C. | Grains and Grain Products | Corn | IMPORT | China | Non-Detect | 1 | 0 |
| 858762 | 2014 | 4/22/2014 | CORN PUFFS (FLAVORED OR UNFLAVORED), VEGETABLE SNACKS (OTHER THAN PULSES) BAKED | Processed Food Products | Other Corn Products | IMPORT | Philippines | Non-Detect | 1 | 0 |
| 858851 | 2014 | 4/23/2014 | OATS, WHOLE GRAIN | Grains and Grain Products | Oats | IMPORT | Canada | Non-Detect | 2.2 | 0 |
| 858925 | 2014 | 4/23/2014 | SOYBEANS (SEE INDUSTRY 37 FOR 'BEAN CURD') | Beans and Legumes | Soybeans | IMPORT | Canada | Non-Detect | 2.2 | 0 |
| 858957 | 2014 | 4/23/2014 | OATS, WHOLE GRAIN | Grains and Grain Products | Oats | IMPORT | Canada | Non-Detect | 2.2 | 0 |
| 859072 | 2014 | 4/23/2014 | BREAD/ROLLS/BUNS, N.E.C. | Processed Food Products | Bread | IMPORT | Greece | Non-Detect | 2.2 | 0 |
| 859076 | 2014 | 4/23/2014 | RYE BREAD/ROLLS/BUNS | Processed Food Products | Bread | IMPORT | Greece | Non-Detect | 2.2 | 0 |
| 859288 | 2014 | 4/23/2014 | CORN (WHOLE KERNEL) SNACKS, VEGETABLE SNACKS (OTHER THAN PULSES) BAKED | Processed Food Products | Other Corn Products | IMPORT | Ukraine | Non-Detect | 1 | 0 |
| 859291 | 2014 | 4/23/2014 | BUCKWHEAT, WHOLE GRAIN | Grains and Grain Products | Buckwheat | IMPORT | Ukraine | Non-Detect | 1 | 0 |
| 859116 | 2014 | 4/24/2014 | URAD DAL (INDIAN DRIED, HULLED AND SPLIT BLACK GRAM BEANS, IVORY WHITE IN COLOR) | Beans and Legumes | Lentils | IMPORT | Thailand | Non-Detect | 2.2 | 0 |
| 859452 | 2014 | 4/24/2014 | BARLEY, WHOLE GRAIN | Grains and Grain Products | Barley | IMPORT | Russia | Non-Detect | 1 | 0 |

| | | | | | | | | | | |
|---|---|---|---|---|---|---|---|---|---|---|
| 859293 | 2014 | 4/25/2014 | SOYBEANS, WHOLE GRAIN | Beans and Legumes | Soybeans | IMPORT | Canada | Non-Detect | 2.2 | 0 |
| 859500 | 2014 | 4/28/2014 | OATS, WHOLE GRAIN | Grains and Grain Products | Oats | IMPORT | Canada | Non-Detect | 2.2 | 0 |
| 859622 | 2014 | 4/28/2014 | OATS, WHOLE GRAIN | Grains and Grain Products | Oats | IMPORT | Canada | Non-Detect | 2.2 | 0 |
| 859641 | 2014 | 4/29/2014 | OATS, WHOLE GRAIN | Grains and Grain Products | Oats | IMPORT | Canada | Non-Detect | 2.2 | 0 |
| 859892 | 2014 | 4/29/2014 | SOYBEANS (SEE INDUSTRY 37 FOR 'BEAN CURD') | Beans and Legumes | Soybeans | IMPORT | Canada | Non-Detect | 2.2 | 0 |
| 859978 | 2014 | 4/30/2014 | COFFEE, BEANS | Non-Juice Beverages | Coffee | IMPORT | Vietnam | Non-Detect | 2.2 | 0 |
| 860441 | 2014 | 5/1/2014 | OATS, WHOLE GRAIN | Grains and Grain Products | Oats | IMPORT | Canada | Non-Detect | 2.2 | 0 |
| 860421 | 2014 | 5/1/2014 | BARLEY, WHOLE GRAIN | Grains and Grain Products | Barley | IMPORT | China | Non-Detect | 1 | 0 |
| 860735 | 2014 | 5/1/2014 | RED BEAN, DRIED OR PASTE | Beans and Legumes | Other Beans and Legumes | IMPORT | China | Non-Detect | 1 | 0 |
| 860534 | 2014 | 5/2/2014 | SOYBEANS, WHOLE GRAIN | Beans and Legumes | Soybeans | IMPORT | Canada | Non-Detect | 2.2 | 0 |
| 860621 | 2014 | 5/2/2014 | SOYBEANS (SEE INDUSTRY 37 FOR 'BEAN CURD') | Beans and Legumes | Soybeans | IMPORT | Canada | Non-Detect | 2.2 | 0 |
| 860628 | 2014 | 5/5/2014 | LENTILS, DRIED OR PASTE | Beans and Legumes | Lentils | IMPORT | Turkey | Non-Detect | 2.2 | 0 |
| 860896 | 2014 | 5/6/2014 | SOYBEANS (EDIBLE SEED) | Beans and Legumes | Soybeans | IMPORT | Canada | Non-Detect | 2.2 | 0 |
| 861337 | 2014 | 5/6/2014 | COFFEE, GROUND | Non-Juice Beverages | Coffee | IMPORT | Guatemala | Non-Detect | 2.2 | 0 |
| 828649 | 2014 | 5/7/2014 | BARLEY, WHOLE GRAIN | Grains and Grain Products | Barley | DOMESTIC | United States | Non-Detect | 2.2 | 0 |
| 861154 | 2014 | 5/7/2014 | OATS, WHOLE GRAIN | Grains and Grain Products | Oats | IMPORT | Canada | Non-Detect | 2.2 | 0 |
| 861165 | 2014 | 5/7/2014 | SOYBEANS, WHOLE GRAIN | Beans and Legumes | Soybeans | IMPORT | Canada | Non-Detect | 2.2 | 0 |
| 861194 | 2014 | 5/7/2014 | BEAN, DRIED OR PASTE, N.E.C. (VEGETABLE) | Beans and Legumes | Other Beans and Legumes | IMPORT | Philippines | Trace | 1 | |
| 861446 | 2014 | 5/8/2014 | COFFEE, BEANS | Non-Juice Beverages | Coffee | IMPORT | Vietnam | Non-Detect | 2.2 | 0 |
| 862074 | 2014 | 5/8/2014 | OATS, WHOLE GRAIN | Grains and Grain Products | Oats | IMPORT | Canada | Non-Detect | 2.2 | 0 |
| 862097 | 2014 | 5/8/2014 | SOYBEANS (EDIBLE SEED) | Beans and Legumes | Soybeans | IMPORT | Canada | Non-Detect | 2.2 | 0 |
| 861366 | 2014 | 5/8/2014 | RED BEAN, DRIED OR PASTE | Beans and Legumes | Other Beans and Legumes | IMPORT | China | Non-Detect | 1 | 0 |
| 862052 | 2014 | 5/12/2014 | SOYBEANS (SEE INDUSTRY 37 FOR 'BEAN CURD') | Beans and Legumes | Soybeans | IMPORT | Canada | Non-Detect | 2.2 | 0 |
| 862265 | 2014 | 5/14/2014 | SOYBEANS (SEE INDUSTRY 37 FOR 'BEAN CURD') | Beans and Legumes | Soybeans | IMPORT | Canada | Non-Detect | 2.2 | 0 |
| 862460 | 2014 | 5/14/2014 | RAISINS, DRIED OR PASTE | Fruit | Raisins | IMPORT | Chile | Non-Detect | 0.233 | 0 |
| 862915 | 2014 | 5/16/2014 | WHEAT FLOUR, WHOLE | Grains and Grain Products | Wheat | IMPORT | India | Non-Detect | 2.2 | 0 |
| 862919 | 2014 | 5/16/2014 | WHEAT FLOUR, N.E.C. | Grains and Grain Products | Wheat | IMPORT | India | Non-Detect | 2.2 | 0 |
| 863244 | 2014 | 5/20/2014 | CORN, WHOLE GRAIN | Grains and Grain Products | Corn | IMPORT | Vietnam | Non-Detect | 1 | 0 |
| 864078 | 2014 | 5/23/2014 | MIXED BEAN/SEED SPROUTS | Beans and Legumes | Other Beans and Legumes | IMPORT | Thailand | Non-Detect | 2.2 | 0 |
| 863919 | 2014 | 5/27/2014 | SEMOLINA, WHEAT | Grains and Grain Products | Wheat | IMPORT | Morocco | Non-Detect | 1 | 0 |
| 864090 | 2014 | 5/28/2014 | PINTO BEAN, DRIED OR PASTE | Beans and Legumes | Pinto Beans | IMPORT | Mexico | Non-Detect | 0.233 | 0 |
| 864460 | 2014 | 5/29/2014 | BARLEY, WHOLE GRAIN | Grains and Grain Products | Barley | IMPORT | China | Non-Detect | 1 | 0 |
| 859862 | 2014 | 6/2/2014 | MALT, BARLEY | Grains and Grain Products | Barley | IMPORT | United States | Non-Detect | 2.2 | 0 |
| 865134 | 2014 | 6/3/2014 | SPAGHETTI | Processed Food Products | Pasta | IMPORT | Italy | Non-Detect | 1 | 0 |
| 865501 | 2014 | 6/5/2014 | WHEAT FLAKES, PUFFS, KRISPIES, LOOPS, SHREDDED, WHEAT GERM READY TO EAT | Processed Food Products | Breakfast Foods | IMPORT | Canada | Non-Detect | 2.2 | 0 |
| 865586 | 2014 | 6/5/2014 | CORN PUFFS (FLAVORED OR UNFLAVORED), VEGETABLE SNACKS (OTHER THAN PULSES) BAKED | Processed Food Products | Other Corn Products | IMPORT | Poland | Non-Detect | 1 | 0 |
| 866826 | 2014 | 6/6/2014 | COFFEE, BEANS | Non-Juice Beverages | Coffee | IMPORT | Italy | Non-Detect | 1 | 0 |
| 723981 | 2014 | 6/10/2014 | SOYBEANS, WHOLE GRAIN | Beans and Legumes | Soybeans | DOMESTIC | United States | Non-Detect | 2.2 | 0 |
| 866135 | 2014 | 6/10/2014 | COFFEE, BEANS | Non-Juice Beverages | Coffee | IMPORT | Colombia | Non-Detect | 1 | 0 |
| 866566 | 2014 | 6/12/2014 | BLACK BEAN, DRIED OR PASTE | Beans and Legumes | Black Beans | IMPORT | Guatemala | Non-Detect | 0.233 | 0 |
| 866636 | 2014 | 6/12/2014 | MIXED CEREAL FLAKES, SHREDDED AND OTHER FORMS READY TO EAT | Processed Food Products | Breakfast Foods | IMPORT | Germany | Non-Detect | 0.233 | 0 |
| 866761 | 2014 | 6/15/2014 | COFFEE, BEANS | Non-Juice Beverages | Coffee | IMPORT | Colombia | Non-Detect | 1 | 0 |
| 866976 | 2014 | 6/16/2014 | RED BEAN, DRIED OR PASTE | Beans and Legumes | Other Beans and Legumes | IMPORT | Guatemala | Non-Detect | 2.2 | 0 |
| 840811 | 2014 | 6/18/2014 | WHEAT BRAN (HUMAN USE) | Grains and Grain Products | Wheat | DOMESTIC | United States | Non-Detect | 1 | 0 |
| 867997 | 2014 | 6/18/2014 | MIXED GRAIN CEREAL (BABY) | Baby Food Products | Baby Cereals | DOMESTIC | Unknown | Non-Detect | 1 | 0 |
| 868928 | 2014 | 6/20/2014 | BLACKEYE PEAS, DRIED OR PASTE | Beans and Legumes | Blackeye Peas | IMPORT | Thailand | Non-Detect | 2.2 | 0 |
| 868299 | 2014 | 6/23/2014 | COFFEE, BEANS | Non-Juice Beverages | Coffee | IMPORT | Haiti | Non-Detect | 0.233 | 0 |
| 868543 | 2014 | 6/24/2014 | COFFEE, BEANS | Non-Juice Beverages | Coffee | IMPORT | Ethiopia | Non-Detect | 2.2 | 0 |
| 778416 | 2014 | 6/25/2014 | OAT CEREAL (BABY) | Baby Food Products | Baby Cereals | DOMESTIC | United States | Non-Detect | 0.233 | 0 |
| 849076 | 2014 | 6/25/2014 | MIXED GRAIN CEREAL (BABY) | Baby Food Products | Baby Cereals | DOMESTIC | United States | Non-Detect | 0.233 | 0 |
| 868826 | 2014 | 6/25/2014 | PEAS | Beans and Legumes | Peas | IMPORT | Ukraine | Non-Detect | 0.233 | 0 |
| 868940 | 2014 | 6/26/2014 | COFFEE, BEANS | Non-Juice Beverages | Coffee | IMPORT | Kenya | Non-Detect | 2.2 | 0 |
| 869087 | 2014 | 6/26/2014 | KIDNEY BEAN, DRIED OR PASTE | Beans and Legumes | Other Beans and Legumes | IMPORT | Argentina | Non-Detect | 2.2 | 0 |
| 869148 | 2014 | 6/26/2014 | MUNG BEAN, DRIED OR PASTE | Beans and Legumes | Other Beans and Legumes | IMPORT | Australia | Non-Detect | 2.2 | 0 |
| 869177 | 2014 | 6/26/2014 | LUPIN BEAN, DRIED OR PASTE | Beans and Legumes | Other Beans and Legumes | IMPORT | Portugal | Non-Detect | 2.2 | 0 |
| 835049 | 2014 | 7/8/2014 | BLACK EYE BEANS | Beans and Legumes | Blackeye Peas | DOMESTIC | United States | Non-Detect | 0.233 | 0 |
| 870330 | 2014 | 7/8/2014 | COFFEE, BEANS | Non-Juice Beverages | Coffee | IMPORT | Uganda | Non-Detect | 0.233 | 0 |
| 870600 | 2014 | 7/9/2014 | BARLEY, WHOLE GRAIN | Grains and Grain Products | Barley | IMPORT | Hong Kong SAR | Non-Detect | 2.2 | 0 |
| 870869 | 2014 | 7/10/2014 | OAT FLAKES, ROLLED OATS, PUFFS, KRISPIES, LOOPS READY TO EAT | Processed Food Products | Breakfast Foods | IMPORT | Canada | Non-Detect | 1 | 0 |
| 827326 | 2014 | 7/14/2014 | WHEAT FLOUR, ENRICHED (ALL PURPOSE) | Grains and Grain Products | Wheat | DOMESTIC | United States | Non-Detect | 2.2 | 0 |
| 871262 | 2014 | 7/14/2014 | CORN, BOLTED MEAL OR FLOUR | Grains and Grain Products | Corn | IMPORT | Italy | Non-Detect | 1 | 0 |
| 871504 | 2014 | 7/15/2014 | WHEAT FLOUR, DURUM | Grains and Grain Products | Wheat | IMPORT | Canada | Non-Detect | 2.2 | 0 |
| 872013 | 2014 | 7/17/2014 | KIDNEY BEAN | Beans and Legumes | Other Beans and Legumes | IMPORT | Egypt | Non-Detect | 2.2 | 0 |
| 872047 | 2014 | 7/17/2014 | LIMA BEAN, DRIED OR PASTE | Beans and Legumes | Other Beans and Legumes | IMPORT | Peru | Trace | 2.4 | |
| 872589 | 2014 | 7/21/2014 | QUINOA SEED (EDIBLE SEED) | Nuts and Edible Seeds | Other Nuts and Edible Seeds | IMPORT | Peru | Non-Detect | 1 | 0 |
| 872434 | 2014 | 7/22/2014 | OAT FLAKES, ROLLED OATS, PUFFS, KRISPIES, LOOPS READY TO EAT | Processed Food Products | Breakfast Foods | IMPORT | Mexico | Non-Detect | 0.233 | 0 |
| 872561 | 2014 | 7/22/2014 | COFFEE, BEANS | Non-Juice Beverages | Coffee | IMPORT | Peru | Non-Detect | 0.233 | 0 |
| 855644 | 2014 | 7/23/2014 | BUCKWHEAT, WHOLE GRAIN | Grains and Grain Products | Buckwheat | DOMESTIC | United States | Non-Detect | 0.233 | 0 |
| 855645 | 2014 | 7/23/2014 | BUCKWHEAT FLOUR | Grains and Grain Products | Buckwheat | DOMESTIC | United States | Non-Detect | 0.233 | 0 |
| 873006 | 2014 | 7/23/2014 | RAISINS, DRIED OR PASTE | Fruit | Raisins | IMPORT | Australia | Non-Detect | 1 | 0 |
| 872915 | 2014 | 7/24/2014 | COFFEE, BEANS | Non-Juice Beverages | Coffee | IMPORT | Mexico | Non-Detect | 2.2 | 0 |
| 873086 | 2014 | 7/25/2014 | WHEAT, WHOLE GRAIN | Grains and Grain Products | Wheat | IMPORT | Canada | Non-Detect | 1 | 0 |
| 873236 | 2014 | 7/28/2014 | SOYBEAN FLOUR | Beans and Legumes | Soybeans | IMPORT | Belgium | Non-Detect | 2.4 | 0 |
| 839570 | 2014 | 7/28/2014 | WHEAT FLOUR, ENRICHED BROMATED | Grains and Grain Products | Wheat | DOMESTIC | United States | Non-Detect | 0.233 | 0 |
| 839572 | 2014 | 7/28/2014 | BARLEY FLOUR | Grains and Grain Products | Barley | DOMESTIC | United States | Non-Detect | 0.233 | 0 |
| 873280 | 2014 | 7/28/2014 | OATMEAL, REGULAR, FRUIT OR SPICE ADDED, QUICK OR INSTANT COOKING | Processed Food Products | Breakfast Foods | IMPORT | Hong Kong SAR | Non-Detect | 1 | 0 |
| 873353 | 2014 | 7/29/2014 | PEAS | Beans and Legumes | Peas | IMPORT | Canada | Non-Detect | 1 | 0 |
| 851332 | 2014 | 7/30/2014 | RAISINS, DRIED OR PASTE | Fruit | Raisins | DOMESTIC | United States | Non-Detect | 0.233 | 0 |
| 873586 | 2014 | 7/30/2014 | OAT FLAKES, ROLLED OATS, PUFFS, KRISPIES, LOOPS READY TO EAT | Processed Food Products | Breakfast Foods | IMPORT | Canada | Non-Detect | 1 | 0 |
| 857814 | 2014 | 8/1/2014 | BARLEY, WHOLE GRAIN | Grains and Grain Products | Barley | DOMESTIC | United States | Non-Detect | 2.2 | 0 |
| 874546 | 2014 | 8/5/2014 | WHEAT, MILLED (CRUSHED, COARSE GROUND OR CRACKED) | Grains and Grain Products | Wheat | IMPORT | Turkey | Non-Detect | 1 | 0 |
| 874552 | 2014 | 8/5/2014 | WHEAT, MILLED (CRUSHED, COARSE GROUND OR CRACKED) | Grains and Grain Products | Wheat | IMPORT | Turkey | Non-Detect | 1 | 0 |
| 874815 | 2014 | 8/12/2014 | WHEAT FLOUR, WHOLE | Grains and Grain Products | Wheat | DOMESTIC | United States | Non-Detect | 2.4 | 0 |
| 875160 | 2014 | 8/12/2014 | RYE FLOUR | Grains and Grain Products | Rye | DOMESTIC | United States | Non-Detect | 2.4 | 0 |
| 875590 | 2014 | 8/13/2014 | MIXED CEREAL FLAKES, SHREDDED AND OTHER FORMS READY TO EAT | Processed Food Products | Breakfast Foods | IMPORT | United States | Non-Detect | 1 | 0 |
| 875803 | 2014 | 8/13/2014 | QUINOA SEED (EDIBLE SEED) | Nuts and Edible Seeds | Other Nuts and Edible Seeds | IMPORT | Bolivia | Non-Detect | 1 | 0 |
| 875609 | 2014 | 8/13/2014 | WHEAT FLOUR, N.E.C. | Grains and Grain Products | Wheat | IMPORT | Canada | Trace | 1 | |
| 876290 | 2014 | 8/15/2014 | OAT FLAKES, ROLLED OATS, PUFFS, KRISPIES, LOOPS READY TO EAT | Processed Food Products | Breakfast Foods | IMPORT | Ireland | Non-Detect | 2.4 | 0 |
| 876425 | 2014 | 8/19/2014 | COFFEE, BEANS | Non-Juice Beverages | Coffee | IMPORT | Rwanda | Non-Detect | 1 | 0 |
| 876414 | 2014 | 8/19/2014 | RYE FLOUR | Grains and Grain Products | Rye | IMPORT | Russia | Trace | 1 | |
| 852489 | 2014 | 8/21/2014 | OATMEAL, REGULAR, FRUIT OR SPICE ADDED, QUICK OR INSTANT COOKING | Processed Food Products | Breakfast Foods | DOMESTIC | Unknown | Non-Detect | 2.2 | 0 |
| 840812 | 2014 | 8/21/2014 | WHEAT BRAN (HUMAN USE) | Grains and Grain Products | Wheat | DOMESTIC | United States | Non-Detect | 1 | 0 |
| 869984 | 2014 | 8/25/2014 | WHEAT FLOUR, WHOLE | Grains and Grain Products | Wheat | DOMESTIC | United States | Non-Detect | 2.2 | 0 |

| | | | | | | | | | | | |
|---|---|---|---|---|---|---|---|---|---|---|---|
| 869985 | 2014 | 8/25/2014 | WHEAT FLOUR, WHOLE | Grains and Grain Products | Wheat | DOMESTIC | United States | Non-Detect | 2.2 | 0 |
| 852490 | 2014 | 8/26/2014 | MALT, BARLEY | Grains and Grain Products | Barley | DOMESTIC | Unknown | Non-Detect | 2.2 | 0 |
| 815183 | 2014 | 8/26/2014 | BUCKWHEAT FLOUR | Grains and Grain Products | Buckwheat | DOMESTIC | United States | Non-Detect | 0.233 | 0 |
| 877585 | 2014 | 8/26/2014 | CORN, BOLTED MEAL OR FLOUR | Grains and Grain Products | Corn | IMPORT | Brazil | Non-Detect | 1 | 0 |
| 877671 | 2014 | 8/26/2014 | CORN, BOLTED MEAL OR FLOUR | Grains and Grain Products | Corn | IMPORT | India | Non-Detect | 1 | 0 |
| 877797 | 2014 | 8/27/2014 | RAISINS, DRIED OR PASTE | Fruit | Raisins | IMPORT | China | Non-Detect | 0.233 | 0 |
| 869159 | 2014 | 9/4/2014 | CORN, MILLED PRODUCT N.E.C. | Grains and Grain Products | Corn | DOMESTIC | United States | Non-Detect | 0.233 | 0 |
| 879023 | 2014 | 9/4/2014 | CORN CHIPS, FRIED | Processed Food Products | Other Corn Products | IMPORT | Canada | Non-Detect | 1 | 0 |
| 877986 | 2014 | 9/8/2014 | SOY BASE FORMULA PRODUCT, LIQUID CONCENTRATE | Baby Food Products | Baby Formula | DOMESTIC | United States | Non-Detect | 0.233 | 0 |
| 831457 | 2014 | 9/8/2014 | WHEAT FLOUR, PLAIN | Grains and Grain Products | Wheat | DOMESTIC | United States | Non-Detect | 1 | 0 |
| 873795 | 2014 | 9/10/2014 | RICE CEREAL (BABY) | Baby Food Products | Baby Cereals | DOMESTIC | United States | Non-Detect | 0.233 | 0 |
| 879990 | 2014 | 9/11/2014 | OAT FLAKES, ROLLED OATS, PUFFS, KRISPIES, LOOPS READY TO EAT | Processed Food Products | Breakfast Foods | IMPORT | Poland | Non-Detect | 2.4 | 0 |
| 866473 | 2014 | 9/12/2014 | KIDNEY BEAN | Beans and Legumes | Other Beans and Legumes | DOMESTIC | United States | Non-Detect | 1 | 0 |
| 866474 | 2014 | 9/12/2014 | NAVY (WHITE) BEANS, (BAKED BEANS, PORK AND BEANS) | Beans and Legumes | Other Beans and Legumes | DOMESTIC | United States | Non-Detect | 1 | 0 |
| 880594 | 2014 | 9/13/2014 | SOY BASE FORMULA PRODUCT, READY TO FEED | Baby Food Products | Baby Formula | DOMESTIC | United States | Non-Detect | 2.4 | 0 |
| 878426 | 2014 | 9/16/2014 | BUCKWHEAT FLOUR | Grains and Grain Products | Buckwheat | DOMESTIC | United States | Non-Detect | 0.233 | 0 |
| 880810 | 2014 | 9/16/2014 | RAISINS, DRIED OR PASTE | Fruit | Raisins | IMPORT | South Africa | Non-Detect | 1 | 0 |
| 880865 | 2014 | 9/16/2014 | ORIENTAL NOODLES (FLAVORED WITH SHRIMP, CHICKEN, BEEF, LOBSTER, CRAB, PLAIN, ETC.) | Processed Food Products | Pasta | IMPORT | Philippines | Non-Detect | 1 | 0 |
| 860883 | 2014 | 9/17/2014 | SOY BASE FORMULA PRODUCT, READY TO FEED | Baby Food Products | Baby Formula | DOMESTIC | Unknown | Non-Detect | 0.233 | 0 |
| 860884 | 2014 | 9/17/2014 | SOY BASE FORMULA PRODUCT, LIQUID CONCENTRATE | Baby Food Products | Baby Formula | DOMESTIC | United States | Non-Detect | 0.233 | 0 |
| 880928 | 2014 | 9/17/2014 | OAT FLAKES, ROLLED OATS, PUFFS, KRISPIES, LOOPS READY TO EAT | Processed Food Products | Breakfast Foods | IMPORT | Ecuador | Non-Detect | 1 | 0 |
| 877823 | 2014 | 9/18/2014 | RAISINS, DRIED OR PASTE | Fruit | Raisins | DOMESTIC | United States | Non-Detect | 1 | 0 |
| 881557 | 2014 | 9/22/2014 | RAISINS (DRIED GRAPES) (BERRY) | Fruit | Raisins | IMPORT | Greece | Non-Detect | 1 | 0 |

## FY2015 Data

| Sample Number | Fiscal Year | Collection Date | Product Name | Food Category | Food Subcategory | Origin Type | Country of Origin | Non-Detect, Trace, or Positive (>LOQ) | LOQ (ppb) | Amount found (ppb) |
|---|---|---|---|---|---|---|---|---|---|---|
| 883145 | 2015 | 10/3/2014 | RYE FLOUR | Grains and Grain Products | Rye | IMPORT | Canada | Trace | 1 | |
| 884325 | 2015 | 10/7/2014 | WHEAT, MILLED (CRUSHED, COARSE GROUND OR CRACKED) | Grains and Grain Products | Wheat | IMPORT | Turkey | Non-Detect | 2.4 | 0 |
| 883515 | 2015 | 10/7/2014 | RAISINS,  DRIED OR PASTE | Fruit | Raisins | IMPORT | Argentina | Positive | 1 | 12.3 |
| 883731 | 2015 | 10/8/2014 | WHEAT, MILLED (CRUSHED, COARSE GROUND OR CRACKED) | Grains and Grain Products | Wheat | IMPORT | Lebanon | Non-Detect | 1 | 0 |
| 883732 | 2015 | 10/8/2014 | WHEAT, MILLED (CRUSHED, COARSE GROUND OR CRACKED) | Grains and Grain Products | Wheat | IMPORT | Canada | Non-Detect | 1 | 0 |
| 872867 | 2015 | 10/9/2014 | SOYBEANS (EDIBLE SEED) | Beans and Legumes | Soybeans | DOMESTIC | United States | Non-Detect | 2.2 | 0 |
| 883968 | 2015 | 10/9/2014 | COFFEE, BEANS | Non-Juice Beverages | Coffee | IMPORT | Ethiopia | Non-Detect | 1 | 0 |
| 884546 | 2015 | 10/15/2014 | WHEAT FLOUR, ENRICHED SELF-RISING | Grains and Grain Products | Wheat | IMPORT | Canada | Non-Detect | 2.4 | 0 |
| 884799 | 2015 | 10/16/2014 | COFFEE, BEANS | Non-Juice Beverages | Coffee | IMPORT | Kenya | Non-Detect | 1 | 0 |
| 884866 | 2015 | 10/17/2014 | OATMEAL, REGULAR, FRUIT OR SPICE ADDED, QUICK OR INSTANT COOKING | Processed Food Products | Breakfast Foods | IMPORT | Nicaragua | Non-Detect | 2.4 | 0 |
| 884847 | 2015 | 10/17/2014 | COFFEE, BEANS | Non-Juice Beverages | Coffee | IMPORT | Haiti | Non-Detect | 0.233 | 0 |
| 884895 | 2015 | 10/17/2014 | CORN CHIPS, FRIED | Processed Food Products | Other Corn Products | IMPORT | Philippines | Non-Detect | 1 | 0 |
| 884924 | 2015 | 10/19/2014 | BARLEY, WHOLE GRAIN | Grains and Grain Products | Barley | IMPORT | Canada | Non-Detect | 1 | 0 |
| 872815 | 2015 | 10/20/2014 | WHEAT, WHOLE GRAIN | Grains and Grain Products | Wheat | DOMESTIC | United States | Non-Detect | 1 | 0 |
| 885487 | 2015 | 10/22/2014 | COFFEE, BEANS | Non-Juice Beverages | Coffee | IMPORT | Peru | Non-Detect | 2.4 | 0 |
| 885572 | 2015 | 10/22/2014 | OATMEAL, REGULAR, FRUIT OR SPICE ADDED, QUICK OR INSTANT COOKING | Processed Food Products | Breakfast Foods | IMPORT | Canada | Non-Detect | 2.4 | 0 |
| 885846 | 2015 | 10/22/2014 | VANILLA FILLED/ICED COOKIES, BISCUITS AND WAFERS | Processed Food Products | Other Bakery Products | IMPORT | Indonesia | Non-Detect | 1 | 0 |
| 857273 | 2015 | 10/23/2014 | RAISINS, DRIED OR PASTE | Fruit | Raisins | DOMESTIC | United States | Non-Detect | 1 | 0 |
| 885954 | 2015 | 10/24/2014 | RED BEAN, DRIED OR PASTE | Beans and Legumes | Other Beans and Legumes | IMPORT | Guatemala | Non-Detect | 2.4 | 0 |
| 886991 | 2015 | 10/24/2014 | WHEAT, WHOLE GRAIN | Grains and Grain Products | Wheat | DOMESTIC | United States | Non-Detect | 0.233 | 0 |
| 886993 | 2015 | 10/24/2014 | OATS, WHOLE GRAIN | Grains and Grain Products | Oats | DOMESTIC | United States | Non-Detect | 0.233 | 0 |
| 886085 | 2015 | 10/27/2014 | GRAM FLOUR | Beans and Legumes | Garbanzo Beans | IMPORT | India | Non-Detect | 2.4 | 0 |
| 886191 | 2015 | 10/27/2014 | LENTILS, DRIED OR PASTE | Beans and Legumes | Lentils | IMPORT | Turkey | Non-Detect | 2.4 | 0 |
| 861174 | 2015 | 10/27/2014 | WHEAT CEREAL (BABY) | Baby Food Products | Baby Cereals | DOMESTIC | United States | Non-Detect | 0.233 | 0 |
| 886375 | 2015 | 10/27/2014 | COFFEE, BEANS | Non-Juice Beverages | Coffee | IMPORT | Brazil | Non-Detect | 1 | 0 |
| 886496 | 2015 | 10/28/2014 | MUNG BEAN, DRIED OR PASTE | Beans and Legumes | Other Beans and Legumes | IMPORT | China | Non-Detect | 2.4 | 0 |
| 886688 | 2015 | 10/29/2014 | RAISINS (DRIED GRAPES) (BERRY) | Fruit | Raisins | IMPORT | Argentina | Positive | 5.49 | 30.1 |
| 887020 | 2015 | 10/30/2014 | COFFEE, BEANS | Non-Juice Beverages | Coffee | IMPORT | Peru | Non-Detect | 2.4 | 0 |
| 886929 | 2015 | 10/30/2014 | COFFEE, BEANS | Non-Juice Beverages | Coffee | IMPORT | Colombia | Non-Detect | 1 | 0 |
| 887164 | 2015 | 11/2/2014 | COFFEE, BEANS | Non-Juice Beverages | Coffee | IMPORT | Vietnam | Trace | 1 | |
| 871329 | 2015 | 11/3/2014 | MALT, BARLEY | Grains and Grain Products | Barley | IMPORT | Germany | Non-Detect | 2.2 | 0 |
| 871330 | 2015 | 11/3/2014 | MALT, BARLEY | Grains and Grain Products | Barley | IMPORT | United Kingdom | Non-Detect | 2.2 | 0 |
| 887267 | 2015 | 11/3/2014 | BUCKWHEAT, WHOLE GRAIN | Grains and Grain Products | Buckwheat | IMPORT | Canada | Non-Detect | 1 | 0 |
| 887538 | 2015 | 11/4/2014 | BARLEY, WHOLE GRAIN | Grains and Grain Products | Barley | IMPORT | Hong Kong SAR | Non-Detect | 1 | 0 |
| 887575 | 2015 | 11/4/2014 | COFFEE CAKE WITHOUT CUSTARD OR CREAM FILLING | Processed Food Products | Other Bakery Products | IMPORT | Canada | Non-Detect | 1 | 0 |
| 887726 | 2015 | 11/5/2014 | SOYBEANS, WHOLE GRAIN | Beans and Legumes | Soybeans | IMPORT | Canada | Non-Detect | 1 | 0 |
| 887733 | 2015 | 11/5/2014 | MALT, BARLEY | Grains and Grain Products | Barley | IMPORT | Canada | Non-Detect | 1 | 0 |
| 887869 | 2015 | 11/6/2014 | WHITE BREAD/ROLLS/BUNS | Processed Food Products | Bread | IMPORT | Mexico | Non-Detect | 0.233 | 0 |
| 888033 | 2015 | 11/6/2014 | RAISINS (DRIED GRAPES) (BERRY) | Fruit | Raisins | IMPORT | Greece | Non-Detect | 1 | 0 |
| 888086 | 2015 | 11/6/2014 | COFFEE, BEANS | Non-Juice Beverages | Coffee | IMPORT | Papua New Guinea | Non-Detect | 1 | 0 |
| 888120 | 2015 | 11/7/2014 | RYE FLOUR | Grains and Grain Products | Rye | IMPORT | Belgium | Non-Detect | 2.4 | 0 |
| 888167 | 2015 | 11/7/2014 | COFFEE, BEANS | Non-Juice Beverages | Coffee | IMPORT | Nicaragua | Non-Detect | 0.233 | 0 |
| 888331 | 2015 | 11/10/2014 | PASTRY SHELL DOUGH OR BATTER WITHOUT CUSTARD OR CREAM FILLING | Processed Food Products | Other Bakery Products | IMPORT | Argentina | Non-Detect | 1 | 0 |
| 869990 | 2015 | 11/12/2014 | WHEAT FLOUR, N.E.C. | Grains and Grain Products | Wheat | DOMESTIC | United States | Non-Detect | 0.233 | 0 |
| 888387 | 2015 | 11/12/2014 | COFFEE, BEANS | Non-Juice Beverages | Coffee | IMPORT | Uganda | Non-Detect | 0.233 | 0 |
| 836281 | 2015 | 11/12/2014 | RYE FLOUR | Grains and Grain Products | Rye | DOMESTIC | United States | Non-Detect | 1 | 0 |
| 861640 | 2015 | 11/13/2014 | OAT FLOUR | Grains and Grain Products | Oats | DOMESTIC | United States | Non-Detect | 2.2 | 0 |
| 888622 | 2015 | 11/13/2014 | COFFEE, BEANS | Non-Juice Beverages | Coffee | IMPORT | Uganda | Trace | 8 | |
| 888854 | 2015 | 11/14/2014 | COFFEE, BEANS | Non-Juice Beverages | Coffee | IMPORT | Colombia | Non-Detect | 0.233 | 0 |
| 888909 | 2015 | 11/14/2014 | CORN FLAKES, PUFFS, KRISPIES, LOOPS READY TO EAT | Processed Food Products | Other Corn Products | IMPORT | Guatemala | Non-Detect | 0.233 | 0 |
| 888913 | 2015 | 11/14/2014 | KIDNEY BEAN | Beans and Legumes | Other Beans and Legumes | IMPORT | Guatemala | Non-Detect | 0.233 | 0 |
| 888904 | 2015 | 11/14/2014 | WHEAT FLOUR, DURUM | Grains and Grain Products | Wheat | IMPORT | Italy | Non-Detect | 1 | 0 |
| 889083 | 2015 | 11/17/2014 | ALMOND, SHELLED | Nuts and Edible Seeds | Almonds | IMPORT | Spain | Non-Detect | 1 | 0 |
| 889553 | 2015 | 11/18/2014 | FILLED/ICED COOKIES, BISCUITS AND WAFERS, N.E.C. | Processed Food Products | Other Bakery Products | IMPORT | Netherlands | Non-Detect | 1 | 0 |
| 845565 | 2015 | 11/19/2014 | WHEAT FLOUR, GLUTEN | Grains and Grain Products | Wheat | IMPORT | Canada | Non-Detect | 2.2 | 0 |
| 889524 | 2015 | 11/19/2014 | FLOURS AND MEALS N.E.C. | Grains and Grain Products | Other Grains | IMPORT | United Kingdom | Non-Detect | 2.4 | 0 |
| 889186 | 2015 | 11/24/2014 | WHEAT, WHOLE GRAIN | Grains and Grain Products | Wheat | DOMESTIC | United States | Non-Detect | 4.99 | 0 |
| 889188 | 2015 | 11/24/2014 | WHEAT, WHOLE GRAIN | Grains and Grain Products | Wheat | DOMESTIC | United States | Non-Detect | 4.99 | 0 |
| 889189 | 2015 | 11/24/2014 | WHEAT, WHOLE GRAIN | Grains and Grain Products | Wheat | DOMESTIC | United States | Non-Detect | 5 | 0 |
| 890239 | 2015 | 11/24/2014 | MIXED PLAIN COOKIES, BISCUITS AND WAFERS | Processed Food Products | Other Bakery Products | IMPORT | Mexico | Non-Detect | 5 | 0 |
| 890573 | 2015 | 11/28/2014 | WHEAT FLOUR, N.E.C. | Grains and Grain Products | Wheat | IMPORT | India | Non-Detect | 1 | 0 |
| 884162 | 2015 | 12/1/2014 | WHEAT FLAKES, PUFFS, KRISPIES, LOOPS, SHREDDED, WHEAT GERM READY TO EAT | Processed Food Products | Breakfast Foods | DOMESTIC | United States | Non-Detect | 1 | 0 |
| 891400 | 2015 | 12/4/2014 | WHEAT FLOUR, WHOLE | Grains and Grain Products | Wheat | IMPORT | India | Non-Detect | 1 | 0 |
| 891502 | 2015 | 12/5/2014 | LIMA BEAN, DRIED OR PASTE | Beans and Legumes | Other Beans and Legumes | IMPORT | Peru | Non-Detect | 2.4 | 0 |
| 891457 | 2015 | 12/5/2014 | WHEAT FLOUR, WHOLE | Grains and Grain Products | Wheat | IMPORT | India | Non-Detect | 1 | 0 |
| 891575 | 2015 | 12/5/2014 | COFFEE, BEANS | Non-Juice Beverages | Coffee | IMPORT | Honduras | Non-Detect | 1 | 0 |
| 891589 | 2015 | 12/8/2014 | WHEAT FLOUR, WHOLE | Grains and Grain Products | Wheat | IMPORT | India | Non-Detect | 1 | 0 |
| 891877 | 2015 | 12/8/2014 | WHEAT FLOUR, WHOLE | Grains and Grain Products | Wheat | IMPORT | India | Non-Detect | 1 | 0 |
| 869608 | 2015 | 12/9/2014 | MALT, BARLEY | Grains and Grain Products | Barley | DOMESTIC | United States | Non-Detect | 2.2 | 0 |
| 891895 | 2015 | 12/9/2014 | WHEAT, WHOLE GRAIN | Grains and Grain Products | Wheat | IMPORT | Canada | Non-Detect | 1 | 0 |
| 892028 | 2015 | 12/9/2014 | COFFEE, BEANS | Non-Juice Beverages | Coffee | IMPORT | Nicaragua | Positive | 5 | 5.78 |
| 892073 | 2015 | 12/10/2014 | BLACKEYED PEA | Beans and Legumes | Blackeye Peas | IMPORT | Madagascar | Non-Detect | 2.4 | 0 |
| 892202 | 2015 | 12/11/2014 | CORN CHIPS, BAKED | Processed Food Products | Other Corn Products | IMPORT | Korea (the Republic of) | Non-Detect | 1 | 0 |
| 892763 | 2015 | 12/15/2014 | TAMALE (MULTIPLE FOOD SPECIALITIES, SIDE DISHES AND DESSERTS) | Processed Food Products | Other Processed Foods | IMPORT | El Salvador | Non-Detect | 1 | 0 |
| 892767 | 2015 | 12/15/2014 | TORTILLAS | Processed Food Products | Other Corn Products | IMPORT | El Salvador | Non-Detect | 1 | 0 |
| 892804 | 2015 | 12/16/2014 | BLACKEYED PEA | Beans and Legumes | Blackeye Peas | DOMESTIC | United States | Non-Detect | 2.4 | 0 |
| 892769 | 2015 | 12/16/2014 | COFFEE, GROUND | Non-Juice Beverages | Coffee | IMPORT | Canada | Non-Detect | 1 | 0 |
| 892843 | 2015 | 12/16/2014 | FARINA, WHEAT | Grains and Grain Products | Wheat | IMPORT | Canada | Non-Detect | 1 | 0 |
| 893708 | 2015 | 12/23/2014 | COFFEE, BEANS | Non-Juice Beverages | Coffee | IMPORT | Canada | Non-Detect | 1 | 0 |
| 893780 | 2015 | 12/28/2014 | FAVA BEAN, DRIED OR PASTE | Beans and Legumes | Other Beans and Legumes | IMPORT | Canada | Non-Detect | 1 | 0 |
| 893848 | 2015 | 12/29/2014 | VANILLA PLAIN COOKIE, BISCUIT AND WAFER | Processed Food Products | Other Bakery Products | IMPORT | Pakistan | Non-Detect | 2.4 | 0 |
| 893839 | 2015 | 12/29/2014 | COFFEE, BEANS | Non-Juice Beverages | Coffee | IMPORT | Italy | Non-Detect | 0.233 | 0 |
| 894055 | 2015 | 1/2/2015 | WHEAT, MILLED (CRUSHED, COARSE GROUND OR CRACKED) | Grains and Grain Products | Wheat | IMPORT | Turkey | Non-Detect | 2.3 | 0 |

| | | | | | | | | | | |
|---|---|---|---|---|---|---|---|---|---|---|
| 894058 | 2015 | 1/2/2015 | WHEAT, MILLED (CRUSHED, COARSE GROUND OR CRACKED) | Grains and Grain Products | Wheat | IMPORT | Turkey | Non-Detect | 2.3 | 0 |
| 894075 | 2015 | 1/2/2015 | WHEAT, MILLED (CRUSHED, COARSE GROUND OR CRACKED) | Grains and Grain Products | Wheat | IMPORT | Turkey | Non-Detect | 2.3 | 0 |
| 894107 | 2015 | 1/5/2015 | COFFEE, BEANS | Non-Juice Beverages | Coffee | IMPORT | Brazil | Positive | 2.3 | 12.6 |
| 881583 | 2015 | 1/6/2015 | OAT FLAKES, ROLLED OATS, PUFFS, KRISPIES, LOOPS READY TO EAT | Processed Food Products | Breakfast Foods | DOMESTIC | United States | Non-Detect | 2.3 | 0 |
| 894349 | 2015 | 1/7/2015 | OAT FLOUR | Grains and Grain Products | Oats | IMPORT | Canada | Non-Detect | 1 | 0 |
| 894562 | 2015 | 1/8/2015 | KIDNEY BEAN | Beans and Legumes | Other Beans and Legumes | IMPORT | Honduras | Non-Detect | 2.3 | 0 |
| 894500 | 2015 | 1/8/2015 | KIDNEY BEAN | Beans and Legumes | Other Beans and Legumes | IMPORT | Nicaragua | Non-Detect | 0.233 | 0 |
| 894614 | 2015 | 1/9/2015 | NUT PLAIN COOKIES, BISCUITS AND WAFERS | Processed Food Products | Other Bakery Products | IMPORT | Mexico | Non-Detect | 0.233 | 0 |
| 895222 | 2015 | 1/15/2015 | COFFEE, BEANS | Non-Juice Beverages | Coffee | IMPORT | Colombia | Non-Detect | 2.3 | 0 |
| 895215 | 2015 | 1/15/2015 | RICE, CULTIVATED, WHOLE GRAIN | Grains and Grain Products | Rice | IMPORT | Canada | Non-Detect | 1 | 0 |
| 895398 | 2015 | 1/16/2015 | MALT, BARLEY | Grains and Grain Products | Barley | IMPORT | Germany | Non-Detect | 2.3 | 0 |
| 895894 | 2015 | 1/16/2015 | RAISINS, DRIED OR PASTE | Fruit | Raisins | IMPORT | Turkey | Positive | 1 | 13.8 |
| 843079 | 2015 | 1/22/2015 | CORN FLAKES, PUFFS, KRISPIES, LOOPS READY TO EAT | Processed Food Products | Other Corn Products | DOMESTIC | United States | Non-Detect | 2.3 | 0 |
| 892808 | 2015 | 1/22/2015 | WHEAT FLOUR, ENRICHED (ALL PURPOSE) | Grains and Grain Products | Wheat | DOMESTIC | United States | Non-Detect | 2.3 | 0 |
| 895990 | 2015 | 1/22/2015 | COFFEE, BEANS | Non-Juice Beverages | Coffee | IMPORT | Tanzania, United Republic Of | Non-Detect | 0.233 | 0 |
| 895939 | 2015 | 1/23/2015 | RYE FLOUR | Grains and Grain Products | Rye | DOMESTIC | United States | Non-Detect | 0.233 | 0 |
| 896154 | 2015 | 1/23/2015 | COFFEE, GROUND | Non-Juice Beverages | Coffee | IMPORT | Jamaica | Non-Detect | 0.233 | 0 |
| 880466 | 2015 | 1/28/2015 | WHEAT, WHOLE GRAIN | Grains and Grain Products | Wheat | IMPORT | United States | Non-Detect | 0.233 | 0 |
| 880467 | 2015 | 1/28/2015 | WHEAT FLOUR, N.E.C. | Grains and Grain Products | Wheat | DOMESTIC | United States | Non-Detect | 0.233 | 0 |
| 896532 | 2015 | 1/28/2015 | KIDNEY BEAN | Beans and Legumes | Other Beans and Legumes | IMPORT | Nicaragua | Non-Detect | 0.233 | 0 |
| 896749 | 2015 | 1/29/2015 | COFFEE, BEANS | Non-Juice Beverages | Coffee | IMPORT | Italy | Non-Detect | 1 | 0 |
| 897260 | 2015 | 1/30/2015 | COFFEE, BEANS | Non-Juice Beverages | Coffee | IMPORT | Mexico | Non-Detect | 0.233 | 0 |
| 896819 | 2015 | 1/30/2015 | OAT FLAKES, ROLLED OATS, PUFFS, KRISPIES, LOOPS READY TO EAT | Processed Food Products | Breakfast Foods | IMPORT | Canada | Non-Detect | 1 | 0 |
| 896927 | 2015 | 2/2/2015 | OATMEAL, REGULAR, FRUIT OR SPICE ADDED, QUICK OR INSTANT COOKING | Processed Food Products | Breakfast Foods | IMPORT | Canada | Non-Detect | 0.233 | 0 |
| 897165 | 2015 | 2/3/2015 | COFFEE, GROUND | Non-Juice Beverages | Coffee | IMPORT | Honduras | Non-Detect | 0.233 | 0 |
| 897294 | 2015 | 2/3/2015 | COFFEE, BEANS | Non-Juice Beverages | Coffee | IMPORT | Mexico | Non-Detect | 0.233 | 0 |
| 897771 | 2015 | 2/5/2015 | COFFEE, BEANS | Non-Juice Beverages | Coffee | IMPORT | Brazil | Non-Detect | 2.3 | 0 |
| 897854 | 2015 | 2/5/2015 | COFFEE, BEANS | Non-Juice Beverages | Coffee | IMPORT | Brazil | Non-Detect | 2.3 | 0 |
| 897811 | 2015 | 2/5/2015 | COFFEE, BEANS | Non-Juice Beverages | Coffee | IMPORT | Guatemala | Non-Detect | 1 | 0 |
| 894618 | 2015 | 2/9/2015 | OAT CEREAL (BABY) | Baby Food Products | Baby Cereals | DOMESTIC | United States | Trace | 4.99 | |
| 894720 | 2015 | 2/10/2015 | MALT, BARLEY | Grains and Grain Products | Barley | DOMESTIC | United States | Non-Detect | 0.233 | 0 |
| 898711 | 2015 | 2/11/2015 | COFFEE, BEANS | Non-Juice Beverages | Coffee | IMPORT | Brazil | Non-Detect | 1 | 0 |
| 898736 | 2015 | 2/12/2015 | OATMEAL, REGULAR, FRUIT OR SPICE ADDED, QUICK OR INSTANT COOKING | Processed Food Products | Breakfast Foods | IMPORT | Australia | Non-Detect | 0.233 | 0 |
| 898907 | 2015 | 2/12/2015 | OATMEAL, REGULAR, FRUIT OR SPICE ADDED, QUICK OR INSTANT COOKING | Processed Food Products | Breakfast Foods | IMPORT | India | Non-Detect | 0.233 | 0 |
| 898770 | 2015 | 2/13/2015 | BLACK EYE BEANS | Beans and Legumes | Blackeye Peas | IMPORT | Guatemala | Non-Detect | 0.233 | 0 |
| 898971 | 2015 | 2/17/2015 | CORN, DRIED OR PASTE | Grains and Grain Products | Corn | IMPORT | Vietnam | Non-Detect | 1 | 0 |
| 897591 | 2015 | 2/19/2015 | BLACKEYED PEA | Beans and Legumes | Blackeye Peas | DOMESTIC | United States | Non-Detect | 2.3 | 0 |
| 897030 | 2015 | 2/24/2015 | WHEAT, WHOLE GRAIN | Grains and Grain Products | Wheat | DOMESTIC | United States | Trace | 5 | |
| 847982 | 2015 | 2/26/2015 | RAISINS, DRIED OR PASTE | Fruit | Raisins | DOMESTIC | United States | Non-Detect | 1 | 0 |
| 900729 | 2015 | 2/27/2015 | GARBANZO BEAN | Beans and Legumes | Garbanzo Beans | IMPORT | Mexico | Non-Detect | 0.233 | 0 |
| 900750 | 2015 | 3/2/2015 | BARLEY, WHOLE GRAIN | Grains and Grain Products | Barley | IMPORT | Ethiopia | Non-Detect | 2.3 | 0 |
| 874800 | 2015 | 3/2/2015 | SOY BASE FORMULA PRODUCT, READY TO FEED | Baby Food Products | Baby Formula | DOMESTIC | United States | Non-Detect | 4.99 | 0 |
| 901210 | 2015 | 3/3/2015 | COFFEE, BEANS | Non-Juice Beverages | Coffee | IMPORT | Vietnam | Non-Detect | 1 | 0 |
| 901030 | 2015 | 3/3/2015 | WHEAT FLOUR, BROMATED | Grains and Grain Products | Wheat | IMPORT | India | Positive | 2.6 | 5.7 |
| 897593 | 2015 | 3/4/2015 | OAT CEREAL (BABY) | Baby Food Products | Baby Cereals | DOMESTIC | United States | Non-Detect | 2.3 | 0 |
| 901083 | 2015 | 3/4/2015 | FLOURS AND MEALS N.E.C. | Grains and Grain Products | Other Grains | IMPORT | Kenya | Non-Detect | 2.3 | 0 |
| 901207 | 2015 | 3/5/2015 | CREAM WHEAT, QUICK OR INSTANT COOKING | Processed Food Products | Breakfast Foods | IMPORT | India | Non-Detect | 1 | 0 |
| 901279 | 2015 | 3/5/2015 | OATMEAL, REGULAR, FRUIT OR SPICE ADDED, QUICK OR INSTANT COOKING | Processed Food Products | Breakfast Foods | IMPORT | Canada | Non-Detect | 1 | 0 |
| 901379 | 2015 | 3/5/2015 | MIXED CEREAL FLAKES, SHREDDED AND OTHER FORMS READY TO EAT | Processed Food Products | Breakfast Foods | IMPORT | Canada | Non-Detect | 1 | 0 |
| 901641 | 2015 | 3/9/2015 | CORN, BOLTED MEAL OR FLOUR | Grains and Grain Products | Corn | IMPORT | China | Non-Detect | 1 | 0 |
| 901726 | 2015 | 3/10/2015 | WHEAT FLOUR, DURUM | Grains and Grain Products | Wheat | IMPORT | Italy | Non-Detect | 2.3 | 0 |
| 867800 | 2015 | 3/10/2015 | BEANS, N.E.C. (VEGETABLE) | Beans and Legumes | Other Beans and Legumes | DOMESTIC | United States | Non-Detect | 1 | 0 |
| 867808 | 2015 | 3/10/2015 | BEANS, N.E.C. (VEGETABLE) | Beans and Legumes | Other Beans and Legumes | DOMESTIC | United States | Non-Detect | 1 | 0 |
| 901927 | 2015 | 3/10/2015 | CORN BEVERAGE BASE | Non-Juice Beverages | Other Beverages | IMPORT | El Salvador | Non-Detect | 1 | 0 |
| 867798 | 2015 | 3/11/2015 | OAT CEREAL (BABY) | Baby Food Products | Baby Cereals | DOMESTIC | United States | Non-Detect | 1 | 0 |
| 867801 | 2015 | 3/11/2015 | BREAKFAST FOODS READY TO EAT, N.E.C. | Processed Food Products | Breakfast Foods | DOMESTIC | United States | Non-Detect | 1 | 0 |
| 901959 | 2015 | 3/11/2015 | COFFEE, BEANS | Non-Juice Beverages | Coffee | IMPORT | Colombia | Non-Detect | 1 | 0 |
| 902178 | 2015 | 3/11/2015 | BLACKEYE PEAS, DRIED OR PASTE | Beans and Legumes | Blackeye Peas | IMPORT | China | Non-Detect | 1 | 0 |
| 902519 | 2015 | 3/13/2015 | COFFEE, BEANS | Non-Juice Beverages | Coffee | IMPORT | Colombia | Non-Detect | 12.15 | 0 |
| 902553 | 2015 | 3/15/2015 | RAISINS, DRIED OR PASTE | Fruit | Raisins | IMPORT | Uzbekistan | Positive | 1 | 2.75 |
| 903389 | 2015 | 3/16/2015 | FAVA BEAN | Beans and Legumes | Other Beans and Legumes | IMPORT | Lebanon | Non-Detect | 2.3 | 0 |
| 845569 | 2015 | 3/17/2015 | KIDNEY BEAN | Beans and Legumes | Other Beans and Legumes | DOMESTIC | United States | Non-Detect | 2.3 | 0 |
| 845570 | 2015 | 3/17/2015 | GREEN PEAS | Beans and Legumes | Peas | DOMESTIC | United States | Non-Detect | 2.3 | 0 |
| 902665 | 2015 | 3/17/2015 | CORN, BOLTED MEAL OR FLOUR | Grains and Grain Products | Corn | DOMESTIC | United States | Non-Detect | 1 | 0 |
| 902976 | 2015 | 3/17/2015 | BARLEY, WHOLE GRAIN | Grains and Grain Products | Barley | IMPORT | China | Non-Detect | 1 | 0 |
| 903321 | 2015 | 3/19/2015 | BLACKEYE PEAS, DRIED OR PASTE | Beans and Legumes | Blackeye Peas | IMPORT | Canada | Non-Detect | 2.3 | 0 |
| 903324 | 2015 | 3/19/2015 | LENTILS, DRIED OR PASTE | Beans and Legumes | Lentils | IMPORT | Canada | Non-Detect | 2.3 | 0 |
| 903807 | 2015 | 3/19/2015 | BLACK BEAN, DRIED OR PASTE | Beans and Legumes | Black Beans | IMPORT | China | Non-Detect | 2.3 | 0 |
| 873272 | 2015 | 3/19/2015 | CORN, ENRICHED MEAL | Grains and Grain Products | Corn | DOMESTIC | Unknown | Non-Detect | 0.233 | 0 |
| 902443 | 2015 | 3/19/2015 | MALT, BARLEY | Grains and Grain Products | Barley | DOMESTIC | United States | Non-Detect | 1 | 0 |
| 903269 | 2015 | 3/19/2015 | BLACK EYE BEANS | Beans and Legumes | Blackeye Peas | IMPORT | Canada | Non-Detect | 1 | 0 |
| 903890 | 2015 | 3/23/2015 | RYE FLOUR | Grains and Grain Products | Rye | IMPORT | Canada | Non-Detect | 1 | 0 |
| 903982 | 2015 | 3/24/2015 | KIDNEY BEAN | Beans and Legumes | Other Beans and Legumes | IMPORT | Canada | Non-Detect | 1 | 0 |
| 902824 | 2015 | 3/25/2015 | OATS, WHOLE GRAIN | Grains and Grain Products | Oats | DOMESTIC | United States | Non-Detect | 0.233 | 0 |
| 902825 | 2015 | 3/25/2015 | CORN, PLAIN MEAL | Grains and Grain Products | Corn | DOMESTIC | United States | Non-Detect | 0.233 | 0 |
| 904115 | 2015 | 3/25/2015 | BARLEY, WHOLE GRAIN | Grains and Grain Products | Barley | IMPORT | Japan | Non-Detect | 1 | 0 |
| 874804 | 2015 | 3/26/2015 | VEGETABLE TURKEY DINNER (BABY) | Baby Food Products | Other Baby Foods | DOMESTIC | United States | Non-Detect | 2.3 | 0 |
| 715310 | 2015 | 3/26/2015 | WHEAT FLOUR, PLAIN | Grains and Grain Products | Wheat | DOMESTIC | United States | Non-Detect | 0.233 | 0 |
| 904292 | 2015 | 3/26/2015 | BREADCRUMBS | Processed Food Products | Bread | IMPORT | China | Non-Detect | 1 | 0 |
| 904323 | 2015 | 3/26/2015 | CORN FLAKES, PUFFS, KRISPIES, LOOPS READY TO EAT | Processed Food Products | Other Corn Products | IMPORT | India | Non-Detect | 1 | 0 |
| 897031 | 2015 | 3/30/2015 | BUCKWHEAT FLOUR | Grains and Grain Products | Buckwheat | DOMESTIC | United States | Non-Detect | 0.233 | 0 |
| 904699 | 2015 | 3/30/2015 | KIDNEY BEAN | Beans and Legumes | Other Beans and Legumes | IMPORT | Nicaragua | Non-Detect | 0.233 | 0 |
| 888218 | 2015 | 3/31/2015 | WHEAT FLOUR, WHOLE | Grains and Grain Products | Wheat | DOMESTIC | United States | Non-Detect | 0.233 | 0 |
| 904871 | 2015 | 3/31/2015 | OAT FLAKES, ROLLED OATS, PUFFS, KRISPIES, LOOPS READY TO EAT | Processed Food Products | Breakfast Foods | IMPORT | Mexico | Non-Detect | 0.233 | 0 |

| | | | | | | | | | | |
|---|---|---|---|---|---|---|---|---|---|---|
| 904885 | 2015 | 3/31/2015 | CORN, WHOLE GRAIN | Grains and Grain Products | Corn | IMPORT | Mexico | Non-Detect | 0.233 | 0 |
| 904895 | 2015 | 3/31/2015 | BLACK BEAN, DRIED OR PASTE | Beans and Legumes | Black Beans | IMPORT | Mexico | Non-Detect | 0.233 | 0 |
| 905245 | 2015 | 4/1/2015 | COFFEE, BEANS | Non-Juice Beverages | Coffee | IMPORT | Brazil | Non-Detect | 1 | 0 |
| 906129 | 2015 | 4/3/2015 | CORN, BOLTED MEAL OR FLOUR | Grains and Grain Products | Corn | IMPORT | Ghana | Trace | 1 | |
| 906130 | 2015 | 4/3/2015 | FLOURS AND MEALS N.E.C. | Grains and Grain Products | Other Grains | IMPORT | Ghana | Trace | 1 | |
| 906191 | 2015 | 4/8/2015 | KIDNEY BEAN | Beans and Legumes | Other Beans and Legumes | IMPORT | Nicaragua | Non-Detect | 0.233 | 0 |
| 906486 | 2015 | 4/9/2015 | BLACK BEAN, DRIED OR PASTE | Beans and Legumes | Black Beans | IMPORT | Vietnam | Non-Detect | 1 | 0 |
| 906725 | 2015 | 4/13/2015 | COFFEE, BEANS | Non-Juice Beverages | Coffee | IMPORT | Nicaragua | Non-Detect | 12.15 | 0 |
| 906735 | 2015 | 4/13/2015 | COFFEE, BEANS | Non-Juice Beverages | Coffee | IMPORT | Brazil | Non-Detect | 12.15 | 0 |
| 907236 | 2015 | 4/15/2015 | COFFEE, GROUND | Non-Juice Beverages | Coffee | IMPORT | El Salvador | Non-Detect | 9.225 | 0 |
| 908058 | 2015 | 4/21/2015 | BUCKWHEAT, WHOLE GRAIN | Grains and Grain Products | Buckwheat | IMPORT | China | Non-Detect | 0.233 | 0 |
| 908181 | 2015 | 4/22/2015 | COCOA BEAN N.E.C. | Candy and Chocolate Products | Other Chocolate Products | IMPORT | Guatemala | Non-Detect | 2.3 | 0 |
| 908471 | 2015 | 4/23/2015 | COFFEE, BEANS | Non-Juice Beverages | Coffee | IMPORT | Mexico | Non-Detect | 12.15 | 0 |
| 908709 | 2015 | 4/27/2015 | KIDNEY BEAN | Beans and Legumes | Other Beans and Legumes | IMPORT | Nicaragua | Non-Detect | 2.3 | 0 |
| 909076 | 2015 | 4/28/2015 | CEREAL PREPARATIONS NOT ELSEWHERE MENTIONED, N.E.C. | Processed Food Products | Breakfast Foods | IMPORT | Mexico | Non-Detect | 5 | 0 |
| 894094 | 2015 | 4/29/2015 | WHEAT, WHOLE GRAIN | Grains and Grain Products | Wheat | DOMESTIC | United States | Non-Detect | 1 | 0 |
| 894095 | 2015 | 4/29/2015 | BARLEY, WHOLE GRAIN | Grains and Grain Products | Barley | DOMESTIC | United States | Non-Detect | 1 | 0 |
| 909274 | 2015 | 4/29/2015 | BEAN, DRIED OR PASTE, N.E.C. (VEGETABLE) | Beans and Legumes | Other Beans and Legumes | IMPORT | Philippines | Trace | 1 | |
| 909375 | 2015 | 4/29/2015 | CORN (WHOLE KERNEL) SNACKS, VEGETABLE SNACKS (OTHER THAN PULSES) BAKED | Processed Food Products | Other Corn Products | IMPORT | Philippines | Trace | 1 | |
| 909575 | 2015 | 5/1/2015 | RAISINS, DRIED OR PASTE | Fruit | Raisins | IMPORT | Chile | Non-Detect | 5 | 0 |
| 909790 | 2015 | 5/1/2015 | CHOCOLATE AND COCOA BEVERAGE BASE, N.E.C. | Candy and Chocolate Products | Other Chocolate Products | IMPORT | United Kingdom | Non-Detect | 5 | 0 |
| 909671 | 2015 | 5/3/2015 | COFFEE, BEANS | Non-Juice Beverages | Coffee | IMPORT | Brazil | Positive | 1 | 2.7 |
| 903439 | 2015 | 5/4/2015 | BARLEY, WHOLE GRAIN | Grains and Grain Products | Barley | DOMESTIC | United States | Non-Detect | 4.99 | 0 |
| 909905 | 2015 | 5/4/2015 | WHEAT, WHOLE GRAIN | Grains and Grain Products | Wheat | IMPORT | Canada | Non-Detect | 5 | 0 |
| 910315 | 2015 | 5/4/2015 | BARLEY, WHOLE GRAIN | Grains and Grain Products | Barley | IMPORT | Canada | Non-Detect | 4.99 | 0 |
| 909929 | 2015 | 5/5/2015 | RAISINS, DRIED OR PASTE | Fruit | Raisins | IMPORT | Chile | Non-Detect | 5 | 0 |
| 910425 | 2015 | 5/6/2015 | COCOA BEAN N.E.C. | Candy and Chocolate Products | Other Chocolate Products | IMPORT | Ecuador | Non-Detect | 2.3 | 0 |
| 862541 | 2015 | 5/7/2015 | RICE CEREAL (BABY | Baby Food Products | Baby Cereals | DOMESTIC | United States | Non-Detect | 4.99 | 0 |
| 871333 | 2015 | 5/8/2015 | MALT, BARLEY | Grains and Grain Products | Barley | IMPORT | Canada | Positive | 2.3 | 6.9 |
| 910959 | 2015 | 5/11/2015 | BEAN, DRIED OR PASTE, N.E.C. (VEGETABLE) | Beans and Legumes | Other Beans and Legumes | IMPORT | Thailand | Non-Detect | 1 | 0 |
| 910960 | 2015 | 5/11/2015 | BEAN, DRIED OR PASTE, N.E.C. (VEGETABLE) | Beans and Legumes | Other Beans and Legumes | IMPORT | Thailand | Non-Detect | 1 | 0 |
| 910953 | 2015 | 5/11/2015 | WHEAT, MILLED (CRUSHED, COARSE GROUND OR CRACKED) | Grains and Grain Products | Wheat | IMPORT | Canada | Trace | 1 | |
| 911243 | 2015 | 5/12/2015 | KIDNEY BEAN, DRIED OR PASTE | Beans and Legumes | Other Beans and Legumes | IMPORT | India | Positive | 1 | 3.2 |
| 882616 | 2015 | 5/13/2015 | WHEAT, WHOLE GRAIN | Grains and Grain Products | Wheat | DOMESTIC | United States | Non-Detect | 5 | 0 |
| 882617 | 2015 | 5/13/2015 | WHEAT, WHOLE GRAIN | Grains and Grain Products | Wheat | DOMESTIC | United States | Non-Detect | 5 | 0 |
| 903491 | 2015 | 5/14/2015 | RYE FLOUR | Grains and Grain Products | Rye | DOMESTIC | United States | Trace | 5 | |
| 911904 | 2015 | 5/18/2015 | OAT FLAKES, ROLLED OATS, PUFFS, KRISPIES, LOOPS READY TO EAT | Processed Food Products | Breakfast Foods | IMPORT | Canada | Non-Detect | 1 | 0 |
| 912318 | 2015 | 5/19/2015 | OAT FLOUR | Grains and Grain Products | Oats | IMPORT | Canada | Non-Detect | 1 | 0 |
| 878207 | 2015 | 5/27/2015 | MACARONI, WHOLE WHEAT | Processed Food Products | Pasta | DOMESTIC | Unknown | Non-Detect | 2.3 | 0 |
| 913284 | 2015 | 5/27/2015 | KIDNEY BEAN | Beans and Legumes | Other Beans and Legumes | IMPORT | Nicaragua | Non-Detect | 2.3 | 0 |
| 913368 | 2015 | 5/27/2015 | COFFEE, BEANS | Non-Juice Beverages | Coffee | IMPORT | Canada | Non-Detect | 2.3 | 0 |
| 913617 | 2015 | 5/28/2015 | COFFEE, BEANS | Non-Juice Beverages | Coffee | IMPORT | Canada | Non-Detect | 2.3 | 0 |
| 913628 | 2015 | 5/28/2015 | COFFEE, BEANS | Non-Juice Beverages | Coffee | IMPORT | Ethiopia | Non-Detect | 2.3 | 0 |
| 913672 | 2015 | 5/28/2015 | COFFEE, BEANS | Non-Juice Beverages | Coffee | IMPORT | Guatemala | Non-Detect | 1 | 0 |
| 875163 | 2015 | 5/29/2015 | CORN, PLAIN MEAL | Grains and Grain Products | Corn | IMPORT | United States | Non-Detect | 2.3 | 0 |
| 899533 | 2015 | 5/29/2015 | BLACK BEAN, DRIED OR PASTE | Beans and Legumes | Black Beans | DOMESTIC | United States | Non-Detect | 4.99 | 0 |
| 913985 | 2015 | 6/2/2015 | KIDNEY BEAN | Beans and Legumes | Other Beans and Legumes | IMPORT | China | Non-Detect | 1 | 0 |
| 913955 | 2015 | 6/3/2015 | WHEAT FLOUR, WHOLE | Grains and Grain Products | Wheat | DOMESTIC | United States | Non-Detect | 5 | 0 |
| 914502 | 2015 | 6/4/2015 | KIDNEY BEAN | Beans and Legumes | Other Beans and Legumes | IMPORT | China | Non-Detect | 2.3 | 0 |
| 877639 | 2015 | 6/4/2015 | WHEAT FLOUR, ENRICHED (ALL PURPOSE) | Grains and Grain Products | Wheat | DOMESTIC | United States | Non-Detect | 5 | 0 |
| 880428 | 2015 | 6/5/2015 | OATMEAL, REGULAR, FRUIT OR SPICE ADDED, QUICK OR INSTANT COOKING | Processed Food Products | Breakfast Foods | DOMESTIC | United States | Non-Detect | 4.99 | 0 |
| 914792 | 2015 | 6/8/2015 | ADZUKI BEANS, DRIED OR PASTE | Beans and Legumes | Other Beans and Legumes | IMPORT | China | Non-Detect | 1 | 0 |
| 915336 | 2015 | 6/9/2015 | COFFEE, BEANS | Non-Juice Beverages | Coffee | IMPORT | El Salvador | Non-Detect | 2.3 | 0 |
| 915348 | 2015 | 6/9/2015 | COFFEE, BEANS | Non-Juice Beverages | Coffee | IMPORT | Costa Rica | Non-Detect | 2.3 | 0 |
| 915216 | 2015 | 6/9/2015 | RAISINS, DRIED OR PASTE | Fruit | Raisins | IMPORT | Canada | Non-Detect | 1 | 0 |
| 915235 | 2015 | 6/10/2015 | RAISINS, DRIED OR PASTE | Fruit | Raisins | IMPORT | China | Non-Detect | 1 | 0 |
| 913956 | 2015 | 6/16/2015 | FLOURS AND MEALS N.E.C. | Grains and Grain Products | Other Grains | DOMESTIC | United States | Non-Detect | 5 | 0 |
| 889390 | 2015 | 6/19/2015 | CORN, PLAIN MEAL | Grains and Grain Products | Corn | DOMESTIC | United States | Non-Detect | 2.3 | 0 |
| 916681 | 2015 | 6/23/2015 | WHEAT, WHOLE GRAIN | Grains and Grain Products | Wheat | IMPORT | Lebanon | Trace | 1 | |
| 916594 | 2015 | 6/24/2015 | BARLEY, WHOLE GRAIN | Grains and Grain Products | Barley | DOMESTIC | United States | Non-Detect | 5 | 0 |
| 916403 | 2015 | 6/25/2015 | CORN, HOMINY GRITS | Grains and Grain Products | Corn | DOMESTIC | United States | Non-Detect | 2.3 | 0 |
| 916404 | 2015 | 6/25/2015 | CORN, HOMINY GRITS | Grains and Grain Products | Corn | DOMESTIC | United States | Non-Detect | 2.3 | 0 |
| 868979 | 2015 | 6/26/2015 | OAT FLOUR | Grains and Grain Products | Oats | DOMESTIC | United States | Non-Detect | 5 | 0 |
| 917424 | 2015 | 6/29/2015 | PASTRY SHELLS WITHOUT CUSTARD OR CREAM FILLING | Processed Food Products | Other Bakery Products | IMPORT | Canada | Non-Detect | 2.3 | 0 |
| 917464 | 2015 | 6/29/2015 | PASTRY SHELLS WITHOUT CUSTARD OR CREAM FILLING | Processed Food Products | Other Bakery Products | IMPORT | Canada | Non-Detect | 2.3 | 0 |
| 917780 | 2015 | 6/29/2015 | RAISINS (DRIED GRAPES) (BERRY) | Fruit | Raisins | IMPORT | Afghanistan | Non-Detect | 2.3 | 0 |
| 917797 | 2015 | 6/29/2015 | RAISINS (DRIED GRAPES) (BERRY) | Fruit | Raisins | IMPORT | Afghanistan | Non-Detect | 2.3 | 0 |
| 878015 | 2015 | 6/29/2015 | BUCKWHEAT, WHOLE GRAIN | Grains and Grain Products | Buckwheat | DOMESTIC | United States | Non-Detect | 4.99 | 0 |
| 916406 | 2015 | 7/8/2015 | CORN, HOMINY GRITS | Grains and Grain Products | Corn | DOMESTIC | United States | Non-Detect | 2.3 | 0 |
| 918530 | 2015 | 7/9/2015 | NAVY (WHITE) BEANS, (BAKED BEANS, PORK AND BEANS) | Beans and Legumes | Other Beans and Legumes | IMPORT | Canada | Non-Detect | 2.3 | 0 |
| 918533 | 2015 | 7/9/2015 | BEANS, N.E.C. (VEGETABLE) | Beans and Legumes | Other Beans and Legumes | IMPORT | Canada | Non-Detect | 2.3 | 0 |
| 918828 | 2015 | 7/10/2015 | GRAIN, WHOLE, N.E.C. | Grains and Grain Products | Other Grains | IMPORT | India | Non-Detect | 1 | 0 |
| 917440 | 2015 | 7/10/2015 | RAISINS, DRIED OR PASTE | Fruit | Raisins | DOMESTIC | United States | Trace | 5 | |
| 868423 | 2015 | 7/14/2015 | RAISINS, DRIED OR PASTE | Fruit | Raisins | DOMESTIC | United States | Non-Detect | 0.233 | 0 |
| 919350 | 2015 | 7/16/2015 | CORN, BOLTED MEAL OR FLOUR | Grains and Grain Products | Corn | IMPORT | India | Positive | 1 | 2.12 |
| 896909 | 2015 | 7/21/2015 | CORN, BOLTED MEAL OR FLOUR | Grains and Grain Products | Corn | DOMESTIC | United States | Non-Detect | 1 | 0 |
| 896910 | 2015 | 7/21/2015 | MIXED CEREAL, QUICK OR INSTANT COOKING | Processed Food Products | Breakfast Foods | DOMESTIC | United States | Non-Detect | 1 | 0 |
| 920410 | 2015 | 7/25/2015 | BREAKFAST FOODS READY TO EAT, N.E.C. | Processed Food Products | Breakfast Foods | IMPORT | Canada | Non-Detect | 1 | 0 |
| 920579 | 2015 | 7/27/2015 | WHEAT CEREAL (BABY) | Baby Food Products | Baby Cereals | IMPORT | Ecuador | Non-Detect | 2.3 | 0 |
| 881626 | 2015 | 7/29/2015 | WHEAT FLOUR, BROMATED | Grains and Grain Products | Wheat | DOMESTIC | United States | Non-Detect | 5 | 0 |
| 921332 | 2015 | 8/2/2015 | WHEAT GERM | Grains and Grain Products | Wheat | IMPORT | Canada | Non-Detect | 1 | 0 |
| 921439 | 2015 | 8/3/2015 | MALT, BARLEY | Grains and Grain Products | Barley | IMPORT | Belgium | Non-Detect | 2.3 | 0 |
| 909327 | 2015 | 8/5/2015 | CORN, BOLTED MEAL OR FLOUR | Grains and Grain Products | Corn | DOMESTIC | United States | Non-Detect | 5 | 0 |
| 916515 | 2015 | 8/5/2015 | WHEAT, WHOLE GRAIN | Grains and Grain Products | Wheat | DOMESTIC | United States | Non-Detect | 4.99 | 0 |

| | | | | | | | | | | |
|---|---|---|---|---|---|---|---|---|---|---|
| 921868 | 2015 | 8/5/2015 | GINGER, GROUND, CRACKED (SPICE) | Spices | Ginger | IMPORT | India | Positive | 5 | 6.07 |
| 922288 | 2015 | 8/10/2015 | KIDNEY BEAN | Beans and Legumes | Other Beans and Legumes | IMPORT | Nicaragua | Non-Detect | 2.3 | 0 |
| 905259 | 2015 | 8/13/2015 | WHEAT CEREAL (BABY) | Baby Food Products | Baby Cereals | DOMESTIC | United States | Non-Detect | 1 | 0 |
| 911411 | 2015 | 8/13/2015 | OATS, WHOLE GRAIN | Grains and Grain Products | Oats | DOMESTIC | United States | Non-Detect | 1 | 0 |
| 922916 | 2015 | 8/14/2015 | COFFEE, BEANS | Non-Juice Beverages | Coffee | IMPORT | Peru | Non-Detect | 2.3 | 0 |
| 922914 | 2015 | 8/14/2015 | FLOR DE MAYO BEAN | Beans and Legumes | Other Beans and Legumes | IMPORT | Mexico | Non-Detect | 5 | 0 |
| 923188 | 2015 | 8/18/2015 | BLACK EYE BEANS | Beans and Legumes | Blackeye Peas | IMPORT | Mexico | Non-Detect | 5 | 0 |
| 923192 | 2015 | 8/18/2015 | PINTO BEAN | Beans and Legumes | Pinto Beans | IMPORT | Mexico | Non-Detect | 5 | 0 |
| 923194 | 2015 | 8/18/2015 | SOYBEANS (SEE INDUSTRY 37 FOR BEAN CURD), DRIED OR PASTE | Beans and Legumes | Soybeans | IMPORT | Canada | Non-Detect | 1 | 0 |
| 923197 | 2015 | 8/18/2015 | CORN, BOLTED MEAL OR FLOUR | Grains and Grain Products | Corn | IMPORT | Pakistan | Non-Detect | 1 | 0 |
| 923496 | 2015 | 8/19/2015 | COFFEE, BEANS | Non-Juice Beverages | Coffee | IMPORT | Guatemala | Non-Detect | 2.3 | 0 |
| 923512 | 2015 | 8/19/2015 | COFFEE, BEANS | Non-Juice Beverages | Coffee | IMPORT | Indonesia | Non-Detect | 2.3 | 0 |
| 923732 | 2015 | 8/19/2015 | COFFEE, BEANS | Non-Juice Beverages | Coffee | IMPORT | Italy | Non-Detect | 2.3 | 0 |
| 905262 | 2015 | 8/24/2015 | CORN FLAKES, PUFFS, KRISPIES, LOOPS READY TO EAT | Processed Food Products | Other Corn Products | DOMESTIC | United States | Non-Detect | 1 | 0 |
| 923945 | 2015 | 8/25/2015 | COFFEE, BEANS | Non-Juice Beverages | Coffee | IMPORT | Nicaragua | Non-Detect | 1 | 0 |
| 883616 | 2015 | 8/27/2015 | NAVY (WHITE) BEANS (BAKED BEANS, PORK AND BEANS), DRIED OR PASTE | Beans and Legumes | Other Beans and Legumes | DOMESTIC | United States | Non-Detect | 5 | 0 |
| 924663 | 2015 | 8/28/2015 | COFFEE, BEANS | Non-Juice Beverages | Coffee | IMPORT | Honduras | Non-Detect | 2.3 | 0 |
| 871931 | 2015 | 9/2/2015 | WHEAT FLAKES, PUFFS, KRISPIES, LOOPS, SHREDDED, WHEAT GERM READY TO EAT | Processed Food Products | Breakfast Foods | IMPORT | Canada | Non-Detect | 2.3 | 0 |
| 871932 | 2015 | 9/2/2015 | WHEAT FLAKES, PUFFS, KRISPIES, LOOPS, SHREDDED, WHEAT GERM READY TO EAT | Processed Food Products | Breakfast Foods | DOMESTIC | United States | Non-Detect | 4.99 | 0 |
| 924665 | 2015 | 9/2/2015 | RAISINS, DRIED OR PASTE | Fruit | Raisins | DOMESTIC | United States | Non-Detect | 5.13 | 0 |
| 883619 | 2015 | 9/3/2015 | MIXED CEREAL FLAKES, SHREDDED AND OTHER FORMS READY TO EAT | Processed Food Products | Breakfast Foods | DOMESTIC | United States | Non-Detect | 5 | 0 |
| 844435 | 2015 | 9/11/2015 | CREAM WHEAT, QUICK OR INSTANT COOKING | Processed Food Products | Breakfast Foods | DOMESTIC | United States | Non-Detect | 0.233 | 0 |
| 926146 | 2015 | 9/14/2015 | RAISINS, DRIED OR PASTE | Fruit | Raisins | IMPORT | China | Non-Detect | 2.3 | 0 |
| 926335 | 2015 | 9/16/2015 | BEAN, DRIED OR PASTE, N.E.C. (VEGETABLE) | Beans and Legumes | Other Beans and Legumes | IMPORT | Canada | Trace | 1 | |
| 926478 | 2015 | 9/16/2015 | RAISINS (DRIED GRAPES) (BERRY) | Fruit | Raisins | IMPORT | United States | Trace | 1 | |
| 904834 | 2015 | 9/21/2015 | RYE FLOUR | Grains and Grain Products | Rye | DOMESTIC | United States | Non-Detect | 5 | 0 |
| 451299 | 2015 | 9/22/2015 | NAVY (WHITE) BEANS (BAKED BEANS, PORK AND BEANS), DRIED OR PASTE | Beans and Legumes | Other Beans and Legumes | DOMESTIC | United States | Non-Detect | 5 | 0 |
| 926526 | 2015 | 9/22/2015 | RAISINS, DRIED OR PASTE | Fruit | Raisins | IMPORT | Mexico | Non-Detect | 4.99 | 0 |
| 927041 | 2015 | 9/23/2015 | BUCKWHEAT FLOUR | Grains and Grain Products | Buckwheat | DOMESTIC | United States | Non-Detect | 5 | 0 |
| 895723 | 2015 | 9/28/2015 | KIDNEY BEAN, DRIED OR PASTE | Beans and Legumes | Other Beans and Legumes | DOMESTIC | United States | Non-Detect | 5 | 0 |
| 895724 | 2015 | 9/28/2015 | LIMA BEAN, DRIED OR PASTE | Beans and Legumes | Other Beans and Legumes | DOMESTIC | United States | Non-Detect | 5 | 0 |
| 927956 | 2015 | 9/30/2015 | WHEAT FLOUR, WHOLE | Grains and Grain Products | Wheat | IMPORT | India | Non-Detect | 2.3 | 0 |

## FY2016 Data

| Sample Number | Fiscal Year | Collection Date | Product Name | Food Category | Food Subcategory | Origin Type | Country of Origin | Non-Detect, Trace, or Positive (>LOQ) | LOQ (ppb) | Amount found (ppb) |
|---|---|---|---|---|---|---|---|---|---|---|
| 928250 | 2016 | 10/1/2015 | SWEET GOODS WITHOUT CUSTARD OR CREAM FILLING, N.E.C. | Processed Food Products | Other Bakery Products | IMPORT | El Salvador | Non-Detect | 1 | 0 |
| 928596 | 2016 | 10/5/2015 | COFFEE, BEANS | Non-Juice Beverages | Coffee | IMPORT | Honduras | Non-Detect | 2.3 | 0 |
| 928698 | 2016 | 10/5/2015 | RAISINS (DRIED GRAPES) (BERRY) | Fruit | Raisins | IMPORT | Uzbekistan | Non-Detect | 2.3 | 0 |
| 928580 | 2016 | 10/6/2015 | PIGEON PEAS, DRIED OR PASTE | Beans and Legumes | Peas | IMPORT | Peru | Non-Detect | 4.99 | 0 |
| 928605 | 2016 | 10/6/2015 | COFFEE, BEANS | Non-Juice Beverages | Coffee | IMPORT | Haiti | Non-Detect | 5 | 0 |
| 879337 | 2016 | 10/8/2015 | RYE FLOUR | Grains and Grain Products | Rye | DOMESTIC | United States | Non-Detect | 4.99 | 0 |
| 879341 | 2016 | 10/8/2015 | GARBANZO BEAN | Beans and Legumes | Garbanzo Beans | DOMESTIC | United States | Non-Detect | 4.99 | 0 |
| 928876 | 2016 | 10/8/2015 | OATS, WHOLE GRAIN | Grains and Grain Products | Oats | DOMESTIC | United States | Non-Detect | 5 | 0 |
| 879336 | 2016 | 10/8/2015 | BUCKWHEAT FLOUR | Grains and Grain Products | Buckwheat | DOMESTIC | United States | Positive | 4.99 | 6.65 |
| 929210 | 2016 | 10/9/2015 | KIDNEY BEAN, DRIED OR PASTE | Beans and Legumes | Other Beans and Legumes | IMPORT | China | Non-Detect | 5 | 0 |
| 929653 | 2016 | 10/15/2015 | WHEAT, WHOLE GRAIN | Grains and Grain Products | Wheat | IMPORT | Canada | Non-Detect | 1 | 0 |
| 929833 | 2016 | 10/16/2015 | BARLEY, WHOLE GRAIN | Grains and Grain Products | Barley | IMPORT | Ethiopia | Non-Detect | 2.3 | 0 |
| 930073 | 2016 | 10/20/2015 | WHEAT FLOUR, PLAIN | Grains and Grain Products | Wheat | IMPORT | Guatemala | Non-Detect | 0.233 | 0 |
| 931336 | 2016 | 10/27/2015 | COFFEE, BEANS | Non-Juice Beverages | Coffee | IMPORT | Colombia | Non-Detect | 1 | 0 |
| 931676 | 2016 | 10/28/2015 | OAT FLOUR | Grains and Grain Products | Oats | IMPORT | Sweden | Non-Detect | 1 | 0 |
| 931802 | 2016 | 10/29/2015 | CORN, PLAIN MEAL | Grains and Grain Products | Corn | IMPORT | Barbados | Non-Detect | 1 | 0 |
| 931959 | 2016 | 10/30/2015 | COFFEE, INSTANT | Non-Juice Beverages | Coffee | IMPORT | Italy | Non-Detect | 2.3 | 0 |
| 931961 | 2016 | 10/30/2015 | COFFEE, INSTANT | Non-Juice Beverages | Coffee | IMPORT | Italy | Non-Detect | 2.7 | 0 |
| 899651 | 2016 | 11/2/2015 | WHEAT, WHOLE GRAIN | Grains and Grain Products | Wheat | DOMESTIC | United States | Non-Detect | 2.3 | 0 |
| 826596 | 2016 | 11/3/2015 | RYE FLOUR | Grains and Grain Products | Rye | DOMESTIC | United States | Trace | 1 | |
| 932756 | 2016 | 11/5/2015 | COFFEE, BEANS | Non-Juice Beverages | Coffee | IMPORT | Papua New Guinea | Non-Detect | 2.3 | 0 |
| 860479 | 2016 | 11/10/2015 | WHEAT FLOUR, WHOLE | Grains and Grain Products | Wheat | DOMESTIC | United States | Non-Detect | 1 | 0 |
| 933520 | 2016 | 11/12/2015 | COFFEE, BEANS | Non-Juice Beverages | Coffee | IMPORT | Haiti | Non-Detect | 9.225 | 0 |
| 933545 | 2016 | 11/12/2015 | NAVY (WHITE BEANS (BAKED BEANS, PORK & BEANS, DRIED OR PASTE) | Beans and Legumes | Other Beans and Legumes | IMPORT | Canada | Positive | 1 | 12.64 |
| 933723 | 2016 | 11/13/2015 | MALT, BARLEY | Grains and Grain Products | Barley | IMPORT | Mexico | Non-Detect | 0.233 | 0 |
| 933730 | 2016 | 11/13/2015 | CREAM WHEAT, QUICK OR INSTANT COOKING | Processed Food Products | Breakfast Foods | IMPORT | Mexico | Non-Detect | 0.233 | 0 |
| 934304 | 2016 | 11/18/2015 | PERUANO BEANS | Beans and Legumes | Other Beans and Legumes | IMPORT | Mexico | Non-Detect | 0.233 | 0 |
| 934547 | 2016 | 11/19/2015 | COFFEE, BEANS | Non-Juice Beverages | Coffee | IMPORT | Peru | Non-Detect | 2.3 | 0 |
| 934831 | 2016 | 11/22/2015 | COFFEE, BEANS | Non-Juice Beverages | Coffee | IMPORT | Honduras | Non-Detect | 12.15 | 0 |
| 917914 | 2016 | 11/23/2015 | WHEAT FLOUR, PLAIN | Grains and Grain Products | Wheat | DOMESTIC | United States | Non-Detect | 2.3 | 0 |
| 849117 | 2016 | 11/24/2015 | WHEAT FLOUR, GLUTEN | Grains and Grain Products | Wheat | IMPORT | Puerto Rico | Non-Detect | 2.3 | 0 |
| 935124 | 2016 | 11/24/2015 | COFFEE, BEANS | Non-Juice Beverages | Coffee | IMPORT | Germany | Non-Detect | 2.3 | 0 |
| 935296 | 2016 | 11/25/2015 | KIDNEY BEAN | Beans and Legumes | Other Beans and Legumes | IMPORT | Nicaragua | Non-Detect | 2.3 | 0 |
| 935680 | 2016 | 11/30/2015 | COFFEE, BEANS | Non-Juice Beverages | Coffee | IMPORT | Vietnam | Trace | 1 | |
| 936179 | 2016 | 12/1/2015 | WHEAT FLOUR, ENRICHED (ALL PURPOSE) | Grains and Grain Products | Wheat | IMPORT | China | Non-Detect | 1 | 0 |
| 936323 | 2016 | 12/2/2015 | WHEAT CEREAL (BABY) | Baby Food Products | Baby Cereals | IMPORT | United Kingdom | Non-Detect | 1 | 0 |
| 937608 | 2016 | 12/4/2015 | CHANA DAL | Beans and Legumes | Garbanzo Beans | IMPORT | United Arab Emirates | Non-Detect | 2.3 | 0 |
| 937621 | 2016 | 12/4/2015 | MUNG BEAN | Beans and Legumes | Other Beans and Legumes | IMPORT | United Arab Emirates | Non-Detect | 2.3 | 0 |
| 936995 | 2016 | 12/6/2015 | PEA, DRIED OR PASTE | Beans and Legumes | Peas | IMPORT | Canada | Trace | 1 | |
| 937253 | 2016 | 12/7/2015 | RAISINS, DRIED OR PASTE | Fruit | Raisins | IMPORT | Afghanistan | Non-Detect | 1 | 0 |
| 937224 | 2016 | 12/8/2015 | PINTO BEAN | Beans and Legumes | Pinto Beans | IMPORT | Brazil | Non-Detect | 2.3 | 0 |
| 937321 | 2016 | 12/8/2015 | COFFEE, BEANS | Non-Juice Beverages | Coffee | IMPORT | Peru | Non-Detect | 2.3 | 0 |
| 937611 | 2016 | 12/8/2015 | WHEAT, WHOLE GRAIN | Grains and Grain Products | Wheat | IMPORT | Italy | Non-Detect | 2.3 | 0 |
| 937736 | 2016 | 12/8/2015 | CORN, BOLTED MEAL OR FLOUR | Grains and Grain Products | Corn | IMPORT | Brazil | Trace | 1 | |
| 937516 | 2016 | 12/9/2015 | RAISINS, DRIED OR PASTE | Fruit | Raisins | IMPORT | South Africa | Non-Detect | 2.629 | 0 |
| 937491 | 2016 | 12/9/2015 | WHEAT FLOUR, ENRICHED (ALL PURPOSE) | Grains and Grain Products | Wheat | IMPORT | Korea (the Republic of) | Non-Detect | 1 | 0 |
| 937758 | 2016 | 12/10/2015 | WHEAT FLOUR, ENRICHED (ALL PURPOSE) | Grains and Grain Products | Wheat | IMPORT | Dominican Republic (the) | Non-Detect | 2.3 | 0 |
| 938110 | 2016 | 12/10/2015 | CORN, BOLTED MEAL OR FLOUR | Grains and Grain Products | Corn | IMPORT | India | Non-Detect | 1 | 0 |
| 938312 | 2016 | 12/14/2015 | TORTILLAS | Processed Food Products | Other Corn Products | IMPORT | Canada | Non-Detect | 1 | 0 |
| 938107 | 2016 | 12/14/2015 | CORN, BOLTED MEAL OR FLOUR | Grains and Grain Products | Corn | IMPORT | India | Trace | 1 | |
| 917015 | 2016 | 12/15/2015 | WHEAT FLOUR, ENRICHED (ALL PURPOSE) | Grains and Grain Products | Wheat | DOMESTIC | United States | Non-Detect | 1 | 0 |
| 938552 | 2016 | 12/15/2015 | QUINOA FLOUR | Grains and Grain Products | Other Grains | IMPORT | Canada | Non-Detect | 1 | 0 |
| 938624 | 2016 | 12/16/2015 | COFFEE, BEANS | Non-Juice Beverages | Coffee | IMPORT | Brazil | Non-Detect | 1 | 0 |
| 938694 | 2016 | 12/16/2015 | BARLEY, WHOLE GRAIN | Grains and Grain Products | Barley | IMPORT | Hong Kong SAR | Non-Detect | 1 | 0 |
| 849118 | 2016 | 12/21/2015 | BEANS, CORN, AND PEA, N.E.C. (VEGETABLE) | Beans and Legumes | Other Beans and Legumes | DOMESTIC | Unknown | Non-Detect | 2.3 | 0 |
| 934841 | 2016 | 12/21/2015 | BUCKWHEAT FLOUR | Grains and Grain Products | Buckwheat | DOMESTIC | United States | Non-Detect | 1 | 0 |
| 939173 | 2016 | 12/21/2015 | CORN CHIPS, BAKED | Processed Food Products | Other Corn Products | IMPORT | Canada | Non-Detect | 1 | 0 |
| 916443 | 2016 | 12/21/2015 | MIXED CEREAL FLAKES, SHREDDED AND OTHER FORMS READY TO EAT | Processed Food Products | Breakfast Foods | DOMESTIC | United States | Trace | 1 | |
| 930086 | 2016 | 12/22/2015 | RAISINS, DRIED OR PASTE | Fruit | Raisins | IMPORT | United States | Non-Detect | 2.629 | 0 |
| 939274 | 2016 | 12/22/2015 | BLACKEYE PEAS, DRIED OR PASTE | Beans and Legumes | Blackeye Peas | IMPORT | China | Non-Detect | 1 | 0 |
| 939278 | 2016 | 12/22/2015 | STRINGBEAN, DRIED OR PASTE | Beans and Legumes | Other Beans and Legumes | IMPORT | China | Non-Detect | 1 | 0 |
| 939320 | 2016 | 12/22/2015 | CORN, BOLTED MEAL OR FLOUR | Grains and Grain Products | Corn | IMPORT | Italy | Non-Detect | 1 | 0 |
| 901819 | 2016 | 12/22/2015 | RAISINS (DRIED GRAPES) (BERRY) | Fruit | Raisins | DOMESTIC | United States | Trace | 1 | |
| 911052 | 2016 | 12/22/2015 | ROMAN OCTOBER BEAN | Beans and Legumes | Other Beans and Legumes | DOMESTIC | United States | Trace | 1 | |
| 916442 | 2016 | 12/22/2015 | WHEAT FLOUR, WHOLE | Grains and Grain Products | Wheat | DOMESTIC | United States | Trace | 1 | |
| 939357 | 2016 | 12/22/2015 | RYE FLOUR | Grains and Grain Products | Rye | IMPORT | Russia | Trace | 1 | |
| 939394 | 2016 | 12/23/2015 | COFFEE, BEANS | Non-Juice Beverages | Coffee | IMPORT | Zambia | Non-Detect | 2.3 | 0 |
| 931190 | 2016 | 12/23/2015 | OAT FLOUR | Grains and Grain Products | Oats | DOMESTIC | United States | Non-Detect | 0.233 | 0 |
| 938813 | 2016 | 12/23/2015 | CORN CHIPS, BAKED | Processed Food Products | Other Corn Products | DOMESTIC | United States | Non-Detect | 1 | 0 |
| 939404 | 2016 | 12/23/2015 | WHEAT FLOUR, WHOLE | Grains and Grain Products | Wheat | IMPORT | Korea (the Republic of) | Non-Detect | 1 | 0 |
| 939411 | 2016 | 12/23/2015 | WHEAT FLOUR, WHOLE | Grains and Grain Products | Wheat | IMPORT | Korea (the Republic of) | Non-Detect | 1 | 0 |
| 931191 | 2016 | 12/29/2015 | OAT FLOUR | Grains and Grain Products | Oats | DOMESTIC | United States | Non-Detect | 0.233 | 0 |
| 939702 | 2016 | 12/29/2015 | COFFEE, BEANS | Non-Juice Beverages | Coffee | IMPORT | Colombia | Non-Detect | 1 | 0 |
| 939682 | 2016 | 12/29/2015 | CORN, WHOLE GRAIN | Grains and Grain Products | Corn | IMPORT | Peru | Trace | 1 | |
| 939698 | 2016 | 12/29/2015 | CORN, WHOLE GRAIN | Grains and Grain Products | Corn | IMPORT | Peru | Trace | 1 | |
| 939816 | 2016 | 12/31/2015 | COFFEE, BEANS | Non-Juice Beverages | Coffee | IMPORT | Colombia | Non-Detect | 12.15 | 0 |
| 939901 | 2016 | 1/3/2016 | COFFEE, BEANS | Non-Juice Beverages | Coffee | IMPORT | Kenya | Trace | 1 | |
| 940184 | 2016 | 1/5/2016 | COFFEE, BEANS | Non-Juice Beverages | Coffee | IMPORT | Canada | Non-Detect | 1 | 0 |
| 940044 | 2016 | 1/5/2016 | OATS, WHOLE GRAIN | Grains and Grain Products | Oats | IMPORT | Canada | Trace | 1 | |
| 940236 | 2016 | 1/6/2016 | WHEAT, MILLED (CRUSHED, COARSE GROUND OR CRACKED) | Grains and Grain Products | Wheat | IMPORT | Turkey | Non-Detect | 1 | 0 |
| 940278 | 2016 | 1/6/2016 | BARLEY, WHOLE GRAIN | Grains and Grain Products | Barley | IMPORT | China | Non-Detect | 1 | 0 |
| 940503 | 2016 | 1/7/2016 | OATMEAL, REGULAR, FRUIT OR SPICE ADDED, QUICK OR INSTANT COOKING | Processed Food Products | Breakfast Foods | IMPORT | Guatemala | Non-Detect | 2.7 | 0 |
| 893164 | 2016 | 1/7/2016 | WHEAT, WHOLE GRAIN | Grains and Grain Products | Wheat | DOMESTIC | United States | Non-Detect | 0.233 | 0 |
| 940630 | 2016 | 1/7/2016 | COFFEE, BEANS | Non-Juice Beverages | Coffee | IMPORT | Brazil | Non-Detect | 1 | 0 |
| 940747 | 2016 | 1/8/2016 | COFFEE, BEANS | Non-Juice Beverages | Coffee | IMPORT | Colombia | Non-Detect | 2.7 | 0 |
| 941019 | 2016 | 1/9/2016 | OATS, WHOLE GRAIN | Grains and Grain Products | Oats | IMPORT | Canada | Non-Detect | 2.7 | 0 |
| 941042 | 2016 | 1/11/2016 | NAVY (WHITE) BEANS, (BAKED BEANS, PORK AND BEANS) | Beans and Legumes | Other Beans and Legumes | IMPORT | Turkey | Non-Detect | 1 | 0 |
| 941091 | 2016 | 1/11/2016 | FLOURS AND MEALS N.E.C. | Grains and Grain Products | Other Grains | IMPORT | Canada | Non-Detect | 1 | 0 |
| 941513 | 2016 | 1/12/2016 | BARLEY, WHOLE GRAIN | Grains and Grain Products | Barley | IMPORT | Peru | Non-Detect | 1 | 0 |
| 941503 | 2016 | 1/12/2016 | COFFEE, BEANS | Non-Juice Beverages | Coffee | IMPORT | Brazil | Positive | 1 | 16.5 |
| 941617 | 2016 | 1/13/2016 | COFFEE, BEANS | Non-Juice Beverages | Coffee | IMPORT | United Arab Emirates | Positive | 2.4 | 15.5 |
| 942109 | 2016 | 1/15/2016 | RICE FLAKES, PUFFS, KRISPIES, LOOPS READY TO EAT | Processed Food Products | Breakfast Foods | IMPORT | Mexico | Non-Detect | 0.233 | 0 |

| | | | | | | | | | | |
|---|---|---|---|---|---|---|---|---|---|---|
| 942118 | 2016 | 1/15/2016 | RAISINS (DRIED GRAPES) (BERRY) | Fruit | Raisins | IMPORT | South Africa | Non-Detect | 1 | 0 |
| 942166 | 2016 | 1/15/2016 | RAISINS,  DRIED OR PASTE | Fruit | Raisins | IMPORT | China | Non-Detect | 1 | 0 |
| 936967 | 2016 | 1/21/2016 | WHEAT, WHOLE GRAIN | Grains and Grain Products | Wheat | DOMESTIC | United States | Non-Detect | 0.233 | 0 |
| 942901 | 2016 | 1/21/2016 | COFFEE, BEANS | Non-Juice Beverages | Coffee | IMPORT | Italy | Non-Detect | 9.225 | 0 |
| 926263 | 2016 | 1/26/2016 | BLACKEYED PEA | Beans and Legumes | Blackeye Peas | DOMESTIC | United States | Non-Detect | 2.7 | 0 |
| 926264 | 2016 | 1/26/2016 | LIMA BEAN, DRIED OR PASTE | Beans and Legumes | Other Beans and Legumes | DOMESTIC | United States | Non-Detect | 2.7 | 0 |
| 871352 | 2016 | 1/27/2016 | MALT, BARLEY | Grains and Grain Products | Barley | IMPORT | United States | Non-Detect | 2.7 | 0 |
| 930491 | 2016 | 1/27/2016 | RYE FLOUR | Grains and Grain Products | Rye | IMPORT | Canada | Non-Detect | 1 | 0 |
| 943911 | 2016 | 1/28/2016 | CARAMEL CANDY PIECES, SOFT, WITH FRUIT  (WITHOUT CHOCOLATE) | Candy and Chocolate Products | Candy with Fruit | IMPORT | Ukraine | Non-Detect | 1 | 0 |
| 943986 | 2016 | 1/28/2016 | WHEAT FLOUR, PLAIN | Grains and Grain Products | Wheat | IMPORT | India | Non-Detect | 1 | 0 |
| 934843 | 2016 | 1/28/2016 | OAT CEREAL (BABY) | Baby Food Products | Baby Cereals | DOMESTIC | United States | Trace | 1 | |
| 944269 | 2016 | 2/1/2016 | BEAN, DRIED OR PASTE, N.E.C. (VEGETABLE) | Beans and Legumes | Other Beans and Legumes | IMPORT | China | Non-Detect | 2.7 | 0 |
| 902512 | 2016 | 2/1/2016 | RICE CEREAL (BABY) | Baby Food Products | Baby Cereals | DOMESTIC | United States | Non-Detect | 0.233 | 0 |
| 944205 | 2016 | 2/1/2016 | MALT, BARLEY | Grains and Grain Products | Barley | IMPORT | Canada | Non-Detect | 1 | 0 |
| 944331 | 2016 | 2/1/2016 | BARLEY, WHOLE GRAIN | Grains and Grain Products | Barley | IMPORT | China | Non-Detect | 1 | 0 |
| 944342 | 2016 | 2/1/2016 | COFFEE, BEANS | Non-Juice Beverages | Coffee | IMPORT | Germany | Non-Detect | 1 | 0 |
| 944352 | 2016 | 2/1/2016 | CORN, BOLTED MEAL OR FLOUR | Grains and Grain Products | Corn | IMPORT | Pakistan | Non-Detect | 1 | 0 |
| 944530 | 2016 | 2/1/2016 | RAISINS,  DRIED OR PASTE | Fruit | Raisins | IMPORT | Argentina | Non-Detect | 1 | 0 |
| 944512 | 2016 | 2/2/2016 | WHEAT FLOUR, N.E.C. | Grains and Grain Products | Wheat | IMPORT | Korea (the Republic of) | Non-Detect | 1 | 0 |
| 944900 | 2016 | 2/3/2016 | BARLEY, WHOLE GRAIN | Grains and Grain Products | Barley | IMPORT | China | Non-Detect | 1 | 0 |
| 945034 | 2016 | 2/4/2016 | CORN, BOLTED MEAL OR FLOUR | Grains and Grain Products | Corn | IMPORT | China | Non-Detect | 1 | 0 |
| 945368 | 2016 | 2/5/2016 | BARLEY, WHOLE GRAIN | Grains and Grain Products | Barley | IMPORT | Korea (the Republic of) | Non-Detect | 0.233 | 0 |
| 919232 | 2016 | 2/5/2016 | RAISINS,  DRIED OR PASTE | Fruit | Raisins | DOMESTIC | United States | Trace | 5 | |
| 945420 | 2016 | 2/8/2016 | ALIMENTARY PASTE | Processed Food Products | Pasta | IMPORT | Italy | Non-Detect | 1 | 0 |
| 945421 | 2016 | 2/8/2016 | ALIMENTARY PASTE | Processed Food Products | Pasta | IMPORT | Italy | Non-Detect | 1 | 0 |
| 945297 | 2016 | 2/8/2016 | RAISINS,  DRIED OR PASTE | Fruit | Raisins | IMPORT | Chile | Trace | 5 | |
| 945530 | 2016 | 2/9/2016 | MALT, BARLEY | Grains and Grain Products | Barley | IMPORT | Germany | Non-Detect | 0.233 | 0 |
| 945795 | 2016 | 2/10/2016 | OAT FLOUR | Grains and Grain Products | Oats | IMPORT | Guatemala | Non-Detect | 2.7 | 0 |
| 945849 | 2016 | 2/10/2016 | CORN, BOLTED MEAL OR FLOUR | Grains and Grain Products | Corn | IMPORT | Pakistan | Non-Detect | 1 | 0 |
| 946083 | 2016 | 2/11/2016 | WHEAT FLOUR, ENRICHED (ALL PURPOSE) | Grains and Grain Products | Wheat | IMPORT | Australia | Non-Detect | 1 | 0 |
| 946228 | 2016 | 2/12/2016 | COFFEE, BEANS | Non-Juice Beverages | Coffee | IMPORT | Italy | Non-Detect | 2.7 | 0 |
| 931918 | 2016 | 2/16/2016 | PINTO BEAN, DRIED OR PASTE | Beans and Legumes | Pinto Beans | DOMESTIC | United States | Non-Detect | 0.233 | 0 |
| 946530 | 2016 | 2/16/2016 | MUNG BEAN | Beans and Legumes | Other Beans and Legumes | IMPORT | China | Non-Detect | 1 | 0 |
| 946855 | 2016 | 2/17/2016 | BEAN, DRIED OR PASTE, N.E.C. (VEGETABLE) | Beans and Legumes | Other Beans and Legumes | IMPORT | Turkey | Non-Detect | 1 | 0 |
| 946923 | 2016 | 2/18/2016 | MALT, BARLEY | Grains and Grain Products | Barley | IMPORT | Netherlands | Non-Detect | 2.7 | 0 |
| 946877 | 2016 | 2/18/2016 | CORN, PLAIN MEAL | Grains and Grain Products | Corn | IMPORT | Italy | Non-Detect | 1 | 0 |
| 946847 | 2016 | 2/18/2016 | WHOLE WHEAT BREAD, ROLLS, BUNS, ETC DOUGH | Processed Food Products | Bread | IMPORT | Germany | Trace | 1 | |
| 947735 | 2016 | 2/24/2016 | COFFEE, BEANS | Non-Juice Beverages | Coffee | IMPORT | Peru | Non-Detect | 2.7 | 0 |
| 947750 | 2016 | 2/24/2016 | COFFEE, BEANS | Non-Juice Beverages | Coffee | IMPORT | Indonesia | Non-Detect | 9.225 | 0 |
| 924689 | 2016 | 2/29/2016 | RAISINS,  DRIED OR PASTE | Fruit | Raisins | DOMESTIC | United States | Non-Detect | 2.6 | 0 |
| 948646 | 2016 | 3/2/2016 | RICE CEREAL (BABY) | Baby Food Products | Baby Cereals | DOMESTIC | United States | Non-Detect | 2.7 | 0 |
| 945449 | 2016 | 3/2/2016 | KIDNEY BEAN, DRIED OR PASTE | Beans and Legumes | Other Beans and Legumes | DOMESTIC | Unknown | Non-Detect | 0.233 | 0 |
| 932691 | 2016 | 3/3/2016 | RICE FLAKES, PUFFS, KRISPIES, LOOPS READY TO EAT | Processed Food Products | Breakfast Foods | DOMESTIC | United States | Non-Detect | 2.3 | 0 |
| 911413 | 2016 | 3/4/2016 | RAISINS,  DRIED OR PASTE | Fruit | Raisins | DOMESTIC | United States | Non-Detect | 1 | 0 |
| 949350 | 2016 | 3/7/2016 | WHEAT FLOUR, PLAIN | Grains and Grain Products | Wheat | IMPORT | Germany | Non-Detect | 2.7 | 0 |
| 887417 | 2016 | 3/9/2016 | OATS, WHOLE GRAIN | Grains and Grain Products | Oats | DOMESTIC | United States | Non-Detect | 2.7 | 0 |
| 950590 | 2016 | 3/10/2016 | RAISINS,  DRIED OR PASTE | Fruit | Raisins | IMPORT | Afghanistan | Non-Detect | 1 | 0 |
| 950593 | 2016 | 3/10/2016 | RAISINS,  DRIED OR PASTE | Fruit | Raisins | IMPORT | Afghanistan | Non-Detect | 1 | 0 |
| 950595 | 2016 | 3/10/2016 | RAISINS,  DRIED OR PASTE | Fruit | Raisins | IMPORT | Afghanistan | Non-Detect | 1 | 0 |
| 950757 | 2016 | 3/11/2016 | KIDNEY BEAN, DRIED OR PASTE | Beans and Legumes | Other Beans and Legumes | IMPORT | China | Non-Detect | 2.7 | 0 |
| 938719 | 2016 | 3/16/2016 | SOY BASE FORMULA PRODUCT, READY TO FEED | Baby Food Products | Baby Formula | DOMESTIC | United States | Non-Detect | 0.233 | 0 |
| 949340 | 2016 | 3/18/2016 | RICE FLOUR, SWEET | Grains and Grain Products | Rice | DOMESTIC | United States | Non-Detect | 0.233 | 0 |
| 952125 | 2016 | 3/21/2016 | BREAD/ROLLS/BUNS, N.E.C. | Processed Food Products | Bread | IMPORT | China | Non-Detect | 1 | 0 |
| 938759 | 2016 | 3/23/2016 | CORN, PLAIN MEAL | Grains and Grain Products | Corn | DOMESTIC | United States | Non-Detect | 2.7 | 0 |
| 941196 | 2016 | 3/23/2016 | OATS, WHOLE GRAIN | Grains and Grain Products | Oats | DOMESTIC | Unknown | Non-Detect | 0.233 | 0 |
| 941197 | 2016 | 3/23/2016 | WHEAT FLOUR, WHOLE | Grains and Grain Products | Wheat | DOMESTIC | Unknown | Non-Detect | 0.233 | 0 |
| 941198 | 2016 | 3/23/2016 | BARLEY, WHOLE GRAIN | Grains and Grain Products | Barley | DOMESTIC | Unknown | Non-Detect | 0.233 | 0 |
| 941200 | 2016 | 3/23/2016 | CORN, ENRICHED MEAL | Grains and Grain Products | Corn | DOMESTIC | Unknown | Non-Detect | 0.233 | 0 |
| 953179 | 2016 | 3/28/2016 | BARLEY, WHOLE GRAIN | Grains and Grain Products | Barley | IMPORT | China | Non-Detect | 0.233 | 0 |
| 953150 | 2016 | 3/29/2016 | BLACK EYE BEANS | Beans and Legumes | Blackeye Peas | DOMESTIC | United States | Non-Detect | 1 | 0 |
| 953820 | 2016 | 3/31/2016 | OATMEAL, REGULAR, FRUIT OR SPICE ADDED, QUICK OR INSTANT COOKING | Processed Food Products | Breakfast Foods | IMPORT | Canada | Non-Detect | 2.7 | 0 |
| 953871 | 2016 | 3/31/2016 | PEAS | Beans and Legumes | Peas | IMPORT | Canada | Non-Detect | 2.7 | 0 |
| 920133 | 2016 | 3/31/2016 | OATS, WHOLE GRAIN | Grains and Grain Products | Oats | DOMESTIC | United States | Non-Detect | 1 | 0 |
| 953900 | 2016 | 4/1/2016 | COFFEE, BEANS | Non-Juice Beverages | Coffee | IMPORT | Peru | Non-Detect | 1 | 0 |
| 954185 | 2016 | 4/4/2016 | RAISINS,  DRIED OR PASTE | Fruit | Raisins | IMPORT | China | Non-Detect | 1 | 0 |
| 954162 | 2016 | 4/4/2016 | CORN, BOLTED MEAL OR FLOUR | Grains and Grain Products | Corn | IMPORT | India | Positive | 1 | 10.22 |
| 954205 | 2016 | 4/5/2016 | WHEAT FLOUR, WHOLE | Grains and Grain Products | Wheat | DOMESTIC | United States | Non-Detect | 0.233 | 0 |
| 954228 | 2016 | 4/5/2016 | OATS, WHOLE GRAIN | Grains and Grain Products | Oats | IMPORT | Canada | Non-Detect | 1 | 0 |
| 954664 | 2016 | 4/5/2016 | FLOURS AND MEALS N.E.C. | Grains and Grain Products | Other Grains | IMPORT | India | Trace | 1 | |
| 923597 | 2016 | 4/6/2016 | WHEAT FLOUR, BROMATED | Grains and Grain Products | Wheat | DOMESTIC | United States | Non-Detect | 2.7 | 0 |
| 954597 | 2016 | 4/7/2016 | WHEAT, WHOLE GRAIN | Grains and Grain Products | Wheat | IMPORT | Canada | Non-Detect | 2.7 | 0 |
| 954769 | 2016 | 4/7/2016 | KIDNEY BEAN | Beans and Legumes | Other Beans and Legumes | IMPORT | Nicaragua | Non-Detect | 0.233 | 0 |
| 954828 | 2016 | 4/7/2016 | BARLEY, WHOLE GRAIN | Grains and Grain Products | Barley | IMPORT | China | Positive | 1 | 2.8 |
| 955099 | 2016 | 4/11/2016 | CORN FLAKES, PUFFS, KRISPIES, LOOPS READY TO EAT | Processed Food Products | Other Corn Products | IMPORT | Mexico | Non-Detect | 2.7 | 0 |
| 955292 | 2016 | 4/11/2016 | LUPIN BEAN, DRIED OR PASTE | Beans and Legumes | Other Beans and Legumes | IMPORT | Chile | Non-Detect | 2.7 | 0 |
| 955921 | 2016 | 4/13/2016 | CORN FLAKES, PUFFS, KRISPIES, LOOPS READY TO EAT | Processed Food Products | Other Corn Products | IMPORT | India | Non-Detect | 1 | 0 |
| 955879 | 2016 | 4/14/2016 | WHEAT FLOUR, ENRICHED SELF RISING | Grains and Grain Products | Wheat | IMPORT | Canada | Non-Detect | 2.7 | 0 |
| 955954 | 2016 | 4/14/2016 | MALT, BARLEY | Grains and Grain Products | Barley | IMPORT | Germany | Non-Detect | 2.7 | 0 |
| 955888 | 2016 | 4/14/2016 | CORN GRITS, BREWERS ENRICHED WHITE OR YELLOW CORN GRITS,CORN MEAL MUSH, QUICK OR INSTANT COOKING | Processed Food Products | Other Corn Products | IMPORT | Italy | Non-Detect | 1 | 0 |
| 956105 | 2016 | 4/15/2016 | RED BEAN, DRIED OR PASTE | Beans and Legumes | Other Beans and Legumes | IMPORT | El Salvador | Non-Detect | 2.7 | 0 |
| 956383 | 2016 | 4/18/2016 | SNACK FOODS NOT ELSEWHERE MENTIONED, N.E.C. | Processed Food Products | Other Snack Foods | IMPORT | Canada | Non-Detect | 1 | 0 |
| 927654 | 2016 | 4/21/2016 | WHEAT FLAKES, PUFFS, KRISPIES, LOOPS, SHREDDED, WHEAT GERM READY TO EAT | Processed Food Products | Breakfast Foods | IMPORT | United States | Non-Detect | 1 | 0 |
| 939964 | 2016 | 4/21/2016 | OATS, WHOLE GRAIN | Grains and Grain Products | Oats | DOMESTIC | United States | Non-Detect | 1 | 0 |
| 957037 | 2016 | 4/21/2016 | CORN FLAKES, PUFFS, KRISPIES, LOOPS READY TO EAT | Processed Food Products | Other Corn Products | IMPORT | Peru | Non-Detect | 1 | 0 |
| 927653 | 2016 | 4/21/2016 | CREAM WHEAT, QUICK OR INSTANT COOKING | Processed Food Products | Breakfast Foods | DOMESTIC | United States | Trace | 1 | |
| 957432 | 2016 | 4/22/2016 | CORN CHIPS, FRIED | Processed Food Products | Other Corn Products | IMPORT | Guatemala | Non-Detect | 2.7 | 0 |
| 957528 | 2016 | 4/22/2016 | COFFEE, BEANS | Non-Juice Beverages | Coffee | IMPORT | Nicaragua | Non-Detect | 2.7 | 0 |
| 905330 | 2016 | 4/26/2016 | WHEAT FLOUR, ENRICHED (ALL PURPOSE) | Grains and Grain Products | Wheat | IMPORT | Spain | Non-Detect | 0.233 | 0 |
| 958420 | 2016 | 4/27/2016 | FLOURS AND MEALS N.E.C. | Grains and Grain Products | Other Grains | IMPORT | Canada | Non-Detect | 2.7 | 0 |
| 911731 | 2016 | 5/2/2016 | SOY BASE FORMULA PRODUCT, READY TO FEED | Baby Food Products | Baby Formula | DOMESTIC | United States | Non-Detect | 0.233 | 0 |
| 958988 | 2016 | 5/3/2016 | BEANS, N.E.C. (VEGETABLE) | Beans and Legumes | Other Beans and Legumes | IMPORT | Spain | Non-Detect | 2.7 | 0 |
| 959280 | 2016 | 5/4/2016 | COFFEE, BEANS | Non-Juice Beverages | Coffee | IMPORT | Guatemala | Non-Detect | 2.7 | 0 |
| 957751 | 2016 | 5/4/2016 | BUCKWHEAT FLOUR | Grains and Grain Products | Buckwheat | DOMESTIC | United States | Non-Detect | 0.233 | 0 |

| | | | | | | | | | | |
|---|---|---|---|---|---|---|---|---|---|---|
| 936273 | 2016 | 5/5/2016 | RAISINS, DRIED OR PASTE | Fruit | Raisins | DOMESTIC | United States | Non-Detect | 1 | 0 |
| 948675 | 2016 | 5/5/2016 | RAISINS, DRIED OR PASTE | Fruit | Raisins | DOMESTIC | United States | Positive | 1 | 5.33 |
| 959894 | 2016 | 5/9/2016 | BARLEY, WHOLE GRAIN | Grains and Grain Products | Barley | IMPORT | Canada | Non-Detect | 2.7 | 0 |
| 959898 | 2016 | 5/9/2016 | BLACKEYED PEA | Beans and Legumes | Blackeye Peas | IMPORT | Madagascar | Non-Detect | 2.7 | 0 |
| 892425 | 2016 | 5/9/2016 | WHEAT FLOUR, ENRICHED (ALL PURPOSE) | Grains and Grain Products | Wheat | DOMESTIC | United States | Non-Detect | 0.233 | 0 |
| 955229 | 2016 | 5/10/2016 | SOY BASE FORMULA PRODUCT, POWDER FORMULA | Baby Food Products | Baby Formula | DOMESTIC | United States | Non-Detect | 0.233 | 0 |
| 960594 | 2016 | 5/11/2016 | LENTILS, BEAN AND PEA SPROUTS | Beans and Legumes | Lentils | IMPORT | Canada | Non-Detect | 2.7 | 0 |
| 960629 | 2016 | 5/11/2016 | MUNG BEAN, BEAN AND PEA SPROUTS | Beans and Legumes | Other Beans and Legumes | IMPORT | Canada | Non-Detect | 2.7 | 0 |
| 960623 | 2016 | 5/11/2016 | PIGEON PEAS, DRIED OR PASTE | Beans and Legumes | Peas | IMPORT | India | Non-Detect | 1 | 0 |
| 961790 | 2016 | 5/19/2016 | COCOA BEAN N.E.C. | Candy and Chocolate Products | Other Chocolate Products | IMPORT | Ecuador | Non-Detect | 2.7 | 0 |
| 962067 | 2016 | 5/20/2016 | BUCKWHEAT, WHOLE GRAIN | Grains and Grain Products | Buckwheat | IMPORT | China | Trace | 1 | |
| 962281 | 2016 | 5/23/2016 | COFFEE, BEANS | Non-Juice Beverages | Coffee | IMPORT | Guatemala | Non-Detect | 2.7 | 0 |
| 871356 | 2016 | 5/24/2016 | MALT, BARLEY | Grains and Grain Products | Barley | IMPORT | United States | Non-Detect | 2.7 | 0 |
| 962491 | 2016 | 5/24/2016 | COFFEE, BEANS | Non-Juice Beverages | Coffee | IMPORT | Costa Rica | Non-Detect | 2.7 | 0 |
| 942241 | 2016 | 5/27/2016 | BUCKWHEAT FLOUR | Grains and Grain Products | Buckwheat | DOMESTIC | United States | Non-Detect | 0.233 | 0 |
| 942244 | 2016 | 5/27/2016 | BUCKWHEAT FLOUR | Grains and Grain Products | Buckwheat | DOMESTIC | United States | Trace | 5 | |
| 963060 | 2016 | 6/1/2016 | OATS, WHOLE GRAIN | Grains and Grain Products | Oats | IMPORT | Mexico | Non-Detect | 0.233 | 0 |
| 961529 | 2016 | 6/2/2016 | CREAM WHEAT, QUICK OR INSTANT COOKING | Processed Food Products | Breakfast Foods | DOMESTIC | United States | Non-Detect | 0.233 | 0 |
| 958521 | 2016 | 6/2/2016 | WHOLE WHEAT BREAD/ROLLS/BUNS | Processed Food Products | Bread | DOMESTIC | Unknown | Non-Detect | 1 | 0 |
| 940251 | 2016 | 6/3/2016 | COFFEE, GROUND | Non-Juice Beverages | Coffee | DOMESTIC | United States | Non-Detect | 2.7 | 0 |
| 944107 | 2016 | 6/7/2016 | KIDNEY BEAN | Beans and Legumes | Other Beans and Legumes | DOMESTIC | United States | Non-Detect | 2.7 | 0 |
| 944108 | 2016 | 6/7/2016 | PINTO BEAN | Beans and Legumes | Pinto Beans | DOMESTIC | United States | Non-Detect | 2.7 | 0 |
| 964135 | 2016 | 6/8/2016 | COFFEE, BEANS | Non-Juice Beverages | Coffee | IMPORT | Nicaragua | Non-Detect | 2.7 | 0 |
| 964860 | 2016 | 6/10/2016 | PEA, DRIED OR PASTE | Beans and Legumes | Peas | IMPORT | Ethiopia | Positive | 2.5 | 19.38 |
| 964884 | 2016 | 6/13/2016 | WHEAT, WHOLE GRAIN | Grains and Grain Products | Wheat | IMPORT | Canada | Non-Detect | 2.7 | 0 |
| 961532 | 2016 | 6/14/2016 | PINTO BEAN, DRIED OR PASTE | Beans and Legumes | Pinto Beans | DOMESTIC | United States | Non-Detect | 0.233 | 0 |
| 961533 | 2016 | 6/14/2016 | OATMEAL, REGULAR, FRUIT OR SPICE ADDED, QUICK OR INSTANT COOKING | Processed Food Products | Breakfast Foods | DOMESTIC | United States | Non-Detect | 0.233 | 0 |
| 965274 | 2016 | 6/15/2016 | WHEAT, MILLED (CRUSHED, COARSE GROUND OR CRACKED) | Grains and Grain Products | Wheat | IMPORT | Lebanon | Non-Detect | 2.7 | 0 |
| 835050 | 2016 | 6/15/2016 | KIDNEY BEAN, DRIED OR PASTE | Beans and Legumes | Other Beans and Legumes | DOMESTIC | United States | Non-Detect | 0.233 | 0 |
| 965101 | 2016 | 6/15/2016 | BUCKWHEAT, WHOLE GRAIN | Grains and Grain Products | Buckwheat | IMPORT | Netherlands | Non-Detect | 1 | 0 |
| 963625 | 2016 | 6/15/2016 | WHEAT FLOUR, WHOLE | Grains and Grain Products | Wheat | DOMESTIC | United States | Trace | 4.99 | |
| 965826 | 2016 | 6/17/2016 | BUCKWHEAT FLOUR | Grains and Grain Products | Buckwheat | IMPORT | Ukraine | Non-Detect | 1 | 0 |
| 961538 | 2016 | 6/21/2016 | WHEAT CEREAL (BABY) | Baby Food Products | Baby Cereals | IMPORT | United States | Non-Detect | 0.233 | 0 |
| 961539 | 2016 | 6/21/2016 | RICE CEREAL (BABY) | Baby Food Products | Baby Cereals | DOMESTIC | United States | Non-Detect | 0.233 | 0 |
| 966215 | 2016 | 6/22/2016 | BLACKEYED PEA | Beans and Legumes | Blackeye Peas | IMPORT | China | Non-Detect | 2.7 | 0 |
| 966226 | 2016 | 6/22/2016 | RYE FLOUR | Grains and Grain Products | Rye | IMPORT | Romania | Non-Detect | 1 | 0 |
| 966230 | 2016 | 6/22/2016 | RYE FLOUR | Grains and Grain Products | Rye | IMPORT | Romania | Non-Detect | 1 | 0 |
| 956851 | 2016 | 6/23/2016 | BUCKWHEAT FLOUR | Grains and Grain Products | Buckwheat | DOMESTIC | United States | Non-Detect | 1 | 0 |
| 966726 | 2016 | 6/24/2016 | COFFEE, BEANS | Non-Juice Beverages | Coffee | IMPORT | Guatemala | Trace | 1 | |
| 944109 | 2016 | 6/27/2016 | MALT, BARLEY | Grains and Grain Products | Barley | DOMESTIC | United States | Non-Detect | 2.7 | 0 |
| 926910 | 2016 | 6/29/2016 | RICE CEREAL (BABY) | Baby Food Products | Baby Cereals | DOMESTIC | United States | Non-Detect | 2.7 | 0 |
| 967532 | 2016 | 6/29/2016 | MIXED CEREAL FLAKES, SHREDDED AND OTHER FORMS READY TO EAT | Processed Food Products | Breakfast Foods | IMPORT | Germany | Non-Detect | 2.7 | 0 |
| 967535 | 2016 | 6/29/2016 | MIXED CEREAL FLAKES, SHREDDED AND OTHER FORMS READY TO EAT | Processed Food Products | Breakfast Foods | IMPORT | Germany | Non-Detect | 2.7 | 0 |
| 967539 | 2016 | 6/29/2016 | MIXED CEREAL FLAKES, SHREDDED AND OTHER FORMS READY TO EAT | Processed Food Products | Breakfast Foods | IMPORT | Germany | Non-Detect | 2.7 | 0 |
| 967542 | 2016 | 6/29/2016 | MIXED CEREAL FLAKES, SHREDDED AND OTHER FORMS READY TO EAT | Processed Food Products | Breakfast Foods | IMPORT | Germany | Non-Detect | 2.7 | 0 |
| 967431 | 2016 | 6/30/2016 | BUCKWHEAT FLOUR | Grains and Grain Products | Buckwheat | IMPORT | Korea (the Republic of) | Non-Detect | 1 | 0 |
| 967745 | 2016 | 7/5/2016 | CEREAL PREPARATIONS NOT ELSEWHERE MENTIONED, N.E.C. | Processed Food Products | Breakfast Foods | IMPORT | Japan | Non-Detect | 2.7 | 0 |
| 943840 | 2016 | 7/5/2016 | GARBANZO BEAN, DRIED OR PASTE | Beans and Legumes | Garbanzo Beans | DOMESTIC | United States | Non-Detect | 0.233 | 0 |
| 891231 | 2016 | 7/7/2016 | WHEAT, WHOLE GRAIN | Grains and Grain Products | Wheat | DOMESTIC | United States | Non-Detect | 2.7 | 0 |
| 968211 | 2016 | 7/7/2016 | OTHER BAKERY PRODUCTS, N.E.C. | Processed Food Products | Other Bakery Products | IMPORT | Pakistan | Non-Detect | 2.7 | 0 |
| 868452 | 2016 | 7/8/2016 | PINTO BEAN, DRIED OR PASTE | Beans and Legumes | Pinto Beans | DOMESTIC | United States | Non-Detect | 2.2 | 0 |
| 958897 | 2016 | 7/13/2016 | MALT, BARLEY | Grains and Grain Products | Barley | DOMESTIC | Unknown | Non-Detect | 2.7 | 0 |
| 968930 | 2016 | 7/14/2016 | MALT, BARLEY | Grains and Grain Products | Barley | IMPORT | France | Non-Detect | 2.7 | 0 |
| 964267 | 2016 | 7/16/2016 | SOY BASE FORMULA PRODUCT, POWDER FORMULA | Baby Food Products | Baby Formula | DOMESTIC | United States | Non-Detect | 0.233 | 0 |
| 964268 | 2016 | 7/16/2016 | SOY BASE FORMULA PRODUCT, POWDER FORMULA | Baby Food Products | Baby Formula | DOMESTIC | United States | Non-Detect | 0.233 | 0 |
| 969723 | 2016 | 7/21/2016 | MIXED CEREAL FLAKES, SHREDDED AND OTHER FORMS READY TO EAT | Processed Food Products | Breakfast Foods | IMPORT | Germany | Non-Detect | 0.233 | 0 |
| 970124 | 2016 | 7/25/2016 | COFFEE, BEANS | Non-Juice Beverages | Coffee | IMPORT | Brazil | Non-Detect | 2.7 | 0 |
| 970105 | 2016 | 7/25/2016 | MALT, BARLEY | Grains and Grain Products | Barley | IMPORT | United Kingdom | Non-Detect | 0.233 | 0 |
| 945130 | 2016 | 8/1/2016 | RYE FLOUR | Grains and Grain Products | Rye | DOMESTIC | United States | Trace | 5 | |
| 971015 | 2016 | 8/2/2016 | COFFEE, BEANS | Non-Juice Beverages | Coffee | IMPORT | Colombia | Non-Detect | 2.7 | 0 |
| 926694 | 2016 | 8/2/2016 | WHEAT, WHOLE GRAIN | Grains and Grain Products | Wheat | DOMESTIC | Unknown | Non-Detect | 1 | 0 |
| 926695 | 2016 | 8/2/2016 | WHEAT, WHOLE GRAIN | Grains and Grain Products | Wheat | DOMESTIC | Unknown | Non-Detect | 1 | 0 |
| 926696 | 2016 | 8/2/2016 | BARLEY, WHOLE GRAIN | Grains and Grain Products | Barley | DOMESTIC | Unknown | Non-Detect | 1 | 0 |
| 948592 | 2016 | 8/2/2016 | BARLEY, WHOLE GRAIN | Grains and Grain Products | Barley | DOMESTIC | Unknown | Non-Detect | 1 | 0 |
| 971309 | 2016 | 8/3/2016 | MALT, BARLEY | Grains and Grain Products | Barley | IMPORT | Germany | Non-Detect | 0.233 | 0 |
| 971530 | 2016 | 8/4/2016 | COFFEE, BEANS | Non-Juice Beverages | Coffee | IMPORT | Vietnam | Non-Detect | 2.7 | 0 |
| 972083 | 2016 | 8/9/2016 | COFFEE, BEANS | Non-Juice Beverages | Coffee | IMPORT | Guatemala | Non-Detect | 2.7 | 0 |
| 892433 | 2016 | 8/9/2016 | WHEAT, WHOLE GRAIN | Grains and Grain Products | Wheat | DOMESTIC | United States | Non-Detect | 0.233 | 0 |
| 955611 | 2016 | 8/10/2016 | BUCKWHEAT FLOUR | Grains and Grain Products | Buckwheat | DOMESTIC | United States | Non-Detect | 0.233 | 0 |
| 892435 | 2016 | 8/12/2016 | WHEAT, WHOLE GRAIN | Grains and Grain Products | Wheat | DOMESTIC | United States | Non-Detect | 0.233 | 0 |
| 972817 | 2016 | 8/15/2016 | COFFEE, BEANS | Non-Juice Beverages | Coffee | IMPORT | Peru | Non-Detect | 2.7 | 0 |
| 972863 | 2016 | 8/15/2016 | COFFEE, BEANS | Non-Juice Beverages | Coffee | IMPORT | China | Non-Detect | 2.7 | 0 |
| 962394 | 2016 | 8/16/2016 | WHEAT, WHOLE GRAIN | Grains and Grain Products | Wheat | DOMESTIC | United States | Non-Detect | 2.7 | 0 |
| 973046 | 2016 | 8/16/2016 | COFFEE, BEANS | Non-Juice Beverages | Coffee | IMPORT | Nicaragua | Non-Detect | 2.7 | 0 |
| 935131 | 2016 | 8/16/2016 | BUCKWHEAT FLOUR | Grains and Grain Products | Buckwheat | DOMESTIC | United States | Non-Detect | 0.233 | 0 |
| 935132 | 2016 | 8/16/2016 | OAT FLOUR | Grains and Grain Products | Oats | DOMESTIC | United States | Non-Detect | 0.233 | 0 |
| 968637 | 2016 | 8/17/2016 | NAVY (WHITE BEANS (BAKED BEANS, PORK & BEANS, DRIED OR PASTE) | Beans and Legumes | Other Beans and Legumes | IMPORT | China | Non-Detect | 0.233 | 0 |
| 948609 | 2016 | 8/19/2016 | MALT, BARLEY | Grains and Grain Products | Barley | DOMESTIC | Unknown | Non-Detect | 1 | 0 |
| 948612 | 2016 | 8/19/2016 | MALT, BARLEY | Grains and Grain Products | Barley | DOMESTIC | Unknown | Non-Detect | 1 | 0 |
| 974414 | 2016 | 8/22/2016 | WHEAT FLOUR, ENRICHED BROMATED | Grains and Grain Products | Wheat | IMPORT | India | Non-Detect | 2.7 | 0 |
| 971983 | 2016 | 8/23/2016 | RAISINS, DRIED OR PASTE | Fruit | Raisins | DOMESTIC | United States | Non-Detect | 2.7 | 0 |
| 974489 | 2016 | 8/24/2016 | WHEAT, WHOLE GRAIN | Grains and Grain Products | Wheat | IMPORT | Canada | Non-Detect | 0.233 | 0 |
| 965186 | 2016 | 8/25/2016 | SOY BASE FORMULA PRODUCT, READY TO FEED | Baby Food Products | Baby Formula | DOMESTIC | United States | Non-Detect | 0.233 | 0 |
| 975781 | 2016 | 9/1/2016 | COFFEE, BEANS | Non-Juice Beverages | Coffee | IMPORT | Ethiopia | Non-Detect | 2.7 | 0 |
| 976032 | 2016 | 9/1/2016 | COFFEE, BEANS | Non-Juice Beverages | Coffee | IMPORT | Lao Peoples Democratic Repblc. | Non-Detect | 2.7 | 0 |
| 975721 | 2016 | 9/1/2016 | WHEAT, MILLED PRODUCT, N.E.C. | Grains and Grain Products | Wheat | IMPORT | India | Non-Detect | 1 | 0 |
| 976058 | 2016 | 9/6/2016 | COFFEE, BEANS | Non-Juice Beverages | Coffee | IMPORT | Honduras | Non-Detect | 2.7 | 0 |
| 965220 | 2016 | 9/6/2016 | LINGUINE | Processed Food Products | Pasta | DOMESTIC | United States | Non-Detect | 1 | 0 |
| 965222 | 2016 | 9/6/2016 | PRETZELS,BAKED | Processed Food Products | Other Snack Foods | DOMESTIC | United States | Non-Detect | 1 | 0 |
| 965223 | 2016 | 9/6/2016 | PRETZELS,BAKED | Processed Food Products | Other Snack Foods | DOMESTIC | United States | Non-Detect | 1 | 0 |
| 937527 | 2016 | 9/8/2016 | OAT CEREAL (BABY) | Baby Food Products | Baby Cereals | DOMESTIC | United States | Non-Detect | 0.233 | 0 |
| 976725 | 2016 | 9/9/2016 | COFFEE, BEANS | Non-Juice Beverages | Coffee | IMPORT | Guatemala | Non-Detect | 2.7 | 0 |
| 976873 | 2016 | 9/12/2016 | COFFEE, BEANS | Non-Juice Beverages | Coffee | IMPORT | Papua New Guinea | Non-Detect | 2.7 | 0 |

| | | | | | | | | | | |
|---|---|---|---|---|---|---|---|---|---|---|
| 977515 | 2016 | 9/14/2016 | COFFEE, INSTANT | Non-Juice Beverages | Coffee | IMPORT | Colombia | Non-Detect | 2.7 | 0 |
| 976823 | 2016 | 9/15/2016 | OAT FLAKES, ROLLED OATS, PUFFS, KRISPIES, LOOPS READY TO EAT | Processed Food Products | Breakfast Foods | DOMESTIC | United States | Non-Detect | 2.7 | 0 |
| 970808 | 2016 | 9/15/2016 | RICE CEREAL (BABY) | Baby Food Products | Baby Cereals | DOMESTIC | United States | Non-Detect | 0.233 | 0 |
| 967184 | 2016 | 9/19/2016 | RYE FLOUR | Grains and Grain Products | Rye | DOMESTIC | United States | Trace | 5 | |
| 975372 | 2016 | 9/20/2016 | WHEAT FLOUR, WHOLE | Grains and Grain Products | Wheat | DOMESTIC | United States | Non-Detect | 1 | 0 |
| 978689 | 2016 | 9/22/2016 | NAVY (WHITE BEANS (BAKED BEANS, PORK & BEANS, DRIED OR PASTE) | Beans and Legumes | Other Beans and Legumes | IMPORT | Nigeria | Non-Detect | 2.7 | 0 |
| 979470 | 2016 | 9/26/2016 | MIXED SPICES AND SEASONING WITH SALT, N.E.C. | Spices | Other Spices | IMPORT | Philippines | Non-Detect | 1 | 0 |
| 871360 | 2016 | 9/28/2016 | MALT, BARLEY | Grains and Grain Products | Barley | IMPORT | United States | Non-Detect | 2.7 | 0 |
| 871361 | 2016 | 9/28/2016 | MALT, BARLEY | Grains and Grain Products | Barley | IMPORT | United States | Non-Detect | 2.7 | 0 |
| 979821 | 2016 | 9/28/2016 | FARINA, WHEAT | Grains and Grain Products | Wheat | IMPORT | India | Non-Detect | 2.4 | 0 |
| 967185 | 2016 | 9/28/2016 | RYE FLOUR | Grains and Grain Products | Rye | DOMESTIC | United States | Non-Detect | 0.233 | 0 |
| 979105 | 2016 | 9/29/2016 | WHEAT BRAN (HUMAN USE) | Grains and Grain Products | Wheat | DOMESTIC | United States | Non-Detect | 2.7 | 0 |

**FY2017 Data**

| Sample Number | Fiscal Year | Collection Date | Product Name | Food Category | Food Subcategory | Origin Type | Country of Origin | Non-Detect, Trace, or Positive (>LOQ) | LOQ (ppb) | Amount found (ppb) |
|---|---|---|---|---|---|---|---|---|---|---|
| 981166 | 2017 | 10/5/2016 | OATMEAL, REGULAR, FRUIT OR SPICE ADDED, QUICK OR INSTANT COOKING | Processed Food Products | Breakfast Foods | IMPORT | Russia | Non-Detect | 1 | 0 |
| 981264 | 2017 | 10/6/2016 | COFFEE, BEANS | Non-Juice Beverages | Coffee | IMPORT | Vietnam | Non-Detect | 2.4 | 0 |
| 981863 | 2017 | 10/12/2016 | BLACK BEAN, DRIED OR PASTE | Beans and Legumes | Black Beans | IMPORT | Costa Rica | Non-Detect | 2.1 | 0 |
| 982327 | 2017 | 10/13/2016 | RAISINS, DRIED OR PASTE | Fruit | Raisins | IMPORT | Chile | Non-Detect | 1 | 0 |
| 982366 | 2017 | 10/14/2016 | BEAN, DRIED OR PASTE, N.E.C. (VEGETABLE) | Beans and Legumes | Other Beans and Legumes | IMPORT | Mexico | Non-Detect | 0.233 | 0 |
| 982553 | 2017 | 10/17/2016 | COFFEE, BEANS | Non-Juice Beverages | Coffee | IMPORT | Brazil | Non-Detect | 1 | 0 |
| 982778 | 2017 | 10/18/2016 | COFFEE, BEANS | Non-Juice Beverages | Coffee | IMPORT | Brazil | Non-Detect | 2.6 | 0 |
| 983182 | 2017 | 10/20/2016 | OATS, WHOLE GRAIN | Grains and Grain Products | Oats | IMPORT | Ireland | Non-Detect | 1 | 0 |
| 983393 | 2017 | 10/20/2016 | OATS, WHOLE GRAIN | Grains and Grain Products | Oats | IMPORT | Ireland | Non-Detect | 1 | 0 |
| 982211 | 2017 | 10/24/2016 | BLACK BEAN, DRIED OR PASTE | Beans and Legumes | Black Beans | DOMESTIC | United States | Trace | 5 | |
| 984628 | 2017 | 10/26/2016 | MUNG BEAN | Beans and Legumes | Other Beans and Legumes | IMPORT | China | Non-Detect | 1 | 0 |
| 984453 | 2017 | 10/27/2016 | COFFEE, BEANS | Non-Juice Beverages | Coffee | IMPORT | Peru | Non-Detect | 2.6 | 0 |
| 984458 | 2017 | 10/27/2016 | COFFEE, BEANS | Non-Juice Beverages | Coffee | IMPORT | Peru | Non-Detect | 1 | 0 |
| 985398 | 2017 | 11/2/2016 | RED BEAN | Beans and Legumes | Other Beans and Legumes | IMPORT | Nicaragua | Non-Detect | 2.3 | 0 |
| 940665 | 2017 | 11/2/2016 | WHEAT FLOUR, BLEACHED | Grains and Grain Products | Wheat | DOMESTIC | United States | Non-Detect | 1 | 0 |
| 985280 | 2017 | 11/2/2016 | OATS, WHOLE GRAIN | Grains and Grain Products | Oats | IMPORT | Canada | Non-Detect | 1 | 0 |
| 985730 | 2017 | 11/3/2016 | COFFEE, BEANS | Non-Juice Beverages | Coffee | IMPORT | Guatemala | Non-Detect | 2.4 | 0 |
| 973994 | 2017 | 11/3/2016 | RICE CEREAL (BABY) | Baby Food Products | Baby Cereals | DOMESTIC | United States | Non-Detect | 0.233 | 0 |
| 963384 | 2017 | 11/3/2016 | RAISINS, DRIED OR PASTE | Fruit | Raisins | DOMESTIC | United States | Positive | 5 | 10.2 |
| 986028 | 2017 | 11/4/2016 | COFFEE, BEANS | Non-Juice Beverages | Coffee | IMPORT | Indonesia | Non-Detect | 12.15 | 0 |
| 910198 | 2017 | 11/7/2016 | OATS, WHOLE GRAIN | Grains and Grain Products | Oats | DOMESTIC | United States | Non-Detect | 2.7 | 0 |
| 979214 | 2017 | 11/8/2016 | RICE CEREAL (BABY) | Baby Food Products | Baby Cereals | DOMESTIC | United States | Non-Detect | 2.7 | 0 |
| 979215 | 2017 | 11/8/2016 | RICE CEREAL (BABY) | Baby Food Products | Baby Cereals | DOMESTIC | United States | Non-Detect | 2.4 | 0 |
| 986188 | 2017 | 11/8/2016 | COFFEE, BEANS | Non-Juice Beverages | Coffee | IMPORT | Peru | Non-Detect | 1 | 0 |
| 943282 | 2017 | 11/9/2016 | RICE CEREAL (BABY) | Baby Food Products | Baby Cereals | DOMESTIC | United States | Non-Detect | 2.7 | 0 |
| 968614 | 2017 | 11/9/2016 | PINTO BEAN, DRIED OR PASTE | Beans and Legumes | Pinto Beans | DOMESTIC | United States | Non-Detect | 0.233 | 0 |
| 987058 | 2017 | 11/14/2016 | SPAGHETTI | Processed Food Products | Pasta | IMPORT | Germany | Non-Detect | 1 | 0 |
| 987176 | 2017 | 11/15/2016 | OATS, WHOLE GRAIN | Grains and Grain Products | Oats | IMPORT | Mexico | Non-Detect | 0.233 | 0 |
| 987228 | 2017 | 11/15/2016 | COFFEE, BEANS | Non-Juice Beverages | Coffee | IMPORT | Colombia | Non-Detect | 1 | 0 |
| 987754 | 2017 | 11/16/2016 | COFFEE, BEANS | Non-Juice Beverages | Coffee | IMPORT | Colombia | Non-Detect | 2.4 | 0 |
| 987335 | 2017 | 11/16/2016 | COFFEE, BEANS | Non-Juice Beverages | Coffee | IMPORT | Guatemala | Non-Detect | 1 | 0 |
| 987871 | 2017 | 11/17/2016 | BULGHUR WHEAT (STEAMED, DRIED AND CRUSHED WHEAT KERNELS FROM MIDDLE EAST) | Grains and Grain Products | Wheat | IMPORT | Turkey | Non-Detect | 2.4 | 0 |
| 987604 | 2017 | 11/17/2016 | COFFEE, BEANS | Non-Juice Beverages | Coffee | IMPORT | Brazil | Non-Detect | 12.15 | 0 |
| 940666 | 2017 | 11/17/2016 | WHEAT FLOUR, BLEACHED | Grains and Grain Products | Wheat | DOMESTIC | United States | Non-Detect | 1 | 0 |
| 978657 | 2017 | 11/21/2016 | WHEAT FLOUR, ENRICHED (ALL PURPOSE) | Grains and Grain Products | Wheat | DOMESTIC | United States | Non-Detect | 2.7 | 0 |
| 978658 | 2017 | 11/21/2016 | WHEAT FLOUR, ENRICHED (ALL PURPOSE) | Grains and Grain Products | Wheat | DOMESTIC | United States | Non-Detect | 2.7 | 0 |
| 988143 | 2017 | 11/21/2016 | RAISINS, DRIED OR PASTE | Fruit | Raisins | IMPORT | Canada | Non-Detect | 1 | 0 |
| 988295 | 2017 | 11/22/2016 | WHEAT FLOUR, ENRICHED (ALL PURPOSE) | Grains and Grain Products | Wheat | IMPORT | Russia | Non-Detect | 1 | 0 |
| 988335 | 2017 | 11/22/2016 | COFFEE, BEANS | Non-Juice Beverages | Coffee | IMPORT | Guatemala | Non-Detect | 1 | 0 |
| 988084 | 2017 | 11/22/2016 | COFFEE, BEANS | Non-Juice Beverages | Coffee | IMPORT | Peru | Trace | 5 | |
| 988362 | 2017 | 11/23/2016 | WHEAT FLOUR, ENRICHED (ALL PURPOSE) | Grains and Grain Products | Wheat | IMPORT | Korea (the Republic of) | Non-Detect | 1 | 0 |
| 988549 | 2017 | 11/23/2016 | TEMPURA KO (JAPANESE LOW GLUTEN WHEAT FLOUR USED TO MAKE TEMPURA BATTER) | Grains and Grain Products | Wheat | IMPORT | Singapore | Non-Detect | 1 | 0 |
| 988314 | 2017 | 11/23/2016 | WHEAT FLOUR, DURUM | Grains and Grain Products | Wheat | IMPORT | Italy | Trace | 5 | |
| 988994 | 2017 | 11/28/2016 | CORN CHIPS, FRIED | Processed Food Products | Other Corn Products | IMPORT | Korea (the Republic of) | Non-Detect | 1 | 0 |
| 989025 | 2017 | 11/28/2016 | CORN CHIPS, FRIED | Processed Food Products | Other Corn Products | IMPORT | Korea (the Republic of) | Non-Detect | 1 | 0 |
| 951991 | 2017 | 11/29/2016 | WHEAT CEREAL (BABY) | Baby Food Products | Baby Cereals | DOMESTIC | United States | Non-Detect | 0.233 | 0 |
| 940668 | 2017 | 11/29/2016 | WHEAT FLOUR, N.E.C. | Grains and Grain Products | Wheat | DOMESTIC | United States | Trace | 1 | |
| 940669 | 2017 | 11/29/2016 | SEMOLINA, WHEAT | Grains and Grain Products | Wheat | DOMESTIC | United States | Trace | 1 | |
| 988864 | 2017 | 11/30/2016 | GLUTEN, WHEAT | Grains and Grain Products | Wheat | IMPORT | Lithuania | Trace | 4.99 | |
| 989042 | 2017 | 11/30/2016 | MALT, BARLEY | Grains and Grain Products | Barley | IMPORT | United Kingdom | Trace | 1 | |
| 989450 | 2017 | 12/2/2016 | CORN, BOLTED MEAL OR FLOUR | Grains and Grain Products | Corn | IMPORT | Italy | Non-Detect | 1 | 0 |
| 968777 | 2017 | 12/5/2016 | CORN, BOLTED MEAL OR FLOUR | Grains and Grain Products | Corn | DOMESTIC | United States | Non-Detect | 0.233 | 0 |
| 989612 | 2017 | 12/5/2016 | CORN, ENRICHED MEAL | Grains and Grain Products | Corn | IMPORT | Italy | Non-Detect | 1 | 0 |
| 989632 | 2017 | 12/5/2016 | COFFEE, INSTANT | Non-Juice Beverages | Coffee | IMPORT | India | Non-Detect | 1 | 0 |
| 990875 | 2017 | 12/5/2016 | CORN, PLAIN MEAL | Grains and Grain Products | Corn | IMPORT | France | Non-Detect | 1 | 0 |
| 989532 | 2017 | 12/7/2016 | RAISINS, DRIED OR PASTE | Fruit | Raisins | DOMESTIC | United States | Non-Detect | 2.1 | 0 |
| 991075 | 2017 | 12/12/2016 | COFFEE, BEANS | Non-Juice Beverages | Coffee | IMPORT | Italy | Non-Detect | 9.225 | 0 |
| 959856 | 2017 | 12/13/2016 | WHEAT FLOUR, BROMATED | Grains and Grain Products | Wheat | DOMESTIC | United States | Non-Detect | 2.7 | 0 |
| 983097 | 2017 | 12/13/2016 | WHEAT FLOUR, BROMATED | Grains and Grain Products | Wheat | DOMESTIC | United States | Non-Detect | 2.5 | 0 |
| 935516 | 2017 | 12/13/2016 | SOY BASE FORMULA PRODUCT, READY TO FEED | Baby Food Products | Baby Formula | DOMESTIC | United States | Non-Detect | 0.233 | 0 |
| 970980 | 2017 | 12/14/2016 | OATS, WHOLE GRAIN | Grains and Grain Products | Oats | DOMESTIC | United States | Non-Detect | 2.5 | 0 |
| 991115 | 2017 | 12/14/2016 | WHEAT FLOUR, WHOLE | Grains and Grain Products | Wheat | DOMESTIC | United States | Non-Detect | 2.5 | 0 |
| 991977 | 2017 | 12/21/2016 | OATMEAL, REGULAR, FRUIT OR SPICE ADDED, QUICK OR INSTANT COOKING | Processed Food Products | Breakfast Foods | IMPORT | Canada | Non-Detect | 1 | 0 |
| 976712 | 2017 | 12/27/2016 | RYE FLOUR | Grains and Grain Products | Rye | DOMESTIC | United States | Non-Detect | 2.5 | 0 |
| 976713 | 2017 | 12/27/2016 | WHEAT FLOUR, WHOLE | Grains and Grain Products | Wheat | DOMESTIC | United States | Non-Detect | 2.5 | 0 |
| 992701 | 2017 | 12/29/2016 | BUCKWHEAT, WHOLE GRAIN | Grains and Grain Products | Buckwheat | IMPORT | Russia | Non-Detect | 1 | 0 |
| 992998 | 2017 | 12/30/2016 | RAISINS, DRIED OR PASTE | Fruit | Raisins | IMPORT | China | Non-Detect | 1 | 0 |
| 993346 | 2017 | 1/6/2017 | OATS, WHOLE GRAIN | Grains and Grain Products | Oats | IMPORT | Canada | Non-Detect | 2.5 | 0 |
| 994175 | 2017 | 1/6/2017 | WHEAT FLOUR, ENRICHED (ALL PURPOSE) | Grains and Grain Products | Wheat | IMPORT | Canada | Non-Detect | 2.5 | 0 |
| 993402 | 2017 | 1/6/2017 | KIDNEY BEAN | Beans and Legumes | Other Beans and Legumes | IMPORT | Nicaragua | Non-Detect | 0.233 | 0 |
| 993535 | 2017 | 1/9/2017 | WHEAT FLOUR, ENRICHED (ALL PURPOSE) | Grains and Grain Products | Wheat | IMPORT | Canada | Non-Detect | 2.5 | 0 |
| 940075 | 2017 | 1/9/2017 | SOY BASE FORMULA PRODUCT, READY TO FEED | Baby Food Products | Baby Formula | DOMESTIC | United States | Non-Detect | 0.233 | 0 |
| 993541 | 2017 | 1/9/2017 | CORN, PLAIN MEAL | Grains and Grain Products | Corn | IMPORT | Guatemala | Non-Detect | 0.233 | 0 |
| 940076 | 2017 | 1/10/2017 | WHEAT, WHOLE GRAIN | Grains and Grain Products | Wheat | DOMESTIC | United States | Non-Detect | 0.233 | 0 |
| 993801 | 2017 | 1/10/2017 | MALT, BARLEY | Grains and Grain Products | Barley | IMPORT | France | Trace | 1 | |
| 983098 | 2017 | 1/11/2017 | MALT, BARLEY | Grains and Grain Products | Barley | DOMESTIC | United States | Non-Detect | 2.5 | 0 |
| 970919 | 2017 | 1/17/2017 | WHEAT FLOUR, PLAIN | Grains and Grain Products | Wheat | DOMESTIC | United States | Non-Detect | 0.233 | 0 |
| 989622 | 2017 | 1/17/2017 | RYE FLOUR | Grains and Grain Products | Rye | DOMESTIC | United States | Positive | 4.99 | 5.61 |
| 986609 | 2017 | 1/18/2017 | MALT, BARLEY | Grains and Grain Products | Barley | DOMESTIC | United States | Non-Detect | 1 | 0 |
| 986608 | 2017 | 1/18/2017 | MALT, BARLEY | Grains and Grain Products | Barley | DOMESTIC | United States | Trace | 1 | |
| 994996 | 2017 | 1/19/2017 | BARLEY, WHOLE GRAIN | Grains and Grain Products | Barley | IMPORT | China | Trace | 1 | |
| 989623 | 2017 | 1/23/2017 | RYE FLOUR | Grains and Grain Products | Rye | DOMESTIC | United States | Non-Detect | 0.233 | 0 |
| 995547 | 2017 | 1/23/2017 | AZUFRADO BEAN, DRIED OR PASTE | Beans and Legumes | Other Beans and Legumes | IMPORT | Mexico | Non-Detect | 0.233 | 0 |
| 995624 | 2017 | 1/24/2017 | BUCKWHEAT, WHOLE GRAIN | Grains and Grain Products | Buckwheat | IMPORT | Russia | Non-Detect | 1 | 0 |
| 977939 | 2017 | 1/25/2017 | MALT, BARLEY | Grains and Grain Products | Barley | DOMESTIC | United States | Non-Detect | 2.5 | 0 |

| | | | | | | | | | | |
|---|---|---|---|---|---|---|---|---|---|---|
| 996082 | 2017 | 1/26/2017 | AZUFRADO BEAN, DRIED OR PASTE | Beans and Legumes | Other Beans and Legumes | IMPORT | Peru | Non-Detect | 1 | 0 |
| 892446 | 2017 | 1/27/2017 | WHEAT FLOUR, PLAIN | Grains and Grain Products | Wheat | DOMESTIC | United States | Non-Detect | 0.233 | 0 |
| 996181 | 2017 | 1/27/2017 | RED BEAN | Beans and Legumes | Other Beans and Legumes | IMPORT | Nicaragua | Non-Detect | 0.233 | 0 |
| 996284 | 2017 | 1/27/2017 | FAVA BEAN | Beans and Legumes | Other Beans and Legumes | IMPORT | Mexico | Non-Detect | 0.233 | 0 |
| 996444 | 2017 | 1/30/2017 | MEDIUM FAT COCOA (EXCEPT CHOCOLATE CANDY AND CHOCOLATE BEVERAGE BASE) | Candy and Chocolate Products | Other Chocolate Products | IMPORT | Mexico | Non-Detect | 2.4 | 0 |
| 894449 | 2017 | 1/30/2017 | RAISINS, DRIED OR PASTE | Fruit | Raisins | IMPORT | China | Non-Detect | 2.629 | 0 |
| 996358 | 2017 | 1/30/2017 | FLOURS AND MEALS N.E.C. | Grains and Grain Products | Other Grains | IMPORT | Canada | Non-Detect | 1 | 0 |
| 996703 | 2017 | 1/31/2017 | COFFEE, BEANS | Non-Juice Beverages | Coffee | IMPORT | Colombia | Non-Detect | 2.5 | 0 |
| 996705 | 2017 | 1/31/2017 | COFFEE, BEANS | Non-Juice Beverages | Coffee | IMPORT | Colombia | Non-Detect | 2.5 | 0 |
| 938078 | 2017 | 1/31/2017 | CORN, ENRICHED MEAL | Grains and Grain Products | Corn | DOMESTIC | Unknown | Non-Detect | 1 | 0 |
| 938079 | 2017 | 1/31/2017 | CORN, ENRICHED MEAL | Grains and Grain Products | Corn | DOMESTIC | Unknown | Non-Detect | 1 | 0 |
| 997358 | 2017 | 2/3/2017 | COFFEE, BEANS | Non-Juice Beverages | Coffee | IMPORT | Honduras | Non-Detect | 2.5 | 0 |
| 997365 | 2017 | 2/3/2017 | COFFEE, BEANS | Non-Juice Beverages | Coffee | IMPORT | Honduras | Non-Detect | 2.5 | 0 |
| 997422 | 2017 | 2/3/2017 | COFFEE, BEANS | Non-Juice Beverages | Coffee | IMPORT | Guatemala | Non-Detect | 2.5 | 0 |
| 997439 | 2017 | 2/3/2017 | COFFEE, BEANS | Non-Juice Beverages | Coffee | IMPORT | Brazil | Non-Detect | 2.5 | 0 |
| 997326 | 2017 | 2/3/2017 | NAVY (WHITE) BEANS (BAKED BEANS, PORK AND BEANS), DRIED OR PASTE | Beans and Legumes | Other Beans and Legumes | IMPORT | Canada | Non-Detect | 0.233 | 0 |
| 997363 | 2017 | 2/3/2017 | CORN FLAKES, PUFFS, KRISPIES, LOOPS READY TO EAT | Processed Food Products | Other Corn Products | IMPORT | Guatemala | Non-Detect | 1 | 0 |
| 997579 | 2017 | 2/6/2017 | COFFEE, BEANS | Non-Juice Beverages | Coffee | IMPORT | Burundi | Non-Detect | 1 | 0 |
| 997585 | 2017 | 2/6/2017 | COFFEE, BEANS | Non-Juice Beverages | Coffee | IMPORT | Burundi | Non-Detect | 1 | 0 |
| 998237 | 2017 | 2/9/2017 | OATS, WHOLE GRAIN | Grains and Grain Products | Oats | IMPORT | Ireland | Non-Detect | 2.5 | 0 |
| 998288 | 2017 | 2/9/2017 | MALT, BARLEY | Grains and Grain Products | Barley | IMPORT | Canada | Non-Detect | 2.5 | 0 |
| 998380 | 2017 | 2/10/2017 | BARLEY, WHOLE GRAIN | Grains and Grain Products | Barley | IMPORT | Canada | Non-Detect | 1 | 0 |
| 956722 | 2017 | 2/13/2017 | WHEAT FLOUR, ENRICHED (ALL PURPOSE) | Grains and Grain Products | Wheat | DOMESTIC | Unknown | Non-Detect | 0.233 | 0 |
| 997901 | 2017 | 2/13/2017 | WHEAT FLOUR, BLEACHED | Grains and Grain Products | Wheat | DOMESTIC | Unknown | Non-Detect | 0.233 | 0 |
| 981169 | 2017 | 2/14/2017 | WHEAT FLOUR, BLEACHED | Grains and Grain Products | Wheat | DOMESTIC | United States | Non-Detect | 2.3 | 0 |
| 998698 | 2017 | 2/14/2017 | COFFEE, BEANS | Non-Juice Beverages | Coffee | IMPORT | Colombia | Non-Detect | 2.3 | 0 |
| 998689 | 2017 | 2/14/2017 | OATS, WHOLE GRAIN | Grains and Grain Products | Oats | IMPORT | Canada | Non-Detect | 1 | 0 |
| 993545 | 2017 | 2/15/2017 | WHEAT FLOUR, WHOLE | Grains and Grain Products | Wheat | DOMESTIC | United States | Non-Detect | 2.3 | 0 |
| 999181 | 2017 | 2/16/2017 | COFFEE, BEANS | Non-Juice Beverages | Coffee | IMPORT | Guatemala | Non-Detect | 2.3 | 0 |
| 972232 | 2017 | 2/16/2017 | OATS, WHOLE GRAIN | Grains and Grain Products | Oats | DOMESTIC | Unknown | Trace | 1 | |
| 999234 | 2017 | 2/16/2017 | FAVA BEAN, DRIED OR PASTE | Beans and Legumes | Other Beans and Legumes | IMPORT | Spain | Trace | 1 | |
| 999366 | 2017 | 2/17/2017 | AZUFRADO BEAN, DRIED OR PASTE | Beans and Legumes | Other Beans and Legumes | IMPORT | Mexico | Non-Detect | 0.233 | 0 |
| 999420 | 2017 | 2/21/2017 | OATMEAL, REGULAR, FRUIT OR SPICE ADDED, QUICK OR INSTANT COOKING | Processed Food Products | Breakfast Foods | IMPORT | Canada | Non-Detect | 2.4 | 0 |
| 999429 | 2017 | 2/21/2017 | MALT, BARLEY | Grains and Grain Products | Barley | IMPORT | United Kingdom | Positive | 5 | 7.59 |
| 999924 | 2017 | 2/22/2017 | TEMPURA FLOUR MIX | Grains and Grain Products | Other Grains | IMPORT | Thailand | Non-Detect | 2.4 | 0 |
| 966901 | 2017 | 2/22/2017 | WHEAT FLOUR, WHOLE | Grains and Grain Products | Wheat | DOMESTIC | United States | Non-Detect | 0.233 | 0 |
| 993641 | 2017 | 2/22/2017 | BUCKWHEAT FLOUR | Grains and Grain Products | Buckwheat | DOMESTIC | United States | Non-Detect | 1 | 0 |
| 999834 | 2017 | 2/23/2017 | OATMEAL, REGULAR, FRUIT OR SPICE ADDED, QUICK OR INSTANT COOKING | Processed Food Products | Breakfast Foods | IMPORT | Canada | Non-Detect | 2.4 | 0 |
| 999836 | 2017 | 2/23/2017 | OATMEAL, REGULAR, FRUIT OR SPICE ADDED, QUICK OR INSTANT COOKING | Processed Food Products | Breakfast Foods | IMPORT | Canada | Non-Detect | 2.4 | 0 |
| 999837 | 2017 | 2/23/2017 | OATMEAL, REGULAR, FRUIT OR SPICE ADDED, QUICK OR INSTANT COOKING | Processed Food Products | Breakfast Foods | IMPORT | Canada | Non-Detect | 2.4 | 0 |
| 944111 | 2017 | 2/23/2017 | WHEAT FLOUR, PLAIN | Grains and Grain Products | Wheat | DOMESTIC | United States | Non-Detect | 0.233 | 0 |
| 968781 | 2017 | 2/23/2017 | SOY BASE FORMULA PRODUCT, READY TO FEED | Baby Food Products | Baby Formula | DOMESTIC | United States | Non-Detect | 0.233 | 0 |
| 979410 | 2017 | 2/23/2017 | RICE CEREAL (BABY) | Baby Food Products | Baby Cereals | DOMESTIC | United States | Non-Detect | 0.233 | 0 |
| 999873 | 2017 | 2/23/2017 | WHEAT FLOUR, WHOLE | Grains and Grain Products | Wheat | IMPORT | United Arab Emirates | Non-Detect | 1 | 0 |
| 999908 | 2017 | 2/23/2017 | WHEAT FLOUR, N.E.C. | Grains and Grain Products | Wheat | IMPORT | Italy | Non-Detect | 1 | 0 |
| 999938 | 2017 | 2/23/2017 | CORN CHIPS, FRIED | Processed Food Products | Other Corn Products | IMPORT | Guatemala | Non-Detect | 1 | 0 |
| 999942 | 2017 | 2/23/2017 | CORN CHIPS, FRIED | Processed Food Products | Other Corn Products | IMPORT | Guatemala | Non-Detect | 1 | 0 |
| 1000154 | 2017 | 2/24/2017 | BUCKWHEAT, WHOLE GRAIN | Grains and Grain Products | Buckwheat | IMPORT | China | Non-Detect | 1 | 0 |
| 1000196 | 2017 | 2/24/2017 | CORN BEVERAGE BASE | Non-Juice Beverages | Other Beverages | IMPORT | Mexico | Non-Detect | 1 | 0 |
| 1000199 | 2017 | 2/24/2017 | CORN BEVERAGE BASE | Non-Juice Beverages | Other Beverages | IMPORT | Mexico | Non-Detect | 1 | 0 |
| 1000390 | 2017 | 2/27/2017 | TORTILLAS | Processed Food Products | Other Corn Products | IMPORT | El Salvador | Non-Detect | 1 | 0 |
| 1001193 | 2017 | 2/28/2017 | GINGER, GROUND, CRACKED (SPICE) | Spices | Ginger | IMPORT | India | Non-Detect | 0.233 | 0 |
| 1000722 | 2017 | 2/28/2017 | CORN, ENRICHED MEAL | Grains and Grain Products | Corn | IMPORT | El Salvador | Non-Detect | 1 | 0 |
| 1001157 | 2017 | 3/2/2017 | OAT FLOUR | Grains and Grain Products | Oats | DOMESTIC | United States | Non-Detect | 0.233 | 0 |
| 1001227 | 2017 | 3/2/2017 | WHEAT, WHOLE GRAIN | Grains and Grain Products | Wheat | IMPORT | Canada | Non-Detect | 1 | 0 |
| 1001877 | 2017 | 3/7/2017 | WHEAT, WHOLE GRAIN | Grains and Grain Products | Wheat | IMPORT | Canada | Non-Detect | 1 | 0 |
| 985312 | 2017 | 3/8/2017 | RICE CEREAL (BABY) | Baby Food Products | Baby Cereals | DOMESTIC | United States | Non-Detect | 1 | 0 |
| 957507 | 2017 | 3/9/2017 | OAT FLOUR | Grains and Grain Products | Oats | DOMESTIC | United States | Non-Detect | 0.233 | 0 |
| 985313 | 2017 | 3/9/2017 | OAT CEREAL (BABY) | Baby Food Products | Baby Cereals | DOMESTIC | United States | Trace | 1 | |
| 1002493 | 2017 | 3/10/2017 | AZUFRADO BEAN, DRIED OR PASTE | Beans and Legumes | Other Beans and Legumes | IMPORT | Mexico | Non-Detect | 0.233 | 0 |
| 1002518 | 2017 | 3/10/2017 | FAVA BEAN, DRIED OR PASTE | Beans and Legumes | Other Beans and Legumes | IMPORT | Mexico | Non-Detect | 0.233 | 0 |
| 1002754 | 2017 | 3/10/2017 | BARLEY, WHOLE GRAIN | Grains and Grain Products | Barley | IMPORT | Russia | Non-Detect | 1 | 0 |
| 1002757 | 2017 | 3/10/2017 | BARLEY, WHOLE GRAIN | Grains and Grain Products | Barley | IMPORT | Russia | Non-Detect | 1 | 0 |
| 995882 | 2017 | 3/13/2017 | WHEAT FLOUR, WHOLE | Grains and Grain Products | Wheat | DOMESTIC | United States | Non-Detect | 1 | 0 |
| 978649 | 2017 | 3/15/2017 | WHEAT FLOUR, N.E.C. | Grains and Grain Products | Wheat | DOMESTIC | United States | Non-Detect | 0.233 | 0 |
| 1003233 | 2017 | 3/15/2017 | KIDNEY BEAN, DRIED OR PASTE | Beans and Legumes | Other Beans and Legumes | IMPORT | Nicaragua | Non-Detect | 1 | 0 |
| 1003512 | 2017 | 3/15/2017 | CORN, BOLTED MEAL OR FLOUR | Grains and Grain Products | Corn | IMPORT | Argentina | Non-Detect | 1 | 0 |
| 1003452 | 2017 | 3/16/2017 | CORN FLAKES, PUFFS, KRISPIES, LOOPS READY TO EAT | Processed Food Products | Other Corn Products | IMPORT | Japan | Non-Detect | 1 | 0 |
| 999383 | 2017 | 3/17/2017 | RYE FLOUR | Grains and Grain Products | Rye | DOMESTIC | United States | Non-Detect | 0.233 | 0 |
| 1003592 | 2017 | 3/19/2017 | OATMEAL, REGULAR, FRUIT OR SPICE ADDED, QUICK OR INSTANT COOKING | Processed Food Products | Breakfast Foods | IMPORT | Canada | Non-Detect | 1 | 0 |
| 995734 | 2017 | 3/20/2017 | KIDNEY BEAN, DRIED OR PASTE | Beans and Legumes | Other Beans and Legumes | DOMESTIC | United States | Non-Detect | 0.233 | 0 |
| 1004186 | 2017 | 3/21/2017 | BEAN, DRIED OR PASTE, N.E.C. (VEGETABLE) | Beans and Legumes | Other Beans and Legumes | IMPORT | Mexico | Non-Detect | 0.233 | 0 |
| 1004197 | 2017 | 3/21/2017 | GARBANZO BEAN, DRIED OR PASTE | Beans and Legumes | Garbanzo Beans | IMPORT | Mexico | Non-Detect | 0.233 | 0 |
| 1004772 | 2017 | 3/22/2017 | WHEAT, WHOLE GRAIN | Grains and Grain Products | Wheat | IMPORT | Canada | Non-Detect | 0.233 | 0 |
| 1004151 | 2017 | 3/22/2017 | GINGER, GROUND, CRACKED (SPICE) | Spices | Ginger | IMPORT | China | Non-Detect | 1 | 0 |
| 1004389 | 2017 | 3/23/2017 | FAVA BEAN | Beans and Legumes | Other Beans and Legumes | IMPORT | China | Non-Detect | 1 | 0 |
| 1003557 | 2017 | 3/24/2017 | LIMA BEAN, DRIED OR PASTE | Beans and Legumes | Other Beans and Legumes | DOMESTIC | United States | Non-Detect | 12 | 0 |
| 1003558 | 2017 | 3/24/2017 | BEAN, DRIED OR PASTE, N.E.C. (VEGETABLE) | Beans and Legumes | Other Beans and Legumes | DOMESTIC | United States | Non-Detect | 12 | 0 |
| 1005667 | 2017 | 3/29/2017 | COFFEE, BEANS | Non-Juice Beverages | Coffee | IMPORT | Kenya | Non-Detect | 12 | 0 |
| 1005678 | 2017 | 3/29/2017 | COFFEE, BEANS | Non-Juice Beverages | Coffee | IMPORT | Ethiopia | Non-Detect | 12 | 0 |
| 1005685 | 2017 | 3/29/2017 | COFFEE, BEANS | Non-Juice Beverages | Coffee | IMPORT | Rwanda | Non-Detect | 12 | 0 |
| 1005300 | 2017 | 3/29/2017 | FLOURS AND MEALS N.E.C. | Grains and Grain Products | Other Grains | IMPORT | Canada | Non-Detect | 0.233 | 0 |
| 1005487 | 2017 | 3/29/2017 | OATS, WHOLE GRAIN | Grains and Grain Products | Oats | IMPORT | Canada | Non-Detect | 1 | 0 |
| 1005707 | 2017 | 3/30/2017 | WHEAT FLAKES, PUFFS, KRISPIES, LOOPS, SHREDDED, WHEAT GERM READY TO EAT | Processed Food Products | Breakfast Foods | IMPORT | Mexico | Non-Detect | 2.2 | 0 |
| 1005709 | 2017 | 3/30/2017 | WHEAT FLAKES, PUFFS, KRISPIES, LOOPS, SHREDDED, WHEAT GERM READY TO EAT | Processed Food Products | Breakfast Foods | IMPORT | Mexico | Non-Detect | 2.2 | 0 |
| 1005786 | 2017 | 3/30/2017 | CORN CHIPS, FRIED | Processed Food Products | Other Corn Products | IMPORT | Korea (the Republic of) | Non-Detect | 1 | 0 |

| | | | | | | | | | | |
|---|---|---|---|---|---|---|---|---|---|---|
| 1005953 | 2017 | 3/31/2017 | MALT, BARLEY | Grains and Grain Products | Barley | IMPORT | Germany | Non-Detect | 1 | 0 |
| 1006002 | 2017 | 4/3/2017 | WHEAT, WHOLE GRAIN | Grains and Grain Products | Wheat | DOMESTIC | United States | Non-Detect | 12 | 0 |
| 1003615 | 2017 | 4/3/2017 | SOY BASE FORMULA PRODUCT, LIQUID CONCENTRATE | Baby Food Products | Baby Formula | DOMESTIC | United States | Non-Detect | 0.233 | 0 |
| 1006159 | 2017 | 4/3/2017 | CORN FLAKES, PUFFS, KRISPIES, LOOPS READY TO EAT | Processed Food Products | Other Corn Products | IMPORT | Japan | Non-Detect | 1 | 0 |
| 1006383 | 2017 | 4/4/2017 | SOYBEANS (SEE INDUSTRY 37 FOR BEAN CURD), DRIED OR PASTE | Beans and Legumes | Soybeans | IMPORT | Canada | Non-Detect | 0.233 | 0 |
| 1006332 | 2017 | 4/4/2017 | RAISINS, DRIED OR PASTE | Fruit | Raisins | IMPORT | Pakistan | Non-Detect | 1 | 0 |
| 1006498 | 2017 | 4/5/2017 | PINTO BEAN, DRIED OR PASTE | Beans and Legumes | Pinto Beans | IMPORT | United States | Non-Detect | 0.233 | 0 |
| 1007093 | 2017 | 4/6/2017 | COFFEE, BEANS | Non-Juice Beverages | Coffee | IMPORT | Ethiopia | Non-Detect | 12 | 0 |
| 1007072 | 2017 | 4/6/2017 | CORN, BOLTED MEAL OR FLOUR | Grains and Grain Products | Corn | IMPORT | India | Positive | 1 | 15.7 |
| 1003559 | 2017 | 4/7/2017 | RICE CEREAL (BABY) | Baby Food Products | Baby Cereals | DOMESTIC | United States | Non-Detect | 12 | 0 |
| 1007526 | 2017 | 4/10/2017 | GARBANZO BEAN | Beans and Legumes | Garbanzo Beans | IMPORT | Canada | Non-Detect | 1 | 0 |
| 1001120 | 2017 | 4/12/2017 | BEAN, DRIED OR PASTE, N.E.C. (VEGETABLE) | Beans and Legumes | Other Beans and Legumes | DOMESTIC | United States | Non-Detect | 0.233 | 0 |
| 1007680 | 2017 | 4/12/2017 | COFFEE, BEANS | Non-Juice Beverages | Coffee | IMPORT | South Africa | Non-Detect | 0.96 | 0 |
| 1007764 | 2017 | 4/12/2017 | BARLEY, WHOLE GRAIN | Grains and Grain Products | Barley | IMPORT | Korea (the Republic of) | Non-Detect | 1 | 0 |
| 1008380 | 2017 | 4/18/2017 | OATS, WHOLE GRAIN | Grains and Grain Products | Oats | IMPORT | Canada | Non-Detect | 1 | 0 |
| 1008666 | 2017 | 4/19/2017 | COFFEE, BEANS | Non-Juice Beverages | Coffee | IMPORT | Honduras | Non-Detect | 12 | 0 |
| 943220 | 2017 | 4/19/2017 | RAISINS, DRIED OR PASTE | Fruit | Raisins | DOMESTIC | United States | Non-Detect | 2.5 | 0 |
| 974916 | 2017 | 4/19/2017 | WHEAT FLOUR, N.E.C. | Grains and Grain Products | Wheat | DOMESTIC | United States | Non-Detect | 0.233 | 0 |
| 1008512 | 2017 | 4/19/2017 | COFFEE, BEANS | Non-Juice Beverages | Coffee | IMPORT | Colombia | Non-Detect | 12.15 | 0 |
| 1008505 | 2017 | 4/19/2017 | COFFEE, BEANS | Non-Juice Beverages | Coffee | IMPORT | Indonesia | Trace | 5 | |
| 1009033 | 2017 | 4/24/2017 | MALT, BARLEY | Grains and Grain Products | Barley | IMPORT | France | Trace | 5 | |
| 1009568 | 2017 | 4/25/2017 | WHEAT FLOUR, N.E.C. | Grains and Grain Products | Wheat | IMPORT | United States | Non-Detect | 12 | 0 |
| 1009405 | 2017 | 4/26/2017 | BUCKWHEAT FLOUR | Grains and Grain Products | Buckwheat | IMPORT | Russia | Non-Detect | 1 | 0 |
| 1009538 | 2017 | 4/27/2017 | CORN FLAKES, PUFFS, KRISPIES, LOOPS READY TO EAT | Processed Food Products | Other Corn Products | IMPORT | Mexico | Non-Detect | 1 | 0 |
| 995153 | 2017 | 4/27/2017 | OATS, WHOLE GRAIN | Grains and Grain Products | Oats | IMPORT | Canada | Non-Detect | 1 | 0 |
| 1009957 | 2017 | 5/1/2017 | COFFEE, BEANS | Non-Juice Beverages | Coffee | IMPORT | Guatemala | Non-Detect | 12 | 0 |
| 1009756 | 2017 | 5/1/2017 | BUCKWHEAT FLOUR | Grains and Grain Products | Buckwheat | DOMESTIC | United States | Positive | 5 | 2.35 |
| 968914 | 2017 | 5/9/2017 | BEANS, N.E.C. (VEGETABLE) | Beans and Legumes | Other Beans and Legumes | DOMESTIC | United States | Non-Detect | 0.233 | 0 |
| 1004899 | 2017 | 5/10/2017 | WHEAT, WHOLE GRAIN | Grains and Grain Products | Wheat | DOMESTIC | United States | Non-Detect | 1 | 0 |
| 1010391 | 2017 | 5/10/2017 | WHEAT FLOUR, PLAIN | Grains and Grain Products | Wheat | DOMESTIC | United States | Non-Detect | 1 | 0 |
| 1011147 | 2017 | 5/10/2017 | RAISINS, DRIED OR PASTE | Fruit | Raisins | IMPORT | Australia | Positive | 1 | 4.6 |
| 986507 | 2017 | 5/17/2017 | WHEAT FLOUR, BLEACHED | Grains and Grain Products | Wheat | DOMESTIC | United States | Non-Detect | 0.233 | 0 |
| 1012255 | 2017 | 5/22/2017 | WHEAT, WHOLE GRAIN | Grains and Grain Products | Wheat | IMPORT | Canada | Non-Detect | 1 | 0 |
| 979791 | 2017 | 5/23/2017 | FARINA, WHEAT | Grains and Grain Products | Wheat | DOMESTIC | United States | Non-Detect | 2.4 | 0 |
| 996762 | 2017 | 5/23/2017 | RICE CEREAL (BABY) | Baby Food Products | Baby Cereals | DOMESTIC | United States | Non-Detect | 12 | 0 |
| 996764 | 2017 | 5/23/2017 | CORN, HOMINY GRITS | Grains and Grain Products | Corn | DOMESTIC | United States | Non-Detect | 1 | 0 |
| 1012600 | 2017 | 5/24/2017 | AZUFRADO BEAN, DRIED OR PASTE | Beans and Legumes | Other Beans and Legumes | IMPORT | Mexico | Non-Detect | 0.233 | 0 |
| 1012774 | 2017 | 5/24/2017 | CORN CHIPS, FRIED | Processed Food Products | Other Corn Products | IMPORT | Mexico | Non-Detect | 0.233 | 0 |
| 1012860 | 2017 | 5/25/2017 | OATS, WHOLE GRAIN | Grains and Grain Products | Oats | IMPORT | Canada | Non-Detect | 1 | 0 |
| 1013135 | 2017 | 5/30/2017 | MALT, BARLEY | Grains and Grain Products | Barley | IMPORT | France | Non-Detect | 0.233 | 0 |
| 1013229 | 2017 | 5/31/2017 | COFFEE, BEANS | Non-Juice Beverages | Coffee | IMPORT | Colombia | Non-Detect | 12 | 0 |
| 1013257 | 2017 | 5/31/2017 | WHEAT, WHOLE GRAIN | Grains and Grain Products | Wheat | IMPORT | Italy | Non-Detect | 12 | 0 |
| 1013454 | 2017 | 5/31/2017 | BARLEY, WHOLE GRAIN | Grains and Grain Products | Barley | IMPORT | Canada | Non-Detect | 12 | 0 |
| 1013527 | 2017 | 6/1/2017 | COFFEE, BEANS | Non-Juice Beverages | Coffee | IMPORT | Tanzania, United Republic Of | Non-Detect | 12 | 0 |
| 1013700 | 2017 | 6/2/2017 | KIDNEY BEAN, DRIED OR PASTE | Beans and Legumes | Other Beans and Legumes | IMPORT | Dominican Republic (the) | Non-Detect | 12 | 0 |
| 991158 | 2017 | 6/2/2017 | RYE FLOUR | Grains and Grain Products | Rye | DOMESTIC | United States | Non-Detect | 0.233 | 0 |
| 1013710 | 2017 | 6/2/2017 | MALT, BARLEY | Grains and Grain Products | Barley | IMPORT | Germany | Non-Detect | 1 | 0 |
| 961329 | 2017 | 6/7/2017 | RICE CEREAL (BABY) | Baby Food Products | Baby Cereals | DOMESTIC | United States | Non-Detect | 2.5 | 0 |
| 1014232 | 2017 | 6/7/2017 | BARLEY, WHOLE GRAIN | Grains and Grain Products | Barley | IMPORT | Hong Kong SAR | Non-Detect | 0.233 | 0 |
| 1014437 | 2017 | 6/8/2017 | OATS, WHOLE GRAIN | Grains and Grain Products | Oats | IMPORT | Russia | Non-Detect | 1 | 0 |
| 1001128 | 2017 | 6/12/2017 | KIDNEY BEAN, DRIED OR PASTE | Beans and Legumes | Other Beans and Legumes | DOMESTIC | United States | Non-Detect | 0.233 | 0 |
| 1015023 | 2017 | 6/14/2017 | RAISINS, DRIED OR PASTE | Fruit | Raisins | IMPORT | China | Non-Detect | 1 | 0 |
| 1015557 | 2017 | 6/15/2017 | COFFEE, BEANS | Non-Juice Beverages | Coffee | IMPORT | Colombia | Non-Detect | 12 | 0 |
| 1015566 | 2017 | 6/15/2017 | COFFEE, BEANS | Non-Juice Beverages | Coffee | IMPORT | Indonesia | Non-Detect | 12 | 0 |
| 1015570 | 2017 | 6/15/2017 | COFFEE, BEANS | Non-Juice Beverages | Coffee | IMPORT | Ethiopia | Non-Detect | 12 | 0 |
| 1015580 | 2017 | 6/15/2017 | COFFEE, BEANS | Non-Juice Beverages | Coffee | IMPORT | Ethiopia | Non-Detect | 12 | 0 |
| 1015734 | 2017 | 6/16/2017 | OATS, WHOLE GRAIN | Grains and Grain Products | Oats | IMPORT | Canada | Non-Detect | 1 | 0 |
| 1016429 | 2017 | 6/20/2017 | PISTACHIO, IN SHELL | Nuts and Edible Seeds | Pistachios | IMPORT | United States | Non-Detect | 1 | 0 |
| 1016761 | 2017 | 6/20/2017 | COFFEE, BEANS | Non-Juice Beverages | Coffee | IMPORT | Colombia | Non-Detect | 1 | 0 |
| 1016782 | 2017 | 6/20/2017 | GARBANZO BEAN, DRIED OR PASTE | Beans and Legumes | Garbanzo Beans | IMPORT | Argentina | Non-Detect | 1 | 0 |
| 1016708 | 2017 | 6/22/2017 | WHEAT, WHOLE GRAIN | Grains and Grain Products | Wheat | IMPORT | Canada | Non-Detect | 1 | 0 |
| 1016841 | 2017 | 6/26/2017 | BARLEY, WHOLE GRAIN | Grains and Grain Products | Barley | IMPORT | China | Non-Detect | 1 | 0 |
| 1002960 | 2017 | 6/27/2017 | RICE CEREAL (BABY) | Baby Food Products | Baby Cereals | DOMESTIC | United States | Non-Detect | 12 | 0 |
| 1002961 | 2017 | 6/27/2017 | RICE CEREAL (BABY) | Baby Food Products | Baby Cereals | DOMESTIC | United States | Non-Detect | 12 | 0 |
| 1002962 | 2017 | 6/27/2017 | BARLEY, WHOLE GRAIN | Grains and Grain Products | Barley | DOMESTIC | United States | Non-Detect | 12 | 0 |
| 1017058 | 2017 | 6/27/2017 | COFFEE, BEANS | Non-Juice Beverages | Coffee | IMPORT | Indonesia | Non-Detect | 12 | 0 |
| 945263 | 2017 | 6/27/2017 | SOY BASE FORMULA PRODUCT, LIQUID CONCENTRATE | Baby Food Products | Baby Formula | DOMESTIC | United States | Non-Detect | 0.233 | 0 |
| 945264 | 2017 | 6/27/2017 | SOY BASE FORMULA PRODUCT, LIQUID CONCENTRATE | Baby Food Products | Baby Formula | DOMESTIC | United States | Non-Detect | 0.233 | 0 |
| 1017145 | 2017 | 6/27/2017 | MALT, BARLEY | Grains and Grain Products | Barley | IMPORT | United Kingdom | Non-Detect | 0.233 | 0 |
| 1017035 | 2017 | 6/27/2017 | COFFEE, BEANS | Non-Juice Beverages | Coffee | IMPORT | El Salvador | Trace | 5 | |
| 1017330 | 2017 | 6/28/2017 | CHANA DAL | Beans and Legumes | Garbanzo Beans | IMPORT | India | Non-Detect | 12 | 0 |
| 1017331 | 2017 | 6/28/2017 | MALT, BARLEY | Grains and Grain Products | Barley | IMPORT | United Kingdom | Non-Detect | 12 | 0 |
| 1017421 | 2017 | 6/28/2017 | LENTILS | Beans and Legumes | Lentils | IMPORT | Canada | Non-Detect | 1 | 0 |
| 1017423 | 2017 | 6/28/2017 | PEA, DRIED OR PASTE | Beans and Legumes | Peas | IMPORT | Canada | Non-Detect | 1 | 0 |
| 1017458 | 2017 | 6/28/2017 | WHEAT, WHOLE GRAIN | Grains and Grain Products | Wheat | IMPORT | Canada | Non-Detect | 1 | 0 |
| 1017447 | 2017 | 6/28/2017 | WHEAT, WHOLE GRAIN | Grains and Grain Products | Wheat | IMPORT | Canada | Positive | 1 | 3.2 |
| 1018308 | 2017 | 7/7/2017 | BUCKWHEAT, WHOLE GRAIN | Grains and Grain Products | Buckwheat | IMPORT | Russia | Non-Detect | 0.233 | 0 |
| 1018072 | 2017 | 7/7/2017 | AZUFRADO BEAN, DRIED OR PASTE | Beans and Legumes | Other Beans and Legumes | IMPORT | Mexico | Non-Detect | 1 | 0 |
| 1018364 | 2017 | 7/10/2017 | MALT, BARLEY | Grains and Grain Products | Barley | IMPORT | United Kingdom | Non-Detect | 12 | 0 |
| 1018500 | 2017 | 7/10/2017 | GARBANZO BEAN | Beans and Legumes | Garbanzo Beans | IMPORT | India | Non-Detect | 12 | 0 |
| 1018515 | 2017 | 7/10/2017 | COFFEE, GROUND | Non-Juice Beverages | Coffee | IMPORT | India | Non-Detect | 12 | 0 |
| 1018617 | 2017 | 7/10/2017 | COFFEE, GROUND | Non-Juice Beverages | Coffee | IMPORT | India | Non-Detect | 12 | 0 |
| 1018432 | 2017 | 7/10/2017 | AZUFRADO BEAN, DRIED OR PASTE | Beans and Legumes | Other Beans and Legumes | IMPORT | Mexico | Non-Detect | 0.233 | 0 |
| 1018520 | 2017 | 7/12/2017 | MALT, BARLEY | Grains and Grain Products | Barley | IMPORT | United Kingdom | Non-Detect | 12 | 0 |
| 1014568 | 2017 | 7/12/2017 | RAISINS (DRIED GRAPES) (BERRY) | Fruit | Raisins | DOMESTIC | United States | Non-Detect | 1 | 0 |
| 983430 | 2017 | 7/12/2017 | CORN, PLAIN MEAL | Grains and Grain Products | Corn | DOMESTIC | United States | Non-Detect | 1 | 0 |

| | | | | | | | | | | |
|---|---|---|---|---|---|---|---|---|---|---|
| 983431 | 2017 | 7/12/2017 | CORN FLAKES, PUFFS, KRISPIES, LOOPS READY TO EAT | Processed Food Products | Other Corn Products | DOMESTIC | United States | Non-Detect | 1 | 0 |
| 983432 | 2017 | 7/12/2017 | RICE CEREAL (BABY) | Baby Food Products | Baby Cereals | DOMESTIC | United States | Non-Detect | 1 | 0 |
| 1014569 | 2017 | 7/12/2017 | RAISINS (DRIED GRAPES) (BERRY) | Fruit | Raisins | DOMESTIC | United States | Trace | 1 | |
| 1018971 | 2017 | 7/13/2017 | COFFEE, BEANS | Non-Juice Beverages | Coffee | IMPORT | Ethiopia | Non-Detect | 12.15 | 0 |
| 1018766 | 2017 | 7/13/2017 | MALT, BARLEY | Grains and Grain Products | Barley | IMPORT | Germany | Non-Detect | 1 | 0 |
| 1018813 | 2017 | 7/13/2017 | COFFEE, BEANS | Non-Juice Beverages | Coffee | IMPORT | Congo, Dem Rep of (Kinshasa) | Non-Detect | 1 | 0 |
| 979914 | 2017 | 7/13/2017 | BARLEY, WHOLE GRAIN | Grains and Grain Products | Barley | DOMESTIC | Unknown | Non-Detect | 1 | 0 |
| 979915 | 2017 | 7/13/2017 | BARLEY, WHOLE GRAIN | Grains and Grain Products | Barley | DOMESTIC | Unknown | Non-Detect | 1 | 0 |
| 1018972 | 2017 | 7/17/2017 | COFFEE, BEANS | Non-Juice Beverages | Coffee | IMPORT | Ethiopia | Non-Detect | 12.15 | 0 |
| 979916 | 2017 | 7/17/2017 | WHEAT, WHOLE GRAIN | Grains and Grain Products | Wheat | DOMESTIC | Unknown | Non-Detect | 1 | 0 |
| 979917 | 2017 | 7/17/2017 | WHEAT, WHOLE GRAIN | Grains and Grain Products | Wheat | DOMESTIC | Unknown | Trace | 1 | |
| 1019170 | 2017 | 7/18/2017 | BLACKEYE PEA, DRIED OR PASTE | Beans and Legumes | Blackeye Peas | IMPORT | China | Non-Detect | 1 | 0 |
| 1019175 | 2017 | 7/18/2017 | ADZUKI BEAN, DRIED OR PASTE | Beans and Legumes | Other Beans and Legumes | IMPORT | China | Non-Detect | 1 | 0 |
| 1019177 | 2017 | 7/18/2017 | MUNG BEAN, DRIED OR PASTE | Beans and Legumes | Other Beans and Legumes | IMPORT | China | Non-Detect | 1 | 0 |
| 1019241 | 2017 | 7/18/2017 | MALT, BARLEY | Grains and Grain Products | Barley | IMPORT | Czech Republic | Non-Detect | 1 | 0 |
| 979129 | 2017 | 7/19/2017 | FIG, DRIED OR PASTE | Fruit | Dried Figs | DOMESTIC | United States | Non-Detect | 0.233 | 0 |
| 1019470 | 2017 | 7/20/2017 | RAISINS, DRIED OR PASTE | Fruit | Raisins | IMPORT | Argentina | Trace | 5 | |
| 1019922 | 2017 | 7/25/2017 | BARLEY, WHOLE GRAIN | Grains and Grain Products | Barley | IMPORT | Ethiopia | Non-Detect | 1 | 0 |
| 1020093 | 2017 | 7/25/2017 | COFFEE, BEANS | Non-Juice Beverages | Coffee | IMPORT | Brazil | Non-Detect | 1 | 0 |
| 1020110 | 2017 | 7/25/2017 | WHEAT FLOUR, PLAIN SELF-RISING | Grains and Grain Products | Wheat | IMPORT | Ecuador | Non-Detect | 1 | 0 |
| 1005617 | 2017 | 7/27/2017 | BARLEY, WHOLE GRAIN | Grains and Grain Products | Barley | IMPORT | Canada | Non-Detect | 1 | 0 |
| 940887 | 2017 | 7/28/2017 | FLUID/DRY MILK PRODUCTS NOT ELSEWHERE MENTIONED, N.E.C. | Dairy and Eggs | Dairy Milk and Milk Products | DOMESTIC | United States | Non-Detect | 2.5 | 0 |
| 1020718 | 2017 | 7/31/2017 | BARLEY FLOUR | Grains and Grain Products | Barley | IMPORT | Canada | Non-Detect | 0.233 | 0 |
| 970788 | 2017 | 7/31/2017 | NAVY (WHITE) BEANS (BAKED BEANS, PORK AND BEANS), DRIED OR PASTE | Beans and Legumes | Other Beans and Legumes | DOMESTIC | United States | Non-Detect | 1 | 0 |
| 970789 | 2017 | 7/31/2017 | BLACK BEAN, DRIED OR PASTE | Beans and Legumes | Black Beans | DOMESTIC | United States | Non-Detect | 1 | 0 |
| 1020568 | 2017 | 7/31/2017 | BEAN, DRIED OR PASTE, N.E.C. (VEGETABLE) | Beans and Legumes | Other Beans and Legumes | IMPORT | India | Trace | 5 | |
| 1020721 | 2017 | 8/1/2017 | RICE FLAKES, PUFFS, KRISPIES, LOOPS READY TO EAT | Processed Food Products | Breakfast Foods | IMPORT | Mexico | Non-Detect | 0.233 | 0 |
| 1021040 | 2017 | 8/2/2017 | RYE, WHOLE GRAIN | Grains and Grain Products | Rye | IMPORT | Canada | Non-Detect | 12 | 0 |
| 1020835 | 2017 | 8/2/2017 | WHOLE WHEAT BREAD, ROLLS, BUNS, ETC PREPARED DRY MIX WITHOUT MILK OR EGG | Processed Food Products | Bread | IMPORT | Germany | Non-Detect | 0.233 | 0 |
| 1013298 | 2017 | 8/2/2017 | BUCKWHEAT FLOUR | Grains and Grain Products | Buckwheat | DOMESTIC | Unknown | Positive | 5 | 3.47 |
| 1013299 | 2017 | 8/2/2017 | BUCKWHEAT FLOUR | Grains and Grain Products | Buckwheat | DOMESTIC | Unknown | Trace | 5 | |
| 1021051 | 2017 | 8/3/2017 | FLOURS AND MEALS N.E.C. | Grains and Grain Products | Other Grains | IMPORT | Germany | Trace | 5 | |
| 1021132 | 2017 | 8/4/2017 | BAKED SNACK FOOD, N.E.C. | Processed Food Products | Other Snack Foods | IMPORT | Hungary | Non-Detect | 12 | 0 |
| 871374 | 2017 | 8/7/2017 | RYE, WHOLE GRAIN | Grains and Grain Products | Rye | DOMESTIC | United States | Non-Detect | 2.5 | 0 |
| 1014909 | 2017 | 8/8/2017 | CORN CEREAL (BABY) | Baby Food Products | Baby Cereals | DOMESTIC | United States | Non-Detect | 12 | 0 |
| 997780 | 2017 | 8/8/2017 | WHEAT FLOUR, BROMATED | Grains and Grain Products | Wheat | DOMESTIC | United States | Non-Detect | 2.5 | 0 |
| 1021440 | 2017 | 8/8/2017 | AZUFRADO BEAN, DRIED OR PASTE | Beans and Legumes | Other Beans and Legumes | IMPORT | Mexico | Non-Detect | 1 | 0 |
| 1021482 | 2017 | 8/8/2017 | COFFEE, BEANS | Non-Juice Beverages | Coffee | IMPORT | Yemen | Non-Detect | 1 | 0 |
| 956754 | 2017 | 8/9/2017 | BUCKWHEAT FLOUR | Grains and Grain Products | Buckwheat | DOMESTIC | Unknown | Non-Detect | 0.233 | 0 |
| 1021603 | 2017 | 8/9/2017 | LIMA BEAN, DRIED OR PASTE | Beans and Legumes | Other Beans and Legumes | IMPORT | Peru | Non-Detect | 0.233 | 0 |
| 982894 | 2017 | 8/10/2017 | WHEAT, WHOLE GRAIN | Grains and Grain Products | Wheat | DOMESTIC | United States | Non-Detect | 2.5 | 0 |
| 1006359 | 2017 | 8/10/2017 | WHEAT FLOUR, N.E.C. | Grains and Grain Products | Wheat | DOMESTIC | United States | Non-Detect | 0.233 | 0 |
| 1016728 | 2017 | 8/10/2017 | BUCKWHEAT FLOUR | Grains and Grain Products | Buckwheat | DOMESTIC | United States | Non-Detect | 0.233 | 0 |
| 1021715 | 2017 | 8/10/2017 | CORN FLAKES, PUFFS, KRISPIES, LOOPS READY TO EAT | Processed Food Products | Other Corn Products | IMPORT | Mexico | Non-Detect | 1 | 0 |
| 1022390 | 2017 | 8/17/2017 | OATS, WHOLE GRAIN | Grains and Grain Products | Oats | IMPORT | Canada | Positive | 1 | 17.4 |
| 1014997 | 2017 | 8/18/2017 | RYE FLOUR | Grains and Grain Products | Rye | DOMESTIC | United States | Non-Detect | 12 | 0 |
| 945276 | 2017 | 8/18/2017 | WHEAT BRAN (HUMAN USE) | Grains and Grain Products | Wheat | DOMESTIC | United States | Non-Detect | 1 | 0 |
| 1022716 | 2017 | 8/22/2017 | OATS, WHOLE GRAIN | Grains and Grain Products | Oats | IMPORT | Canada | Non-Detect | 12 | 0 |
| 961542 | 2017 | 8/22/2017 | KIDNEY BEAN, DRIED OR PASTE | Beans and Legumes | Other Beans and Legumes | DOMESTIC | United States | Non-Detect | 0.233 | 0 |
| 1022428 | 2017 | 8/24/2017 | RAISINS, DRIED OR PASTE | Fruit | Raisins | DOMESTIC | United States | Non-Detect | 12 | 0 |
| 997781 | 2017 | 8/24/2017 | COFFEE, BEANS | Non-Juice Beverages | Coffee | IMPORT | Ethiopia | Non-Detect | 9.225 | 0 |
| 1021197 | 2017 | 8/24/2017 | CORN FLAKES, PUFFS, KRISPIES, LOOPS READY TO EAT | Processed Food Products | Other Corn Products | DOMESTIC | United States | Non-Detect | 1 | 0 |
| 1021198 | 2017 | 8/24/2017 | CORN FLAKES, PUFFS, KRISPIES, LOOPS READY TO EAT | Processed Food Products | Other Corn Products | DOMESTIC | United States | Non-Detect | 1 | 0 |
| 1023096 | 2017 | 8/24/2017 | OTHER BAKERY PRODUCTS, N.E.C. | Processed Food Products | Other Bakery Products | IMPORT | Tunisia | Non-Detect | 1 | 0 |
| 802012 | 2017 | 8/28/2017 | BARLEY, WHOLE GRAIN | Grains and Grain Products | Barley | DOMESTIC | United States | Non-Detect | 0.233 | 0 |
| 1023235 | 2017 | 8/28/2017 | SPAGHETTI | Processed Food Products | Pasta | DOMESTIC | United States | Non-Detect | 1 | 0 |
| 1023236 | 2017 | 8/28/2017 | WHEAT FLOUR, WHOLE | Grains and Grain Products | Wheat | DOMESTIC | United States | Positive | 1 | 9 |
| 1005077 | 2017 | 8/28/2017 | RAISINS, DRIED OR PASTE | Fruit | Raisins | DOMESTIC | United States | Trace | 1 | |
| 1023477 | 2017 | 8/30/2017 | MALT, BARLEY | Grains and Grain Products | Barley | IMPORT | United Kingdom | Non-Detect | 0.233 | 0 |
| 985927 | 2017 | 8/31/2017 | CORN, WHOLE GRAIN | Grains and Grain Products | Corn | DOMESTIC | United States | Non-Detect | 1 | 0 |
| 985929 | 2017 | 8/31/2017 | CORN, WHOLE GRAIN | Grains and Grain Products | Corn | DOMESTIC | United States | Non-Detect | 1 | 0 |
| 971885 | 2017 | 9/6/2017 | BARLEY, WHOLE GRAIN | Grains and Grain Products | Barley | DOMESTIC | Unknown | Non-Detect | 0.233 | 0 |
| 973481 | 2017 | 9/6/2017 | MALT, BARLEY | Grains and Grain Products | Barley | DOMESTIC | United States | Non-Detect | 0.233 | 0 |
| 944208 | 2017 | 9/7/2017 | COFFEE, BEANS | Non-Juice Beverages | Coffee | DOMESTIC | United States | Non-Detect | 2.5 | 0 |
| 1021199 | 2017 | 9/7/2017 | CORN, SELF-RISING MEAL | Grains and Grain Products | Corn | DOMESTIC | United States | Non-Detect | 0.233 | 0 |
| 1012446 | 2017 | 9/12/2017 | OAT CEREAL (BABY) | Baby Food Products | Baby Cereals | DOMESTIC | United States | Non-Detect | 2.4 | 0 |
| 965877 | 2017 | 9/14/2017 | RYE FLOUR | Grains and Grain Products | Rye | DOMESTIC | United States | Non-Detect | 1 | 0 |
| 1025082 | 2017 | 9/18/2017 | WHEAT FLOUR, N.E.C. | Grains and Grain Products | Wheat | IMPORT | Serbia | Non-Detect | 2.4 | 0 |
| 1025123 | 2017 | 9/20/2017 | RAISINS, DRIED OR PASTE | Fruit | Raisins | IMPORT | China | Non-Detect | 2.629 | 0 |
| 968267 | 2017 | 9/26/2017 | PINTO BEAN, DRIED OR PASTE | Beans and Legumes | Pinto Beans | DOMESTIC | United States | Trace | 4.99 | |
| 1021203 | 2017 | 9/29/2017 | CORN, ENRICHED MEAL | Grains and Grain Products | Corn | DOMESTIC | United States | Non-Detect | 0.233 | 0 |

## FY2018 Data

| Sample Number | Fiscal Year | Collection Date | Product Name | Food Category | Food Subcategory | Origin Type | Country of Origin | Non-Detect, Trace, or Positive (>LOQ) | LOQ (ppb) | Amount found (ppb) |
|---|---|---|---|---|---|---|---|---|---|---|
| 1025872 | 2018 | 9/27/2017 | COFFEE, BEANS | Non-Juice Beverages | Coffee | IMPORT | Colombia | Non-Detect | 9.225 | 0 |
| 1026145 | 2018 | 10/2/2017 | MALT, BARLEY | Grains and Grain Products | Barley | IMPORT | Germany | Non-Detect | 2.4 | 0 |
| 1026479 | 2018 | 10/3/2017 | BUCKWHEAT FLOUR | Grains and Grain Products | Buckwheat | IMPORT | Russia | Non-Detect | 1 | 0 |
| 1027012 | 2018 | 10/10/2017 | COFFEE, BEANS | Non-Juice Beverages | Coffee | IMPORT | Guatemala | Non-Detect | 12.15 | 0 |
| 1027265 | 2018 | 10/11/2017 | COFFEE, BEANS | Non-Juice Beverages | Coffee | IMPORT | Guatemala | Non-Detect | 2.4 | 0 |
| 1027186 | 2018 | 10/12/2017 | COFFEE, BEANS | Non-Juice Beverages | Coffee | IMPORT | Rwanda | Non-Detect | 1 | 0 |
| 986257 | 2018 | 10/12/2017 | CORN, WHOLE GRAIN | Grains and Grain Products | Corn | DOMESTIC | United States | Non-Detect | 1 | 0 |
| 1027385 | 2018 | 10/16/2017 | WHEAT, WHOLE GRAIN | Grains and Grain Products | Wheat | IMPORT | Peru | Non-Detect | 2.4 | 0 |
| 1020336 | 2018 | 10/17/2017 | POPCORN (NOT POPPED), BULK, WHOLE GRAIN | Grains and Grain Products | Corn | DOMESTIC | United States | Non-Detect | 12 | 0 |
| 1024087 | 2018 | 10/17/2017 | WHEAT, WHOLE GRAIN | Grains and Grain Products | Wheat | DOMESTIC | United States | Non-Detect | 1 | 0 |
| 1020337 | 2018 | 10/18/2017 | POPCORN (NOT POPPED), BULK, WHOLE GRAIN | Grains and Grain Products | Corn | DOMESTIC | United States | Non-Detect | 12 | 0 |
| 1027648 | 2018 | 10/19/2017 | OATS, WHOLE GRAIN | Grains and Grain Products | Oats | DOMESTIC | United States | Non-Detect | 2.4 | 0 |
| 1027649 | 2018 | 10/19/2017 | BARLEY, WHOLE GRAIN | Grains and Grain Products | Barley | DOMESTIC | United States | Non-Detect | 2.4 | 0 |
| 1028173 | 2018 | 10/19/2017 | GRANOLA BARS | Processed Food Products | Other Processed Foods | IMPORT | Canada | Non-Detect | 2.4 | 0 |
| 1028189 | 2018 | 10/19/2017 | GRANOLA BARS | Processed Food Products | Other Processed Foods | IMPORT | Canada | Non-Detect | 2.4 | 0 |
| 1028246 | 2018 | 10/20/2017 | NAVY (WHITE) BEANS, (BAKED BEANS, PORK AND BEANS) | Beans and Legumes | Other Beans and Legumes | DOMESTIC | United States | Non-Detect | 0.233 | 0 |
| 1028106 | 2018 | 10/23/2017 | OAT FLOUR | Grains and Grain Products | Oats | IMPORT | Guatemala | Non-Detect | 2.4 | 0 |
| 1028402 | 2018 | 10/23/2017 | LUMPIA WRAPPERS (INCLUDES PASTRY WRAPPERS AND SHEETS) | Processed Food Products | Other Bakery Products | IMPORT | Mexico | Non-Detect | 1 | 0 |
| 1028378 | 2018 | 10/24/2017 | FLOURS AND MEALS N.E.C. | Grains and Grain Products | Other Grains | IMPORT | India | Non-Detect | 2.4 | 0 |
| 1028301 | 2018 | 10/25/2017 | BUCKWHEAT FLOUR | Grains and Grain Products | Buckwheat | DOMESTIC | United States | Non-Detect | 0.233 | 0 |
| 1028647 | 2018 | 10/25/2017 | COFFEE, BEANS | Non-Juice Beverages | Coffee | IMPORT | Colombia | Non-Detect | 12.15 | 0 |
| 1028655 | 2018 | 10/26/2017 | PIGEON PEAS, DRIED OR PASTE | Beans and Legumes | Peas | IMPORT | Burma | Non-Detect | 0.233 | 0 |
| 1027652 | 2018 | 10/26/2017 | BARLEY, WHOLE GRAIN | Grains and Grain Products | Barley | DOMESTIC | United States | Non-Detect | 1 | 0 |
| 1028959 | 2018 | 10/27/2017 | KIDNEY BEAN | Beans and Legumes | Other Beans and Legumes | IMPORT | China | Non-Detect | 1 | 0 |
| 1019946 | 2018 | 10/30/2017 | RICE CEREAL (BABY) | Baby Food Products | Baby Cereals | DOMESTIC | United States | Non-Detect | 12 | 0 |
| 1019947 | 2018 | 10/30/2017 | WHEAT CEREAL (BABY) | Baby Food Products | Baby Cereals | DOMESTIC | United States | Non-Detect | 12 | 0 |
| 1029194 | 2018 | 10/31/2017 | OAT FLAKES, ROLLED OATS, PUFFS, KRISPIES, LOOPS READY TO EAT | Processed Food Products | Breakfast Foods | IMPORT | Ecuador | Non-Detect | 2.4 | 0 |
| 1029197 | 2018 | 10/31/2017 | OAT FLAKES, ROLLED OATS, PUFFS, KRISPIES, LOOPS READY TO EAT | Processed Food Products | Breakfast Foods | IMPORT | Ecuador | Non-Detect | 2.4 | 0 |
| 1029213 | 2018 | 10/31/2017 | BARLEY, WHOLE GRAIN | Grains and Grain Products | Barley | IMPORT | Ecuador | Non-Detect | 2.4 | 0 |
| 1017102 | 2018 | 11/1/2017 | BUCKWHEAT FLOUR | Grains and Grain Products | Buckwheat | DOMESTIC | Unknown | Non-Detect | 0.233 | 0 |
| 995294 | 2018 | 11/2/2017 | BEAN, DRIED OR PASTE, N.E.C. (VEGETABLE) | Beans and Legumes | Other Beans and Legumes | DOMESTIC | United States | Non-Detect | 1 | 0 |
| 1029873 | 2018 | 11/3/2017 | LENTILS | Beans and Legumes | Lentils | IMPORT | Canada | Non-Detect | 1 | 0 |
| 1030430 | 2018 | 11/6/2017 | DOW SEE CHINESE FERMENTED BLACK BEANS | Beans and Legumes | Black Beans | IMPORT | China | Positive | 2.4 | 4.35 |
| 1025831 | 2018 | 11/7/2017 | WHEAT FLOUR, ENRICHED (ALL PURPOSE) | Grains and Grain Products | Wheat | DOMESTIC | United States | Non-Detect | 1 | 0 |
| 1030571 | 2018 | 11/8/2017 | CORN CHIPS, FRIED | Processed Food Products | Other Corn Products | IMPORT | Mexico | Non-Detect | 1 | 0 |
| 978249 | 2018 | 11/8/2017 | SPAGHETTI | Processed Food Products | Pasta | DOMESTIC | United States | Trace | 1 | |
| 1020338 | 2018 | 11/14/2017 | OAT FLOUR | Grains and Grain Products | Oats | DOMESTIC | United States | Non-Detect | 12 | 0 |
| 1031191 | 2018 | 11/14/2017 | WHEAT, MILLED (CRUSHED, COARSE GROUND OR CRACKED) | Grains and Grain Products | Wheat | IMPORT | Turkey | Non-Detect | 1 | 0 |
| 1031173 | 2018 | 11/15/2017 | BUCKWHEAT FLOUR | Grains and Grain Products | Buckwheat | DOMESTIC | United States | Non-Detect | 2.4 | 0 |
| 977754 | 2018 | 11/16/2017 | BEAN, DRIED OR PASTE, N.E.C. (VEGETABLE) | Beans and Legumes | Other Beans and Legumes | DOMESTIC | United States | Non-Detect | 0.233 | 0 |
| 1031571 | 2018 | 11/16/2017 | WHEAT, MILLED (CRUSHED, COARSE GROUND OR CRACKED) | Grains and Grain Products | Wheat | IMPORT | Turkey | Trace | 1 | |
| 1004783 | 2018 | 11/20/2017 | WHOLE WHEAT BREAD/ROLLS/BUNS | Processed Food Products | Bread | DOMESTIC | United States | Non-Detect | 1 | 0 |
| 1028570 | 2018 | 11/21/2017 | BLACK BEAN, DRIED OR PASTE | Beans and Legumes | Black Beans | DOMESTIC | United States | Non-Detect | 0.233 | 0 |
| 1028571 | 2018 | 11/21/2017 | NAVY (WHITE) BEANS (BAKED BEANS, PORK AND BEANS), DRIED OR PASTE | Beans and Legumes | Other Beans and Legumes | DOMESTIC | United States | Non-Detect | 0.233 | 0 |
| 1031960 | 2018 | 11/21/2017 | BEANS, N.E.C. (VEGETABLE) | Beans and Legumes | Other Beans and Legumes | IMPORT | Mexico | Non-Detect | 0.233 | 0 |
| 1032284 | 2018 | 11/27/2017 | COFFEE, BEANS | Non-Juice Beverages | Coffee | IMPORT | Peru | Non-Detect | 1 | 0 |
| 1032293 | 2018 | 11/27/2017 | CORN CHIPS, FRIED | Processed Food Products | Other Corn Products | IMPORT | Mexico | Non-Detect | 1 | 0 |
| 1032695 | 2018 | 11/28/2017 | COFFEE, BEANS | Non-Juice Beverages | Coffee | IMPORT | Guatemala | Non-Detect | 2.4 | 0 |
| 1032485 | 2018 | 11/28/2017 | FARRO | Grains and Grain Products | Wheat | IMPORT | Canada | Non-Detect | 0.97 | |
| 1024697 | 2018 | 11/29/2017 | BUCKWHEAT, WHOLE GRAIN | Grains and Grain Products | Buckwheat | DOMESTIC | United States | Non-Detect | 2.4 | 0 |
| 1028821 | 2018 | 11/29/2017 | CORN FLAKES, PUFFS, KRISPIES, LOOPS READY TO EAT | Processed Food Products | Other Corn Products | DOMESTIC | United States | Non-Detect | 1 | 0 |
| 1032889 | 2018 | 11/30/2017 | COFFEE, BEANS | Non-Juice Beverages | Coffee | IMPORT | Brazil | Non-Detect | 1 | 0 |
| 1032915 | 2018 | 11/30/2017 | COFFEE, BEANS | Non-Juice Beverages | Coffee | IMPORT | Indonesia | Non-Detect | 1 | 0 |
| 1032930 | 2018 | 12/1/2017 | MALT, BARLEY | Grains and Grain Products | Barley | IMPORT | Canada | Trace | 4.99 | |
| 1033100 | 2018 | 12/1/2017 | COFFEE, GROUND | Non-Juice Beverages | Coffee | IMPORT | Mexico | Trace | 5 | |
| 1028822 | 2018 | 12/4/2017 | BUCKWHEAT, WHOLE GRAIN | Grains and Grain Products | Buckwheat | DOMESTIC | United States | Non-Detect | 1 | 0 |
| 1033411 | 2018 | 12/5/2017 | CAPSICUMS (CAYENNE CHILI, HOT PEPPERS), GROUND, CRACKED (SPICE) | Spices | Capsicums, including Paprika | IMPORT | Mexico | Non-Detect | 0.233 | 0 |
| 1033612 | 2018 | 12/5/2017 | COFFEE, BEANS | Non-Juice Beverages | Coffee | IMPORT | Switzerland | Non-Detect | 1 | 0 |
| 1023896 | 2018 | 12/5/2017 | RAISINS, DRIED OR PASTE | Fruit | Raisins | DOMESTIC | United States | Trace | 1 | |
| 1026308 | 2018 | 12/6/2017 | CORN GRITS, BREWERS ENRICHED WHITE OR YELLOW CORN GRITS, CORN MEAL MUSH, QUICK OR INSTANT COOKING | Processed Food Products | Other Corn Products | DOMESTIC | Unknown | Non-Detect | 1 | 0 |
| 1033557 | 2018 | 12/6/2017 | MALT, BARLEY | Grains and Grain Products | Barley | IMPORT | Canada | Non-Detect | 1 | 0 |
| 1033566 | 2018 | 12/6/2017 | COFFEE, BEANS | Non-Juice Beverages | Coffee | IMPORT | Brazil | Non-Detect | 1 | 0 |
| 1033734 | 2018 | 12/7/2017 | OATMEAL, REGULAR, FRUIT OR SPICE ADDED, QUICK OR INSTANT COOKING | Processed Food Products | Breakfast Foods | IMPORT | United Kingdom | Non-Detect | 2.4 | 0 |
| 1033979 | 2018 | 12/8/2017 | COFFEE, BEANS | Non-Juice Beverages | Coffee | IMPORT | Nicaragua | Non-Detect | 2.4 | 0 |
| 1025933 | 2018 | 12/11/2017 | RAISINS (DRIED GRAPES) (BERRY) | Fruit | Raisins | DOMESTIC | United States | Non-Detect | 2.4 | 0 |
| 1016196 | 2018 | 12/11/2017 | RYE FLOUR | Grains and Grain Products | Rye | DOMESTIC | United States | Trace | 5 | |
| 1034511 | 2018 | 12/13/2017 | OATS, WHOLE GRAIN | Grains and Grain Products | Oats | IMPORT | Ireland | Non-Detect | 1 | 0 |
| 1033372 | 2018 | 12/14/2017 | CEREAL, N.E.C. (BABY) | Baby Food Products | Baby Cereals | DOMESTIC | United States | Non-Detect | 2.4 | 0 |
| 1034981 | 2018 | 12/19/2017 | RICE CEREAL (BABY) | Baby Food Products | Baby Cereals | IMPORT | Mexico | Non-Detect | 0.233 | 0 |
| 1035096 | 2018 | 12/19/2017 | BEANS, N.E.C. (VEGETABLE) | Beans and Legumes | Other Beans and Legumes | DOMESTIC | United States | Non-Detect | 0.233 | 0 |
| 1034835 | 2018 | 12/19/2017 | RAISINS, DRIED OR PASTE | Fruit | Raisins | DOMESTIC | United States | Trace | 5 | |
| 1032562 | 2018 | 12/21/2017 | RAISINS, DRIED OR PASTE | Fruit | Raisins | DOMESTIC | United States | Non-Detect | 2.4 | 0 |
| 1035816 | 2018 | 12/27/2017 | COFFEE, BEANS | Non-Juice Beverages | Coffee | IMPORT | Colombia | Non-Detect | 12.15 | 0 |
| 1035724 | 2018 | 12/28/2017 | WHEAT FLOUR, BROMATED | Grains and Grain Products | Wheat | IMPORT | Canada | Trace | 1 | |
| 1035967 | 2018 | 1/2/2018 | COFFEE, BEANS | Non-Juice Beverages | Coffee | IMPORT | Peru | Non-Detect | 12.15 | 0 |
| 1036018 | 2018 | 1/3/2018 | WHEAT FLOUR, WHOLE | Grains and Grain Products | Wheat | IMPORT | United Arab Emirates | Non-Detect | 0.233 | 0 |
| 1036294 | 2018 | 1/5/2018 | COFFEE, BEANS | Non-Juice Beverages | Coffee | IMPORT | India | Non-Detect | 1 | 0 |
| 1036328 | 2018 | 1/5/2018 | TORTILLAS | Processed Food Products | Other Corn Products | IMPORT | Mexico | Non-Detect | 1 | 0 |
| 1005975 | 2018 | 1/8/2018 | SOY BASE FORMULA PRODUCT, POWDER FORMULA | Baby Food Products | Baby Formula | DOMESTIC | United States | Non-Detect | 2.4 | 0 |
| 1030749 | 2018 | 1/9/2018 | SOY BASE FORMULA PRODUCT, POWDER FORMULA | Baby Food Products | Baby Formula | DOMESTIC | United States | Non-Detect | 2.4 | 0 |
| 1036739 | 2018 | 1/9/2018 | BEANS, CORN, AND PEA, N.E.C. (VEGETABLE) | Beans and Legumes | Other Beans and Legumes | IMPORT | Nicaragua | Non-Detect | 1 | 0 |
| 1037003 | 2018 | 1/10/2018 | COFFEE, BEANS | Non-Juice Beverages | Coffee | IMPORT | Colombia | Non-Detect | 1 | 0 |
| 941789 | 2018 | 1/10/2018 | CORN, ENRICHED MEAL | Grains and Grain Products | Corn | DOMESTIC | United States | Non-Detect | 1 | 0 |
| 970329 | 2018 | 1/10/2018 | RICE CEREAL (BABY) | Baby Food Products | Baby Cereals | DOMESTIC | United States | Non-Detect | 1 | 0 |
| 1031334 | 2018 | 1/11/2018 | PINTO BEAN | Beans and Legumes | Pinto Beans | DOMESTIC | United States | Non-Detect | 1 | 0 |
| 1032868 | 2018 | 1/11/2018 | CORN, PLAIN MEAL | Grains and Grain Products | Corn | DOMESTIC | United States | Non-Detect | 1 | 0 |
| 1025934 | 2018 | 1/11/2018 | RAISINS (DRIED GRAPES) (BERRY) | Fruit | Raisins | DOMESTIC | United States | Positive | 4.99 | 8.58 |
| 998859 | 2018 | 1/17/2018 | BUCKWHEAT, WHOLE GRAIN | Grains and Grain Products | Buckwheat | DOMESTIC | United States | Non-Detect | 0.233 | 0 |
| 1037496 | 2018 | 1/17/2018 | COFFEE, BEANS | Non-Juice Beverages | Coffee | IMPORT | Burundi | Non-Detect | 1 | 0 |
| 1037782 | 2018 | 1/19/2018 | COFFEE, BEANS | Non-Juice Beverages | Coffee | IMPORT | Ethiopia | Non-Detect | 1 | 0 |
| 1037677 | 2018 | 1/19/2018 | KIDNEY BEAN, DRIED OR PASTE | Beans and Legumes | Other Beans and Legumes | DOMESTIC | United States | Trace | 4.99 | |
| 1038317 | 2018 | 1/23/2018 | OATS, WHOLE GRAIN | Grains and Grain Products | Oats | IMPORT | Russia | Non-Detect | 1 | 0 |

| | | | | | | | | | | |
|---|---|---|---|---|---|---|---|---|---|---|
| 1038325 | 2018 | 1/23/2018 | BARLEY, WHOLE GRAIN | Grains and Grain Products | Barley | IMPORT | Russia | Non-Detect | 1 | 0 |
| 1038116 | 2018 | 1/24/2018 | WHEAT CEREAL (BABY) | Baby Food Products | Baby Cereals | DOMESTIC | United States | Non-Detect | 2.4 | 0 |
| 1038502 | 2018 | 1/24/2018 | COFFEE, BEANS | Non-Juice Beverages | Coffee | IMPORT | Colombia | Non-Detect | 2.4 | 0 |
| 1026402 | 2018 | 1/24/2018 | OATS, WHOLE GRAIN | Grains and Grain Products | Oats | DOMESTIC | Unknown | Non-Detect | 1 | 0 |
| 1038208 | 2018 | 1/24/2018 | CORN, BOLTED MEAL OR FLOUR | Grains and Grain Products | Corn | DOMESTIC | United States | Non-Detect | 1 | 0 |
| 1029939 | 2018 | 1/25/2018 | WHEAT FLOUR, WHOLE | Grains and Grain Products | Wheat | DOMESTIC | United States | Non-Detect | 2.4 | 0 |
| 1038546 | 2018 | 1/25/2018 | COFFEE, BEANS | Non-Juice Beverages | Coffee | IMPORT | Indonesia | Non-Detect | 1 | 0 |
| 1038604 | 2018 | 1/25/2018 | WHEAT FLOUR, WHOLE | Grains and Grain Products | Wheat | IMPORT | India | Non-Detect | 1 | 0 |
| 1038623 | 2018 | 1/26/2018 | COFFEE, BEANS | Non-Juice Beverages | Coffee | IMPORT | Guatemala | Non-Detect | 12.15 | 0 |
| 1038669 | 2018 | 1/26/2018 | COFFEE, BEANS | Non-Juice Beverages | Coffee | IMPORT | Ecuador | Non-Detect | 12.15 | 0 |
| 1038957 | 2018 | 1/30/2018 | FAVA BEAN | Beans and Legumes | Other Beans and Legumes | IMPORT | Mexico | Non-Detect | 2.4 | 0 |
| 1039023 | 2018 | 1/30/2018 | BUCKWHEAT, WHOLE GRAIN | Grains and Grain Products | Buckwheat | IMPORT | China | Non-Detect | 2.4 | 0 |
| 1039149 | 2018 | 1/30/2018 | COFFEE, BEANS | Non-Juice Beverages | Coffee | IMPORT | Brazil | Non-Detect | 1 | 0 |
| 1039271 | 2018 | 1/30/2018 | WHEAT FLOUR, WHOLE | Grains and Grain Products | Wheat | IMPORT | India | Non-Detect | 1 | 0 |
| 1039511 | 2018 | 1/31/2018 | RAISINS,  DRIED OR PASTE | Fruit | Raisins | IMPORT | Iran | Non-Detect | 2.4 | 0 |
| 991171 | 2018 | 1/31/2018 | WHEAT FLOUR, BLEACHED | Grains and Grain Products | Wheat | DOMESTIC | United States | Non-Detect | 2.4 | 0 |
| 1026213 | 2018 | 1/31/2018 | RICE CEREAL (BABY) | Baby Food Products | Baby Cereals | DOMESTIC | United States | Non-Detect | 1 | 0 |
| 1039678 | 2018 | 2/1/2018 | OATMEAL, REGULAR, FRUIT OR SPICE ADDED, QUICK OR INSTANT COOKING | Processed Food Products | Breakfast Foods | IMPORT | Canada | Non-Detect | 2.4 | 0 |
| 1039829 | 2018 | 2/1/2018 | TURMERIC, GROUND, CRACKED (SPICE) | Spices | Turmeric | IMPORT | India | Non-Detect | 0.233 | 0 |
| 1039688 | 2018 | 2/1/2018 | GARBANZO BEAN | Beans and Legumes | Garbanzo Beans | IMPORT | India | Non-Detect | 1 | 0 |
| 1039731 | 2018 | 2/2/2018 | GINGER, GROUND, CRACKED  (SPICE) | Spices | Ginger | IMPORT | India | Positive | 4.99 | 8.1 |
| 1039919 | 2018 | 2/4/2018 | RYE FLOUR | Grains and Grain Products | Rye | IMPORT | Canada | Non-Detect | 2.4 | 0 |
| 1040122 | 2018 | 2/5/2018 | WHEAT FLOUR, DURUM | Grains and Grain Products | Wheat | IMPORT | Italy | Non-Detect | 1 | 0 |
| 1040126 | 2018 | 2/5/2018 | MALT, BARLEY | Grains and Grain Products | Barley | IMPORT | Canada | Non-Detect | 1 | 0 |
| 1040116 | 2018 | 2/5/2018 | CORN, BOLTED MEAL OR FLOUR | Grains and Grain Products | Corn | IMPORT | Italy | Trace | 1 | |
| 1025488 | 2018 | 2/6/2018 | WHEAT FLOUR, N.E.C. | Grains and Grain Products | Wheat | DOMESTIC | United States | Non-Detect | 0.233 | 0 |
| 1040125 | 2018 | 2/6/2018 | CREAM WHEAT, QUICK OR INSTANT COOKING | Processed Food Products | Breakfast Foods | IMPORT | Canada | Non-Detect | 1 | 0 |
| 1040871 | 2018 | 2/6/2018 | LENTILS | Beans and Legumes | Lentils | IMPORT | Turkey | Non-Detect | 1 | 0 |
| 1040259 | 2018 | 2/7/2018 | NOODLES AND NOODLE PRODUCTS, N.E.C. | Processed Food Products | Pasta | IMPORT | Lebanon | Non-Detect | 2.4 | 0 |
| 1040460 | 2018 | 2/8/2018 | FARRO | Grains and Grain Products | Wheat | IMPORT | Canada | Non-Detect | 2.4 | 0 |
| 1040592 | 2018 | 2/9/2018 | SEMOLINA, WHEAT | Grains and Grain Products | Wheat | DOMESTIC | United States | Non-Detect | 1 | 0 |
| 1040593 | 2018 | 2/9/2018 | WHEAT GERM | Grains and Grain Products | Wheat | DOMESTIC | United States | Non-Detect | 1 | 0 |
| 1040594 | 2018 | 2/9/2018 | WHEAT GERM | Grains and Grain Products | Wheat | DOMESTIC | United States | Non-Detect | 1 | 0 |
| 1040596 | 2018 | 2/9/2018 | CORN, BOLTED MEAL OR FLOUR | Grains and Grain Products | Corn | DOMESTIC | United States | Non-Detect | 1 | 0 |
| 1041007 | 2018 | 2/13/2018 | OAT FLOUR | Grains and Grain Products | Oats | IMPORT | Canada | Non-Detect | 2.4 | 0 |
| 1041079 | 2018 | 2/13/2018 | BEANS, N.E.C. (VEGETABLE) | Beans and Legumes | Other Beans and Legumes | IMPORT | Mexico | Non-Detect | 1 | 0 |
| 1041333 | 2018 | 2/14/2018 | CAPSICUMS (CAYENNE CHILI, HOT PEPPERS), GROUND, CRACKED  (SPICE) | Spices | Capsicums, including Paprika | IMPORT | Mexico | Trace | 5 | |
| 1041558 | 2018 | 2/16/2018 | BUCKWHEAT, WHOLE GRAIN | Grains and Grain Products | Buckwheat | IMPORT | Russia | Non-Detect | 0.233 | 0 |
| 1041655 | 2018 | 2/16/2018 | CAPSICUMS (CAYENNE CHILI, HOT PEPPERS), WHOLE  (SPICE) | Spices | Capsicums, including Paprika | IMPORT | India | Positive | 4.99 | 5.9 |
| 1041739 | 2018 | 2/20/2018 | BEANS, N.E.C. (VEGETABLE) | Beans and Legumes | Other Beans and Legumes | IMPORT | Nicaragua | Non-Detect | 2.4 | 0 |
| 1042177 | 2018 | 2/21/2018 | OATS, WHOLE GRAIN | Grains and Grain Products | Oats | IMPORT | Russia | Non-Detect | 1 | 0 |
| 1042285 | 2018 | 2/22/2018 | COFFEE, BEANS | Non-Juice Beverages | Coffee | IMPORT | Colombia | Non-Detect | 2.4 | 0 |
| 1042327 | 2018 | 2/22/2018 | BLACKEYED PEA | Beans and Legumes | Blackeye Peas | IMPORT | Madagascar | Non-Detect | 2.4 | 0 |
| 1042523 | 2018 | 2/22/2018 | COFFEE, BEANS | Non-Juice Beverages | Coffee | IMPORT | Colombia | Non-Detect | 2.4 | 0 |
| 951137 | 2018 | 2/22/2018 | WHEAT FLOUR, BLEACHED | Grains and Grain Products | Wheat | DOMESTIC | Unknown | Non-Detect | 2.4 | 0 |
| 1027948 | 2018 | 2/22/2018 | WHEAT, WHOLE GRAIN | Grains and Grain Products | Wheat | DOMESTIC | United States | Non-Detect | 1 | 0 |
| 1042098 | 2018 | 2/22/2018 | COFFEE, BEANS | Non-Juice Beverages | Coffee | IMPORT | Brazil | Non-Detect | 1 | 0 |
| 1042164 | 2018 | 2/22/2018 | CORN GRITS, BREWERS ENRICHED WHITE OR YELLOW CORN GRITS, CORN MEAL MUSH, QUICK OR INSTANT COOKING | Processed Food Products | Other Corn Products | IMPORT | China | Non-Detect | 1 | 0 |
| 1044025 | 2018 | 2/22/2018 | WHEAT CEREAL (BABY) | Baby Food Products | Baby Cereals | IMPORT | Egypt | Non-Detect | 1 | 0 |
| 1042359 | 2018 | 2/23/2018 | COFFEE, BEANS | Non-Juice Beverages | Coffee | IMPORT | Guatemala | Non-Detect | 2.4 | 0 |
| 1024209 | 2018 | 2/27/2018 | BUCKWHEAT, WHOLE GRAIN | Grains and Grain Products | Buckwheat | DOMESTIC | United States | Non-Detect | 2.4 | 0 |
| 1042590 | 2018 | 2/27/2018 | GRAM FLOUR | Beans and Legumes | Garbanzo Beans | IMPORT | Canada | Non-Detect | 2.4 | 0 |
| 1042609 | 2018 | 2/27/2018 | FOODS WITH SUPPLEMENTAL NUTRIENTS ADDED, WITH OR WITHOUT ARTIFICAL SWEETNERS | Other | Other | IMPORT | Mexico | Non-Detect | 2.4 | 0 |
| 1042798 | 2018 | 2/27/2018 | RAISINS (DRIED GRAPES)  (BERRY) | Fruit | Raisins | IMPORT | South Africa | Trace | 9.97 | |
| 1044022 | 2018 | 2/28/2018 | WHEAT FLOUR, DURUM | Grains and Grain Products | Wheat | IMPORT | Canada | Non-Detect | 1 | 0 |
| 1044023 | 2018 | 2/28/2018 | WHEAT FLOUR, DURUM | Grains and Grain Products | Wheat | IMPORT | Canada | Non-Detect | 1 | 0 |
| 1013836 | 2018 | 3/5/2018 | OAT FLOUR | Grains and Grain Products | Oats | DOMESTIC | United States | Non-Detect | 1 | 0 |
| 1044047 | 2018 | 3/5/2018 | CORN, PLAIN MEAL | Grains and Grain Products | Corn | IMPORT | United States | Non-Detect | 1 | 0 |
| 1044054 | 2018 | 3/5/2018 | WHEAT, WHOLE GRAIN | Grains and Grain Products | Wheat | IMPORT | Canada | Non-Detect | 1 | 0 |
| 1030970 | 2018 | 3/6/2018 | KIDNEY BEAN, DRIED OR PASTE | Beans and Legumes | Other Beans and Legumes | DOMESTIC | United States | Non-Detect | 2.4 | 0 |
| 1044352 | 2018 | 3/6/2018 | COFFEE, BEANS | Non-Juice Beverages | Coffee | IMPORT | Brazil | Non-Detect | 2.4 | 0 |
| 1044270 | 2018 | 3/6/2018 | BUCKWHEAT, WHOLE GRAIN | Grains and Grain Products | Buckwheat | IMPORT | China | Non-Detect | 1 | 0 |
| 1044698 | 2018 | 3/7/2018 | OATS, WHOLE GRAIN | Grains and Grain Products | Oats | IMPORT | Canada | Non-Detect | 2.4 | 0 |
| 973880 | 2018 | 3/13/2018 | BEANS, N.E.C. (VEGETABLE) | Beans and Legumes | Other Beans and Legumes | DOMESTIC | United States | Non-Detect | 0.233 | 0 |
| 973881 | 2018 | 3/13/2018 | PINTO BEAN | Beans and Legumes | Pinto Beans | DOMESTIC | United States | Non-Detect | 0.233 | 0 |
| 977758 | 2018 | 3/13/2018 | RYE FLOUR | Grains and Grain Products | Rye | DOMESTIC | United States | Non-Detect | 0.233 | 0 |
| 977759 | 2018 | 3/13/2018 | BUCKWHEAT FLOUR | Grains and Grain Products | Buckwheat | DOMESTIC | United States | Non-Detect | 0.233 | 0 |
| 1045260 | 2018 | 3/13/2018 | COFFEE, BEANS | Non-Juice Beverages | Coffee | IMPORT | Uganda | Positive | 5 | 9.36 |
| 1046181 | 2018 | 3/14/2018 | BEAN, CORN, PEA, DRIED OR PASTE, N.E.C. (VEGETABLE) | Beans and Legumes | Other Beans and Legumes | IMPORT | Taiwan | Non-Detect | 1 | 0 |
| 1021170 | 2018 | 3/15/2018 | WHEAT FLOUR, BLEACHED | Grains and Grain Products | Wheat | DOMESTIC | United States | Non-Detect | 1 | 0 |
| 1021171 | 2018 | 3/15/2018 | MALT, BARLEY | Grains and Grain Products | Barley | DOMESTIC | United States | Non-Detect | 1 | 0 |
| 1045702 | 2018 | 3/15/2018 | MALT, BARLEY | Grains and Grain Products | Barley | IMPORT | Germany | Non-Detect | 1 | 0 |
| 1045966 | 2018 | 3/19/2018 | MALT, BARLEY | Grains and Grain Products | Barley | IMPORT | United Kingdom | Non-Detect | 2.4 | 0 |
| 1046071 | 2018 | 3/19/2018 | COFFEE, BEANS | Non-Juice Beverages | Coffee | IMPORT | Mexico | Non-Detect | 9.225 | 0 |
| 1046546 | 2018 | 3/21/2018 | COFFEE, BEANS | Non-Juice Beverages | Coffee | IMPORT | Guatemala | Non-Detect | 2.4 | 0 |
| 1035696 | 2018 | 3/21/2018 | WHEAT FLOUR, N.E.C. | Grains and Grain Products | Wheat | DOMESTIC | United States | Non-Detect | 0.233 | 0 |
| 1046473 | 2018 | 3/21/2018 | BARLEY, WHOLE GRAIN | Grains and Grain Products | Barley | IMPORT | United Kingdom | Non-Detect | 0.233 | 0 |
| 1046477 | 2018 | 3/21/2018 | MALT, BARLEY | Grains and Grain Products | Barley | IMPORT | United Kingdom | Non-Detect | 0.233 | 0 |
| 1046409 | 2018 | 3/21/2018 | TORTILLAS | Processed Food Products | Other Corn Products | IMPORT | Mexico | Non-Detect | 1 | 0 |
| 1046859 | 2018 | 3/21/2018 | PISTACHIO, SHELLED | Nuts and Edible Seeds | Pistachios | IMPORT | United States | Non-Detect | 1 | 0 |
| 1024059 | 2018 | 3/22/2018 | RYE FLOUR | Grains and Grain Products | Rye | DOMESTIC | United States | Non-Detect | 2.4 | 0 |
| 1025493 | 2018 | 3/22/2018 | CORN GLUTEN | Grains and Grain Products | Corn | DOMESTIC | United States | Non-Detect | 0.233 | 0 |
| 1046596 | 2018 | 3/22/2018 | BEANS, N.E.C. (VEGETABLE) | Beans and Legumes | Other Beans and Legumes | IMPORT | Mexico | Non-Detect | 0.233 | 0 |
| 1038534 | 2018 | 3/27/2018 | CORN, BOLTED MEAL OR FLOUR | Grains and Grain Products | Corn | DOMESTIC | United States | Non-Detect | 1 | 0 |
| 1046951 | 2018 | 3/27/2018 | GARBANZO BEAN, DRIED OR PASTE | Beans and Legumes | Garbanzo Beans | IMPORT | Argentina | Non-Detect | 1 | 0 |
| 1047864 | 2018 | 3/28/2018 | COFFEE, BEANS | Non-Juice Beverages | Coffee | IMPORT | Colombia | Non-Detect | 2.4 | 0 |
| 1045750 | 2018 | 3/28/2018 | RYE FLOUR | Grains and Grain Products | Rye | DOMESTIC | United States | Non-Detect | 1 | 0 |
| 1045751 | 2018 | 3/28/2018 | RYE FLOUR | Grains and Grain Products | Rye | DOMESTIC | United States | Non-Detect | 1 | 0 |
| 1047621 | 2018 | 3/28/2018 | CORN CHIPS, FRIED | Processed Food Products | Other Corn Products | IMPORT | Philippines | Non-Detect | 1 | 0 |
| 1047627 | 2018 | 3/28/2018 | CORN CHIPS, FRIED | Processed Food Products | Other Corn Products | IMPORT | Philippines | Non-Detect | 1 | 0 |
| 1047490 | 2018 | 3/29/2018 | MALT, BARLEY | Grains and Grain Products | Barley | IMPORT | Belgium | Non-Detect | 2.4 | 0 |
| 996920 | 2018 | 3/29/2018 | SOY BASE FORMULA PRODUCT, POWDER FORMULA | Baby Food Products | Baby Formula | DOMESTIC | United States | Non-Detect | 1 | 0 |
| 1048695 | 2018 | 4/2/2018 | BARLEY, WHOLE GRAIN | Grains and Grain Products | Barley | IMPORT | Canada | Trace | 4.99 | |

| | | | | | | | | | | |
|---|---|---|---|---|---|---|---|---|---|---|
| 1026771 | 2018 | 4/3/2018 | BARLEY, WHOLE GRAIN | Grains and Grain Products | Barley | DOMESTIC | United States | Non-Detect | 1 | 0 |
| 1048412 | 2018 | 4/4/2018 | TURMERIC, GROUND, CRACKED (SPICE) | Spices | Turmeric | IMPORT | India | Trace | 1 | |
| 1048444 | 2018 | 4/5/2018 | BEANS, N.E.C. (VEGETABLE) | Beans and Legumes | Other Beans and Legumes | IMPORT | Mexico | Non-Detect | 0.233 | 0 |
| 1048969 | 2018 | 4/9/2018 | TURMERIC, GROUND, CRACKED (SPICE) | Spices | Turmeric | IMPORT | India | Non-Detect | 2.4 | 0 |
| 1024211 | 2018 | 4/9/2018 | WHEAT CEREAL (BABY) | Baby Food Products | Baby Cereals | DOMESTIC | United States | Non-Detect | 0.233 | 0 |
| 1020977 | 2018 | 4/10/2018 | RICE CEREAL (BABY) | Baby Food Products | Baby Cereals | DOMESTIC | United States | Non-Detect | 12 | 0 |
| 1049035 | 2018 | 4/10/2018 | BEAN, CORN, PEA, DRIED OR PASTE, N.E.C. (VEGETABLE) | Beans and Legumes | Other Beans and Legumes | IMPORT | Philippines | Non-Detect | 1 | 0 |
| 1049565 | 2018 | 4/12/2018 | CORN CHIPS, FRIED | Processed Food Products | Other Corn Products | IMPORT | Philippines | Non-Detect | 1 | 0 |
| 1049762 | 2018 | 4/13/2018 | TURMERIC, GROUND, CRACKED (SPICE) | Spices | Turmeric | IMPORT | India | Non-Detect | 0.233 | 0 |
| 1049874 | 2018 | 4/16/2018 | COFFEE, BEANS | Non-Juice Beverages | Coffee | IMPORT | Ethiopia | Non-Detect | 2.4 | 0 |
| 1024350 | 2018 | 4/17/2018 | WHEAT FLOUR, GRAHAM | Grains and Grain Products | Wheat | DOMESTIC | United States | Non-Detect | 1 | 0 |
| 1050526 | 2018 | 4/18/2018 | BLACKEYED PEA | Beans and Legumes | Blackeye Peas | IMPORT | Peru | Non-Detect | 2.4 | 0 |
| 1050398 | 2018 | 4/18/2018 | BEAN, CORN, PEA, DRIED OR PASTE, N.E.C. (VEGETABLE) | Beans and Legumes | Other Beans and Legumes | IMPORT | Mexico | Non-Detect | 0.233 | 0 |
| 1050412 | 2018 | 4/18/2018 | GARBANZO BEAN, DRIED OR PASTE | Beans and Legumes | Garbanzo Beans | IMPORT | Mexico | Non-Detect | 0.233 | 0 |
| 1050510 | 2018 | 4/18/2018 | WHEAT, WHOLE GRAIN | Grains and Grain Products | Wheat | IMPORT | Canada | Non-Detect | 0.233 | 0 |
| 1039636 | 2018 | 4/19/2018 | RICE, CULTIVATED, WHOLE GRAIN | Grains and Grain Products | Rice | DOMESTIC | United States | Non-Detect | 1 | 0 |
| 1050684 | 2018 | 4/20/2018 | WHEAT FLOUR, ENRICHED (ALL PURPOSE) | Grains and Grain Products | Wheat | IMPORT | Peru | Non-Detect | 2.4 | 0 |
| 1050686 | 2018 | 4/20/2018 | GINGER, GROUND, CRACKED (SPICE) | Spices | Ginger | IMPORT | India | Non-Detect | 1 | 0 |
| 1050788 | 2018 | 4/22/2018 | COFFEE, BEANS | Non-Juice Beverages | Coffee | IMPORT | Brazil | Non-Detect | 1 | 0 |
| 1050852 | 2018 | 4/23/2018 | OAT FLOUR | Grains and Grain Products | Oats | IMPORT | Canada | Non-Detect | 1 | 0 |
| 1036232 | 2018 | 4/24/2018 | RICE CEREAL (BABY) | Baby Food Products | Baby Cereals | DOMESTIC | United States | Non-Detect | 2.4 | 0 |
| 1051037 | 2018 | 4/24/2018 | KIDNEY BEAN | Beans and Legumes | Other Beans and Legumes | IMPORT | Nicaragua | Non-Detect | 0.233 | 0 |
| 1049226 | 2018 | 4/24/2018 | RYE FLOUR | Grains and Grain Products | Rye | DOMESTIC | United States | Positive | 5 | 5.01 |
| 1051587 | 2018 | 4/25/2018 | LENTILS | Beans and Legumes | Lentils | IMPORT | Canada | Non-Detect | 1 | 0 |
| 1052172 | 2018 | 5/1/2018 | COFFEE, BEANS | Non-Juice Beverages | Coffee | IMPORT | Honduras | Non-Detect | 12.15 | 0 |
| 1024581 | 2018 | 5/1/2018 | WHEAT FLOUR, WHOLE | Grains and Grain Products | Wheat | DOMESTIC | United States | Trace | 5 | |
| 1022331 | 2018 | 5/3/2018 | MALT, BARLEY | Grains and Grain Products | Barley | DOMESTIC | United States | Non-Detect | 12 | 0 |
| 1029003 | 2018 | 5/3/2018 | WHEAT, WHOLE GRAIN | Grains and Grain Products | Wheat | DOMESTIC | United States | Non-Detect | 0.233 | 0 |
| 1038396 | 2018 | 5/7/2018 | BEANS, N.E.C. (VEGETABLE) | Beans and Legumes | Other Beans and Legumes | DOMESTIC | United States | Non-Detect | 2.4 | 0 |
| 1054958 | 2018 | 5/7/2018 | COFFEE, BEANS | Non-Juice Beverages | Coffee | IMPORT | Brazil | Non-Detect | 1 | 0 |
| 1054988 | 2018 | 5/7/2018 | COFFEE, BEANS | Non-Juice Beverages | Coffee | IMPORT | Colombia | Non-Detect | 1 | 0 |
| 1050807 | 2018 | 5/8/2018 | GRAPE JUICE, BERRY JUICE OR CONCENTRATES | Fruit | Other Juices | DOMESTIC | Unknown | Non-Detect | 1 | 0 |
| 1050808 | 2018 | 5/8/2018 | GRAPE JUICE, BERRY JUICE OR CONCENTRATES | Fruit | Other Juices | DOMESTIC | Unknown | Non-Detect | 1 | 0 |
| 1050809 | 2018 | 5/8/2018 | GRAPE JUICE, BERRY JUICE OR CONCENTRATES | Fruit | Other Juices | DOMESTIC | Unknown | Non-Detect | 1 | 0 |
| 1050810 | 2018 | 5/8/2018 | GRAPE JUICE, BERRY JUICE OR CONCENTRATES | Fruit | Other Juices | DOMESTIC | United States | Non-Detect | 1 | 0 |
| 1054940 | 2018 | 5/8/2018 | COFFEE, BEANS | Non-Juice Beverages | Coffee | IMPORT | Guatemala | Non-Detect | 1 | 0 |
| 1035441 | 2018 | 5/9/2018 | RAISINS (DRIED GRAPES) (BERRY) | Fruit | Raisins | DOMESTIC | United States | Non-Detect | 1 | 0 |
| 1053626 | 2018 | 5/9/2018 | GRAPE JUICE, BERRY JUICE OR CONCENTRATES | Fruit | Other Juices | DOMESTIC | United States | Non-Detect | 1 | 0 |
| 1053627 | 2018 | 5/9/2018 | GRAPE JUICE, BERRY JUICE OR CONCENTRATES | Fruit | Other Juices | DOMESTIC | Unknown | Non-Detect | 1 | 0 |
| 1054949 | 2018 | 5/9/2018 | GINGER, GROUND, CRACKED (SPICE) | Spices | Ginger | DOMESTIC | United States | Non-Detect | 1 | 0 |
| 1044670 | 2018 | 5/9/2018 | CAPSICUMS (CAYENNE CHILI, HOT PEPPERS), GROUND, CRACKED (SPICE) | Spices | Capsicums, including Paprika | DOMESTIC | United States | Positive | 4.99 | 7.35 |
| 1044669 | 2018 | 5/9/2018 | CAPSICUMS (CAYENNE CHILI, HOT PEPPERS), WHOLE (SPICE) | Spices | Capsicums, including Paprika | DOMESTIC | United States | Trace | 5 | |
| 1058697 | 2018 | 5/10/2018 | OATS, WHOLE GRAIN | Grains and Grain Products | Oats | IMPORT | Ireland | Non-Detect | 1 | 0 |
| 1058700 | 2018 | 5/10/2018 | OATS, WHOLE GRAIN | Grains and Grain Products | Oats | IMPORT | Ireland | Non-Detect | 1 | 0 |
| 1058693 | 2018 | 5/10/2018 | OATS, WHOLE GRAIN | Grains and Grain Products | Oats | IMPORT | Ireland | Trace | 1 | |
| 1054950 | 2018 | 5/16/2018 | MALT, BARLEY | Grains and Grain Products | Barley | DOMESTIC | United States | Non-Detect | 0.233 | 0 |
| 1061549 | 2018 | 5/17/2018 | COFFEE, BEANS | Non-Juice Beverages | Coffee | IMPORT | Costa Rica | Non-Detect | 12.15 | 0 |
| 1061902 | 2018 | 5/18/2018 | CORN, PLAIN MEAL | Grains and Grain Products | Corn | IMPORT | Colombia | Non-Detect | 1 | 0 |
| 1006177 | 2018 | 5/24/2018 | RICE CEREAL (BABY) | Baby Food Products | Baby Cereals | DOMESTIC | United States | Non-Detect | 1 | 0 |
| 1065703 | 2018 | 5/24/2018 | KIDNEY BEAN | Beans and Legumes | Other Beans and Legumes | IMPORT | Canada | Non-Detect | 1 | 0 |
| 1065705 | 2018 | 5/24/2018 | KIDNEY BEAN | Beans and Legumes | Other Beans and Legumes | IMPORT | Canada | Non-Detect | 1 | 0 |
| 1065707 | 2018 | 5/24/2018 | CRANBERRY BEAN | Beans and Legumes | Other Beans and Legumes | IMPORT | Canada | Non-Detect | 1 | 0 |
| 1046777 | 2018 | 5/30/2018 | BUCKWHEAT FLOUR | Grains and Grain Products | Buckwheat | DOMESTIC | United States | Non-Detect | 0.233 | 0 |
| 1046778 | 2018 | 5/30/2018 | BUCKWHEAT FLOUR | Grains and Grain Products | Buckwheat | DOMESTIC | United States | Trace | 4.99 | |
| 1038028 | 2018 | 6/4/2018 | OAT FLOUR | Grains and Grain Products | Oats | DOMESTIC | United States | Non-Detect | 0.233 | 0 |
| 1038029 | 2018 | 6/4/2018 | BUCKWHEAT FLOUR | Grains and Grain Products | Buckwheat | DOMESTIC | United States | Non-Detect | 0.233 | 0 |
| 1068251 | 2018 | 6/4/2018 | CORN, PLAIN MEAL | Grains and Grain Products | Corn | DOMESTIC | United States | Non-Detect | 1 | 0 |
| 1069808 | 2018 | 6/4/2018 | CORN FLAKES, PUFFS, KRISPIES, LOOPS READY TO EAT | Processed Food Products | Other Corn Products | DOMESTIC | United States | Non-Detect | 1.48 | 0 |
| 1070655 | 2018 | 6/7/2018 | OAT FLOUR | Grains and Grain Products | Oats | IMPORT | Canada | Non-Detect | 1.53 | 0 |
| 1071023 | 2018 | 6/8/2018 | BEAN, CORN, PEA, DRIED OR PASTE, N.E.C. (VEGETABLE) | Beans and Legumes | Other Beans and Legumes | IMPORT | Mexico | Non-Detect | 0.233 | 0 |
| 1018097 | 2018 | 6/18/2018 | BLACK EYE BEANS | Beans and Legumes | Blackeye Peas | DOMESTIC | United States | Non-Detect | 0.233 | 0 |
| 1072894 | 2018 | 6/19/2018 | BARLEY, WHOLE GRAIN | Grains and Grain Products | Barley | IMPORT | Ethiopia | Non-Detect | 1 | 0 |
| 1009167 | 2018 | 6/19/2018 | GINGER, GROUND, CRACKED (SPICE) | Spices | Ginger | DOMESTIC | United States | Trace | 5 | |
| 1067221 | 2018 | 6/19/2018 | OATS, WHOLE GRAIN | Grains and Grain Products | Oats | DOMESTIC | United States | Trace | 5 | |
| 1072869 | 2018 | 6/20/2018 | BUCKWHEAT FLOUR | Grains and Grain Products | Buckwheat | DOMESTIC | United States | Non-Detect | 0.233 | 0 |
| 1073250 | 2018 | 6/20/2018 | COFFEE, BEANS | Non-Juice Beverages | Coffee | IMPORT | Tanzania, United Republic Of | Non-Detect | 1 | 0 |
| 1073619 | 2018 | 6/25/2018 | OATMEAL, REGULAR, FRUIT OR SPICE ADDED, QUICK OR INSTANT COOKING | Processed Food Products | Breakfast Foods | IMPORT | Ireland | Non-Detect | 1 | 0 |
| 1073505 | 2018 | 6/26/2018 | OAT FLAKES, ROLLED OATS, PUFFS, KRISPIES, LOOPS READY TO EAT | Processed Food Products | Breakfast Foods | IMPORT | Canada | Non-Detect | 1 | 0 |
| 1073884 | 2018 | 6/27/2018 | COFFEE, BEANS | Non-Juice Beverages | Coffee | IMPORT | Colombia | Non-Detect | 1 | 0 |
| 1074451 | 2018 | 7/2/2018 | COFFEE, GROUND | Non-Juice Beverages | Coffee | IMPORT | India | Trace | 9.225 | |
| 1074286 | 2018 | 7/9/2018 | RYE FLOUR | Grains and Grain Products | Rye | DOMESTIC | United States | Non-Detect | 0.233 | 0 |
| 1074963 | 2018 | 7/9/2018 | WHEAT, MILLED PRODUCT, N.E.C. | Grains and Grain Products | Wheat | IMPORT | Iran | Non-Detect | 0.233 | 0 |
| 1074956 | 2018 | 7/10/2018 | COFFEE, BEANS | Non-Juice Beverages | Coffee | IMPORT | El Salvador | Non-Detect | 12.15 | 0 |
| 1075032 | 2018 | 7/10/2018 | SPAGHETTI | Processed Food Products | Pasta | IMPORT | China | Non-Detect | 1 | 0 |
| 1075096 | 2018 | 7/11/2018 | RAISINS (DRIED GRAPES) (BERRY) | Fruit | Raisins | IMPORT | South Africa | Non-Detect | 2.4 | 0 |
| 1045727 | 2018 | 7/11/2018 | PINTO BEAN | Beans and Legumes | Pinto Beans | DOMESTIC | United States | Non-Detect | 0.233 | 0 |
| 1075215 | 2018 | 7/11/2018 | AZUFRADO BEAN | Beans and Legumes | Other Beans and Legumes | IMPORT | Mexico | Non-Detect | 1 | 0 |
| 1075291 | 2018 | 7/12/2018 | SPAGHETTI | Processed Food Products | Pasta | IMPORT | Italy | Non-Detect | 1 | 0 |
| 1075295 | 2018 | 7/12/2018 | PRETZELS, BAKED | Processed Food Products | Other Snack Foods | IMPORT | Austria | Non-Detect | 1 | 0 |
| 1075297 | 2018 | 7/12/2018 | COFFEE, BEANS | Non-Juice Beverages | Coffee | IMPORT | Colombia | Non-Detect | 1 | 0 |
| 1075401 | 2018 | 7/13/2018 | COFFEE, BEANS | Non-Juice Beverages | Coffee | IMPORT | Ethiopia | Non-Detect | 1 | 0 |
| 1075607 | 2018 | 7/13/2018 | RAISINS (DRIED GRAPES) (BERRY) | Fruit | Raisins | IMPORT | Argentina | Positive | 2.629 | 49.2 |
| 1072593 | 2018 | 7/16/2018 | MALT, BARLEY | Grains and Grain Products | Barley | DOMESTIC | United States | Non-Detect | 1 | 0 |
| 1075929 | 2018 | 7/19/2018 | MIXED CEREAL FLAKES, SHREDDED AND OTHER FORMS READY TO EAT | Processed Food Products | Breakfast Foods | IMPORT | Mexico | Non-Detect | 0.233 | 0 |
| 1076422 | 2018 | 7/23/2018 | BARLEY, WHOLE GRAIN | Grains and Grain Products | Barley | IMPORT | Russia | Non-Detect | 1 | 0 |
| 1030187 | 2018 | 7/24/2018 | OAT FLOUR | Grains and Grain Products | Oats | DOMESTIC | United States | Non-Detect | 0.233 | 0 |
| 1072861 | 2018 | 7/25/2018 | RAISINS, DRIED OR PASTE | Fruit | Raisins | DOMESTIC | United States | Trace | 2.629 | |
| 1076743 | 2018 | 7/26/2018 | COFFEE, BEANS | Non-Juice Beverages | Coffee | IMPORT | Costa Rica | Non-Detect | 12 | 0 |
| 1076770 | 2018 | 7/26/2018 | COFFEE, BEANS | Non-Juice Beverages | Coffee | IMPORT | Brazil | Non-Detect | 12 | 0 |
| 1030188 | 2018 | 7/29/2018 | OAT FLOUR | Grains and Grain Products | Oats | DOMESTIC | United States | Non-Detect | 2.4 | 0 |
| 1040948 | 2018 | 8/2/2018 | MALT, BARLEY | Grains and Grain Products | Barley | DOMESTIC | United States | Non-Detect | 2.4 | 0 |
| 1077821 | 2018 | 8/6/2018 | RAISINS, DRIED OR PASTE | Fruit | Raisins | IMPORT | Chile | Non-Detect | 2.4 | 0 |
| 1037708 | 2018 | 8/6/2018 | SOY BASE FORMULA PRODUCT, POWDER FORMULA | Baby Food Products | Baby Formula | DOMESTIC | United States | Non-Detect | 1 | 0 |

| | | | | | | | | | | | |
|---|---|---|---|---|---|---|---|---|---|---|---|
| 1078098 | 2018 | 8/8/2018 | BARLEY, WHOLE GRAIN | Grains and Grain Products | Barley | IMPORT | Canada | Positive | 1 | 6.77 |
| 1078276 | 2018 | 8/9/2018 | RYE, WHOLE GRAIN | Grains and Grain Products | Rye | IMPORT | Canada | Non-Detect | 0.233 | 0 |
| 1078812 | 2018 | 8/10/2018 | WHEAT, WHOLE GRAIN | Grains and Grain Products | Wheat | IMPORT | Canada | Non-Detect | 2.4 | 0 |
| 1078807 | 2018 | 8/13/2018 | RYE, WHOLE GRAIN | Grains and Grain Products | Rye | IMPORT | Canada | Non-Detect | 2.4 | 0 |
| 1078488 | 2018 | 8/13/2018 | RAISINS, DRIED OR PASTE | Fruit | Raisins | IMPORT | Afghanistan | Non-Detect | 1 | 0 |
| 1079028 | 2018 | 8/15/2018 | WHEAT, WHOLE GRAIN | Grains and Grain Products | Wheat | IMPORT | Canada | Non-Detect | 2.4 | 0 |
| 1079105 | 2018 | 8/16/2018 | COFFEE, BEANS | Non-Juice Beverages | Coffee | IMPORT | Ethiopia | Non-Detect | 12.15 | 0 |
| 1038876 | 2018 | 8/16/2018 | OATS, WHOLE GRAIN | Grains and Grain Products | Oats | DOMESTIC | United States | Positive | 1 | 7.1 |
| 1079766 | 2018 | 8/22/2018 | OATMEAL, REGULAR, FRUIT OR SPICE ADDED, QUICK OR INSTANT COOKING | Processed Food Products | Breakfast Foods | IMPORT | Canada | Non-Detect | 2.4 | 0 |
| 1079786 | 2018 | 8/22/2018 | WHEAT, WHOLE GRAIN | Grains and Grain Products | Wheat | IMPORT | Canada | Positive | 5 | 6.29 |
| 1080080 | 2018 | 8/23/2018 | BARLEY, WHOLE GRAIN | Grains and Grain Products | Barley | IMPORT | Canada | Non-Detect | 1 | 0 |
| 1073763 | 2018 | 8/27/2018 | RAISINS, DRIED OR PASTE | Fruit | Raisins | IMPORT | Uzbekistan | Positive | 1 | 5.2 |
| 1080801 | 2018 | 8/28/2018 | NUTMEG, GROUND, CRACKED (SPICE) | Spices | Nutmeg | IMPORT | India | Positive | 1 | 52.89 |
| 1071798 | 2018 | 8/28/2018 | SOY BASE FORMULA PRODUCT, POWDER FORMULA | Baby Food Products | Baby Formula | DOMESTIC | United States | Non-Detect | 2.4 | 0 |
| 1080838 | 2018 | 8/28/2018 | NUTMEG, WHOLE (SPICE) | Spices | Nutmeg | IMPORT | India | Positive | 1 | 4.68 |
| 1039237 | 2018 | 8/28/2018 | WHEAT FLOUR, WHOLE | Grains and Grain Products | Wheat | DOMESTIC | United States | Trace | 1 | |
| 1080495 | 2018 | 8/29/2018 | BUCKWHEAT FLOUR | Grains and Grain Products | Buckwheat | DOMESTIC | United States | Non-Detect | 0.233 | 0 |
| 973709 | 2018 | 8/30/2018 | GINGER, GROUND, CRACKED (SPICE) | Spices | Ginger | IMPORT | India | Non-Detect | 0.233 | 0 |
| 1019692 | 2018 | 9/5/2018 | SOY BASE FORMULA PRODUCT, READY TO FEED | Baby Food Products | Baby Formula | DOMESTIC | United States | Non-Detect | 0.233 | 0 |
| 1082259 | 2018 | 9/5/2018 | CAPSICUMS (CAYENNE CHILI, HOT PEPPERS), GROUND, CRACKED (SPICE) | Spices | Capsicums, including Paprika | IMPORT | Mexico | Non-Detect | 0.233 | 0 |
| 1078764 | 2018 | 9/6/2018 | OATS, WHOLE GRAIN | Grains and Grain Products | Oats | DOMESTIC | United States | Non-Detect | 2.5 | 0 |
| 1078855 | 2018 | 9/6/2018 | MALT, BARLEY | Grains and Grain Products | Barley | DOMESTIC | United States | Non-Detect | 1 | 0 |
| 1081068 | 2018 | 9/6/2018 | OATS, WHOLE GRAIN | Grains and Grain Products | Oats | IMPORT | United States | Non-Detect | 1 | 0 |
| 1081069 | 2018 | 9/6/2018 | OATS, WHOLE GRAIN | Grains and Grain Products | Oats | IMPORT | United States | Non-Detect | 1 | 0 |
| 1081070 | 2018 | 9/6/2018 | OATS, WHOLE GRAIN | Grains and Grain Products | Oats | IMPORT | United States | Non-Detect | 1 | 0 |
| 1082583 | 2018 | 9/7/2018 | BARLEY, WHOLE GRAIN | Grains and Grain Products | Barley | IMPORT | Russia | Non-Detect | 1 | 0 |
| 1082853 | 2018 | 9/10/2018 | SOYBEANS (EDIBLE) | Beans and Legumes | Soybeans | DOMESTIC | United States | Non-Detect | 2.5 | 0 |
| 1082854 | 2018 | 9/10/2018 | SOYBEANS (EDIBLE) | Beans and Legumes | Soybeans | DOMESTIC | United States | Non-Detect | 2.5 | 0 |
| 1083143 | 2018 | 9/11/2018 | OAT FLOUR | Grains and Grain Products | Oats | IMPORT | Canada | Non-Detect | 1 | 0 |
| 1083357 | 2018 | 9/12/2018 | MALT, BARLEY | Grains and Grain Products | Barley | IMPORT | Germany | Non-Detect | 1 | 0 |
| 1083614 | 2018 | 9/14/2018 | TURMERIC, WHOLE (SPICE) | Spices | Turmeric | IMPORT | India | Non-Detect | 0.233 | 0 |
| 974747 | 2018 | 9/18/2018 | BARLEY, WHOLE GRAIN | Grains and Grain Products | Barley | IMPORT | United States | Non-Detect | 0.233 | 0 |
| 1051837 | 2018 | 9/18/2018 | BARLEY, WHOLE GRAIN | Grains and Grain Products | Barley | IMPORT | Italy | Non-Detect | 0.233 | 0 |
| 974746 | 2018 | 9/18/2018 | BARLEY, WHOLE GRAIN | Grains and Grain Products | Barley | DOMESTIC | United States | Trace | 4.99 | |
| 1085039 | 2018 | 9/21/2018 | BARLEY, WHOLE GRAIN | Grains and Grain Products | Barley | IMPORT | Russia | Non-Detect | 1 | 0 |
| 1044059 | 2018 | 9/24/2018 | WHEAT FLOUR, WHOLE | Grains and Grain Products | Wheat | DOMESTIC | United States | Non-Detect | 1 | 0 |
| 1077639 | 2018 | 9/24/2018 | WHEAT FLOUR, WHOLE | Grains and Grain Products | Wheat | DOMESTIC | United States | Trace | 1 | |
| 1023572 | 2018 | 9/25/2018 | CORN, ENRICHED MEAL | Grains and Grain Products | Corn | DOMESTIC | United States | Non-Detect | 0.233 | 0 |
| 1023574 | 2018 | 9/25/2018 | OAT FLOUR | Grains and Grain Products | Oats | DOMESTIC | United States | Non-Detect | 0.233 | 0 |
| 1085210 | 2018 | 9/25/2018 | BARLEY, WHOLE GRAIN | Grains and Grain Products | Barley | IMPORT | United States | Non-Detect | 1 | 0 |
| 1026242 | 2018 | 9/26/2018 | WHEAT FLOUR, WHOLE | Grains and Grain Products | Wheat | DOMESTIC | United States | Non-Detect | 0.233 | 0 |
| 904526 | 2018 | 9/27/2018 | CORN, HOMINY GRITS | Grains and Grain Products | Corn | DOMESTIC | United States | Non-Detect | 1 | 0 |

## FY2019 Data

| Sample Number | Fiscal Year | Collection Date | Product Name | Food Category | Food Subcategory | Origin Type | Country of Origin | Non-Detect, Trace, or Positive (>LOQ) | LOQ (ppb) | Amount found (ppb) |
|---|---|---|---|---|---|---|---|---|---|---|
| 1086601 | 2019 | 10/5/2018 | MALT WHEAT | Grains and Grain Products | Wheat | IMPORT | Netherlands | Non-Detect | 0.233 | 0 |
| 1087128 | 2019 | 10/11/2018 | BUCKWHEAT WHOLE GRAIN | Grains and Grain Products | Buckwheat | IMPORT | United States | Trace | 1 | |
| 1087828 | 2019 | 10/17/2018 | COFFEE BEANS | Non-Juice Beverages | Coffee | IMPORT | El Salvador | Non-Detect | 2.5 | 0 |
| 1088030 | 2019 | 10/18/2018 | RAISINS DRIED OR PASTE | Fruit | Raisins | IMPORT | Chile | Non-Detect | 1 | 0 |
| 1088117 | 2019 | 10/19/2018 | COFFEE BEANS | Non-Juice Beverages | Coffee | IMPORT | Honduras | Non-Detect | 1 | 0 |
| 1030191 | 2019 | 10/23/2018 | WHEAT WHOLE GRAIN | Grains and Grain Products | Wheat | DOMESTIC | United States | Non-Detect | 0.233 | 0 |
| 1088589 | 2019 | 10/24/2018 | RAISINS DRIED OR PASTE | Fruit | Raisins | DOMESTIC | United States | Trace | 2.629 | |
| 1088925 | 2019 | 10/25/2018 | CORN CHIPS FRIED | Processed Food Products | Other Corn Products | IMPORT | Mexico | Non-Detect | 1 | 0 |
| 1089138 | 2019 | 10/29/2018 | COFFEE BEANS | Non-Juice Beverages | Coffee | IMPORT | Ecuador | Non-Detect | 12 | 0 |
| 1089410 | 2019 | 10/30/2018 | PISTACHIO SHELLED | Nuts and Edible Seeds | Pistachios | IMPORT | Afghanistan | Non-Detect | 1 | 0 |
| 1089599 | 2019 | 10/30/2018 | MUNG BEAN | Beans and Legumes | Other Beans and Legumes | IMPORT | Australia | Non-Detect | 1 | 0 |
| 1077545 | 2019 | 10/31/2018 | RAISINS DRIED OR PASTE | Fruit | Raisins | DOMESTIC | United States | Non-Detect | 1 | 0 |
| 1039242 | 2019 | 11/1/2018 | BEANS NEC VEGETABLE | Beans and Legumes | Other Beans and Legumes | DOMESTIC | United States | Non-Detect | 0.233 | 0 |
| 1081507 | 2019 | 11/1/2018 | FIG DRIED OR PASTE | Fruit | Dried Figs | DOMESTIC | United States | Non-Detect | 1 | 0 |
| 1090420 | 2019 | 11/6/2018 | BREAKFAST FOODS READY TO EAT NEC | Processed Food Products | Breakfast Foods | IMPORT | Canada | Non-Detect | 1 | 0 |
| 1090718 | 2019 | 11/7/2018 | GARBANZO BEAN | Beans and Legumes | Garbanzo Beans | IMPORT | Afghanistan | Non-Detect | 2.4 | 0 |
| 1090587 | 2019 | 11/8/2018 | WHEAT FLAKES PUFFS KRISPIES LOOPS SHREDDED WHEAT GERM READY TO EAT | Processed Food Products | Breakfast Foods | IMPORT | Canada | Non-Detect | 2.4 | 0 |
| 1080577 | 2019 | 11/8/2018 | WHEAT WHOLE GRAIN | Grains and Grain Products | Wheat | DOMESTIC | United States | Non-Detect | 0.233 | 0 |
| 1090373 | 2019 | 11/8/2018 | CORN BOLTED MEAL OR FLOUR | Grains and Grain Products | Corn | DOMESTIC | United States | Non-Detect | 1 | 0 |
| 919533 | 2019 | 11/9/2018 | WHEAT FLOUR WHOLE | Grains and Grain Products | Wheat | DOMESTIC | United States | Non-Detect | 0.233 | 0 |
| 1090786 | 2019 | 11/9/2018 | MALT BARLEY | Grains and Grain Products | Barley | IMPORT | Germany | Non-Detect | 1 | 0 |
| 1091179 | 2019 | 11/13/2018 | BARLEY WHOLE GRAIN | Grains and Grain Products | Barley | IMPORT | Canada | Non-Detect | 2.4 | 0 |
| 1090933 | 2019 | 11/13/2018 | MIXED CEREAL QUICK OR INSTANT COOKING | Processed Food Products | Breakfast Foods | IMPORT | Canada | Non-Detect | 1 | 0 |
| 1091183 | 2019 | 11/14/2018 | COFFEE BEANS | Non-Juice Beverages | Coffee | IMPORT | Colombia | Non-Detect | 12.15 | 0 |
| 1091256 | 2019 | 11/14/2018 | WHOLE WHEAT BREAD ROLLS BUNS | Processed Food Products | Bread | IMPORT | Canada | Trace | 0.233 | |
| 1091444 | 2019 | 11/15/2018 | OAT CEREAL BABY | Baby Food Products | Baby Cereals | IMPORT | Canada | Non-Detect | 0.233 | 0 |
| 1089201 | 2019 | 11/19/2018 | BEANS NEC VEGETABLE | Beans and Legumes | Other Beans and Legumes | DOMESTIC | United States | Non-Detect | 1 | 0 |
| 1089204 | 2019 | 11/19/2018 | SPAGHETTI | Processed Food Products | Pasta | DOMESTIC | United States | Non-Detect | 1 | 0 |
| 1091805 | 2019 | 11/20/2018 | WHEAT FLOUR BLEACHED | Grains and Grain Products | Wheat | DOMESTIC | United States | Non-Detect | 2.5 | 0 |
| 1091765 | 2019 | 11/20/2018 | KIDNEY BEAN | Beans and Legumes | Other Beans and Legumes | IMPORT | Nicaragua | Non-Detect | 1 | 0 |
| 1091884 | 2019 | 11/21/2018 | COFFEE BEANS | Non-Juice Beverages | Coffee | IMPORT | Italy | Non-Detect | 2.5 | 0 |
| 1092446 | 2019 | 11/24/2018 | RAISINS DRIED GRAPES BERRY | Fruit | Raisins | IMPORT | Afghanistan | Non-Detect | 2.629 | 0 |
| 1092451 | 2019 | 11/24/2018 | RAISINS DRIED GRAPES BERRY | Fruit | Raisins | IMPORT | Afghanistan | Non-Detect | 2.629 | 0 |
| 1092485 | 2019 | 11/24/2018 | RAISINS DRIED GRAPES BERRY | Fruit | Raisins | IMPORT | Afghanistan | Non-Detect | 2.629 | 0 |
| 1092490 | 2019 | 11/24/2018 | RAISINS DRIED GRAPES BERRY | Fruit | Raisins | IMPORT | Afghanistan | Non-Detect | 2.629 | 0 |
| 1092395 | 2019 | 11/27/2018 | COFFEE BEANS | Non-Juice Beverages | Coffee | IMPORT | Honduras | Non-Detect | 12.15 | 0 |
| 1078152 | 2019 | 11/28/2018 | WHEAT FLOUR DURUM | Grains and Grain Products | Wheat | DOMESTIC | United States | Non-Detect | 0.233 | 0 |
| 1092518 | 2019 | 11/28/2018 | COFFEE BEANS | Non-Juice Beverages | Coffee | IMPORT | Brazil | Non-Detect | 12.15 | 0 |
| 1092877 | 2019 | 11/28/2018 | RAISINS DRIED OR PASTE | Fruit | Raisins | IMPORT | Afghanistan | Non-Detect | 1 | 0 |
| 1092819 | 2019 | 11/29/2018 | COFFEE BEANS | Non-Juice Beverages | Coffee | IMPORT | Ethiopia | Non-Detect | 1 | 0 |
| 1092923 | 2019 | 11/29/2018 | OAT FLAKES ROLLED OATS PUFFS KRISPIES LOOPS READY TO EAT | Processed Food Products | Breakfast Foods | IMPORT | United States | Non-Detect | 1 | 0 |
| 1078155 | 2019 | 11/30/2018 | WHEAT FLOUR ENRICHED BROMATED | Grains and Grain Products | Wheat | DOMESTIC | United States | Non-Detect | 0.233 | 0 |
| 1093037 | 2019 | 12/2/2018 | GARBANZO BEAN | Beans and Legumes | Garbanzo Beans | IMPORT | Canada | Non-Detect | 1 | 0 |
| 1093314 | 2019 | 12/4/2018 | COFFEE BEANS | Non-Juice Beverages | Coffee | IMPORT | Colombia | Non-Detect | 2.5 | 0 |
| 1093265 | 2019 | 12/4/2018 | MALT BARLEY | Grains and Grain Products | Barley | IMPORT | Canada | Non-Detect | 1 | 0 |
| 1093277 | 2019 | 12/4/2018 | COCOA BEAN NEC | Candy and Chocolate Products | Other Chocolate Products | IMPORT | Mexico | Non-Detect | 1 | 0 |
| 1093581 | 2019 | 12/7/2018 | OATMEAL REGULAR FRUIT OR SPICE ADDED QUICK OR INSTANT COOKING | Processed Food Products | Breakfast Foods | IMPORT | Ireland | Non-Detect | 0.233 | 0 |
| 1093596 | 2019 | 12/7/2018 | COFFEE GROUND | Non-Juice Beverages | Coffee | IMPORT | Italy | Non-Detect | 9.225 | 0 |
| 1093615 | 2019 | 12/7/2018 | COFFEE BEANS | Non-Juice Beverages | Coffee | IMPORT | Papua New Guinea | Non-Detect | 1 | 0 |
| 1094158 | 2019 | 12/12/2018 | MALT BARLEY | Grains and Grain Products | Barley | IMPORT | Canada | Non-Detect | 2.5 | 0 |
| 1092210 | 2019 | 12/12/2018 | CORN HOMINY GRITS | Grains and Grain Products | Corn | DOMESTIC | United States | Non-Detect | 1 | 0 |
| 1094024 | 2019 | 12/12/2018 | WHEAT FLOUR ENRICHED ALL PURPOSE | Grains and Grain Products | Wheat | IMPORT | Canada | Non-Detect | 1 | 0 |
| 1094121 | 2019 | 12/12/2018 | BARLEY WHOLE GRAIN | Grains and Grain Products | Barley | IMPORT | Canada | Non-Detect | 1 | 0 |
| 1094075 | 2019 | 12/12/2018 | RAISINS DRIED OR PASTE | Fruit | Raisins | DOMESTIC | United States | Trace | 5 | |
| 1094219 | 2019 | 12/13/2018 | COFFEE BEANS | Non-Juice Beverages | Coffee | IMPORT | Brazil | Non-Detect | 2.5 | 0 |
| 1094319 | 2019 | 12/13/2018 | MALT BARLEY | Grains and Grain Products | Barley | IMPORT | Canada | Non-Detect | 2.5 | 0 |
| 1094352 | 2019 | 12/13/2018 | RAISINS DRIED OR PASTE | Fruit | Raisins | IMPORT | China | Non-Detect | 2.5 | 0 |
| 919535 | 2019 | 12/14/2018 | WHEAT FLOUR WHOLE | Grains and Grain Products | Wheat | DOMESTIC | United States | Non-Detect | 0.233 | 0 |
| 1094458 | 2019 | 12/15/2018 | OAT FLAKES ROLLED OATS PUFFS KRISPIES LOOPS READY TO EAT | Processed Food Products | Breakfast Foods | IMPORT | Canada | Non-Detect | 1 | 0 |
| 1094736 | 2019 | 12/17/2018 | CORN CHIPS FRIED | Processed Food Products | Other Corn Products | IMPORT | Philippines | Non-Detect | 1 | 0 |
| 1085433 | 2019 | 12/18/2018 | PINE NUT PINON SHELLED | Nuts and Edible Seeds | Pine Nuts | DOMESTIC | United States | Non-Detect | 2.5 | 0 |
| 1077770 | 2019 | 12/18/2018 | WHEAT FLOUR WHOLE | Grains and Grain Products | Wheat | DOMESTIC | United States | Non-Detect | 0.233 | 0 |
| 1075461 | 2019 | 12/20/2018 | CORN CEREAL BABY | Baby Food Products | Baby Cereals | DOMESTIC | United States | Non-Detect | 2.4 | 0 |
| 1085697 | 2019 | 12/20/2018 | RAISINS DRIED OR PASTE | Fruit | Raisins | DOMESTIC | United States | Non-Detect | 1 | 0 |
| 1094907 | 2019 | 12/20/2018 | BEANS NEC VEGETABLE | Beans and Legumes | Other Beans and Legumes | IMPORT | Mexico | Non-Detect | 1 | 0 |
| 1095438 | 2019 | 1/17/2019 | FLOURS AND MEALS NEC | Grains and Grain Products | Other Grains | IMPORT | Canada | Non-Detect | 2.5 | 0 |
| 1095443 | 2019 | 1/17/2019 | BARLEY WHOLE GRAIN | Grains and Grain Products | Barley | IMPORT | China | Non-Detect | 2.5 | 0 |
| 1095577 | 2019 | 1/23/2019 | MOONG DAL INDIAN DRIED HULLED SPLIT GREEN GRAMBEANS OR MUNGBEANS YELLOW AND USED IN SOUPS | Beans and Legumes | Other Beans and Legumes | IMPORT | Thailand | Non-Detect | 1 | 0 |
| 1095824 | 2019 | 1/30/2019 | BLACKEYE PEAS DRIED OR PASTE | Beans and Legumes | Blackeye Peas | IMPORT | Peru | Non-Detect | 1 | 0 |
| 1095892 | 2019 | 1/30/2019 | BEAN CORN PEA DRIED O RPASTE NEC VEGETABLE | Beans and Legumes | Other Beans and Legumes | IMPORT | Mexico | Non-Detect | 1 | 0 |
| 1095906 | 2019 | 1/30/2019 | FAVA BEAN | Beans and Legumes | Other Beans and Legumes | IMPORT | China | Non-Detect | 1 | 0 |
| 1093886 | 2019 | 1/31/2019 | SPAGHETTI | Processed Food Products | Pasta | DOMESTIC | United States | Non-Detect | 1 | 0 |
| 1095932 | 2019 | 1/31/2019 | COFFEE BEANS | Non-Juice Beverages | Coffee | IMPORT | Brazil | Non-Detect | 0.21 | 0 |
| 1095962 | 2019 | 1/31/2019 | TORTILLAS | Processed Food Products | Other Corn Products | IMPORT | Mexico | Non-Detect | 1 | 0 |
| 1095975 | 2019 | 1/31/2019 | LIMA BEAN DRIED OR PASTE | Beans and Legumes | Other Beans and Legumes | IMPORT | Peru | Non-Detect | 1 | 0 |
| 1095976 | 2019 | 1/31/2019 | FIG DRIED OR PASTE | Fruit | Dried Figs | IMPORT | Turkey | Non-Detect | 1 | 0 |
| 1072017 | 2019 | 2/4/2019 | BARLEY WHOLE GRAIN | Grains and Grain Products | Barley | DOMESTIC | United States | Non-Detect | 2.4 | 0 |
| 1096794 | 2019 | 2/5/2019 | COFFEE BEANS DECAFFEINATED | Non-Juice Beverages | Coffee | IMPORT | Brazil | Non-Detect | 2.4 | 0 |
| 1038179 | 2019 | 2/5/2019 | WHEAT MILLED PRODUCT NEC | Grains and Grain Products | Wheat | DOMESTIC | United States | Non-Detect | 0.233 | 0 |
| 1096543 | 2019 | 2/5/2019 | MALT BARLEY | Grains and Grain Products | Barley | IMPORT | Netherlands | Non-Detect | 0.233 | 0 |
| 1096348 | 2019 | 2/5/2019 | COFFEE BEANS | Non-Juice Beverages | Coffee | IMPORT | Dominican Republic (the) | Positive | 10 | 14.3 |
| 1094357 | 2019 | 2/7/2019 | BREAD ROLLS BUNS ETC NEC PREPARED DRY MIX WITH MILK OR EGG | Processed Food Products | Bread | DOMESTIC | Unknown | Non-Detect | 1 | 0 |
| 1096678 | 2019 | 2/7/2019 | OATMEAL REGULAR FRUIT OR SPICE ADDED QUICK OR INSTANT COOKING | Processed Food Products | Breakfast Foods | IMPORT | Canada | Non-Detect | 1 | 0 |
| 1096911 | 2019 | 2/7/2019 | CORN PLAIN MEAL | Grains and Grain Products | Corn | IMPORT | United States | Non-Detect | 1 | 0 |
| 1097215 | 2019 | 2/8/2019 | BEANS NEC VEGETABLE | Beans and Legumes | Other Beans and Legumes | IMPORT | Mexico | Non-Detect | 0.233 | 0 |
| 1096986 | 2019 | 2/9/2019 | LENTILS DRIED OR PASTE | Beans and Legumes | Lentils | IMPORT | Canada | Non-Detect | 2.4 | 0 |
| 1096989 | 2019 | 2/9/2019 | PEA DRIED OR PASTE | Beans and Legumes | Peas | IMPORT | Canada | Non-Detect | 2.4 | 0 |
| 1096991 | 2019 | 2/9/2019 | LENTILS DRIED OR PASTE | Beans and Legumes | Lentils | IMPORT | Canada | Non-Detect | 2.4 | 0 |

| | | | | | | | | | | |
|---|---|---|---|---|---|---|---|---|---|---|
| 1096993 | 2019 | 2/9/2019 | GARBANZO BEAN | Beans and Legumes | Garbanzo Beans | IMPORT | Australia | Non-Detect | 2.4 | 0 |
| 1096995 | 2019 | 2/9/2019 | BLACKEYED PEA | Beans and Legumes | Blackeye Peas | IMPORT | Madagascar | Non-Detect | 2.4 | 0 |
| 1097426 | 2019 | 2/11/2019 | COFFEE BEANS | Non-Juice Beverages | Coffee | IMPORT | Brazil | Non-Detect | 2.4 | 0 |
| 1097449 | 2019 | 2/13/2019 | COFFEE BEANS | Non-Juice Beverages | Coffee | IMPORT | Guatemala | Non-Detect | 2.4 | 0 |
| 1094927 | 2019 | 2/13/2019 | BUCKWHEAT WHOLE GRAIN | Grains and Grain Products | Buckwheat | DOMESTIC | United States | Non-Detect | 0.233 | 0 |
| 1097814 | 2019 | 2/14/2019 | COFFEE BEANS | Non-Juice Beverages | Coffee | IMPORT | Brazil | Non-Detect | 9.225 | 0 |
| 1097682 | 2019 | 2/15/2019 | BLACK BEAN DRIED OR PASTE | Beans and Legumes | Black Beans | IMPORT | Canada | Non-Detect | 1 | 0 |
| 1097864 | 2019 | 2/19/2019 | CORN BOLTED MEAL OR FLOUR | Grains and Grain Products | Corn | IMPORT | Guatemala | Non-Detect | 0.233 | 0 |
| 1086168 | 2019 | 2/20/2019 | RAISINS DRIED GRAPES BERRY | Fruit | Raisins | DOMESTIC | United States | Non-Detect | 2.629 | 0 |
| 1098349 | 2019 | 2/21/2019 | KIDNEY BEAN | Beans and Legumes | Other Beans and Legumes | IMPORT | Nicaragua | Non-Detect | 1 | 0 |
| 1098360 | 2019 | 2/21/2019 | KIDNEY BEAN | Beans and Legumes | Other Beans and Legumes | IMPORT | Nicaragua | Non-Detect | 1 | 0 |
| 1098639 | 2019 | 2/22/2019 | RYE FLOUR | Grains and Grain Products | Rye | IMPORT | Russia | Non-Detect | 1 | 0 |
| 1098930 | 2019 | 2/25/2019 | CHICK PEA DRIED OR PASTE | Beans and Legumes | Garbanzo Beans | IMPORT | Australia | Non-Detect | 2.4 | 0 |
| 1083798 | 2019 | 2/25/2019 | CREAM WHEAT QUICK OR INSTANT COOKING | Processed Food Products | Breakfast Foods | DOMESTIC | United States | Non-Detect | 1 | 0 |
| 1097067 | 2019 | 2/26/2019 | BUCKWHEAT FLOUR | Grains and Grain Products | Buckwheat | DOMESTIC | United States | Non-Detect | 2.4 | 0 |
| 1097857 | 2019 | 2/26/2019 | BUCKWHEAT FLOUR | Grains and Grain Products | Buckwheat | DOMESTIC | United States | Non-Detect | 2.4 | 0 |
| 1098820 | 2019 | 2/26/2019 | SEMOLINA WHEAT | Grains and Grain Products | Wheat | IMPORT | Canada | Non-Detect | 2.4 | 0 |
| 1098828 | 2019 | 2/26/2019 | COFFEE BEANS | Non-Juice Beverages | Coffee | IMPORT | Brazil | Non-Detect | 2.4 | 0 |
| 1098893 | 2019 | 2/26/2019 | COFFEE BEANS | Non-Juice Beverages | Coffee | IMPORT | Guatemala | Non-Detect | 2.4 | 0 |
| 1098933 | 2019 | 2/26/2019 | BARLEY WHOLE GRAIN | Grains and Grain Products | Barley | IMPORT | Canada | Non-Detect | 2.4 | 0 |
| 1098936 | 2019 | 2/26/2019 | WHEAT MILLED PRODUCT NEC | Grains and Grain Products | Wheat | IMPORT | United States | Non-Detect | 2.4 | 0 |
| 1098990 | 2019 | 2/27/2019 | GARBANZO BEAN | Beans and Legumes | Garbanzo Beans | IMPORT | Canada | Non-Detect | 2.4 | 0 |
| 1029931 | 2019 | 2/27/2019 | PISTACHIO IN SHELL | Nuts and Edible Seeds | Pistachios | DOMESTIC | United States | Non-Detect | 1 | 0 |
| 1099648 | 2019 | 2/28/2019 | LENTILS DRIED OR PASTE | Beans and Legumes | Lentils | IMPORT | Canada | Non-Detect | 0.233 | 0 |
| 1099750 | 2019 | 2/28/2019 | PEA DRIED OR PASTE | Beans and Legumes | Peas | IMPORT | Canada | Non-Detect | 0.233 | 0 |
| 1099759 | 2019 | 2/28/2019 | PEA DRIED OR PASTE | Beans and Legumes | Peas | IMPORT | Canada | Non-Detect | 0.233 | 0 |
| 1099200 | 2019 | 2/28/2019 | COFFEE BEANS | Non-Juice Beverages | Coffee | IMPORT | Brazil | Non-Detect | 1 | 0 |
| 1099263 | 2019 | 2/28/2019 | COFFEE BEANS | Non-Juice Beverages | Coffee | IMPORT | Indonesia | Non-Detect | 1 | 0 |
| 1099872 | 2019 | 2/28/2019 | MUNG BEAN | Beans and Legumes | Other Beans and Legumes | IMPORT | Canada | Non-Detect | 1 | 0 |
| 1099908 | 2019 | 2/28/2019 | GARBANZO BEAN | Beans and Legumes | Garbanzo Beans | IMPORT | Canada | Non-Detect | 1 | 0 |
| 1099988 | 2019 | 2/28/2019 | PIGEON PEAS | Beans and Legumes | Peas | IMPORT | Canada | Non-Detect | 1 | 0 |
| 1100001 | 2019 | 2/28/2019 | MUNG BEAN | Beans and Legumes | Other Beans and Legumes | IMPORT | Canada | Non-Detect | 1 | 0 |
| 1100008 | 2019 | 2/28/2019 | BEAN DRIED OR PASTE NEC VEGETABLE | Beans and Legumes | Other Beans and Legumes | IMPORT | Canada | Non-Detect | 1 | 0 |
| 1099635 | 2019 | 3/1/2019 | COFFEE BEANS | Non-Juice Beverages | Coffee | IMPORT | Honduras | Non-Detect | 9.225 | 0 |
| 1099438 | 2019 | 3/1/2019 | COFFEE BEANS | Non-Juice Beverages | Coffee | IMPORT | Italy | Non-Detect | 1 | 0 |
| 1099849 | 2019 | 3/5/2019 | OAT FLOUR | Grains and Grain Products | Oats | IMPORT | Canada | Non-Detect | 12 | 0 |
| 1099698 | 2019 | 3/5/2019 | COFFEE BEANS | Non-Juice Beverages | Coffee | IMPORT | Colombia | Non-Detect | 9.225 | 0 |
| 1092085 | 2019 | 3/6/2019 | FIG DRIED OR PASTE | Fruit | Dried Figs | IMPORT | Turkey | Non-Detect | 1 | 0 |
| 1092086 | 2019 | 3/6/2019 | RICE FLOUR | Grains and Grain Products | Rice | DOMESTIC | United States | Non-Detect | 1 | 0 |
| 1098519 | 2019 | 3/6/2019 | SOY BASE FORMULA PRODUCT LIQUID CONCENTRATE | Baby Food Products | Baby Formula | DOMESTIC | United States | Non-Detect | 1 | 0 |
| 1098520 | 2019 | 3/6/2019 | SOY BASE FORMULA PRODUCT LIQUID CONCENTRATE | Baby Food Products | Baby Formula | DOMESTIC | United States | Non-Detect | 1 | 0 |
| 1100012 | 2019 | 3/6/2019 | COFFEE BEANS | Non-Juice Beverages | Coffee | IMPORT | Ethiopia | Non-Detect | 1 | 0 |
| 1100011 | 2019 | 3/6/2019 | COFFEE BEANS | Non-Juice Beverages | Coffee | IMPORT | Tanzania, United Republic Of | Trace | 1 | |
| 1089743 | 2019 | 3/7/2019 | OATS WHOLE GRAIN | Grains and Grain Products | Oats | IMPORT | Canada | Non-Detect | 2.5 | 0 |
| 1068299 | 2019 | 3/7/2019 | PISTACHIO IN SHELL | Nuts and Edible Seeds | Pistachios | DOMESTIC | United States | Non-Detect | 1 | 0 |
| 1100645 | 2019 | 3/8/2019 | COFFEE BEANS | Non-Juice Beverages | Coffee | IMPORT | Nicaragua | Non-Detect | 12 | 0 |
| 1100689 | 2019 | 3/8/2019 | COFFEE BEANS | Non-Juice Beverages | Coffee | IMPORT | Guatemala | Non-Detect | 12 | 0 |
| 1100705 | 2019 | 3/8/2019 | WHEAT MEAL | Grains and Grain Products | Wheat | IMPORT | Canada | Non-Detect | 0.86 | 0 |
| 1100852 | 2019 | 3/11/2019 | WHEAT FLOUR ENRICHED ALL PURPOSE | Grains and Grain Products | Wheat | IMPORT | Mexico | Non-Detect | 0.233 | 0 |
| 1100841 | 2019 | 3/11/2019 | COFFEE BEANS | Non-Juice Beverages | Coffee | IMPORT | Guatemala | Non-Detect | 1 | 0 |
| 968201 | 2019 | 3/11/2019 | WHEAT BRAN HUMAN USE | Grains and Grain Products | Wheat | DOMESTIC | United States | Non-Detect | 1 | 0 |
| 1098857 | 2019 | 3/12/2019 | BARLEY CEREAL BABY | Baby Food Products | Baby Cereals | DOMESTIC | United States | Non-Detect | 0.233 | 0 |
| 1100910 | 2019 | 3/13/2019 | CORN WHOLE GRAIN | Grains and Grain Products | Corn | DOMESTIC | United States | Non-Detect | 1 | 0 |
| 1101144 | 2019 | 3/13/2019 | OATS WHOLE GRAIN | Grains and Grain Products | Oats | IMPORT | Canada | Positive | 1 | 6 |
| 1101778 | 2019 | 3/14/2019 | CEREAL PREPARATIONS NOT ELSEWHERE MENTIONED NEC | Processed Food Products | Breakfast Foods | IMPORT | Canada | Non-Detect | 0.233 | 0 |
| 1092277 | 2019 | 3/14/2019 | OAT CEREAL BABY | Baby Food Products | Baby Cereals | DOMESTIC | United States | Trace | 1 | |
| 1101674 | 2019 | 3/15/2019 | COFFEE BEANS | Non-Juice Beverages | Coffee | IMPORT | Honduras | Non-Detect | 1 | 0 |
| 969540 | 2019 | 3/18/2019 | RAISINS DRIED OR PASTE | Fruit | Raisins | DOMESTIC | United States | Non-Detect | 2.629 | 0 |
| 1089401 | 2019 | 3/18/2019 | CORN FLAKES PUFFS KRISPIES LOOPS READY TO EAT | Processed Food Products | Other Corn Products | DOMESTIC | United States | Non-Detect | 1 | 0 |
| 1101586 | 2019 | 3/18/2019 | RAISINS DRIED OR PASTE | Fruit | Raisins | DOMESTIC | United States | Trace | 1 | |
| 1101856 | 2019 | 3/19/2019 | COFFEE BEANS | Non-Juice Beverages | Coffee | IMPORT | Honduras | Non-Detect | 1 | 0 |
| 1051466 | 2019 | 3/20/2019 | PINTO BEAN DRIED OR PASTE | Beans and Legumes | Pinto Beans | IMPORT | Canada | Non-Detect | 0.233 | 0 |
| 1102106 | 2019 | 3/20/2019 | RAISINS DRIED GRAPES BERRY | Fruit | Raisins | IMPORT | South Africa | Non-Detect | 2.629 | 0 |
| 1080362 | 2019 | 3/20/2019 | RAISINS DRIED OR PASTE | Fruit | Raisins | DOMESTIC | United States | Positive | 5 | 12 |
| 1102261 | 2019 | 3/21/2019 | COFFEE BEANS | Non-Juice Beverages | Coffee | IMPORT | Colombia | Non-Detect | 2.4 | 0 |
| 1075507 | 2019 | 3/21/2019 | PISTACHIO IN SHELL | Nuts and Edible Seeds | Pistachios | DOMESTIC | United States | Non-Detect | 1 | 0 |
| 1100635 | 2019 | 3/25/2019 | RICE CEREAL BABY | Baby Food Products | Baby Cereals | DOMESTIC | United States | Non-Detect | 1 | 0 |
| 1100636 | 2019 | 3/25/2019 | RICE CEREAL BABY | Baby Food Products | Baby Cereals | DOMESTIC | United States | Non-Detect | 1 | 0 |
| 1103161 | 2019 | 3/26/2019 | OATS WHOLE GRAIN | Grains and Grain Products | Oats | IMPORT | Canada | Non-Detect | 0.233 | 0 |
| 1103085 | 2019 | 3/27/2019 | COCOA BEAN NEC | Candy and Chocolate Products | Other Chocolate Products | IMPORT | Ivory Coast | Trace | 5 | |
| 1097527 | 2019 | 3/28/2019 | PEPPER BLACK GROUND CRACKED SPICE | Spices | Pepper | DOMESTIC | United States | Non-Detect | 0.233 | 0 |
| 1103278 | 2019 | 3/28/2019 | PISTACHIO IN SHELL | Nuts and Edible Seeds | Pistachios | IMPORT | Turkey | Non-Detect | 1 | 0 |
| 1097526 | 2019 | 3/28/2019 | GINGER GROUND CRACKED SPICE | Spices | Ginger | DOMESTIC | United States | Trace | 5 | |
| 1103331 | 2019 | 3/29/2019 | OATMEAL REGULAR FRUIT OR SPICE ADDED QUICK OR INSTANT COOKING | Processed Food Products | Breakfast Foods | IMPORT | Canada | Non-Detect | 1 | 0 |
| 1103403 | 2019 | 3/29/2019 | WHEAT WHOLE GRAIN | Grains and Grain Products | Wheat | IMPORT | China | Non-Detect | 1 | 0 |
| 1103587 | 2019 | 4/1/2019 | LENTILS | Beans and Legumes | Lentils | IMPORT | India | Non-Detect | 0.233 | 0 |
| 1081911 | 2019 | 4/2/2019 | MALT BARLEY | Grains and Grain Products | Barley | IMPORT | Canada | Non-Detect | 0.233 | 0 |
| 1104168 | 2019 | 4/3/2019 | KIDNEY BEAN | Beans and Legumes | Other Beans and Legumes | IMPORT | China | Non-Detect | 2.4 | 0 |
| 1104151 | 2019 | 4/3/2019 | COFFEE BEANS | Non-Juice Beverages | Coffee | IMPORT | Honduras | Non-Detect | 12.15 | 0 |
| 1104871 | 2019 | 4/8/2019 | FIBER NEC | Other | Other | IMPORT | Mexico | Non-Detect | 1 | 0 |
| 1105315 | 2019 | 4/9/2019 | RYE FLOUR | Grains and Grain Products | Rye | DOMESTIC | United States | Non-Detect | 2.4 | 0 |
| 1105369 | 2019 | 4/9/2019 | RAISINS DRIED OR PASTE | Fruit | Raisins | IMPORT | Afghanistan | Non-Detect | 2.4 | 0 |
| 1105394 | 2019 | 4/9/2019 | RAISINS DRIED OR PASTE | Fruit | Raisins | IMPORT | Afghanistan | Non-Detect | 2.4 | 0 |
| 1089745 | 2019 | 4/9/2019 | OATS WHOLE GRAIN | Grains and Grain Products | Oats | IMPORT | Canada | Non-Detect | 0.233 | 0 |
| 1098546 | 2019 | 4/9/2019 | OATS WHOLE GRAIN | Grains and Grain Products | Oats | DOMESTIC | United States | Trace | 0.8 | |
| 1105448 | 2019 | 4/10/2019 | CHICORY COFFEE MIXTURES COFFEE SUBSTITUTE | Non-Juice Beverages | Coffee | IMPORT | India | Non-Detect | 1 | 0 |
| 1105484 | 2019 | 4/10/2019 | BUCKWHEAT WHOLE GRAIN | Grains and Grain Products | Buckwheat | IMPORT | China | Non-Detect | 1 | 0 |
| 1105366 | 2019 | 4/11/2019 | BARLEY WHOLE GRAIN | Grains and Grain Products | Barley | IMPORT | Canada | Non-Detect | 1 | 0 |
| 1105585 | 2019 | 4/12/2019 | OATS WHOLE GRAIN | Grains and Grain Products | Oats | IMPORT | Canada | Non-Detect | 1 | 0 |

| | | | | | | | | | | |
|---|---|---|---|---|---|---|---|---|---|---|
| 1093892 | 2019 | 4/15/2019 | CORN PLAIN MEAL | Grains and Grain Products | Corn | DOMESTIC | United States | Non-Detect | 1 | 0 |
| 1093893 | 2019 | 4/15/2019 | CHAMPION BEAN | Beans and Legumes | Other Beans and Legumes | DOMESTIC | United States | Non-Detect | 1 | 0 |
| 1105804 | 2019 | 4/15/2019 | SOY BASE FORMULA PRODUCT POWDER FORMULA | Baby Food Products | Baby Formula | DOMESTIC | United States | Non-Detect | 1 | 0 |
| 1090344 | 2019 | 4/17/2019 | RICE CEREAL BABY | Baby Food Products | Baby Cereals | DOMESTIC | United States | Non-Detect | 1 | 0 |
| 1099010 | 2019 | 4/17/2019 | CORN PLAIN MEAL | Grains and Grain Products | Corn | IMPORT | Brazil | Non-Detect | 1 | 0 |
| 1099011 | 2019 | 4/17/2019 | CORN PLAIN MEAL | Grains and Grain Products | Corn | IMPORT | Brazil | Non-Detect | 1 | 0 |
| 1107121 | 2019 | 4/24/2019 | COFFEE BEANS | Non-Juice Beverages | Coffee | IMPORT | Indonesia | Non-Detect | 2.6 | 0 |
| 1106985 | 2019 | 4/24/2019 | COFFEE BEANS | Non-Juice Beverages | Coffee | IMPORT | Nicaragua | Non-Detect | 12.15 | 0 |
| 1107579 | 2019 | 4/24/2019 | BEAN DRIED OR PASTE NEC VEGETABLE | Beans and Legumes | Other Beans and Legumes | IMPORT | Australia | Non-Detect | 1 | 0 |
| 1107119 | 2019 | 4/24/2019 | COFFEE BEANS | Non-Juice Beverages | Coffee | IMPORT | Indonesia | Positive | 2.6 | 11.93 |
| 1107452 | 2019 | 4/24/2019 | BUCKWHEAT WHOLE GRAIN | Grains and Grain Products | Buckwheat | IMPORT | China | Positive | 0.79 | 2.2 |
| 1107176 | 2019 | 4/25/2019 | BUCKWHEAT FLOUR | Grains and Grain Products | Buckwheat | DOMESTIC | United States | Non-Detect | 2.6 | 0 |
| 1076279 | 2019 | 4/25/2019 | PISTACHIO SHELLED | Nuts and Edible Seeds | Pistachios | DOMESTIC | United States | Non-Detect | 1 | 0 |
| 1107437 | 2019 | 4/25/2019 | CORN ENRICHED MEAL | Grains and Grain Products | Corn | IMPORT | Spain | Non-Detect | 1 | 0 |
| 1101591 | 2019 | 4/29/2019 | OAT FLOUR | Grains and Grain Products | Oats | IMPORT | Canada | Non-Detect | 2.6 | 0 |
| 1107704 | 2019 | 4/29/2019 | BEAN CORN PEA DRIED O RPASTE NEC VEGETABLE | Beans and Legumes | Other Beans and Legumes | IMPORT | Brazil | Non-Detect | 2.6 | 0 |
| 1107894 | 2019 | 4/30/2019 | RAISINS DRIED OR PASTE | Fruit | Raisins | IMPORT | Argentina | Trace | 4.99 | |
| 1084221 | 2019 | 5/1/2019 | OAT FLOUR | Grains and Grain Products | Oats | DOMESTIC | United States | Non-Detect | 12 | 0 |
| 1108139 | 2019 | 5/1/2019 | COFFEE BEANS | Non-Juice Beverages | Coffee | IMPORT | Guatemala | Non-Detect | 2.4 | 0 |
| 1105284 | 2019 | 5/1/2019 | CORN PLAIN MEAL | Grains and Grain Products | Corn | DOMESTIC | United States | Non-Detect | 1 | 0 |
| 1070398 | 2019 | 5/2/2019 | PINTO BEAN DRIED OR PASTE | Beans and Legumes | Pinto Beans | DOMESTIC | Unknown | Non-Detect | 2.4 | 0 |
| 1082413 | 2019 | 5/7/2019 | BUCKWHEAT FLOUR | Grains and Grain Products | Buckwheat | DOMESTIC | United States | Non-Detect | 1 | 0 |
| 1089747 | 2019 | 5/8/2019 | WHEAT FLOUR ENRICHED ALL PURPOSE | Grains and Grain Products | Wheat | DOMESTIC | United States | Non-Detect | 2.6 | 0 |
| 1089748 | 2019 | 5/8/2019 | WHEAT BRAN HUMAN USE | Grains and Grain Products | Wheat | DOMESTIC | United States | Non-Detect | 2.6 | 0 |
| 1109258 | 2019 | 5/8/2019 | VEGETABLE PROTEIN PRODUCTS NOT ELSEWHERE MENTIONED NEC | Other | Other | IMPORT | China | Non-Detect | 1 | 0 |
| 1107858 | 2019 | 5/10/2019 | WHEAT WHOLE GRAIN | Grains and Grain Products | Wheat | DOMESTIC | United States | Non-Detect | 2.6 | 0 |
| 1097761 | 2019 | 5/15/2019 | BUCKWHEAT FLOUR | Grains and Grain Products | Buckwheat | DOMESTIC | United States | Non-Detect | 0.233 | 0 |
| 1110484 | 2019 | 5/17/2019 | CORN CHIPS FRIED | Processed Food Products | Other Corn Products | IMPORT | Mexico | Non-Detect | 1 | 0 |
| 1059021 | 2019 | 5/20/2019 | WHEAT FLOUR WHOLE | Grains and Grain Products | Wheat | DOMESTIC | United States | Non-Detect | 1 | 0 |
| 1104116 | 2019 | 5/21/2019 | CAPSICUMS CAYENNE CHILI HOT PEPPERS GROUND CRACKED SPICE | Spices | Capsicums, including Paprika | DOMESTIC | United States | Non-Detect | 1 | 0 |
| 1110755 | 2019 | 5/21/2019 | GARBANZO BEAN | Beans and Legumes | Garbanzo Beans | IMPORT | Canada | Non-Detect | 1 | 0 |
| 1104117 | 2019 | 5/21/2019 | GINGER GROUND CRACKED SPICE | Spices | Ginger | DOMESTIC | United States | Positive | 1.03 | 1.5 |
| 1096846 | 2019 | 5/22/2019 | BARLEY WHOLE GRAIN | Grains and Grain Products | Barley | DOMESTIC | United States | Non-Detect | 2.4 | 0 |
| 1110614 | 2019 | 5/22/2019 | WHEAT BRAN HUMAN USE | Grains and Grain Products | Wheat | DOMESTIC | United States | Non-Detect | 1 | 0 |
| 1110969 | 2019 | 5/22/2019 | MALT BARLEY | Grains and Grain Products | Barley | IMPORT | Canada | Non-Detect | 1 | 0 |
| 1111096 | 2019 | 5/23/2019 | COFFEE BEANS | Non-Juice Beverages | Coffee | IMPORT | Guatemala | Non-Detect | 1 | 0 |
| 1111360 | 2019 | 5/24/2019 | RAISINS DRIED GRAPES BERRY PUREES | Fruit | Raisins | IMPORT | Chile | Non-Detect | 1 | 0 |
| 1111329 | 2019 | 5/25/2019 | BEAN DRIED OR PASTE NEC VEGETABLE | Beans and Legumes | Other Beans and Legumes | IMPORT | Canada | Non-Detect | 1 | 0 |
| 1059022 | 2019 | 5/28/2019 | LIMA BEAN DRIED OR PASTE | Beans and Legumes | Other Beans and Legumes | DOMESTIC | Unknown | Non-Detect | 1 | 0 |
| 1059023 | 2019 | 5/28/2019 | PEPPER MIXED | Spices | Pepper | IMPORT | Unknown | Positive | 0.96 | 2.2 |
| 1108318 | 2019 | 5/29/2019 | WHEAT FLOUR ENRICHED ALL PURPOSE | Grains and Grain Products | Wheat | DOMESTIC | United States | Non-Detect | 2.4 | 0 |
| 1111756 | 2019 | 5/30/2019 | CORN BOLTED MEAL OR FLOUR | Grains and Grain Products | Corn | IMPORT | Italy | Non-Detect | 2.6 | 0 |
| 1111852 | 2019 | 5/30/2019 | COFFEE BEANS | Non-Juice Beverages | Coffee | IMPORT | Brazil | Non-Detect | 12.15 | 0 |
| 1101584 | 2019 | 5/30/2019 | RYE FLOUR | Grains and Grain Products | Rye | DOMESTIC | United States | Non-Detect | 1 | 0 |
| 1112000 | 2019 | 5/31/2019 | BARLEY WHOLE GRAIN | Grains and Grain Products | Barley | IMPORT | Peru | Non-Detect | 1 | 0 |
| 1112061 | 2019 | 6/3/2019 | MALT BARLEY | Grains and Grain Products | Barley | IMPORT | Canada | Non-Detect | 1 | 0 |
| 1112549 | 2019 | 6/3/2019 | OATS WHOLE GRAIN | Grains and Grain Products | Oats | IMPORT | Canada | Non-Detect | 1 | 0 |
| 1081305 | 2019 | 6/4/2019 | BUCKWHEAT WHOLE GRAIN | Grains and Grain Products | Buckwheat | DOMESTIC | United States | Non-Detect | 2.6 | 0 |
| 1092927 | 2019 | 6/4/2019 | BUCKWHEAT WHOLE GRAIN | Grains and Grain Products | Buckwheat | DOMESTIC | United States | Non-Detect | 2.5 | 0 |
| 1112371 | 2019 | 6/4/2019 | PINTO BEAN | Beans and Legumes | Pinto Beans | IMPORT | Canada | Non-Detect | 2.5 | 0 |
| 1112536 | 2019 | 6/4/2019 | BEAN DRIED OR PASTE NEC VEGETABLE | Beans and Legumes | Other Beans and Legumes | IMPORT | Mexico | Non-Detect | 1 | 0 |
| 1112738 | 2019 | 6/5/2019 | BARLEY WHOLE GRAIN | Grains and Grain Products | Barley | IMPORT | Canada | Non-Detect | 1 | 0 |
| 1113376 | 2019 | 6/11/2019 | OKOME SHORT GRAIN HULLED RICE THAT IS MAINSTAY IN JAPANESE DIET | Grains and Grain Products | Rice | IMPORT | Bangladesh | Non-Detect | 0.233 | 0 |
| 1033534 | 2019 | 6/11/2019 | CORN WHOLE GRAIN | Grains and Grain Products | Corn | DOMESTIC | United States | Non-Detect | 1 | 0 |
| 1103114 | 2019 | 6/11/2019 | CAPSICUMS CAYENNE CHILI HOT PEPPERS GROUND CRACKED SPICE | Spices | Capsicums, including Paprika | DOMESTIC | United States | Positive | 5 | 25.7 |
| 1109580 | 2019 | 6/13/2019 | PINTO BEAN | Beans and Legumes | Pinto Beans | DOMESTIC | United States | Non-Detect | 2.6 | 0 |
| 1109581 | 2019 | 6/13/2019 | KIDNEY BEAN | Beans and Legumes | Other Beans and Legumes | DOMESTIC | United States | Non-Detect | 2.6 | 0 |
| 1112798 | 2019 | 6/13/2019 | CAPSICUMS CAYENNE CHILI HOT PEPPERS GROUND CRACKED SPICE | Spices | Capsicums, including Paprika | DOMESTIC | United States | Positive | 2.6 | 12.43 |
| 1113841 | 2019 | 6/14/2019 | OATMEAL REGULAR FRUIT OR SPICE ADDED QUICK OR INSTANT COOKING | Processed Food Products | Breakfast Foods | IMPORT | Canada | Non-Detect | 1 | 0 |
| 1108033 | 2019 | 6/18/2019 | OAT FLOUR | Grains and Grain Products | Oats | DOMESTIC | United States | Non-Detect | 2.6 | 0 |
| 1100170 | 2019 | 6/21/2019 | SPAGHETTI | Processed Food Products | Pasta | DOMESTIC | United States | Non-Detect | 1 | 0 |
| 1114579 | 2019 | 6/21/2019 | BARLEY WHOLE GRAIN | Grains and Grain Products | Barley | IMPORT | Canada | Non-Detect | 1 | 0 |
| 1114724 | 2019 | 6/24/2019 | BLACKEYED PEA | Beans and Legumes | Blackeye Peas | IMPORT | Burma | Non-Detect | 1 | 0 |
| 1114770 | 2019 | 6/25/2019 | RICE CULTIVATED WHOLE GRAIN | Grains and Grain Products | Rice | DOMESTIC | Unknown | Non-Detect | 0.233 | 0 |
| 1009675 | 2019 | 6/26/2019 | MIXED GRAIN CEREAL BABY | Baby Food Products | Baby Cereals | DOMESTIC | United States | Positive | 1.13 | 1.2 |
| 1115222 | 2019 | 6/27/2019 | OATS WHOLE GRAIN | Grains and Grain Products | Oats | IMPORT | Russia | Non-Detect | 1 | 0 |
| 1115278 | 2019 | 6/27/2019 | BUCKWHEAT FLOUR | Grains and Grain Products | Buckwheat | IMPORT | Russia | Non-Detect | 1 | 0 |
| 1059024 | 2019 | 7/1/2019 | GARBANZO BEAN DRIED OR PASTE | Beans and Legumes | Garbanzo Beans | DOMESTIC | United States | Non-Detect | 1 | 0 |
| 1107842 | 2019 | 7/1/2019 | HOMINY | Grains and Grain Products | Corn | DOMESTIC | Unknown | Non-Detect | 1 | 0 |
| 1115288 | 2019 | 7/1/2019 | COFFEE BEANS | Non-Juice Beverages | Coffee | IMPORT | Guatemala | Non-Detect | 1 | 0 |
| 1098548 | 2019 | 7/10/2019 | SPAGHETTI | Processed Food Products | Pasta | DOMESTIC | United States | Non-Detect | 1 | 0 |
| 1105085 | 2019 | 7/11/2019 | OATS WHOLE GRAIN | Grains and Grain Products | Oats | DOMESTIC | United States | Non-Detect | 1 | 0 |
| 1116274 | 2019 | 7/11/2019 | COFFEE BEANS | Non-Juice Beverages | Coffee | IMPORT | Guatemala | Non-Detect | 1 | 0 |
| 1116443 | 2019 | 7/15/2019 | BARLEY WHOLE GRAIN | Grains and Grain Products | Barley | IMPORT | Canada | Non-Detect | 2.6 | 0 |
| 1038404 | 2019 | 7/15/2019 | OATS WHOLE GRAIN | Grains and Grain Products | Oats | IMPORT | Canada | Non-Detect | 0.233 | 0 |
| 1038405 | 2019 | 7/16/2019 | CORN BOLTED MEAL OR FLOUR | Grains and Grain Products | Corn | DOMESTIC | United States | Non-Detect | 0.233 | 0 |
| 1116619 | 2019 | 7/16/2019 | COFFEE BEANS | Non-Juice Beverages | Coffee | IMPORT | Colombia | Non-Detect | 12.15 | 0 |
| 1115933 | 2019 | 7/18/2019 | CORN ENRICHED MEAL | Grains and Grain Products | Corn | DOMESTIC | United States | Non-Detect | 2.6 | 0 |
| 1082418 | 2019 | 7/18/2019 | BUCKWHEAT FLOUR | Grains and Grain Products | Buckwheat | DOMESTIC | United States | Non-Detect | 0.233 | 0 |
| 1114131 | 2019 | 7/18/2019 | SOY BASE FORMULA PRODUCT READY TO FEED | Baby Food Products | Baby Formula | DOMESTIC | Unknown | Non-Detect | 1 | 0 |
| 1104931 | 2019 | 7/22/2019 | BARLEY CEREAL BABY | Baby Food Products | Baby Cereals | DOMESTIC | United States | Non-Detect | 0.233 | 0 |
| 1117150 | 2019 | 7/22/2019 | CORN PLAIN MEAL | Grains and Grain Products | Corn | IMPORT | Mexico | Non-Detect | 0.233 | 0 |
| 1117011 | 2019 | 7/22/2019 | PEAS | Beans and Legumes | Peas | IMPORT | Canada | Non-Detect | 1 | 0 |
| 1117168 | 2019 | 7/23/2019 | CAPSICUMS CAYENNE CHILI HOT PEPPERS WHOLE SPICE | Spices | Capsicums, including Paprika | IMPORT | Mexico | Non-Detect | 0.233 | 0 |
| 1117134 | 2019 | 7/23/2019 | WHEAT WHOLE GRAIN | Grains and Grain Products | Wheat | IMPORT | Canada | Non-Detect | 1 | 0 |
| 1117568 | 2019 | 7/24/2019 | COFFEE BEANS | Non-Juice Beverages | Coffee | IMPORT | Guatemala | Non-Detect | 2.6 | 0 |
| 1117327 | 2019 | 7/24/2019 | MALT BARLEY | Grains and Grain Products | Barley | IMPORT | Canada | Non-Detect | 1 | 0 |
| 1117711 | 2019 | 7/26/2019 | WHEAT WHOLE GRAIN | Grains and Grain Products | Wheat | IMPORT | Canada | Non-Detect | 2.6 | 0 |
| 1101736 | 2019 | 7/29/2019 | WHEAT CEREAL BABY | Baby Food Products | Baby Cereals | DOMESTIC | United States | Non-Detect | 2.6 | 0 |

| | | | | | | | | | | |
|---|---|---|---|---|---|---|---|---|---|---|
| 1101737 | 2019 | 7/30/2019 | MALT BARLEY | Grains and Grain Products | Barley | IMPORT | Italy | Non-Detect | 0.233 | 0 |
| 1108294 | 2019 | 7/30/2019 | BARLEY CEREAL BABY | Baby Food Products | Baby Cereals | DOMESTIC | United States | Non-Detect | 1 | 0 |
| 1099629 | 2019 | 8/1/2019 | WHEAT CEREAL BABY | Baby Food Products | Baby Cereals | DOMESTIC | United States | Non-Detect | 0.233 | 0 |
| 1078132 | 2019 | 8/1/2019 | CORN PLAIN MEAL | Grains and Grain Products | Corn | DOMESTIC | United States | Non-Detect | 1 | 0 |
| 1080130 | 2019 | 8/1/2019 | BARLEY WHOLE GRAIN | Grains and Grain Products | Barley | DOMESTIC | United States | Non-Detect | 1 | 0 |
| 1118345 | 2019 | 8/1/2019 | WHEAT WHOLE GRAIN | Grains and Grain Products | Wheat | IMPORT | Canada | Non-Detect | 1 | 0 |
| 1118369 | 2019 | 8/1/2019 | COFFEE BEANS | Non-Juice Beverages | Coffee | IMPORT | Guatemala | Non-Detect | 1 | 0 |
| 1118425 | 2019 | 8/1/2019 | COFFEE BEANS | Non-Juice Beverages | Coffee | IMPORT | Colombia | Non-Detect | 1 | 0 |
| 969554 | 2019 | 8/1/2019 | WHOLE SPICE NEC | Spices | Other Spices | DOMESTIC | United States | Positive | 12 | 7.56 |
| 1115485 | 2019 | 8/3/2019 | FIG DRIED OR PASTE | Fruit | Dried Figs | DOMESTIC | United States | Non-Detect | 0.233 | 0 |
| 1117039 | 2019 | 8/6/2019 | OATS WHOLE GRAIN | Grains and Grain Products | Oats | IMPORT | Ireland | Non-Detect | 2.6 | 0 |
| 1118648 | 2019 | 8/6/2019 | RAISINS DRIED GRAPES BERRY | Fruit | Raisins | IMPORT | South Africa | Non-Detect | 2.6 | 0 |
| 1113684 | 2019 | 8/6/2019 | GINGER WHOLE SPICE | Spices | Ginger | IMPORT | India | Trace | 5 | |
| 1115256 | 2019 | 8/7/2019 | PEPPER BLACK GROUND CRACKED SPICE | Spices | Pepper | DOMESTIC | United States | Non-Detect | 2.6 | 0 |
| 1119129 | 2019 | 8/7/2019 | WHEAT WHOLE GRAIN | Grains and Grain Products | Wheat | DOMESTIC | United States | Non-Detect | 1 | 0 |
| 1119130 | 2019 | 8/7/2019 | BUCKWHEAT WHOLE GRAIN | Grains and Grain Products | Buckwheat | DOMESTIC | United States | Non-Detect | 1 | 0 |
| 1084025 | 2019 | 8/12/2019 | PISTACHIO SHELLED | Nuts and Edible Seeds | Pistachios | DOMESTIC | United States | Non-Detect | 1 | 0 |
| 1112620 | 2019 | 8/12/2019 | BUCKWHEAT WHOLE GRAIN | Grains and Grain Products | Buckwheat | DOMESTIC | United States | Non-Detect | 1 | 0 |
| 1119402 | 2019 | 8/12/2019 | BARLEY WHOLE GRAIN | Grains and Grain Products | Barley | IMPORT | Italy | Non-Detect | 1 | 0 |
| 1105316 | 2019 | 8/13/2019 | RYE FLOUR | Grains and Grain Products | Rye | DOMESTIC | United States | Non-Detect | 0.233 | 0 |
| 1105925 | 2019 | 8/14/2019 | BUCKWHEAT FLOUR | Grains and Grain Products | Buckwheat | DOMESTIC | United States | Non-Detect | 0.233 | 0 |
| 1105926 | 2019 | 8/14/2019 | RYE FLOUR | Grains and Grain Products | Rye | DOMESTIC | United States | Non-Detect | 0.233 | 0 |
| 1105927 | 2019 | 8/14/2019 | BUCKWHEAT FLOUR | Grains and Grain Products | Buckwheat | DOMESTIC | United States | Non-Detect | 0.233 | 0 |
| 1076836 | 2019 | 8/15/2019 | BARLEY WHOLE GRAIN | Grains and Grain Products | Barley | DOMESTIC | United States | Non-Detect | 1 | 0 |
| 1112139 | 2019 | 8/19/2019 | RICE CEREAL BABY | Baby Food Products | Baby Cereals | DOMESTIC | United States | Non-Detect | 2.6 | 0 |
| 1120269 | 2019 | 8/19/2019 | COFFEE BEANS | Non-Juice Beverages | Coffee | IMPORT | El Salvador | Non-Detect | 2.6 | 0 |
| 1059027 | 2019 | 8/20/2019 | WHEAT FLOUR WHOLE | Grains and Grain Products | Wheat | DOMESTIC | United States | Non-Detect | 1 | 0 |
| 1059028 | 2019 | 8/20/2019 | PEPPER BLACK GROUND CRACKED SPICE | Spices | Pepper | IMPORT | India | Non-Detect | 0.9 | 0 |
| 1090967 | 2019 | 8/20/2019 | RICE CULTIVATED WHOLE GRAIN | Grains and Grain Products | Rice | DOMESTIC | United States | Non-Detect | 1 | 0 |
| 1117042 | 2019 | 8/20/2019 | CAPSICUMS CAYENNE CHILI HOT PEPPERS GROUND CRACKED SPICE | Spices | Capsicums, including Paprika | DOMESTIC | United States | Positive | 2.6 | 10.41 |
| 1012704 | 2019 | 8/21/2019 | SOY BASE FORMULA PRODUCT POWDER FORMULA | Baby Food Products | Baby Formula | DOMESTIC | United States | Non-Detect | 2.4 | 0 |
| 1120429 | 2019 | 8/21/2019 | OAT FLOUR | Grains and Grain Products | Oats | IMPORT | Canada | Non-Detect | 2.6 | 0 |
| 1120726 | 2019 | 8/22/2019 | CORN CHIPS FRIED | Processed Food Products | Other Corn Products | IMPORT | Mexico | Non-Detect | 1 | 0 |
| 1120792 | 2019 | 8/22/2019 | SPAGHETTI | Processed Food Products | Pasta | IMPORT | Italy | Non-Detect | 0.84 | 0 |
| 1083709 | 2019 | 8/23/2019 | BUCKWHEAT WHOLE GRAIN | Grains and Grain Products | Buckwheat | DOMESTIC | United States | Non-Detect | 0.84 | 0 |
| 1112624 | 2019 | 8/23/2019 | RICE CEREAL BABY | Baby Food Products | Baby Cereals | DOMESTIC | United States | Non-Detect | 0.84 | 0 |
| 1117044 | 2019 | 8/23/2019 | CAPSICUMS CAYENNE CHILI HOT PEPPERS GROUND CRACKED SPICE | Spices | Capsicums, including Paprika | DOMESTIC | Unknown | Positive | 1 | 18 |
| 1117045 | 2019 | 8/26/2019 | GINGER GROUND CRACKED SPICE | Spices | Ginger | DOMESTIC | United States | Non-Detect | 2.6 | 0 |
| 1121196 | 2019 | 8/27/2019 | BEAN DRIED OR PASTE NEC VEGETABLE | Beans and Legumes | Other Beans and Legumes | IMPORT | India | Non-Detect | 0.233 | 0 |
| 1121113 | 2019 | 8/28/2019 | BUCKWHEAT WHOLE GRAIN | Grains and Grain Products | Buckwheat | DOMESTIC | United States | Non-Detect | 0.233 | 0 |
| 1119133 | 2019 | 9/3/2019 | BARLEY WHOLE GRAIN | Grains and Grain Products | Barley | DOMESTIC | United States | Trace | 1 | |
| 1121473 | 2019 | 9/4/2019 | OATS WHOLE GRAIN | Grains and Grain Products | Oats | DOMESTIC | United States | Non-Detect | 2.6 | 0 |
| 1122001 | 2019 | 9/5/2019 | RAISINS DRIED OR PASTE | Fruit | Raisins | IMPORT | Chile | Trace | 1 | |
| 1122321 | 2019 | 9/10/2019 | FARRO | Grains and Grain Products | Wheat | IMPORT | Canada | Non-Detect | 1 | 0 |
| 1123259 | 2019 | 9/18/2019 | CAPSICUMS CAYENNE CHILI HOT PEPPERS GROUND CRACKED SPICE | Spices | Capsicums, including Paprika | IMPORT | India | Positive | 1.02 | 1.3 |
| 1123553 | 2019 | 9/20/2019 | RAISINS DRIED GRAPES BERRY | Fruit | Raisins | IMPORT | Argentina | Positive | 1 | 2 |
| 1120745 | 2019 | 9/23/2019 | OATMEAL REGULAR FRUIT OR SPICE ADDED QUICK OR INSTANT COOKING | Processed Food Products | Breakfast Foods | DOMESTIC | United States | Non-Detect | 0.233 | 0 |
| 1121641 | 2019 | 9/25/2019 | BARLEY WHOLE GRAIN | Grains and Grain Products | Barley | IMPORT | Canada | Non-Detect | 0.233 | 0 |
| 1118787 | 2019 | 9/26/2019 | PISTACHIO IN SHELL | Nuts and Edible Seeds | Pistachios | DOMESTIC | United States | Non-Detect | 1 | 0 |
| 1124422 | 2019 | 9/26/2019 | RAISINS DRIED OR PASTE | Fruit | Raisins | IMPORT | South Africa | Non-Detect | 1 | 0 |
| 1078087 | 2019 | 9/26/2019 | FIG DRIED OR PASTE | Fruit | Dried Figs | DOMESTIC | United States | Trace | 5 | |
| 1121323 | 2019 | 9/30/2019 | PISTACHIO SHELLED | Nuts and Edible Seeds | Pistachios | DOMESTIC | Unknown | Non-Detect | 2.6 | 0 |
| 1124401 | 2019 | 9/30/2019 | RYE FLOUR | Grains and Grain Products | Rye | DOMESTIC | United States | Non-Detect | 2.6 | 0 |
| 1124402 | 2019 | 9/30/2019 | RYE FLOUR | Grains and Grain Products | Rye | DOMESTIC | United States | Non-Detect | 1 | 0 |

**FY2020 Data**

| Sample Number | Fiscal Year | Collection Date | Product Name | Food Category | Food Subcategory | Origin Type | Country of Origin | Non-Detect, Trace, or Positive (>LOQ) | LOQ (ppb) | Amount found (ppb) |
|---|---|---|---|---|---|---|---|---|---|---|
| 1084964 | 2020 | 10/1/2019 | SOYBEANS WHOLE GRAIN | Beans and Legumes | Soybeans | DOMESTIC | United States | Non-Detect | 1 | 0 |
| 1124352 | 2020 | 10/2/2019 | KIDNEY BEAN | Beans and Legumes | Other Beans and Legumes | DOMESTIC | United States | Non-Detect | 1 | 0 |
| 1125033 | 2020 | 10/3/2019 | BARLEY WHOLE GRAIN | Grains and Grain Products | Barley | IMPORT | Turkey | Non-Detect | 0.233 | 0 |
| 1125044 | 2020 | 10/3/2019 | MACARONI | Processed Food Products | Pasta | IMPORT | Italy | Non-Detect | 1 | 0 |
| 1125119 | 2020 | 10/3/2019 | CRANBERRY BEAN DRIED OR PASTE | Beans and Legumes | Other Beans and Legumes | IMPORT | Canada | Non-Detect | 1 | 0 |
| 1125481 | 2020 | 10/8/2019 | WHEAT FLOUR NEC | Grains and Grain Products | Wheat | IMPORT | Guatemala | Trace | 5 | |
| 1086508 | 2020 | 10/9/2019 | KIDNEY BEAN DRIED OR PASTE | Beans and Legumes | Other Beans and Legumes | DOMESTIC | United States | Non-Detect | 0.233 | 0 |
| 1125818 | 2020 | 10/9/2019 | CHICKPEA DRIED OR PASTE | Beans and Legumes | Garbanzo Beans | IMPORT | Afghanistan | Non-Detect | 0.233 | 0 |
| 1126183 | 2020 | 10/9/2019 | RAISINS DRIED OR PASTE | Fruit | Raisins | IMPORT | United States | Non-Detect | 2.629 | 0 |
| 1125958 | 2020 | 10/10/2019 | RICE PLAIN WHITE OR POLISHED PROCESSED PACKAGED | Grains and Grain Products | Rice | IMPORT | India | Non-Detect | 0.233 | 0 |
| 1117758 | 2020 | 10/16/2019 | RAISINS DRIED OR PASTE | Fruit | Raisins | DOMESTIC | United States | Non-Detect | 2.629 | 0 |
| 1117759 | 2020 | 10/16/2019 | PISTACHIO IN SHELL | Nuts and Edible Seeds | Pistachios | DOMESTIC | United States | Non-Detect | 1 | 0 |
| 1126200 | 2020 | 10/16/2019 | WHEAT FLOUR ENRICHED ALL PURPOSE | Grains and Grain Products | Wheat | DOMESTIC | Unknown | Non-Detect | 1 | 0 |
| 1126363 | 2020 | 10/16/2019 | PISTACHIO IN SHELL | Nuts and Edible Seeds | Pistachios | DOMESTIC | United States | Non-Detect | 1 | 0 |
| 1126492 | 2020 | 10/16/2019 | TORTELLINI MULTIPLE FOOD SPECIALITIES SIDE DISHES AND DESSERTS | Processed Food Products | Other Processed Foods | IMPORT | Canada | Non-Detect | 1 | 0 |
| 1125113 | 2020 | 10/16/2019 | WHEAT FLOUR PLAIN | Grains and Grain Products | Wheat | DOMESTIC | United States | Positive | 5 | 19.4 |
| 1126362 | 2020 | 10/16/2019 | RAISINS DRIED OR PASTE | Fruit | Raisins | DOMESTIC | United States | Trace | 10 | |
| 1117305 | 2020 | 10/17/2019 | BUCKWHEAT FLOUR | Grains and Grain Products | Buckwheat | DOMESTIC | United States | Non-Detect | 1 | 0 |
| 1119857 | 2020 | 10/17/2019 | BUCKWHEAT FLOUR | Grains and Grain Products | Buckwheat | DOMESTIC | United States | Non-Detect | 1 | 0 |
| 1119858 | 2020 | 10/17/2019 | RYE FLOUR | Grains and Grain Products | Rye | DOMESTIC | United States | Non-Detect | 1 | 0 |
| 1123239 | 2020 | 10/17/2019 | GARBANZO BEAN | Beans and Legumes | Garbanzo Beans | DOMESTIC | United States | Non-Detect | 1 | 0 |
| 1126797 | 2020 | 10/18/2019 | CORN WHOLE GRAIN | Grains and Grain Products | Corn | IMPORT | India | Non-Detect | 1 | 0 |
| 1127274 | 2020 | 10/21/2019 | VEGETABLE SPAGHETTI PRODUCTS | Processed Food Products | Pasta | IMPORT | Italy | Non-Detect | 1 | 0 |
| 1127034 | 2020 | 10/22/2019 | OAT FLOUR | Grains and Grain Products | Oats | DOMESTIC | United States | Non-Detect | 0.233 | 0 |
| 1127165 | 2020 | 10/23/2019 | COFFEE BEANS | Non-Juice Beverages | Coffee | IMPORT | Brazil | Non-Detect | 1 | 0 |
| 1127429 | 2020 | 10/24/2019 | FIG DRIED OR PASTE | Fruit | Dried Figs | DOMESTIC | United States | Non-Detect | 1 | 0 |
| 1100200 | 2020 | 10/28/2019 | PISTACHIO IN SHELL | Nuts and Edible Seeds | Pistachios | DOMESTIC | Unknown | Non-Detect | 1 | 0 |
| 1125114 | 2020 | 10/30/2019 | RYE FLOUR | Grains and Grain Products | Rye | DOMESTIC | United States | Non-Detect | 0.233 | 0 |
| 1128287 | 2020 | 10/30/2019 | COFFEE BEANS | Non-Juice Beverages | Coffee | IMPORT | Colombia | Non-Detect | 1 | 0 |
| 1128441 | 2020 | 10/30/2019 | RAISINS DRIED OR PASTE | Fruit | Raisins | IMPORT | South Africa | Non-Detect | 1 | 0 |
| 1128458 | 2020 | 10/30/2019 | RAISINS DRIED OR PASTE | Fruit | Raisins | IMPORT | Chile | Non-Detect | 1 | 0 |
| 1128059 | 2020 | 10/30/2019 | PEAS | Beans and Legumes | Peas | IMPORT | Canada | Positive | 1 | 5.4 |
| 1128288 | 2020 | 10/30/2019 | COFFEE BEANS | Non-Juice Beverages | Coffee | IMPORT | Vietnam | Positive | 1 | 1.4 |
| 1128975 | 2020 | 10/31/2019 | RAISINS DRIED OR PASTE | Fruit | Raisins | IMPORT | Chile | Non-Detect | 1 | 0 |
| 1128685 | 2020 | 11/4/2019 | RYE FLOUR | Grains and Grain Products | Rye | IMPORT | Canada | Non-Detect | 2.6 | 0 |
| 1128932 | 2020 | 11/5/2019 | AZUFRADO BEAN | Beans and Legumes | Other Beans and Legumes | IMPORT | Mexico | Non-Detect | 0.233 | 0 |
| 1129019 | 2020 | 11/5/2019 | MILLED GRAIN PRODUCTS NEC | Grains and Grain Products | Other Grains | IMPORT | Korea (the Republic of) | Non-Detect | 0.233 | 0 |
| 1126509 | 2020 | 11/5/2019 | WHEAT WHOLE GRAIN | Grains and Grain Products | Wheat | DOMESTIC | United States | Non-Detect | 1.02 | 0 |
| 1128954 | 2020 | 11/5/2019 | MALT BARLEY | Grains and Grain Products | Barley | IMPORT | United Kingdom | Non-Detect | 1 | 0 |
| 1128966 | 2020 | 11/5/2019 | MALT BARLEY | Grains and Grain Products | Barley | IMPORT | United Kingdom | Non-Detect | 1 | 0 |
| 1129201 | 2020 | 11/6/2019 | COFFEE BEANS | Non-Juice Beverages | Coffee | IMPORT | Colombia | Non-Detect | 12.15 | 0 |
| 1129235 | 2020 | 11/7/2019 | COFFEE BEANS | Non-Juice Beverages | Coffee | IMPORT | Colombia | Non-Detect | 1 | 0 |
| 1129246 | 2020 | 11/7/2019 | RAISINS DRIED OR PASTE | Fruit | Raisins | DOMESTIC | United States | Non-Detect | 1 | 0 |
| 1129226 | 2020 | 11/8/2019 | OATS WHOLE GRAIN | Grains and Grain Products | Oats | IMPORT | Canada | Non-Detect | 0.233 | 0 |
| 1129227 | 2020 | 11/8/2019 | RYE FLOUR | Grains and Grain Products | Rye | IMPORT | Canada | Non-Detect | 0.233 | 0 |
| 1129350 | 2020 | 11/8/2019 | RAISINS DRIED OR PASTE | Fruit | Raisins | IMPORT | Canada | Positive | 1 | 5.2 |
| 1110437 | 2020 | 11/12/2019 | PINTO BEAN DRIED OR PASTE | Beans and Legumes | Pinto Beans | DOMESTIC | United States | Non-Detect | 1 | 0 |
| 1127446 | 2020 | 11/13/2019 | PISTACHIO SHELLED | Nuts and Edible Seeds | Pistachios | DOMESTIC | United States | Non-Detect | 2.6 | 0 |
| 1129571 | 2020 | 11/13/2019 | HOMMUS HUMMUS MULTIPLE FOOD SPECIALTIES SIDE DISHES AND DESSERTS | Processed Food Products | Other Processed Foods | IMPORT | Turkey | Non-Detect | 2.6 | 0 |
| 1129228 | 2020 | 11/13/2019 | WHEAT BRAN HUMAN USE | Grains and Grain Products | Wheat | DOMESTIC | United States | Non-Detect | 1 | 0 |
| 1129229 | 2020 | 11/13/2019 | WHEAT GERM | Grains and Grain Products | Wheat | DOMESTIC | United States | Non-Detect | 1 | 0 |
| 1129730 | 2020 | 11/14/2019 | CORN FLAKES PUFFS KRISPIES LOOPS READY TO EAT | Processed Food Products | Other Corn Products | IMPORT | Mexico | Non-Detect | 1 | 0 |
| 1130222 | 2020 | 11/14/2019 | CHANA DAL DRIED OR PASTE | Beans and Legumes | Garbanzo Beans | IMPORT | India | Non-Detect | 1 | 0 |
| 1130252 | 2020 | 11/15/2019 | COFFEE BEANS | Non-Juice Beverages | Coffee | IMPORT | Italy | Non-Detect | 1 | 0 |
| 1130218 | 2020 | 11/18/2019 | COFFEE BEANS | Non-Juice Beverages | Coffee | IMPORT | Peru | Non-Detect | 1.08 | 0 |
| 1130236 | 2020 | 11/18/2019 | KIDNEY BEAN | Beans and Legumes | Other Beans and Legumes | IMPORT | Nicaragua | Non-Detect | 1 | 0 |
| 1130243 | 2020 | 11/18/2019 | COFFEE BEANS | Non-Juice Beverages | Coffee | IMPORT | Colombia | Non-Detect | 1 | 0 |
| 1130315 | 2020 | 11/18/2019 | COFFEE BEANS | Non-Juice Beverages | Coffee | IMPORT | Colombia | Non-Detect | 1 | 0 |
| 1130430 | 2020 | 11/19/2019 | BLACK EYE BEANS | Beans and Legumes | Blackeye Peas | IMPORT | Mexico | Non-Detect | 1 | 0 |
| 1131178 | 2020 | 11/25/2019 | COFFEE BEANS | Non-Juice Beverages | Coffee | IMPORT | Brazil | Non-Detect | 2.6 | 0 |
| 1131285 | 2020 | 11/26/2019 | COFFEE BEANS | Non-Juice Beverages | Coffee | IMPORT | Indonesia | Non-Detect | 1 | 0 |
| 1131286 | 2020 | 11/26/2019 | COFFEE BEANS | Non-Juice Beverages | Coffee | IMPORT | Colombia | Non-Detect | 1 | 0 |
| 1131326 | 2020 | 11/26/2019 | CORN BOLTED MEAL OR FLOUR | Grains and Grain Products | Corn | IMPORT | Mexico | Non-Detect | 1 | 0 |
| 1127943 | 2020 | 12/2/2019 | PUMPKIN SEED EDIBLE SEED | Nuts and Edible Seeds | Pumpkin Seeds | DOMESTIC | United States | Non-Detect | 1 | 0 |
| 1131642 | 2020 | 12/2/2019 | OATS WHOLE GRAIN | Grains and Grain Products | Oats | IMPORT | Canada | Non-Detect | 1 | 0 |
| 1131855 | 2020 | 12/3/2019 | COFFEE BEANS | Non-Juice Beverages | Coffee | IMPORT | Peru | Non-Detect | 12.15 | 0 |
| 1132033 | 2020 | 12/4/2019 | OATMEAL REGULAR FRUIT OR SPICE ADDED QUICK OR INSTANT COOKING | Processed Food Products | Breakfast Foods | IMPORT | Australia | Non-Detect | 1 | 0 |
| 1131940 | 2020 | 12/4/2019 | GINGER GROUND CRACKED SPICE | Spices | Ginger | IMPORT | Canada | Positive | 5 | 6.93 |
| 1132181 | 2020 | 12/5/2019 | SOYBEANS SEE INDUSTRY 37 FOR 'BEAN CURD' | Beans and Legumes | Soybeans | IMPORT | Taiwan | Non-Detect | 1 | 0 |
| 1132221 | 2020 | 12/6/2019 | RICE CULTIVATED WHOLE GRAIN | Grains and Grain Products | Rice | IMPORT | Spain | Non-Detect | 1 | 0 |
| 1120926 | 2020 | 12/9/2019 | PISTACHIO IN SHELL | Nuts and Edible Seeds | Pistachios | DOMESTIC | United States | Non-Detect | 2.5 | 0 |
| 1100210 | 2020 | 12/11/2019 | PISTACHIO IN SHELL | Nuts and Edible Seeds | Pistachios | DOMESTIC | Unknown | Non-Detect | 1 | 0 |
| 1132894 | 2020 | 12/12/2019 | FRUIT FILLED COOKIE BISCUIT WAFER DOUGH | Processed Food Products | Other Bakery Products | IMPORT | United Arab Emirates | Non-Detect | 2.5 | 0 |
| 1132979 | 2020 | 12/13/2019 | COFFEE BEANS | Non-Juice Beverages | Coffee | IMPORT | Guatemala | Non-Detect | 2.5 | 0 |
| 1133099 | 2020 | 12/13/2019 | NAVY WHITE BEANS BAKED BEANS PORK BEANS DRIED OR PASTE | Beans and Legumes | Other Beans and Legumes | IMPORT | Canada | Non-Detect | 1 | 0 |
| 1115870 | 2020 | 12/16/2019 | RYE FLOUR | Grains and Grain Products | Rye | DOMESTIC | Unknown | Non-Detect | 2.5 | 0 |
| 1133252 | 2020 | 12/16/2019 | WHEAT FLAKES BUCKWHEAT PUFFS KRISPIES LOOPS SHREDDED WHEAT GERM READY TO EAT | Processed Food Products | Breakfast Foods | IMPORT | Poland | Non-Detect | 2.5 | 0 |
| 1133260 | 2020 | 12/16/2019 | OAT FLAKES ROLLED OATS PUFFS KRISPIES LOOPS READY TO EAT | Processed Food Products | Breakfast Foods | IMPORT | Germany | Non-Detect | 2.5 | 0 |
| 1133117 | 2020 | 12/16/2019 | COFFEE BEANS | Non-Juice Beverages | Coffee | IMPORT | Honduras | Non-Detect | 1 | 0 |
| 1090624 | 2020 | 12/17/2019 | PISTACHIO IN SHELL | Nuts and Edible Seeds | Pistachios | DOMESTIC | United States | Non-Detect | 2.5 | 0 |
| 1133259 | 2020 | 12/17/2019 | BLACKEYE PEAS DRIED OR PASTE | Beans and Legumes | Blackeye Peas | IMPORT | Peru | Non-Detect | 2.5 | 0 |
| 1133365 | 2020 | 12/17/2019 | HOMMUS HUMMUS MULTIPLE FOOD SPECIALTIES SIDE DISHES AND DESSERTS | Processed Food Products | Other Processed Foods | IMPORT | Canada | Non-Detect | 2.5 | 0 |
| 1133380 | 2020 | 12/17/2019 | HOMMUS HUMMUS MULTIPLE FOOD SPECIALTIES SIDE DISHES AND DESSERTS | Processed Food Products | Other Processed Foods | IMPORT | Canada | Non-Detect | 2.5 | 0 |

| | | | | | | | | | | |
|---|---|---|---|---|---|---|---|---|---|---|
| 1133283 | 2020 | 12/17/2019 | BARLEY FLOUR | Grains and Grain Products | Barley | IMPORT | Mexico | Non-Detect | 0.233 | 0 |
| 1132273 | 2020 | 12/18/2019 | OATS WHOLE GRAIN | Grains and Grain Products | Oats | DOMESTIC | United States | Non-Detect | 2.5 | 0 |
| 1132274 | 2020 | 12/18/2019 | RAISINS DRIED OR PASTE | Fruit | Raisins | DOMESTIC | United States | Non-Detect | 2.5 | 0 |
| 1131114 | 2020 | 12/18/2019 | RYE FLOUR | Grains and Grain Products | Rye | DOMESTIC | United States | Non-Detect | 0.233 | 0 |
| 1133692 | 2020 | 12/18/2019 | GINGER GROUND CRACKED SPICE | Spices | Ginger | IMPORT | India | Positive | 1.03 | 5.4 |
| 1133435 | 2020 | 12/19/2019 | WHEAT FLOUR BLEACHED | Grains and Grain Products | Wheat | DOMESTIC | United States | Non-Detect | 1 | 0 |
| 1133596 | 2020 | 12/19/2019 | OAT FLAKES ROLLED OATS PUFFS KRISPIES LOOPS READY TO EAT | Processed Food Products | Breakfast Foods | IMPORT | Canada | Non-Detect | 1 | 0 |
| 1113471 | 2020 | 12/19/2019 | OAT FLOUR | Grains and Grain Products | Oats | DOMESTIC | United States | Positive | 1 | 1.9 |
| 1133934 | 2020 | 12/20/2019 | TURMERIC WHOLE SPICE | Spices | Turmeric | IMPORT | India | Non-Detect | 0.233 | 0 |
| 1133937 | 2020 | 12/20/2019 | NUTMEG GROUND CRACKED SPICE | Spices | Nutmeg | IMPORT | India | Positive | 5 | 20.2 |
| 1133717 | 2020 | 12/26/2019 | HOMINY DRIED OR PASTE | Grains and Grain Products | Corn | DOMESTIC | Unknown | Positive | 1.03 | 1.6 |
| 1133774 | 2020 | 12/27/2019 | COFFEE BEANS | Non-Juice Beverages | Coffee | IMPORT | Indonesia | Non-Detect | 1 | 0 |
| 1130740 | 2020 | 1/2/2020 | PISTACHIO IN SHELL | Nuts and Edible Seeds | Pistachios | DOMESTIC | United States | Positive | 1 | 9.5 |
| 1134276 | 2020 | 1/3/2020 | BARLEY WHOLE GRAIN | Grains and Grain Products | Barley | IMPORT | Ethiopia | Non-Detect | 0.233 | 0 |
| 1121546 | 2020 | 1/6/2020 | BUCKWHEAT FLOUR | Grains and Grain Products | Buckwheat | DOMESTIC | Unknown | Non-Detect | 0.233 | 0 |
| 1121547 | 2020 | 1/6/2020 | BUCKWHEAT FLOUR | Grains and Grain Products | Buckwheat | DOMESTIC | Unknown | Non-Detect | 0.233 | 0 |
| 1134467 | 2020 | 1/6/2020 | LENTILS DRIED OR PASTE | Beans and Legumes | Lentils | IMPORT | Canada | Non-Detect | 1 | 0 |
| 1106593 | 2020 | 1/8/2020 | WHOLE WHEAT FLOUR | Grains and Grain Products | Wheat | DOMESTIC | United States | Non-Detect | 2.5 | 0 |
| 1134476 | 2020 | 1/8/2020 | LENTILS DRIED OR PASTE | Beans and Legumes | Lentils | IMPORT | United Arab Emirates | Non-Detect | 2.5 | 0 |
| 1120929 | 2020 | 1/8/2020 | NAVY WHITE BEANS BAKED BEANS PORK BEANS | Beans and Legumes | Other Beans and Legumes | DOMESTIC | United States | Non-Detect | 1 | 0 |
| 1127948 | 2020 | 1/8/2020 | BARLEY WHOLE GRAIN | Grains and Grain Products | Barley | DOMESTIC | United States | Non-Detect | 1 | 0 |
| 1132692 | 2020 | 1/8/2020 | GINGER GROUND CRACKED SPICE | Spices | Ginger | DOMESTIC | Unknown | Positive | 2.5 | 3 |
| 1122941 | 2020 | 1/9/2020 | WHEAT FLOUR WHOLE | Grains and Grain Products | Wheat | DOMESTIC | United States | Non-Detect | 2.5 | 0 |
| 1131077 | 2020 | 1/9/2020 | WHEAT FLOUR WHOLE | Grains and Grain Products | Wheat | DOMESTIC | United States | Non-Detect | 2.5 | 0 |
| 1123295 | 2020 | 1/9/2020 | RICE CULTIVATED WHOLE GRAIN | Grains and Grain Products | Rice | DOMESTIC | United States | Non-Detect | 0.233 | 0 |
| 1134636 | 2020 | 1/9/2020 | OATMEAL REGULAR FRUIT OR SPICE ADDED QUICK OR INSTANT COOKING | Processed Food Products | Breakfast Foods | IMPORT | Mexico | Non-Detect | 0.233 | 0 |
| 1134909 | 2020 | 1/9/2020 | BARLEY WHOLE GRAIN | Grains and Grain Products | Barley | IMPORT | Canada | Non-Detect | 0.233 | 0 |
| 1134808 | 2020 | 1/10/2020 | WHEAT CEREAL BABY | Baby Food Products | Baby Cereals | IMPORT | United Kingdom | Non-Detect | 2.5 | 0 |
| 1134938 | 2020 | 1/13/2020 | KIDNEY BEAN | Beans and Legumes | Other Beans and Legumes | IMPORT | Nicaragua | Non-Detect | 2.5 | 0 |
| 1130285 | 2020 | 1/15/2020 | PINTO BEAN | Beans and Legumes | Pinto Beans | DOMESTIC | United States | Non-Detect | 1 | 0 |
| 1132280 | 2020 | 1/15/2020 | PISTACHIO IN SHELL | Nuts and Edible Seeds | Pistachios | DOMESTIC | Unknown | Non-Detect | 1 | 0 |
| 1135211 | 2020 | 1/15/2020 | COFFEE INSTANT | Non-Juice Beverages | Coffee | IMPORT | Brazil | Non-Detect | 1 | 0 |
| 1135356 | 2020 | 1/15/2020 | FETTUCINE | Processed Food Products | Pasta | IMPORT | Italy | Non-Detect | 1 | 0 |
| 1132776 | 2020 | 1/16/2020 | RICE CEREAL BABY | Baby Food Products | Baby Cereals | DOMESTIC | Unknown | Non-Detect | 1 | 0 |
| 1135603 | 2020 | 1/17/2020 | ADZUKI BEANS DRIED OR PASTE | Beans and Legumes | Other Beans and Legumes | IMPORT | China | Non-Detect | 1 | 0 |
| 1117509 | 2020 | 1/17/2020 | SOY BASE FORMULA PRODUCT POWDER FORMULA | Baby Food Products | Baby Formula | DOMESTIC | United States | Trace | 4.99 | |
| 1135777 | 2020 | 1/20/2020 | MIXED SPICE AND SEAONINGS WITHOUT SALT NEC | Spices | Other Spices | IMPORT | Canada | Trace | 5 | |
| 1113686 | 2020 | 1/21/2020 | PISTACHIO IN SHELL | Nuts and Edible Seeds | Pistachios | DOMESTIC | United States | Non-Detect | 1 | 0 |
| 1134481 | 2020 | 1/22/2020 | RICE CULTIVATED WHOLE GRAIN | Grains and Grain Products | Rice | DOMESTIC | United States | Non-Detect | 0.233 | 0 |
| 1135882 | 2020 | 1/22/2020 | GLUTEN WHEAT | Grains and Grain Products | Wheat | IMPORT | Austria | Non-Detect | 0.233 | 0 |
| 1109055 | 2020 | 1/22/2020 | SOY BASE FORMULA PRODUCT READY TO FEED | Baby Food Products | Baby Formula | DOMESTIC | United States | Non-Detect | 1 | 0 |
| 1086737 | 2020 | 1/22/2020 | OAT FLOUR | Grains and Grain Products | Oats | DOMESTIC | Unknown | Trace | 5 | |
| 1101741 | 2020 | 1/23/2020 | RICE CULTIVATED WHOLE GRAIN | Grains and Grain Products | Rice | DOMESTIC | United States | Non-Detect | 0.233 | 0 |
| 1120928 | 2020 | 1/23/2020 | RICE CULTIVATED WHOLE GRAIN | Grains and Grain Products | Rice | DOMESTIC | United States | Non-Detect | 0.233 | 0 |
| 1134584 | 2020 | 1/23/2020 | WHEAT FLOUR BROMATED | Grains and Grain Products | Wheat | DOMESTIC | United States | Non-Detect | 0.233 | 0 |
| 1135816 | 2020 | 1/23/2020 | RICE CULTIVATED WHOLE GRAIN | Grains and Grain Products | Rice | DOMESTIC | United States | Non-Detect | 0.233 | 0 |
| 1136201 | 2020 | 1/23/2020 | WHEAT FLOUR NEC | Grains and Grain Products | Wheat | IMPORT | Hong Kong SAR | Non-Detect | 1 | 0 |
| 1136338 | 2020 | 1/23/2020 | COFFEE BEANS | Non-Juice Beverages | Coffee | IMPORT | Colombia | Non-Detect | 1 | 0 |
| 1136344 | 2020 | 1/23/2020 | BLACK BEAN DRIED OR PASTE | Beans and Legumes | Black Beans | IMPORT | China | Non-Detect | 1 | 0 |
| 1136346 | 2020 | 1/23/2020 | KIDNEY BEAN | Beans and Legumes | Other Beans and Legumes | IMPORT | China | Non-Detect | 1 | 0 |
| 1136375 | 2020 | 1/23/2020 | WHEAT MILLED PRODUCT NEC | Grains and Grain Products | Wheat | IMPORT | Mexico | Non-Detect | 1 | 0 |
| 1136394 | 2020 | 1/23/2020 | WHEAT MILLED PRODUCT NEC | Grains and Grain Products | Wheat | IMPORT | Mexico | Non-Detect | 1 | 0 |
| 1136350 | 2020 | 1/23/2020 | BUCKWHEAT FLOUR | Grains and Grain Products | Buckwheat | IMPORT | China | Positive | 1.2 | 2.1 |
| 1136361 | 2020 | 1/24/2020 | RAISINS DRIED OR PASTE | Fruit | Raisins | IMPORT | Chile | Non-Detect | 2.629 | 0 |
| 1120661 | 2020 | 1/27/2020 | FIG DRIED OR PASTE | Fruit | Dried Figs | DOMESTIC | United States | Non-Detect | 1 | 0 |
| 1136083 | 2020 | 1/27/2020 | PISTACHIO IN SHELL | Nuts and Edible Seeds | Pistachios | DOMESTIC | United States | Non-Detect | 1 | 0 |
| 1136085 | 2020 | 1/27/2020 | WHEAT FLOUR NEC | Grains and Grain Products | Wheat | DOMESTIC | United States | Trace | 5 | |
| 1136849 | 2020 | 1/28/2020 | COFFEE BEANS | Non-Juice Beverages | Coffee | IMPORT | Brazil | Non-Detect | 0.233 | 0 |
| 1137068 | 2020 | 1/28/2020 | COFFEE BEANS | Non-Juice Beverages | Coffee | IMPORT | Colombia | Non-Detect | 1 | 0 |
| 1074594 | 2020 | 1/28/2020 | MIXED GRAIN CEREAL BABY | Baby Food Products | Baby Cereals | IMPORT | Chile | Trace | 5 | |
| 1137418 | 2020 | 1/29/2020 | COFFEE BEANS | Non-Juice Beverages | Coffee | IMPORT | Colombia | Non-Detect | 1 | 0 |
| 1137395 | 2020 | 1/30/2020 | COFFEE BEANS | Non-Juice Beverages | Coffee | IMPORT | Brazil | Non-Detect | 2.5 | 0 |
| 1074595 | 2020 | 1/31/2020 | RICE CEREAL BABY | Baby Food Products | Baby Cereals | IMPORT | Chile | Trace | 5 | |
| 1074596 | 2020 | 2/3/2020 | OAT CEREAL BABY | Baby Food Products | Baby Cereals | IMPORT | United States | Non-Detect | 0.233 | 0 |
| 1137535 | 2020 | 2/3/2020 | OATS WHOLE GRAIN | Grains and Grain Products | Oats | IMPORT | Canada | Non-Detect | 1 | 0 |
| 1137582 | 2020 | 2/3/2020 | COFFEE BEANS | Non-Juice Beverages | Coffee | IMPORT | Yemen | Non-Detect | 1 | 0 |
| 1137593 | 2020 | 2/3/2020 | ADZUKI BEANS | Beans and Legumes | Other Beans and Legumes | IMPORT | China | Non-Detect | 1 | 0 |
| 1137750 | 2020 | 2/4/2020 | PISTACHIO IN SHELL | Nuts and Edible Seeds | Pistachios | DOMESTIC | United States | Non-Detect | 2.5 | 0 |
| 1074132 | 2020 | 2/4/2020 | BLACKEYE PEAS DRIED OR PASTE | Beans and Legumes | Blackeye Peas | DOMESTIC | United States | Non-Detect | 1 | 0 |
| 1116950 | 2020 | 2/4/2020 | CORN DEGERMINATED MEAL | Grains and Grain Products | Corn | DOMESTIC | United States | Non-Detect | 1 | 0 |
| 1116951 | 2020 | 2/4/2020 | CORN ENRICHED MEAL | Grains and Grain Products | Corn | DOMESTIC | United States | Non-Detect | 1 | 0 |
| 1131926 | 2020 | 2/4/2020 | GINGER GROUND CRACKED SPICE | Spices | Ginger | IMPORT | China | Trace | 4.99 | |
| 1138068 | 2020 | 2/5/2020 | OAT FLAKES ROLLED OATS PUFFS KRISPIES LOOPS READY TO EAT | Processed Food Products | Breakfast Foods | IMPORT | United States | Non-Detect | 2.5 | 0 |
| 1138173 | 2020 | 2/5/2020 | GINGER GROUND CRACKED SPICE | Spices | Ginger | IMPORT | Pakistan | Non-Detect | 2.5 | 0 |
| 1000508 | 2020 | 2/5/2020 | RYE FLOUR | Grains and Grain Products | Rye | DOMESTIC | United States | Non-Detect | 0.233 | 0 |
| 1074824 | 2020 | 2/5/2020 | BLACK EYE BEANS | Beans and Legumes | Blackeye Peas | DOMESTIC | United States | Non-Detect | 1 | 0 |
| 1074825 | 2020 | 2/5/2020 | PINTO BEAN | Beans and Legumes | Pinto Beans | DOMESTIC | United States | Non-Detect | 1 | 0 |
| 1131899 | 2020 | 2/5/2020 | WHEAT MILLED CRUSHED COARSE GROUND OR CRACKED | Grains and Grain Products | Wheat | DOMESTIC | United States | Non-Detect | 1 | 0 |
| 1137841 | 2020 | 2/5/2020 | COFFEE BEANS | Non-Juice Beverages | Coffee | IMPORT | Honduras | Non-Detect | 1 | 0 |
| 1137855 | 2020 | 2/5/2020 | COFFEE BEANS | Non-Juice Beverages | Coffee | IMPORT | Brazil | Non-Detect | 1 | 0 |
| 1137331 | 2020 | 2/6/2020 | BARLEY WHOLE GRAIN | Grains and Grain Products | Barley | DOMESTIC | United States | Non-Detect | 2.5 | 0 |
| 1138023 | 2020 | 2/6/2020 | MIXED CEREAL FLAKES SHREDDED AND OTHER FORMS READY TO EAT | Processed Food Products | Breakfast Foods | IMPORT | Canada | Non-Detect | 2.5 | 0 |
| 1138133 | 2020 | 2/6/2020 | BLACKEYED PEA | Beans and Legumes | Blackeye Peas | IMPORT | Peru | Non-Detect | 2.5 | 0 |
| 1138017 | 2020 | 2/6/2020 | KIDNEY BEAN | Beans and Legumes | Other Beans and Legumes | IMPORT | Nicaragua | Non-Detect | 0.233 | 0 |

| | | | | | | | | | | |
|---|---|---|---|---|---|---|---|---|---|---|
| 1115755 | 2020 | 2/7/2020 | BUCKWHEAT FLOUR | Grains and Grain Products | Buckwheat | DOMESTIC | United States | Non-Detect | 2.5 | 0 |
| 1138112 | 2020 | 2/7/2020 | OAT FLAKES ROLLED OATS PUFFS KRISPIES LOOPS READY TO EAT | Processed Food Products | Breakfast Foods | IMPORT | Mexico | Non-Detect | 2.5 | 0 |
| 1135270 | 2020 | 2/10/2020 | RAISINS DRIED OR PASTE | Fruit | Raisins | DOMESTIC | United States | Positive | 1 | 2.6 |
| 1124002 | 2020 | 2/11/2020 | COOKIES BABY | Baby Food Products | Other Baby Foods | DOMESTIC | United States | Non-Detect | 1 | 0 |
| 1137333 | 2020 | 2/14/2020 | OATS WHOLE GRAIN | Grains and Grain Products | Oats | IMPORT | Canada | Non-Detect | 2.5 | 0 |
| 1136573 | 2020 | 2/18/2020 | BLACK BEAN DRIED OR PASTE | Beans and Legumes | Black Beans | DOMESTIC | United States | Non-Detect | 2.5 | 0 |
| 1096881 | 2020 | 2/18/2020 | GARBANZO BEAN | Beans and Legumes | Garbanzo Beans | DOMESTIC | United States | Non-Detect | 1 | 0 |
| 1118212 | 2020 | 2/19/2020 | CAPSICUMS CAYENNE CHILI HOT PEPPERS WHOLE SPICE | Spices | Capsicums, including Paprika | DOMESTIC | United States | Non-Detect | 0.233 | 0 |
| 1139634 | 2020 | 2/21/2020 | COFFEE BEANS | Non-Juice Beverages | Coffee | IMPORT | Brazil | Non-Detect | 1 | 0 |
| 1139641 | 2020 | 2/21/2020 | IMITATION CHOCOLATE AND COCOA PRODUCTS NEC | Candy and Chocolate Products | Other Chocolate Products | IMPORT | Canada | Non-Detect | 1 | 0 |
| 1139901 | 2020 | 2/24/2020 | KIDNEY BEAN DRIED OR PASTE | Beans and Legumes | Other Beans and Legumes | IMPORT | Mexico | Non-Detect | 0.233 | 0 |
| 1139973 | 2020 | 2/24/2020 | COFFEE BEANS | Non-Juice Beverages | Coffee | IMPORT | Guatemala | Non-Detect | 12.15 | 0 |
| 1139095 | 2020 | 2/26/2020 | OATS WHOLE GRAIN | Grains and Grain Products | Oats | DOMESTIC | United States | Non-Detect | 0.233 | 0 |
| 1140172 | 2020 | 2/26/2020 | RAISINS DRIED OR PASTE | Fruit | Raisins | DOMESTIC | United States | Non-Detect | 2.629 | 0 |
| 1140214 | 2020 | 2/26/2020 | CRANBERRY BEAN | Beans and Legumes | Other Beans and Legumes | IMPORT | Canada | Non-Detect | 0.233 | 0 |
| 1140353 | 2020 | 2/26/2020 | COFFEE BEANS | Non-Juice Beverages | Coffee | IMPORT | Guatemala | Non-Detect | 12.15 | 0 |
| 1140171 | 2020 | 2/26/2020 | PISTACHIO IN SHELL | Nuts and Edible Seeds | Pistachios | DOMESTIC | United States | Non-Detect | 1 | 0 |
| 1140521 | 2020 | 2/27/2020 | BEANS NEC VEGETABLE | Beans and Legumes | Other Beans and Legumes | IMPORT | Mexico | Non-Detect | 1 | 0 |
| 1140882 | 2020 | 3/3/2020 | BRAN FLAKES SHREDDED AND OTHER FORMS READY TO EAT | Processed Food Products | Breakfast Foods | IMPORT | Mexico | Non-Detect | 1 | 0 |
| 1140898 | 2020 | 3/3/2020 | CORN FLAKES PUFFS KRISPIES LOOPS READY TO EAT | Processed Food Products | Other Corn Products | IMPORT | Mexico | Non-Detect | 1 | 0 |
| 1140900 | 2020 | 3/3/2020 | OAT FLAKES ROLLED OATS PUFFS KRISPIES LOOPS READY TO EAT | Processed Food Products | Breakfast Foods | IMPORT | Mexico | Non-Detect | 1 | 0 |
| 1140925 | 2020 | 3/3/2020 | SEMOLINA WHEAT | Grains and Grain Products | Wheat | IMPORT | India | Non-Detect | 1 | 0 |
| 1138260 | 2020 | 3/4/2020 | BEAN DRIED OR PASTE NEC VEGETABLE | Beans and Legumes | Other Beans and Legumes | DOMESTIC | United States | Non-Detect | 1 | 0 |
| 1141239 | 2020 | 3/5/2020 | COFFEE INSTANT | Non-Juice Beverages | Coffee | IMPORT | Italy | Non-Detect | 2.5 | 0 |
| 1141443 | 2020 | 3/10/2020 | PISTACHIO IN SHELL | Nuts and Edible Seeds | Pistachios | DOMESTIC | United States | Non-Detect | 2.5 | 0 |
| 1141444 | 2020 | 3/10/2020 | RAISINS DRIED OR PASTE | Fruit | Raisins | DOMESTIC | United States | Non-Detect | 2.5 | 0 |
| 1141445 | 2020 | 3/10/2020 | RAISINS DRIED OR PASTE | Fruit | Raisins | DOMESTIC | United States | Non-Detect | 2.5 | 0 |
| 1124368 | 2020 | 3/11/2020 | CORN ENRICHED MEAL | Grains and Grain Products | Corn | DOMESTIC | United States | Non-Detect | 1 | 0 |
| 1141635 | 2020 | 3/11/2020 | CHICKPEA DRIED OR PASTE | Beans and Legumes | Garbanzo Beans | IMPORT | Turkey | Non-Detect | 1 | 0 |
| 1141633 | 2020 | 3/11/2020 | COFFEE BEANS | Non-Juice Beverages | Coffee | IMPORT | Vietnam | Positive | 1 | 1.2 |
| 1141747 | 2020 | 3/12/2020 | CORN PLAIN MEAL | Grains and Grain Products | Corn | IMPORT | Colombia | Non-Detect | 1 | 0 |
| 1141774 | 2020 | 3/12/2020 | BEAN CORN PEA DRIED OR PASTE NEC VEGETABLE | Beans and Legumes | Other Beans and Legumes | IMPORT | Australia | Non-Detect | 1 | 0 |
| 1141775 | 2020 | 3/12/2020 | BEAN CORN PEA DRIED OR PASTE NEC VEGETABLE | Beans and Legumes | Other Beans and Legumes | IMPORT | Burma | Non-Detect | 1 | 0 |
| 1141779 | 2020 | 3/12/2020 | PIGEON PEAS DRIED OR PASTE | Beans and Legumes | Peas | IMPORT | India | Non-Detect | 1 | 0 |
| 1141942 | 2020 | 3/16/2020 | COFFEE BEANS | Non-Juice Beverages | Coffee | IMPORT | Peru | Non-Detect | 2.5 | 0 |
| 1142097 | 2020 | 3/16/2020 | BEAN DRIED OR PASTE NEC VEGETABLE | Beans and Legumes | Other Beans and Legumes | IMPORT | Burma | Non-Detect | 2.5 | 0 |
| 1142113 | 2020 | 3/17/2020 | LENTILS DRIED OR PASTE | Beans and Legumes | Lentils | IMPORT | Canada | Non-Detect | 0.233 | 0 |
| 1142116 | 2020 | 3/17/2020 | LENTILS DRIED OR PASTE | Beans and Legumes | Lentils | IMPORT | Canada | Non-Detect | 0.233 | 0 |
| 1143280 | 2020 | 5/1/2020 | MALT BARLEY | Grains and Grain Products | Barley | IMPORT | United Kingdom | Non-Detect | 2.5 | 0 |

## FY2021 Data

| Sample Number | Fiscal Year | Collection Date | Product Name | Food Category | Food Subcategory | Origin Type | Country of Origin | Non-Detect, Trace, or Positive (>LOQ) | LOQ (ppb) | Amount found (ppb) |
|---|---|---|---|---|---|---|---|---|---|---|
| 1149230 | 2021 | 10/7/2020 | COFFEE BEANS | Non-Juice Beverages | Coffee | IMPORT | Honduras | Non-Detect | 2.5 | 0 |
| 1149717 | 2021 | 10/18/2020 | BLACKEYE PEAS DRIED OR PASTE | Beans and Legumes | Blackeye Peas | IMPORT | Brazil | Non-Detect | 1 | 0 |
| 1135760 | 2021 | 10/19/2020 | PISTACHIO IN SHELL | Nuts and Edible Seeds | Pistachios | DOMESTIC | United States | Non-Detect | 1 | 0 |
| 1134340 | 2021 | 10/19/2020 | RAISINS DRIED OR PASTE | Fruit | Raisins | DOMESTIC | United States | Positive | 1 | 1.7 |
| 1135761 | 2021 | 10/19/2020 | RAISINS DRIED GRAPES BERRY | Fruit | Raisins | DOMESTIC | United States | Positive | 1 | 1.1 |
| 1149412 | 2021 | 10/20/2020 | OATS WHOLE GRAIN | Grains and Grain Products | Oats | DOMESTIC | United States | Non-Detect | 2.5 | 0 |
| 1150165 | 2021 | 10/26/2020 | MOONG DAL INDIAN DRIED HULLED SPLIT GREEN GRAM BEANS OR MUNG BEANS YELLOW AND USED IN SOUPS | Beans and Legumes | Other Beans and Legumes | IMPORT | Thailand | Non-Detect | 2.5 | 0 |
| 1130978 | 2021 | 10/28/2020 | BUCKWHEAT WHOLE GRAIN | Grains and Grain Products | Buckwheat | IMPORT | Russia | Non-Detect | 2.5 | 0 |
| 1130979 | 2021 | 10/28/2020 | OAT FLOUR | Grains and Grain Products | Oats | DOMESTIC | United States | Non-Detect | 2.5 | 0 |
| 1130980 | 2021 | 10/28/2020 | RAISINS DRIED OR PASTE | Fruit | Raisins | DOMESTIC | United States | Non-Detect | 2.5 | 0 |
| 1151107 | 2021 | 11/6/2020 | COFFEE BEANS | Non-Juice Beverages | Coffee | IMPORT | Colombia | Non-Detect | 1 | 0 |
| 1151329 | 2021 | 11/11/2020 | COFFEE BEANS | Non-Juice Beverages | Coffee | IMPORT | Colombia | Non-Detect | 2.5 | 0 |
| 1151332 | 2021 | 11/11/2020 | COFFEE BEANS | Non-Juice Beverages | Coffee | IMPORT | Indonesia | Non-Detect | 2.5 | 0 |
| 1151575 | 2021 | 11/13/2020 | CEREAL NEC BABY | Baby Food Products | Baby Cereals | IMPORT | Mexico | Non-Detect | 2.5 | 0 |
| 1151583 | 2021 | 11/13/2020 | CEREAL NEC BABY | Baby Food Products | Baby Cereals | IMPORT | Mexico | Non-Detect | 2.5 | 0 |
| 1152093 | 2021 | 11/24/2020 | COFFEE NEC | Non-Juice Beverages | Coffee | IMPORT | Jamaica | Non-Detect | 2.5 | 0 |
| 1152094 | 2021 | 11/24/2020 | WHEAT FLOUR ENRICHED ALL PURPOSE | Grains and Grain Products | Wheat | IMPORT | Guatemala | Non-Detect | 1 | 0 |
| 1152318 | 2021 | 12/2/2020 | TEMPURA KO JAPANESE LOW GLUTEN WHEAT FLOUR USED TO MAKE TEMPURA BATTER | Grains and Grain Products | Wheat | IMPORT | Italy | Non-Detect | 2.5 | 0 |
| 1152467 | 2021 | 12/3/2020 | COFFEE BEANS | Non-Juice Beverages | Coffee | IMPORT | Colombia | Non-Detect | 2.5 | 0 |
| 1152809 | 2021 | 12/14/2020 | RAISINS DRIED OR PASTE | Fruit | Raisins | IMPORT | Afghanistan | Non-Detect | 2.5 | 0 |
| 1153038 | 2021 | 12/20/2020 | NUTMEG WHOLE SPICE | Spices | Nutmeg | IMPORT | Grenada | Non-Detect | 2.5 | 0 |
| 1153575 | 2021 | 1/14/2021 | SEMOLINA WHEAT | Grains and Grain Products | Wheat | IMPORT | Russia | Non-Detect | 2.5 | 0 |
| 1153576 | 2021 | 1/14/2021 | WHEAT WHOLE GRAIN | Grains and Grain Products | Wheat | IMPORT | Russia | Non-Detect | 2.5 | 0 |
| 1153847 | 2021 | 1/14/2021 | CEREAL NEC BABY | Baby Food Products | Baby Cereals | IMPORT | Germany | Non-Detect | 1 | 0 |
| 1153856 | 2021 | 1/14/2021 | CEREAL NEC BABY | Baby Food Products | Baby Cereals | IMPORT | Switzerland | Non-Detect | 1 | 0 |
| 1153858 | 2021 | 1/14/2021 | MIXED FRUIT BABY | Baby Food Products | Other Baby Foods | IMPORT | Switzerland | Non-Detect | 1 | 0 |
| 1153738 | 2021 | 1/21/2021 | COFFEE BEANS | Non-Juice Beverages | Coffee | IMPORT | Colombia | Non-Detect | 2.5 | 0 |
| 1153801 | 2021 | 1/21/2021 | MALT FLOUR | Grains and Grain Products | Other Grains | IMPORT | France | Non-Detect | 2.5 | 0 |
| 1153982 | 2021 | 1/27/2021 | COFFEE BEANS | Non-Juice Beverages | Coffee | IMPORT | Colombia | Non-Detect | 2.5 | 0 |
| 1154035 | 2021 | 1/28/2021 | BUCKWHEAT WHOLE GRAIN | Grains and Grain Products | Buckwheat | IMPORT | Belarus | Non-Detect | 2.5 | 0 |
| 1154105 | 2021 | 2/1/2021 | RAISINS DRIED GRAPES BERRY | Fruit | Raisins | IMPORT | Pakistan | Non-Detect | 2.5 | 0 |
| 1137931 | 2021 | 2/2/2021 | RAISINS DRIED OR PASTE | Fruit | Raisins | DOMESTIC | United States | Non-Detect | 1 | 0 |
| 1155005 | 2021 | 2/11/2021 | AZUFRADO BEAN DRIED OR PASTE | Beans and Legumes | Other Beans and Legumes | IMPORT | Mexico | Non-Detect | 1 | 0 |
| 1155083 | 2021 | 2/12/2021 | MALT BARLEY | Grains and Grain Products | Barley | IMPORT | Netherlands | Non-Detect | 2.5 | 0 |
| 1155355 | 2021 | 2/17/2021 | COFFEE BEANS | Non-Juice Beverages | Coffee | IMPORT | Honduras | Non-Detect | 2.5 | 0 |
| 1094531 | 2021 | 2/18/2021 | BUCKWHEAT WHOLE GRAIN | Grains and Grain Products | Buckwheat | DOMESTIC | United States | Non-Detect | 2.5 | 0 |
| 1094532 | 2021 | 2/18/2021 | BUCKWHEAT WHOLE GRAIN | Grains and Grain Products | Buckwheat | DOMESTIC | United States | Non-Detect | 2.5 | 0 |
| 1110886 | 2021 | 2/18/2021 | WHEAT FLOUR ENRICHED ALL PURPOSE | Grains and Grain Products | Wheat | DOMESTIC | United States | Non-Detect | 2.5 | 0 |
| 1156033 | 2021 | 2/25/2021 | RAISINS DRIED OR PASTE | Fruit | Raisins | IMPORT | United States | Non-Detect | 2.5 | 0 |
| 1130981 | 2021 | 2/26/2021 | BARLEY WHOLE GRAIN | Grains and Grain Products | Barley | DOMESTIC | United States | Non-Detect | 1 | 0 |
| 1130982 | 2021 | 2/26/2021 | CORN PLAIN MEAL | Grains and Grain Products | Corn | DOMESTIC | Unknown | Non-Detect | 1 | 0 |
| 1156560 | 2021 | 3/3/2021 | RAISINS DRIED OR PASTE | Fruit | Raisins | IMPORT | Canada | Non-Detect | 2.5 | 0 |
| 1131272 | 2021 | 3/4/2021 | CREAM WHEAT QUICK OR INSTANT COOKING | Processed Food Products | Breakfast Foods | DOMESTIC | United States | Non-Detect | 1 | 0 |
| 1156314 | 2021 | 3/4/2021 | CORN HOMINY GRITS | Grains and Grain Products | Corn | DOMESTIC | United States | Non-Detect | 1 | 0 |
| 1157833 | 2021 | 3/16/2021 | ORIENTAL NOODLES FLAVORED WITH SHRIMP CHICKEN BEEF LOBSTER CRAB PLAIN ETC | Processed Food Products | Pasta | IMPORT | Japan | Non-Detect | 0.233 | 0 |
| 1157852 | 2021 | 3/17/2021 | FIG DRIED OR PASTE | Fruit | Dried Figs | IMPORT | Turkmenistan | Non-Detect | 2.5 | 0 |
| 1154075 | 2021 | 3/17/2021 | CORN FLAKES PUFFS KRISPIES LOOPS READY TO EAT | Processed Food Products | Other Corn Products | DOMESTIC | United States | Non-Detect | 1 | 0 |
| 1158045 | 2021 | 3/18/2021 | COFFEE BEANS | Non-Juice Beverages | Coffee | IMPORT | Tanzania, United Republic Of | Non-Detect | 1 | 0 |
| 1132167 | 2021 | 3/24/2021 | SOY BASE FORMULA PRODUCT POWDER FORMULA | Baby Food Products | Baby Formula | DOMESTIC | United States | Non-Detect | 2.5 | 0 |
| 1158956 | 2021 | 3/24/2021 | NAVY WHITE BEANS BAKED BEANS PORK BEANS DRIED OR PASTE | Beans and Legumes | Other Beans and Legumes | IMPORT | China | Non-Detect | 2.5 | 0 |
| 1158703 | 2021 | 3/26/2021 | CHANA DAL DRIED OR PASTE | Beans and Legumes | Garbanzo Beans | IMPORT | India | Non-Detect | 0.233 | 0 |
| 1158741 | 2021 | 3/29/2021 | COFFEE BEANS | Non-Juice Beverages | Coffee | IMPORT | Tanzania, United Republic Of | Non-Detect | 2.5 | 0 |
| 1158953 | 2021 | 3/30/2021 | COFFEE BEANS | Non-Juice Beverages | Coffee | IMPORT | Burundi | Non-Detect | 1 | 0 |
| 1151636 | 2021 | 4/1/2021 | PISTACHIO IN SHELL | Nuts and Edible Seeds | Pistachios | DOMESTIC | United States | Non-Detect | 1 | 0 |
| 1159714 | 2021 | 4/12/2021 | RAISINS DRIED GRAPES BERRY | Fruit | Raisins | DOMESTIC | United States | Positive | 1 | 1.8 |
| 1160252 | 2021 | 4/15/2021 | COFFEE BEANS | Non-Juice Beverages | Coffee | IMPORT | Colombia | Non-Detect | 1 | 0 |
| 1161202 | 2021 | 4/29/2021 | COFFEE BEANS | Non-Juice Beverages | Coffee | IMPORT | Brazil | Non-Detect | 12.15 | 0 |
| 1154448 | 2021 | 5/4/2021 | OATS WHOLE GRAIN | Grains and Grain Products | Oats | IMPORT | United States | Non-Detect | 2.5 | 0 |
| 1150237 | 2021 | 5/12/2021 | OATS WHOLE GRAIN | Grains and Grain Products | Oats | DOMESTIC | United States | Non-Detect | 1 | 0 |
| 1163389 | 2021 | 6/2/2021 | COFFEE BEANS | Non-Juice Beverages | Coffee | IMPORT | Guatemala | Non-Detect | 2.5 | 0 |
| 1163795 | 2021 | 6/8/2021 | WHEAT FLOUR GLUTEN | Grains and Grain Products | Wheat | IMPORT | United States | Positive | 1 | 2.6 |
| 1139969 | 2021 | 6/9/2021 | RYE FLOUR | Grains and Grain Products | Rye | DOMESTIC | United States | Non-Detect | 2.5 | 0 |
| 1109816 | 2021 | 6/10/2021 | PISTACHIO IN SHELL | Nuts and Edible Seeds | Pistachios | DOMESTIC | United States | Non-Detect | 1 | 0 |
| 1145118 | 2021 | 6/10/2021 | PISTACHIO IN SHELL | Nuts and Edible Seeds | Pistachios | DOMESTIC | United States | Non-Detect | 1 | 0 |
| 1098275 | 2021 | 6/15/2021 | CORN FLAKES PUFFS KRISPIES LOOPS READY TO EAT | Processed Food Products | Other Corn Products | DOMESTIC | United States | Non-Detect | 1 | 0 |
| 1164351 | 2021 | 6/17/2021 | COFFEE BEANS | Non-Juice Beverages | Coffee | IMPORT | Guatemala | Non-Detect | 1 | 0 |
| 1165019 | 2021 | 6/28/2021 | RAISINS DRIED GRAPES BERRY | Fruit | Raisins | IMPORT | Pakistan | Positive | 1.16 | 1.2 |
| 1165303 | 2021 | 6/30/2021 | RAISINS DRIED GRAPES BERRY | Fruit | Raisins | IMPORT | South Africa | Non-Detect | 2.5 | 0 |
| 1163187 | 2021 | 6/30/2021 | GINGER GROUND CRACKED SPICE | Spices | Ginger | IMPORT | India | Non-Detect | 1 | 0 |
| 1165312 | 2021 | 7/1/2021 | COFFEE BEANS DECAFFEINATED | Non-Juice Beverages | Coffee | IMPORT | Mexico | Non-Detect | 2.5 | 0 |
| 1165314 | 2021 | 7/1/2021 | RAISINS DRIED OR PASTE | Fruit | Raisins | IMPORT | South Africa | Non-Detect | 2.5 | 0 |
| 1165869 | 2021 | 7/8/2021 | QUINOA SEED EDIBLE SEED | Nuts and Edible Seeds | Other Nuts and Edible Seeds | IMPORT | United States | Non-Detect | 1 | 0 |
| 1166444 | 2021 | 7/14/2021 | WHEAT CEREAL BABY | Baby Food Products | Baby Cereals | IMPORT | Lithuania | Non-Detect | 2.5 | 0 |
| 1166288 | 2021 | 7/22/2021 | OAT CEREAL BABY | Baby Food Products | Baby Cereals | DOMESTIC | United States | Non-Detect | 2.5 | 0 |
| 1167099 | 2021 | 7/22/2021 | COFFEE BEANS | Non-Juice Beverages | Coffee | IMPORT | Nicaragua | Non-Detect | 1 | 0 |
| 1167357 | 2021 | 7/23/2021 | CAPSICUMS CAYENNE CHILI HOT PEPPERS GROUND CRACKED SPICE | Spices | Capsicums, including Paprika | IMPORT | India | Positive | 2.5 | 49.72 |
| 1144634 | 2021 | 7/26/2021 | PISTACHIO IN SHELL | Nuts and Edible Seeds | Pistachios | DOMESTIC | United States | Non-Detect | 2.5 | 0 |
| 1167409 | 2021 | 7/26/2021 | RICE CEREAL BABY | Baby Food Products | Baby Cereals | IMPORT | Serbia | Non-Detect | 2.5 | 0 |
| 1167741 | 2021 | 7/30/2021 | CORN PLAIN MEAL | Grains and Grain Products | Corn | IMPORT | Mexico | Non-Detect | 0.233 | 0 |
| 1167839 | 2021 | 8/2/2021 | MALT BARLEY | Grains and Grain Products | Barley | IMPORT | France | Non-Detect | 2.5 | 0 |
| 1168340 | 2021 | 8/2/2021 | SOYBEANS WHOLE GRAIN | Beans and Legumes | Soybeans | IMPORT | Canada | Non-Detect | 2.5 | 0 |
| 1167940 | 2021 | 8/2/2021 | BEAN CORN PEA DRIED OR PASTE NEC VEGETABLE | Beans and Legumes | Other Beans and Legumes | IMPORT | Mexico | Non-Detect | 1 | 0 |
| 1168463 | 2021 | 8/5/2021 | COFFEE BEANS | Non-Juice Beverages | Coffee | IMPORT | Guatemala | Non-Detect | 2.5 | 0 |
| 1166289 | 2021 | 8/6/2021 | SOY BASE FORMULA PRODUCT LIQUID CONCENTRATE | Baby Food Products | Baby Formula | DOMESTIC | United States | Non-Detect | 2.5 | 0 |
| 1168831 | 2021 | 8/10/2021 | MALT BARLEY | Grains and Grain Products | Barley | IMPORT | Belgium | Non-Detect | 2.5 | 0 |
| 1168875 | 2021 | 8/10/2021 | RED BEAN | Beans and Legumes | Other Beans and Legumes | IMPORT | El Salvador | Trace | 5 | |
| 1169107 | 2021 | 8/13/2021 | BEAN DRIED OR PASTE NEC VEGETABLE | Beans and Legumes | Other Beans and Legumes | IMPORT | Mexico | Non-Detect | 2.5 | 0 |
| 1162593 | 2021 | 8/16/2021 | OAT CEREAL BABY | Baby Food Products | Baby Cereals | DOMESTIC | United States | Non-Detect | 2.5 | 0 |

| | | | | | | | | | | |
|---|---|---|---|---|---|---|---|---|---|---|
| 1162602 | 2021 | 8/16/2021 | RICE CEREAL BABY | Baby Food Products | Baby Cereals | DOMESTIC | United States | Non-Detect | 2.5 | 0 |
| 1162603 | 2021 | 8/16/2021 | WHEAT CEREAL BABY | Baby Food Products | Baby Cereals | DOMESTIC | United States | Non-Detect | 2.5 | 0 |
| 1162877 | 2021 | 8/17/2021 | FIG DRIED OR PASTE | Fruit | Dried Figs | DOMESTIC | Unknown | Non-Detect | 2.5 | 0 |
| 1169433 | 2021 | 8/18/2021 | AZUFRADO BEAN | Beans and Legumes | Other Beans and Legumes | IMPORT | Mexico | Non-Detect | 1 | 0 |
| 1169404 | 2021 | 8/18/2021 | CAPSICUMS CAYENNE CHILI HOT PEPPERS WHOLE SPICE | Spices | Capsicums, including Paprika | DOMESTIC | United States | Positive | 2.5 | 9.24 |
| 1169523 | 2021 | 8/19/2021 | COFFEE BEANS | Non-Juice Beverages | Coffee | IMPORT | Uganda | Non-Detect | 2.5 | 0 |
| 1169754 | 2021 | 8/23/2021 | WHEAT FLOUR GLUTEN | Grains and Grain Products | Wheat | IMPORT | China | Non-Detect | 0.233 | 0 |
| 1147289 | 2021 | 8/25/2021 | RICE CEREAL BABY | Baby Food Products | Baby Cereals | DOMESTIC | United States | Non-Detect | 0.233 | 0 |
| 1166291 | 2021 | 8/26/2021 | GINGER GROUND CRACKED SPICE | Spices | Ginger | DOMESTIC | United States | Non-Detect | 2.5 | 0 |
| 1170397 | 2021 | 8/30/2021 | KOLA NUT RAW MATERIAL FOR EXTRACTS AND FLAVORS | Spices | Other Spices | IMPORT | Nigeria | Non-Detect | 2.5 | 0 |
| 1170501 | 2021 | 8/31/2021 | COFFEE BEANS | Non-Juice Beverages | Coffee | IMPORT | Nicaragua | Non-Detect | 1 | 0 |
| 1170694 | 2021 | 8/31/2021 | LENTILS DRIED OR PASTE | Beans and Legumes | Lentils | IMPORT | China | Non-Detect | 1 | 0 |
| 1162950 | 2021 | 9/8/2021 | SOY BASE FORMULA PRODUCT READY TO FEED | Baby Food Products | Baby Formula | DOMESTIC | United States | Non-Detect | 2.5 | 0 |
| 1171337 | 2021 | 9/10/2021 | STARCH PRODUCTS NEC | Grains and Grain Products | Other Grains | IMPORT | Belgium | Non-Detect | 1 | 0 |
| 1167313 | 2021 | 9/16/2021 | BUCKWHEAT WHOLE GRAIN | Grains and Grain Products | Buckwheat | DOMESTIC | United States | Non-Detect | 2.5 | 0 |
| 1164907 | 2021 | 9/21/2021 | RYE FLOUR | Grains and Grain Products | Rye | DOMESTIC | United States | Non-Detect | 2.5 | 0 |
| 1147293 | 2021 | 9/22/2021 | CAPSICUMS CAYENNE CHILI HOT PEPPERS GROUND CRACKED SPICE | Spices | Capsicums, including Paprika | DOMESTIC | United States | Positive | 5 | 9.55 |
| 1172296 | 2021 | 9/23/2021 | RICE CEREAL BABY | Baby Food Products | Baby Cereals | DOMESTIC | United States | Non-Detect | 1 | 0 |
| 1172440 | 2021 | 9/23/2021 | WHEAT FLOUR, N.E.C. | Grains and Grain Products | Wheat | IMPORT | India | Non-Detect | 1 | 0 |
| 1172464 | 2021 | 9/23/2021 | WHEAT, MILLED PRODUCT, N.E.C. | Grains and Grain Products | Wheat | IMPORT | India | Non-Detect | 1 | 0 |
| 1171654 | 2021 | 9/23/2021 | WHEAT BRAN (HUMAN USE) | Grains and Grain Products | Wheat | DOMESTIC | United States | Trace | 1 | |
| 1155982 | 2021 | 9/28/2021 | BUCKWHEAT FLOUR | Grains and Grain Products | Buckwheat | DOMESTIC | United States | Trace | 5 | |
| 1172771 | 2021 | 9/30/2021 | WHEAT BRAN (HUMAN USE) | Grains and Grain Products | Wheat | IMPORT | Russia | Non-Detect | 1 | 0 |

**FY2022 Data**

| Sample Number | Fiscal Year | Collection Date | Product Name | Food Category | Food Subcategory | Origin Type | Country of Origin | Non-Detect, Trace, or Positive (>LOQ) | LOQ (ppb) | Amount found (ppb) |
|---|---|---|---|---|---|---|---|---|---|---|
| 1172902 | 2022 | 10/4/2021 | GRAIN, WHOLE, N.E.C. | Grains and Grain Products | Other Grains | IMPORT | Spain | Non-Detect | 0.233 | 0 |
| 1164288 | 2022 | 10/4/2021 | RAISINS (DRIED GRAPES) (BERRY) | Fruit | Raisins | DOMESTIC | United States | Non-Detect | 1 | 0 |
| 1173519 | 2022 | 10/13/2021 | RAISINS (DRIED GRAPES) (BERRY) | Fruit | Raisins | DOMESTIC | United States | Trace | 5 | |
| 1169272 | 2022 | 10/19/2021 | RAISINS (DRIED GRAPES) (BERRY) | Fruit | Raisins | DOMESTIC | United States | Non-Detect | 1 | 0 |
| 1174173 | 2022 | 10/20/2021 | COFFEE, BEANS | Non-Juice Beverages | Coffee | IMPORT | Mexico | Non-Detect | 2.5 | 0 |
| 1174150 | 2022 | 10/20/2021 | COFFEE, BEANS | Non-Juice Beverages | Coffee | IMPORT | Colombia | Non-Detect | 12.15 | 0 |
| 1168483 | 2022 | 10/25/2021 | OAT CEREAL (BABY) | Baby Food Products | Baby Cereals | DOMESTIC | United States | Non-Detect | 2.5 | 0 |
| 1170136 | 2022 | 10/27/2021 | OATS, WHOLE GRAIN | Grains and Grain Products | Oats | DOMESTIC | United States | Non-Detect | 2.5 | 0 |
| 1175090 | 2022 | 11/2/2021 | CREAM WHEAT, QUICK OR INSTANT COOKING | Processed Food Products | Breakfast Foods | IMPORT | Guatemala | Non-Detect | 2.5 | 0 |
| 1175523 | 2022 | 11/2/2021 | RAISINS, DRIED OR PASTE | Fruit | Raisins | IMPORT | South Africa | Trace | 5.01 | |
| 1164661 | 2022 | 11/3/2021 | RAISINS (DRIED GRAPES) (BERRY) | Fruit | Raisins | DOMESTIC | United States | Non-Detect | 1 | 0 |
| 1175838 | 2022 | 11/4/2021 | COCOA DAIRY MIX | Candy and Chocolate Products | Other Chocolate Products | IMPORT | United States | Non-Detect | 2.5 | 0 |
| 1138202 | 2022 | 11/4/2021 | SOY BASE FORMULA PRODUCT, POWDER FORMULA | Baby Food Products | Baby Formula | DOMESTIC | United States | Non-Detect | 0.233 | 0 |
| 1175408 | 2022 | 11/4/2021 | RAISINS (DRIED GRAPES) (BERRY) | Fruit | Raisins | IMPORT | South Africa | Trace | 4.99 | |
| 1175457 | 2022 | 11/7/2021 | COFFEE, BEANS | Non-Juice Beverages | Coffee | IMPORT | Brazil | Non-Detect | 12.15 | 0 |
| 1175622 | 2022 | 11/8/2021 | COFFEE, BEANS | Non-Juice Beverages | Coffee | IMPORT | Guatemala | Non-Detect | 2.5 | 0 |
| 1170429 | 2022 | 11/9/2021 | BUCKWHEAT FLOUR | Grains and Grain Products | Buckwheat | DOMESTIC | United States | Non-Detect | 0.233 | 0 |
| 1176030 | 2022 | 11/15/2021 | COFFEE, BEANS | Non-Juice Beverages | Coffee | IMPORT | Brazil | Non-Detect | 2.5 | 0 |
| 1176136 | 2022 | 11/16/2021 | MUNG BEAN, DRIED OR PASTE | Beans and Legumes | Other Beans and Legumes | IMPORT | Thailand | Non-Detect | 2.5 | 0 |
| 1176191 | 2022 | 11/16/2021 | MUNG BEAN | Beans and Legumes | Other Beans and Legumes | IMPORT | Thailand | Non-Detect | 2.5 | 0 |
| 1158108 | 2022 | 11/16/2021 | RYE FLOUR | Grains and Grain Products | Rye | DOMESTIC | United States | Non-Detect | 1 | 0 |
| 1176360 | 2022 | 11/17/2021 | COFFEE, BEANS | Non-Juice Beverages | Coffee | IMPORT | Ethiopia | Non-Detect | 1 | 0 |
| 1176418 | 2022 | 11/17/2021 | FLAVORED OR PARTY CRACKERS | Processed Food Products | Other Bakery Products | IMPORT | Canada | Non-Detect | 1 | 0 |
| 1176602 | 2022 | 11/22/2021 | BARLEY, WHOLE GRAIN | Grains and Grain Products | Barley | DOMESTIC | United States | Non-Detect | 2.5 | 0 |
| 1170431 | 2022 | 11/22/2021 | BUCKWHEAT FLOUR | Grains and Grain Products | Buckwheat | DOMESTIC | United States | Trace | 2.5 | |
| 1171091 | 2022 | 11/22/2021 | OAT FLOUR | Grains and Grain Products | Oats | DOMESTIC | United States | Trace | 2.5 | |
| 1171092 | 2022 | 11/23/2021 | CORN, PLAIN MEAL | Grains and Grain Products | Corn | DOMESTIC | United States | Non-Detect | 2.5 | 0 |
| 1146352 | 2022 | 11/30/2021 | BEAN, DRIED OR PASTE, N.E.C. (VEGETABLE) | Beans and Legumes | Other Beans and Legumes | DOMESTIC | United States | Non-Detect | 2.5 | 0 |
| 1175493 | 2022 | 11/30/2021 | OAT CEREAL (BABY) | Baby Food Products | Baby Cereals | DOMESTIC | Unknown | Non-Detect | 0.233 | 0 |
| 1176844 | 2022 | 11/30/2021 | CORN, DEGERMINATED MEAL | Grains and Grain Products | Corn | DOMESTIC | United States | Non-Detect | 1 | 0 |
| 1177044 | 2022 | 12/1/2021 | COFFEE, BEANS | Non-Juice Beverages | Coffee | IMPORT | Colombia | Non-Detect | 2.5 | 0 |
| 1177118 | 2022 | 12/2/2021 | WHEAT FLOUR, PLAIN | Grains and Grain Products | Wheat | IMPORT | Guatemala | Non-Detect | 2.5 | 0 |
| 1177213 | 2022 | 12/2/2021 | COFFEE, BEANS | Non-Juice Beverages | Coffee | IMPORT | Guatemala | Non-Detect | 2.5 | 0 |
| 1177564 | 2022 | 12/3/2021 | AZUFRADO BEAN, BEAN AND PEA SPROUTS | Beans and Legumes | Other Beans and Legumes | IMPORT | Canada | Non-Detect | 1 | 0 |
| 1159741 | 2022 | 12/7/2021 | PISTACHIO, IN SHELL | Nuts and Edible Seeds | Pistachios | DOMESTIC | United States | Non-Detect | 1 | 0 |
| 1177501 | 2022 | 12/8/2021 | COFFEE, BEANS | Non-Juice Beverages | Coffee | IMPORT | Italy | Non-Detect | 2.5 | 0 |
| 1175013 | 2022 | 12/9/2021 | PISTACHIO, IN SHELL | Nuts and Edible Seeds | Pistachios | DOMESTIC | United States | Non-Detect | 1 | 0 |
| 1177666 | 2022 | 12/10/2021 | OATMEAL, REGULAR, FRUIT OR SPICE ADDED, QUICK OR INSTANT COOKING | Processed Food Products | Breakfast Foods | IMPORT | Ireland | Non-Detect | 1 | 0 |
| 1178197 | 2022 | 12/14/2021 | RAISINS, DRIED OR PASTE | Fruit | Raisins | IMPORT | Afghanistan | Non-Detect | 2.629 | 0 |
| 1178025 | 2022 | 12/14/2021 | COFFEE, BEANS | Non-Juice Beverages | Coffee | IMPORT | Guatemala | Non-Detect | 1 | 0 |
| 1178198 | 2022 | 12/14/2021 | RAISINS, DRIED OR PASTE | Fruit | Raisins | IMPORT | Afghanistan | Trace | 5 | |
| 1178158 | 2022 | 12/15/2021 | COFFEE, BEANS | Non-Juice Beverages | Coffee | IMPORT | Brazil | Trace | 5 | |
| 1173975 | 2022 | 12/16/2021 | PISTACHIO, IN SHELL | Nuts and Edible Seeds | Pistachios | DOMESTIC | United States | Non-Detect | 2.38 | 0 |
| 1176847 | 2022 | 12/16/2021 | BUCKWHEAT FLOUR | Grains and Grain Products | Buckwheat | DOMESTIC | United States | Trace | 5 | |
| 1178313 | 2022 | 12/17/2021 | COFFEE, BEANS | Non-Juice Beverages | Coffee | IMPORT | Brazil | Non-Detect | 2.5 | 0 |
| 1178388 | 2022 | 12/20/2021 | KIDNEY BEAN, DRIED OR PASTE | Beans and Legumes | Other Beans and Legumes | IMPORT | Honduras | Non-Detect | 2.5 | 0 |
| 1178551 | 2022 | 12/21/2021 | COFFEE, BEANS | Non-Juice Beverages | Coffee | IMPORT | Guatemala | Non-Detect | 1 | 0 |
| 1171732 | 2022 | 12/27/2021 | FIG, DRIED OR PASTE | Fruit | Dried Figs | IMPORT | Turkey | Non-Detect | 2.5 | 0 |
| 1179400 | 2022 | 12/29/2021 | FRIED SNACK FOODS, N.E.C. | Processed Food Products | Other Snack Foods | IMPORT | India | Trace | 5 | |
| 1179736 | 2022 | 1/7/2022 | LENTILS, DRIED OR PASTE | Beans and Legumes | Lentils | IMPORT | Turkey | Non-Detect | 1 | 0 |
| 1180064 | 2022 | 1/13/2022 | BLACK BEAN, DRIED OR PASTE | Beans and Legumes | Black Beans | IMPORT | Thailand | Non-Detect | 1 | 0 |
| 1180245 | 2022 | 1/19/2022 | COFFEE, GROUND | Non-Juice Beverages | Coffee | IMPORT | United States | Non-Detect | 2.5 | 0 |
| 1180334 | 2022 | 1/20/2022 | COFFEE, BEANS | Non-Juice Beverages | Coffee | IMPORT | Colombia | Non-Detect | 2.5 | 0 |
| 1180436 | 2022 | 1/20/2022 | COFFEE, BEANS | Non-Juice Beverages | Coffee | IMPORT | Ecuador | Non-Detect | 2.5 | 0 |
| 1180437 | 2022 | 1/20/2022 | COFFEE, BEANS | Non-Juice Beverages | Coffee | IMPORT | Rwanda | Non-Detect | 2.5 | 0 |
| 1180668 | 2022 | 1/25/2022 | COFFEE, BEANS | Non-Juice Beverages | Coffee | IMPORT | Colombia | Non-Detect | 1 | 0 |
| 1181043 | 2022 | 1/27/2022 | RAISINS, DRIED OR PASTE | Fruit | Raisins | IMPORT | Afghanistan | Non-Detect | 2.5 | 0 |
| 1181044 | 2022 | 1/27/2022 | RAISINS, DRIED OR PASTE | Fruit | Raisins | IMPORT | Afghanistan | Non-Detect | 2.5 | 0 |
| 1181045 | 2022 | 1/27/2022 | RAISINS, DRIED OR PASTE | Fruit | Raisins | IMPORT | Afghanistan | Non-Detect | 2.5 | 0 |
| 1181046 | 2022 | 1/27/2022 | OTHER FRUIT AND FRUIT PRODUCTS, DRIED AND PASTE, N.E.C. | Fruit | Other Dried Fruits | IMPORT | Afghanistan | Positive | 2.5 | 42.44 |
| 1181188 | 2022 | 2/1/2022 | COFFEE, BEANS | Non-Juice Beverages | Coffee | IMPORT | Nicaragua | Non-Detect | 2.5 | 0 |
| 1181189 | 2022 | 2/1/2022 | COFFEE, BEANS | Non-Juice Beverages | Coffee | IMPORT | Nicaragua | Non-Detect | 2.5 | 0 |
| 1181179 | 2022 | 2/1/2022 | COFFEE, BEANS | Non-Juice Beverages | Coffee | IMPORT | Brazil | Non-Detect | 12.15 | 0 |
| 1181442 | 2022 | 2/4/2022 | COFFEE, BEANS | Non-Juice Beverages | Coffee | IMPORT | Colombia | Non-Detect | 1 | 0 |
| 1181646 | 2022 | 2/8/2022 | COFFEE, BEANS | Non-Juice Beverages | Coffee | IMPORT | Vietnam | Non-Detect | 1 | 0 |
| 1181659 | 2022 | 2/8/2022 | WHEAT FLOUR, ENRICHED (ALL PURPOSE) | Grains and Grain Products | Wheat | IMPORT | Korea (the Republic of) | Non-Detect | 1 | 0 |
| 1175474 | 2022 | 2/10/2022 | RICE CEREAL (BABY) | Baby Food Products | Baby Cereals | DOMESTIC | United States | Non-Detect | 2.5 | 0 |
| 1175475 | 2022 | 2/10/2022 | WHEAT CEREAL (BABY) | Baby Food Products | Baby Cereals | DOMESTIC | United States | Non-Detect | 2.5 | 0 |
| 1181985 | 2022 | 2/10/2022 | COFFEE, BEANS | Non-Juice Beverages | Coffee | IMPORT | Costa Rica | Non-Detect | 2.5 | 0 |
| 1181878 | 2022 | 2/10/2022 | BARLEY, WHOLE GRAIN | Grains and Grain Products | Barley | IMPORT | Israel | Non-Detect | 0.233 | 0 |
| 1181976 | 2022 | 2/11/2022 | COFFEE, BEANS, DECAFFEINATED | Non-Juice Beverages | Coffee | IMPORT | Tanzania, United Republic Of | Non-Detect | 12.15 | 0 |
| 1182149 | 2022 | 2/14/2022 | COFFEE, BEANS | Non-Juice Beverages | Coffee | IMPORT | Costa Rica | Non-Detect | 2.5 | 0 |
| 1182639 | 2022 | 2/18/2022 | FARRO | Grains and Grain Products | Wheat | IMPORT | Italy | Non-Detect | 1 | 0 |
| 1182649 | 2022 | 2/18/2022 | WHEAT FLOUR, WHOLE | Grains and Grain Products | Wheat | IMPORT | India | Trace | 5 | |
| 1182827 | 2022 | 2/23/2022 | WHEAT FLOUR, ENRICHED (ALL PURPOSE) | Grains and Grain Products | Wheat | IMPORT | India | Non-Detect | 1 | 0 |
| 1182896 | 2022 | 2/23/2022 | COFFEE, BEANS | Non-Juice Beverages | Coffee | IMPORT | Colombia | Non-Detect | 1 | 0 |
| 1182973 | 2022 | 2/24/2022 | OAT FLAKES, ROLLED OATS, PUFFS, KRISPIES, LOOPS READY TO EAT | Processed Food Products | Breakfast Foods | IMPORT | Chile | Non-Detect | 2.5 | 0 |
| 1183152 | 2022 | 2/24/2022 | PINTO BEAN | Beans and Legumes | Pinto Beans | IMPORT | Mexico | Non-Detect | 1.01 | 0 |
| 1175982 | 2022 | 3/1/2022 | FIG, DRIED OR PASTE | Fruit | Dried Figs | DOMESTIC | United States | Positive | 1 | 1.25 |
| 1164910 | 2022 | 3/3/2022 | RICE, CULTIVATED, WHOLE GRAIN | Grains and Grain Products | Rice | DOMESTIC | United States | Non-Detect | 2.5 | 0 |
| 1177540 | 2022 | 3/3/2022 | MALT, BARLEY | Grains and Grain Products | Barley | DOMESTIC | United States | Non-Detect | 1.01 | 0 |
| 1184101 | 2022 | 3/4/2022 | CHANA DAL | Beans and Legumes | Garbanzo Beans | IMPORT | Canada | Non-Detect | 1 | 0 |
| 1183958 | 2022 | 3/7/2022 | WHEAT FLOUR, PLAIN | Grains and Grain Products | Wheat | IMPORT | Korea (the Republic of) | Non-Detect | 2.5 | 0 |
| 1184303 | 2022 | 3/8/2022 | GARBANZO BEAN | Beans and Legumes | Garbanzo Beans | IMPORT | Mexico | Non-Detect | 1 | 0 |
| 1180247 | 2022 | 3/10/2022 | WHEAT FLOUR, WHOLE | Grains and Grain Products | Wheat | DOMESTIC | Unknown | Non-Detect | 0.233 | 0 |
| 1184378 | 2022 | 3/10/2022 | BARLEY, WHOLE GRAIN | Grains and Grain Products | Barley | IMPORT | Poland | Non-Detect | 0.233 | 0 |
| 1169637 | 2022 | 3/10/2022 | OATS, WHOLE GRAIN | Grains and Grain Products | Oats | DOMESTIC | United States | Trace | 5 | |

| | | | | | | | | | | |
|---|---|---|---|---|---|---|---|---|---|---|
| 1184656 | 2022 | 3/14/2022 | AZUFRADO BEAN | Beans and Legumes | Other Beans and Legumes | IMPORT | Mexico | Non-Detect | 0.233 | 0 |
| 1180250 | 2022 | 3/14/2022 | RYE FLOUR | Grains and Grain Products | Rye | DOMESTIC | Unknown | Non-Detect | 1 | 0 |
| 1184714 | 2022 | 3/15/2022 | BEAN, DRIED OR PASTE, N.E.C. (VEGETABLE) | Beans and Legumes | Other Beans and Legumes | IMPORT | India | Non-Detect | 2.5 | 0 |
| 1184716 | 2022 | 3/15/2022 | BEAN, DRIED OR PASTE, N.E.C. (VEGETABLE) | Beans and Legumes | Other Beans and Legumes | IMPORT | India | Non-Detect | 2.5 | 0 |
| 1184854 | 2022 | 3/15/2022 | BLACK BEAN, DRIED OR PASTE | Beans and Legumes | Black Beans | IMPORT | Vietnam | Non-Detect | 2.5 | 0 |
| 1184803 | 2022 | 3/15/2022 | COFFEE, BEANS | Non-Juice Beverages | Coffee | IMPORT | Papua New Guinea | Non-Detect | 0.77 | 0 |
| 1180252 | 2022 | 3/16/2022 | WHEAT FLOUR, WHOLE | Grains and Grain Products | Wheat | DOMESTIC | Unknown | Non-Detect | 2.5 | 0 |
| 1184907 | 2022 | 3/16/2022 | RICE, PROCESSED (PACKAGED) N.E.C. | Grains and Grain Products | Rice | IMPORT | Spain | Non-Detect | 2.5 | 0 |
| 1184967 | 2022 | 3/16/2022 | BEAN, DRIED OR PASTE, N.E.C. (VEGETABLE) | Beans and Legumes | Other Beans and Legumes | IMPORT | Mexico | Non-Detect | 1 | 0 |
| 1185221 | 2022 | 3/18/2022 | COFFEE, INSTANT | Non-Juice Beverages | Coffee | IMPORT | Germany | Non-Detect | 2.5 | 0 |
| 1185361 | 2022 | 3/23/2022 | SALTED CRACKERS | Processed Food Products | Other Bakery Products | DOMESTIC | United States | Non-Detect | 0.233 | 0 |
| 1185598 | 2022 | 3/23/2022 | WHEAT FLOUR, ENRICHED SELF-RISING | Grains and Grain Products | Wheat | IMPORT | Ecuador | Non-Detect | 0.233 | 0 |
| 1182604 | 2022 | 3/23/2022 | GINGER, GROUND, CRACKED (SPICE) | Spices | Ginger | IMPORT | India | Non-Detect | 1 | 0 |
| 1186520 | 2022 | 3/25/2022 | COFFEE, BEANS | Non-Juice Beverages | Coffee | IMPORT | Turkey | Non-Detect | 1 | 0 |
| 1186085 | 2022 | 3/29/2022 | SEMOLINA, WHEAT | Grains and Grain Products | Wheat | IMPORT | France | Non-Detect | 0.233 | 0 |
| 1186778 | 2022 | 3/29/2022 | CAPSICUMS (CAYENNE CHILI, HOT PEPPERS), GROUND, CRACKED (SPICE) | Spices | Capsicums, including Paprika | IMPORT | India | Non-Detect | 1 | 0 |
| 1173536 | 2022 | 3/31/2022 | OAT FLOUR | Grains and Grain Products | Oats | DOMESTIC | United States | Positive | 1 | 11.16 |
| 1186773 | 2022 | 4/5/2022 | WHEAT FLOUR, ENRICHED (ALL PURPOSE) | Grains and Grain Products | Wheat | IMPORT | Korea (the Republic of) | Non-Detect | 1 | 0 |
| 1186958 | 2022 | 4/6/2022 | WHEAT FLOUR, WHOLE | Grains and Grain Products | Wheat | DOMESTIC | United States | Non-Detect | 1 | 0 |
| 1187008 | 2022 | 4/6/2022 | OATMEAL, REGULAR, FRUIT OR SPICE ADDED, QUICK OR INSTANT COOKING | Processed Food Products | Breakfast Foods | IMPORT | Finland | Non-Detect | 1 | 0 |
| 1116479 | 2022 | 4/12/2022 | WHEAT FLOUR, WHOLE | Grains and Grain Products | Wheat | DOMESTIC | United States | Non-Detect | 0.233 | 0 |
| 1167316 | 2022 | 4/12/2022 | BUCKWHEAT, WHOLE GRAIN | Grains and Grain Products | Buckwheat | DOMESTIC | United States | Non-Detect | 0.233 | 0 |
| 1120499 | 2022 | 4/12/2022 | WHEAT, WHOLE GRAIN | Grains and Grain Products | Wheat | DOMESTIC | United States | Non-Detect | 1 | 0 |
| 1187709 | 2022 | 4/13/2022 | MALT, BARLEY | Grains and Grain Products | Barley | IMPORT | Czech Republic | Non-Detect | 1 | 0 |
| 1184749 | 2022 | 4/14/2022 | RYE FLOUR | Grains and Grain Products | Rye | DOMESTIC | Unknown | Non-Detect | 0.233 | 0 |
| 1187055 | 2022 | 4/14/2022 | PISTACHIO, IN SHELL | Nuts and Edible Seeds | Pistachios | DOMESTIC | United States | Non-Detect | 1 | 0 |
| 1188402 | 2022 | 4/19/2022 | RAISINS, DRIED OR PASTE | Fruit | Raisins | IMPORT | Afghanistan | Non-Detect | 1 | 0 |
| 1116480 | 2022 | 4/20/2022 | WHEAT FLOUR, WHOLE | Grains and Grain Products | Wheat | DOMESTIC | United States | Non-Detect | 2.5 | 0 |
| 1175801 | 2022 | 4/20/2022 | PISTACHIO, SHELLED | Nuts and Edible Seeds | Pistachios | DOMESTIC | United States | Non-Detect | 1 | 0 |
| 1187116 | 2022 | 4/20/2022 | PISTACHIO, IN SHELL | Nuts and Edible Seeds | Pistachios | DOMESTIC | Unknown | Non-Detect | 1 | 0 |
| 1184746 | 2022 | 4/21/2022 | PISTACHIO, IN SHELL | Nuts and Edible Seeds | Pistachios | DOMESTIC | Unknown | Non-Detect | 2.5 | 0 |
| 1188308 | 2022 | 4/25/2022 | PISTACHIO, IN SHELL | Nuts and Edible Seeds | Pistachios | DOMESTIC | United States | Non-Detect | 2.5 | 0 |
| 1188816 | 2022 | 4/25/2022 | BLACK BEAN, DRIED OR PASTE | Beans and Legumes | Black Beans | IMPORT | Mexico | Non-Detect | 0.233 | 0 |
| 1157294 | 2022 | 4/26/2022 | OAT CEREAL (BABY) | Baby Food Products | Baby Cereals | DOMESTIC | United States | Non-Detect | 0.233 | 0 |
| 1162339 | 2022 | 4/26/2022 | PINTO BEAN | Beans and Legumes | Pinto Beans | DOMESTIC | United States | Non-Detect | 2.05 | 0 |
| 1189144 | 2022 | 4/27/2022 | OATS, WHOLE GRAIN | Grains and Grain Products | Oats | IMPORT | Ireland | Non-Detect | 1.02 | 0 |
| 1189261 | 2022 | 4/28/2022 | COFFEE, BEANS | Non-Juice Beverages | Coffee | IMPORT | Colombia | Non-Detect | 2.5 | 0 |
| 1189610 | 2022 | 4/29/2022 | COFFEE, BEANS | Non-Juice Beverages | Coffee | IMPORT | Kenya | Non-Detect | 1 | 0 |
| 1189613 | 2022 | 4/29/2022 | COFFEE, BEANS | Non-Juice Beverages | Coffee | IMPORT | Rwanda | Non-Detect | 1 | 0 |
| 1189623 | 2022 | 4/29/2022 | PINTO BEAN | Beans and Legumes | Pinto Beans | IMPORT | Mexico | Non-Detect | 1 | 0 |
| 1158673 | 2022 | 5/2/2022 | SOY BASE FORMULA PRODUCT, READY TO FEED | Baby Food Products | Baby Formula | DOMESTIC | Unknown | Non-Detect | 1 | 0 |
| 1189630 | 2022 | 5/2/2022 | OTHER FRUIT AND FRUIT PRODUCTS, DRIED AND PASTE, N.E.C. | Fruit | Other Dried Fruits | IMPORT | Mexico | Non-Detect | 1 | 0 |
| 1189960 | 2022 | 5/4/2022 | KIDNEY BEAN, DRIED OR PASTE | Beans and Legumes | Other Beans and Legumes | IMPORT | Thailand | Non-Detect | 2.5 | 0 |
| 1154765 | 2022 | 5/4/2022 | BARLEY, WHOLE GRAIN | Grains and Grain Products | Barley | DOMESTIC | United States | Non-Detect | 1.03 | 0 |
| 1177481 | 2022 | 5/4/2022 | OAT CEREAL (BABY) | Baby Food Products | Baby Cereals | DOMESTIC | United States | Non-Detect | 1.03 | 0 |
| 1189962 | 2022 | 5/5/2022 | KIDNEY BEAN, DRIED OR PASTE | Beans and Legumes | Other Beans and Legumes | IMPORT | Thailand | Non-Detect | 2.5 | 0 |
| 1189964 | 2022 | 5/5/2022 | BLACK BEAN, DRIED OR PASTE | Beans and Legumes | Black Beans | IMPORT | Thailand | Non-Detect | 2.5 | 0 |
| 1190090 | 2022 | 5/6/2022 | CORN CHIPS, FRIED | Processed Food Products | Other Corn Products | IMPORT | Mexico | Non-Detect | 0.233 | 0 |
| 1190423 | 2022 | 5/10/2022 | COFFEE, BEANS | Non-Juice Beverages | Coffee | IMPORT | Guatemala | Non-Detect | 2.5 | 0 |
| 1190936 | 2022 | 5/13/2022 | MALT, BARLEY | Grains and Grain Products | Barley | IMPORT | United Kingdom | Non-Detect | 1 | 0 |
| 1191012 | 2022 | 5/13/2022 | MALT, BARLEY | Grains and Grain Products | Barley | IMPORT | Denmark | Non-Detect | 1 | 0 |
| 1190995 | 2022 | 5/16/2022 | COFFEE, BEANS | Non-Juice Beverages | Coffee | IMPORT | Nicaragua | Non-Detect | 1 | 0 |
| 1191164 | 2022 | 5/16/2022 | BLACKEYE PEAS, DRIED OR PASTE | Beans and Legumes | Blackeye Peas | IMPORT | Belize | Non-Detect | 1 | 0 |
| 1191439 | 2022 | 5/18/2022 | COFFEE, BEANS | Non-Juice Beverages | Coffee | IMPORT | Guatemala | Non-Detect | 1 | 0 |
| 1182033 | 2022 | 5/19/2022 | PISTACHIO, IN SHELL | Nuts and Edible Seeds | Pistachios | DOMESTIC | United States | Non-Detect | 1 | 0 |
| 1191746 | 2022 | 5/23/2022 | TURMERIC, GROUND, CRACKED (SPICE) | Spices | Turmeric | IMPORT | India | Positive | 2.5 | 19.13 |
| 1191851 | 2022 | 5/24/2022 | RAISINS, DRIED OR PASTE | Fruit | Raisins | IMPORT | South Africa | Non-Detect | 2.5 | 0 |
| 1189823 | 2022 | 5/24/2022 | WHEAT FLOUR, WHOLE | Grains and Grain Products | Wheat | DOMESTIC | United States | Non-Detect | 0.233 | 0 |
| 1189824 | 2022 | 5/24/2022 | WHEAT FLOUR, WHOLE | Grains and Grain Products | Wheat | DOMESTIC | United States | Non-Detect | 0.233 | 0 |
| 1191860 | 2022 | 5/24/2022 | FLOURS AND MEALS N.E.C. | Grains and Grain Products | Other Grains | IMPORT | Mexico | Trace | 5 | |
| 1152170 | 2022 | 5/26/2022 | WHEAT FLOUR, WHOLE | Grains and Grain Products | Wheat | DOMESTIC | United States | Non-Detect | 1 | 0 |
| 1192466 | 2022 | 6/1/2022 | RAISINS, DRIED OR PASTE | Fruit | Raisins | IMPORT | South Africa | Positive | 1 | 5.249 |
| 1192785 | 2022 | 6/3/2022 | COFFEE, BEANS | Non-Juice Beverages | Coffee | IMPORT | Costa Rica | Non-Detect | 2.5 | 0 |
| 1191522 | 2022 | 6/3/2022 | TURMERIC, GROUND, CRACKED (SPICE) | Spices | Turmeric | IMPORT | Nigeria | Positive | 2.5 | 14.03 |
| 1193114 | 2022 | 6/7/2022 | MALT, BARLEY | Grains and Grain Products | Barley | IMPORT | United Kingdom | Non-Detect | 2.5 | 0 |
| 1193126 | 2022 | 6/7/2022 | MALT, BARLEY | Grains and Grain Products | Barley | IMPORT | United Kingdom | Non-Detect | 2.5 | 0 |
| 1187443 | 2022 | 6/9/2022 | COFFEE, BEANS | Non-Juice Beverages | Coffee | IMPORT | Honduras | Non-Detect | 1 | 0 |
| 1193490 | 2022 | 6/13/2022 | WHEAT FLOUR, DURUM | Grains and Grain Products | Wheat | IMPORT | Italy | Non-Detect | 2.5 | 0 |
| 1186932 | 2022 | 6/21/2022 | WHEAT, WHOLE GRAIN | Grains and Grain Products | Wheat | DOMESTIC | United States | Non-Detect | 0.233 | 0 |
| 1194040 | 2022 | 6/23/2022 | WHEAT FLOUR, WHOLE | Grains and Grain Products | Wheat | DOMESTIC | Unknown | Non-Detect | 2.5 | 0 |
| 1194284 | 2022 | 6/23/2022 | COFFEE, BEANS | Non-Juice Beverages | Coffee | IMPORT | Colombia | Non-Detect | 2.5 | 0 |
| 1194318 | 2022 | 6/23/2022 | COFFEE, BEANS | Non-Juice Beverages | Coffee | IMPORT | Honduras | Non-Detect | 2.5 | 0 |
| 1194322 | 2022 | 6/23/2022 | COFFEE, BEANS | Non-Juice Beverages | Coffee | IMPORT | Honduras | Non-Detect | 2.5 | 0 |
| 1195159 | 2022 | 6/23/2022 | SPAGHETTI | Processed Food Products | Pasta | IMPORT | Romania | Non-Detect | 2.5 | 0 |
| 1194939 | 2022 | 6/24/2022 | COFFEE, BEANS | Non-Juice Beverages | Coffee | IMPORT | Taiwan | Non-Detect | 9.225 | 0 |
| 1194651 | 2022 | 6/27/2022 | COFFEE, BEANS | Non-Juice Beverages | Coffee | IMPORT | India | Non-Detect | 1 | 0 |
| 1194741 | 2022 | 6/28/2022 | MALT, BARLEY | Grains and Grain Products | Barley | IMPORT | United Kingdom | Non-Detect | 1 | 0 |
| 1195267 | 2022 | 7/5/2022 | COFFEE, BEANS | Non-Juice Beverages | Coffee | IMPORT | Guatemala | Non-Detect | 2.5 | 0 |
| 1184929 | 2022 | 7/5/2022 | BARLEY, WHOLE GRAIN | Grains and Grain Products | Barley | DOMESTIC | United States | Non-Detect | 1 | 0 |
| 1184930 | 2022 | 7/5/2022 | BUCKWHEAT, WHOLE GRAIN | Grains and Grain Products | Buckwheat | DOMESTIC | United States | Non-Detect | 1 | 0 |
| 1191925 | 2022 | 7/7/2022 | PINTO BEAN | Beans and Legumes | Pinto Beans | DOMESTIC | United States | Non-Detect | 1 | 0 |
| 1195581 | 2022 | 7/8/2022 | RAISINS, DRIED OR PASTE | Fruit | Raisins | IMPORT | South Africa | Non-Detect | 1 | 0 |
| 1195585 | 2022 | 7/8/2022 | RAISINS, DRIED OR PASTE | Fruit | Raisins | IMPORT | Argentina | Non-Detect | 1 | 0 |
| 1195679 | 2022 | 7/11/2022 | MUNG BEAN, DRIED OR PASTE | Beans and Legumes | Other Beans and Legumes | IMPORT | India | Non-Detect | 1 | 0 |
| 1195689 | 2022 | 7/11/2022 | RICE, CULTIVATED, WHOLE GRAIN | Grains and Grain Products | Rice | IMPORT | Thailand | Non-Detect | 1 | 0 |
| 1194209 | 2022 | 7/12/2022 | WHEAT, WHOLE GRAIN | Grains and Grain Products | Wheat | DOMESTIC | United States | Non-Detect | 0.233 | 0 |
| 1196126 | 2022 | 7/14/2022 | NUTS OR EDIBLE SEEDS NOT MENTIONED ELSEWHERE, N.E.C. | Nuts and Edible Seeds | Other Nuts and Edible Seeds | IMPORT | India | Non-Detect | 1 | 0 |
| 1196339 | 2022 | 7/18/2022 | COFFEE, BEANS | Non-Juice Beverages | Coffee | IMPORT | Colombia | Non-Detect | 0.225 | 0 |

| | | | | | | | | | | |
|---|---|---|---|---|---|---|---|---|---|---|
| 1196557 | 2022 | 7/20/2022 | KIDNEY BEAN | Beans and Legumes | Other Beans and Legumes | IMPORT | Nicaragua | Non-Detect | 2.5 | 0 |
| 1196697 | 2022 | 7/21/2022 | KIDNEY BEAN, BEAN AND PEA SPROUTS | Beans and Legumes | Other Beans and Legumes | IMPORT | Nicaragua | Non-Detect | 2.5 | 0 |
| 1197250 | 2022 | 7/27/2022 | COFFEE, BEANS | Non-Juice Beverages | Coffee | IMPORT | Nicaragua | Non-Detect | 12.15 | 0 |
| 1197291 | 2022 | 7/28/2022 | CANDY BAR OR PIECES (NOT CARAMEL OR FONDANT), SOFT, WITH FRUIT (WITHOUT CHOCOLATE) | Candy and Chocolate Products | Candy with Fruit | IMPORT | Canada | Non-Detect | 2.5 | 0 |
| 1194136 | 2022 | 8/2/2022 | LIMA BEAN | Beans and Legumes | Other Beans and Legumes | DOMESTIC | United States | Non-Detect | 2.5 | 0 |
| 1197768 | 2022 | 8/3/2022 | OAT FLAKES, ROLLED OATS, PUFFS, KRISPIES, LOOPS READY TO EAT | Processed Food Products | Breakfast Foods | IMPORT | Canada | Non-Detect | 2.5 | 0 |
| 1197778 | 2022 | 8/3/2022 | OAT FLAKES, ROLLED OATS, PUFFS, KRISPIES, LOOPS READY TO EAT | Processed Food Products | Breakfast Foods | IMPORT | Canada | Non-Detect | 2.5 | 0 |
| 1197783 | 2022 | 8/3/2022 | OAT FLAKES, ROLLED OATS, PUFFS, KRISPIES, LOOPS READY TO EAT | Processed Food Products | Breakfast Foods | IMPORT | Canada | Non-Detect | 2.5 | 0 |
| 1197788 | 2022 | 8/3/2022 | OAT FLAKES, ROLLED OATS, PUFFS, KRISPIES, LOOPS READY TO EAT | Processed Food Products | Breakfast Foods | IMPORT | Canada | Non-Detect | 2.5 | 0 |
| 1109058 | 2022 | 8/4/2022 | OAT FLOUR | Grains and Grain Products | Oats | IMPORT | Dominican Republic (the) | Non-Detect | 2.5 | 0 |
| 1197991 | 2022 | 8/4/2022 | COFFEE, BEANS | Non-Juice Beverages | Coffee | IMPORT | Guatemala | Non-Detect | 2.5 | 0 |
| 1197946 | 2022 | 8/4/2022 | OAT FLAKES, ROLLED OATS, PUFFS, KRISPIES, LOOPS READY TO EAT | Processed Food Products | Breakfast Foods | IMPORT | Canada | Positive | 2.5 | 3.54 |
| 1174207 | 2022 | 8/9/2022 | RICE CEREAL (BABY) | Baby Food Products | Baby Cereals | DOMESTIC | United States | Non-Detect | 1 | 0 |
| 1198699 | 2022 | 8/15/2022 | OATMEAL, REGULAR, FRUIT OR SPICE ADDED, QUICK OR INSTANT COOKING | Processed Food Products | Breakfast Foods | IMPORT | China | Non-Detect | 2.5 | 0 |
| 1196949 | 2022 | 8/16/2022 | PISTACHIO, IN SHELL | Nuts and Edible Seeds | Pistachios | DOMESTIC | United States | Non-Detect | 2.5 | 0 |
| 1198734 | 2022 | 8/16/2022 | COFFEE, BEANS | Non-Juice Beverages | Coffee | IMPORT | El Salvador | Non-Detect | 2.5 | 0 |
| 1198772 | 2022 | 8/16/2022 | COFFEE, BEANS | Non-Juice Beverages | Coffee | IMPORT | Costa Rica | Non-Detect | 2.5 | 0 |
| 1198951 | 2022 | 8/17/2022 | RAISINS, DRIED OR PASTE | Fruit | Raisins | IMPORT | Chile | Non-Detect | 2.5 | 0 |
| 1189268 | 2022 | 8/22/2022 | BARLEY, WHOLE GRAIN | Grains and Grain Products | Barley | DOMESTIC | Unknown | Non-Detect | 0.233 | 0 |
| 1149772 | 2022 | 8/22/2022 | WHEAT, WHOLE GRAIN | Grains and Grain Products | Wheat | DOMESTIC | United States | Non-Detect | 1 | 0 |
| 1199470 | 2022 | 8/22/2022 | MIXED BEAN/SEED SPROUTS, BEAN AND PEA SPROUTS | Beans and Legumes | Other Beans and Legumes | IMPORT | Canada | Non-Detect | 1 | 0 |
| 1167321 | 2022 | 8/23/2022 | BUCKWHEAT, WHOLE GRAIN | Grains and Grain Products | Buckwheat | DOMESTIC | United States | Positive | 5 | 16.6 |
| 1199843 | 2022 | 8/25/2022 | WHEAT FLOUR, DURUM | Grains and Grain Products | Wheat | IMPORT | Canada | Non-Detect | 2.5 | 0 |
| 1199935 | 2022 | 8/26/2022 | COFFEE, BEANS | Non-Juice Beverages | Coffee | IMPORT | Nicaragua | Non-Detect | 2.5 | 0 |
| 1200308 | 2022 | 8/30/2022 | BLACK BEAN, DRIED OR PASTE | Beans and Legumes | Black Beans | IMPORT | Canada | Non-Detect | 2.5 | 0 |
| 1195693 | 2022 | 9/1/2022 | PISTACHIO, IN SHELL | Nuts and Edible Seeds | Pistachios | DOMESTIC | United States | Non-Detect | 1 | 0 |
| 1200583 | 2022 | 9/1/2022 | COFFEE, BEANS | Non-Juice Beverages | Coffee | IMPORT | Guatemala | Non-Detect | 1 | 0 |
| 1200750 | 2022 | 9/6/2022 | COFFEE, BEANS | Non-Juice Beverages | Coffee | IMPORT | Honduras | Non-Detect | 2.5 | 0 |
| 1194519 | 2022 | 9/8/2022 | BEAN, DRIED OR PASTE, N.E.C. (VEGETABLE) | Beans and Legumes | Other Beans and Legumes | DOMESTIC | United States | Non-Detect | 2.5 | 0 |
| 1183622 | 2022 | 9/21/2022 | PINTO BEAN | Beans and Legumes | Pinto Beans | DOMESTIC | United States | Non-Detect | 2.5 | 0 |
| 1183623 | 2022 | 9/21/2022 | BLACK EYE BEANS | Beans and Legumes | Blackeye Peas | DOMESTIC | United States | Non-Detect | 2.5 | 0 |
| 1120515 | 2022 | 9/21/2022 | CORN, BOLTED MEAL OR FLOUR | Grains and Grain Products | Corn | DOMESTIC | United States | Non-Detect | 0.233 | 0 |
| 1187371 | 2022 | 9/22/2022 | BLACK EYE BEANS | Beans and Legumes | Blackeye Peas | DOMESTIC | United States | Non-Detect | 0.233 | 0 |
| 1202376 | 2022 | 9/22/2022 | PISTACHIO, IN SHELL | Nuts and Edible Seeds | Pistachios | IMPORT | Turkey | Non-Detect | 1 | 0 |
| 1202612 | 2022 | 9/26/2022 | BUCKWHEAT, WHOLE GRAIN | Grains and Grain Products | Buckwheat | IMPORT | Russia | Non-Detect | 1 | 0 |
| 1202912 | 2022 | 9/29/2022 | COFFEE, BEANS | Non-Juice Beverages | Coffee | IMPORT | Brazil | Non-Detect | 1 | 0 |
